# Author Correction: Understanding the genetic determinants of the brain with MOSTest

**DOI:** 10.1038/s41467-020-18628-w

**Published:** 2020-09-14

**Authors:** Dennis van der Meer, Oleksandr Frei, Tobias Kaufmann, Alexey A. Shadrin, Anna Devor, Olav B. Smeland, Wesley K. Thompson, Chun Chieh Fan, Dominic Holland, Lars T. Westlye, Ole A. Andreassen, Anders M. Dale

**Affiliations:** 1 NORMENT Centre, Division of Mental Health and Addiction, Oslo University Hospital & Institute of Clinical Medicine, University of Oslo, Oslo, Norway; 2 School of Mental Health and Neuroscience, Faculty of Health, Medicine and Life Sciences, Maastricht University, Maastricht, The Netherlands; 3 Center for Bioinformatics, Department of Informatics, University of Oslo, Oslo, Norway; 4 Departments of Neurosciences and Radiology, University of California at San Diego, La Jolla, CA 92037 USA; 5 Martinos Center for Biomedical Imaging, MGH/HMS, Charlestown, MA 02129 USA; 6 Department of Family Medicine and Public Health, University of California at San Diego, La Jolla, CA 92037 USA; 7 Center for Multimodal Imaging and Genetics, University of California at San Diego, La Jolla, CA 92037 USA; 8 Department of Psychology, University of Oslo, Oslo, Norway

**Keywords:** Genome-wide association studies, Genetics of the nervous system

Correction to: *Nature Communications* 10.1038/s41467-020-17368-1, published online 14 July 2020.

The original version of the Supplementary information associated with this Article contained errors in Supplementary Figs. 2A, 3A, 4A, 5B, 7A, 7B, and 9. The HTML has been updated to include a corrected version of the Supplementary information; the original incorrect versions of these figures can be found as Supplementary information associated with this Correction.

## Supplementary information

Supplementary Information

