# Supplementary material for: Understanding the genetic determinants of the brain with MOSTest
Source: Nat Commun. 2020 Jul 14;11:3512. doi: 10.1038/s41467-020-17368-1 (PMC7360598; doi:10.1038/s41467-020-17368-1)
Supplement: Supplementary file 1 — Supplementary Information [file 41467_2020_17368_MOESM1_ESM.pdf]

Supplementary Information

Supplementary Notes

Morphological features included

Please see Supplementary Table 1 for an overview of all regional morphological features included in the analyses. We included all features outputted by the default Freesurfer subcortical and cortical processing streams, except for the range of global measures, CSF, surface holes, vessels, optic chiasm and hypointensities, as we did not consider these measures of regional brain morphology.

List of discovered loci

Please see Supplementary Table 2 for an overview of the number of whole-genome significant SNPs, the number of independent SNPs, and the number of independent loci (see the Methods for definitions), per test and per feature set. Supplementary Data 1-3 contains an overview of each of the individual loci discovered over all the 171 features combined, by both MOSTest and min-P (92 loci), by MOSTest only (255) and by min-P only (20). These tables further lists for how many and which regions the lead SNPs were significant. Overall, loci discovered by both tests had significant effects on multiple regions, loci discovered by min-P only had effects on 1 or 2 regions, and loci discovered only by MOSTest often had no whole-genome significant effect on any of the regions.

The tables of discovered loci for each of the subsets of features, for both MOSTest and min-P are in Supplementary Data 4-11. These include information on lead SNP, genomic location, significance, and mapped genes, as outputted by FUMA.

Results from the replication

Table 1 lists the results from the replication attempt of the main GWAS via MOSTest and min-P. For this, we used a new batch of neuroimaging data of 4,884 healthy White European UKB

participants that was released in October 2019, after we ran our primary analyses and made the results of this available via bioRxiv. This data was processed identically to the main sample and then analysed through MOSTest and min-P. We subsequently calculated the percentage of loci discovered in the main analyses, per test and per feature set, that was nominally significant in this additional sample. As can be seen in Table 1, for each combination of test and feature set, the replication rate was approximately 40%, indicating no major difference in the replication rates. From this it follows that the absolute number of loci replicating is threefold higher for MOSTest than for min-P.

Supplementary Data 1-3 further lists the replication p-values, by both MOSTest and min-P for each of the specific lead SNPs.

Significant genes and genetic pathways

Please see Supplementary Data tables 12 and 13 for a list of all multiple-comparison corrected significant genes (1034) and GO biological processes pathways (136), as identified by MOSTest on all features combined.

Test for genomic inflation in min-P and MOSTest

We applied LD score regression[33] to test for genomic inflation in summary statistics output of MOSTest and min-P. The highest observed value of the LD score regression intercept across all analyses was around 1.01, indicating that MOSTest and min-P results represent a true polygenic signal, with no signatures of genomic inflation. Full results are shown in Supplementary Table 6.

Validation and formal comparison between MOSTest and MQFAM on real data

We compared the -log10(p-value) for a subset of SNPs, calculated by MQFAM and MOSTest. MQFAM takes 10,000x longer to run (requiring approximately 250K CPU hours, instead of 24 CPU hours with MOSTest), it was therefore infeasible to run the analysis on the entire set of 7.4M SNPs. Instead we tested a set of 356 LD-independent SNPs (a subset of all genome-wide significant SNPs remaining after LD-based clumping with r2=0.6 threshold) with a p-value from min-P below the genome-wide significance threshold. The results, as displayed in Supplementary Figure 10, show a very high correlation (r=0.9976) between MQFAM and MOSTest -log10(p-values), with median of 14.16 (MOSTest) versus 14.40 (MQFAM). Another recently developed multivariate test, aMAT[13], uses the same test statistic as MOSTest, but after applying regularization (spectral filtering) to the correlation matrix R. Regularization was not necessary in our data, as the conditioning number was reasonably low, see Supplementary Table 4, leading to a well-defined matrix R^{-1}.

Rank-based inverse normal transformation

We carried out a rank-based inverse normal transformation of the measures, otherwise non-normally distributed measures can inflate p-values and thus elevate type-I error. Supplementary Figure 11 shows the empirical distribution of MOSTest and min-P test statistics under the null (calculated via permutations), along with p-values calculated from the test, with the rank-based INT, showing correct behaviour.

Supplementary Figure 12 shows the distributions after running MOSTest without the transformation, leading to deviations, highlighting that this transformation is important for maintaining correct type-I error.

A

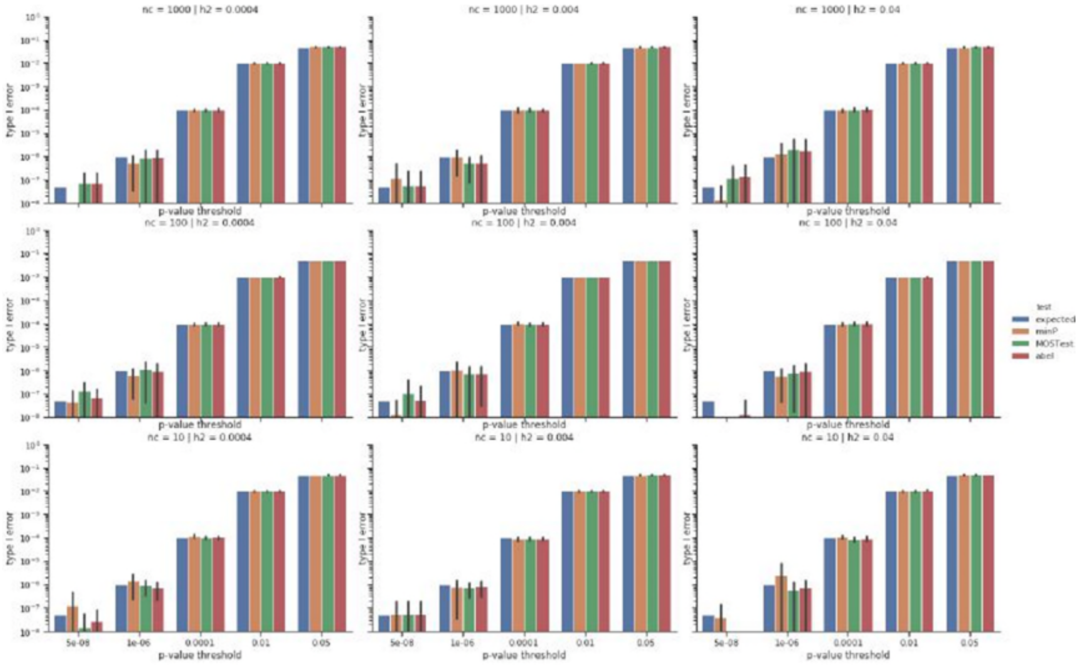

B

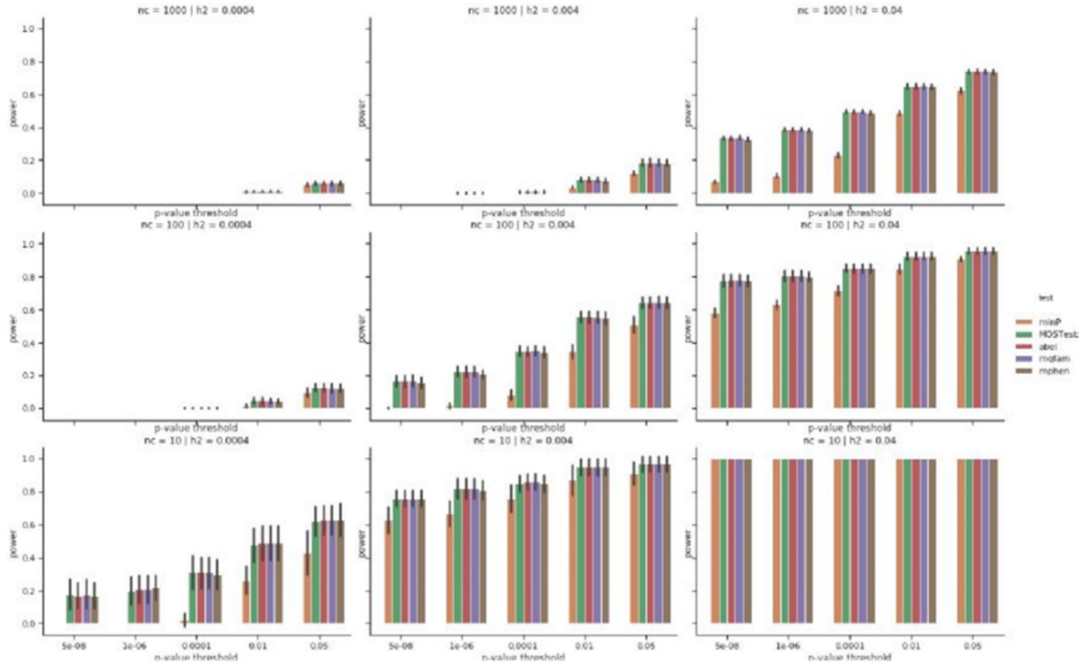

Supplementary Figure 1. Results from simulation of high to low polygenicity (nc), from top to bottom panels, and low to high heritability (h2), from left to right panels.
A) Type-I error on the y-axis (log10 scale), calculated as a fraction of all null SNPs (i.e. SNPs on all chromosomes except chr21), with p-value from a multivariate test falling below a specific significance threshold (5e-8, 1e-6, 1e-4, 0.01 and 0.05) as indicated on the x-axis. **B)** Power to detect non-null effects on the y-axis, calculated as a fraction of causal SNPs from chr21 with p-value falling below significance thresholds on the x-axis. The colours of the bars indicate the multivariate test, as shown in the legend ("abel" corresponds to the results from MultiABEL software, and "mqfam" – to MV-PLINK, also known as "MQFAM"). Each bar visualize an average value, obtained from n=10 independent simulation runs, with error bars corresponding to the standard deviation across runs.

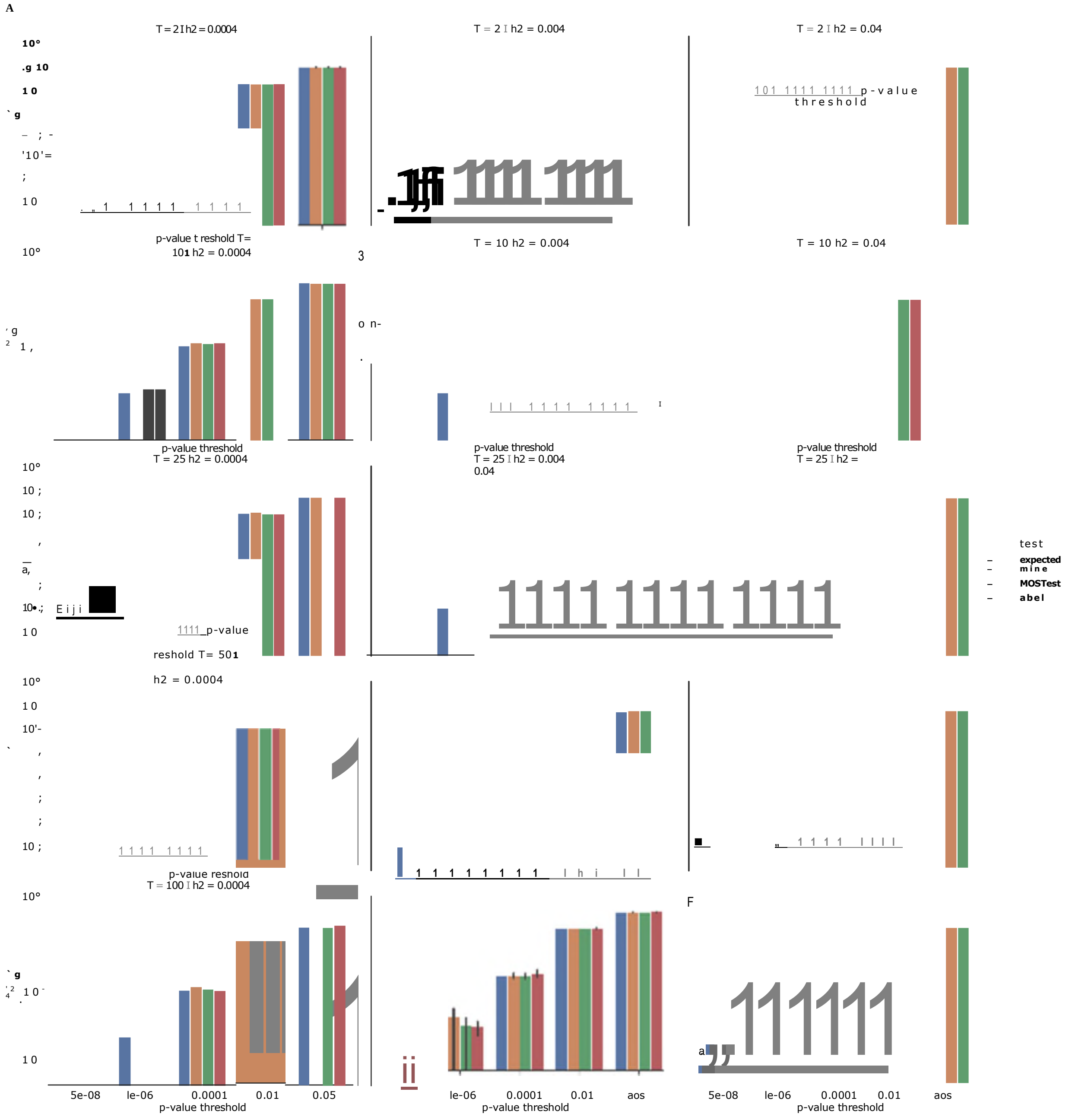
A
T = 2 I h2 = 0.0004
T = 2 I h2 = 0.004
T = 2 I h2 = 0.04
T = 10 h2 = 0.004
T = 10 h2 = 0.04
p-value threshold
T = 25 h2 = 0.0004
T = 25 I h2 = 0.004
h2 = 0.0004
T = 100 I h2 = 0.0004
test
expected
MOSTest
F
5e-08
le-06
0.0001
0.01
0.05
aos

B

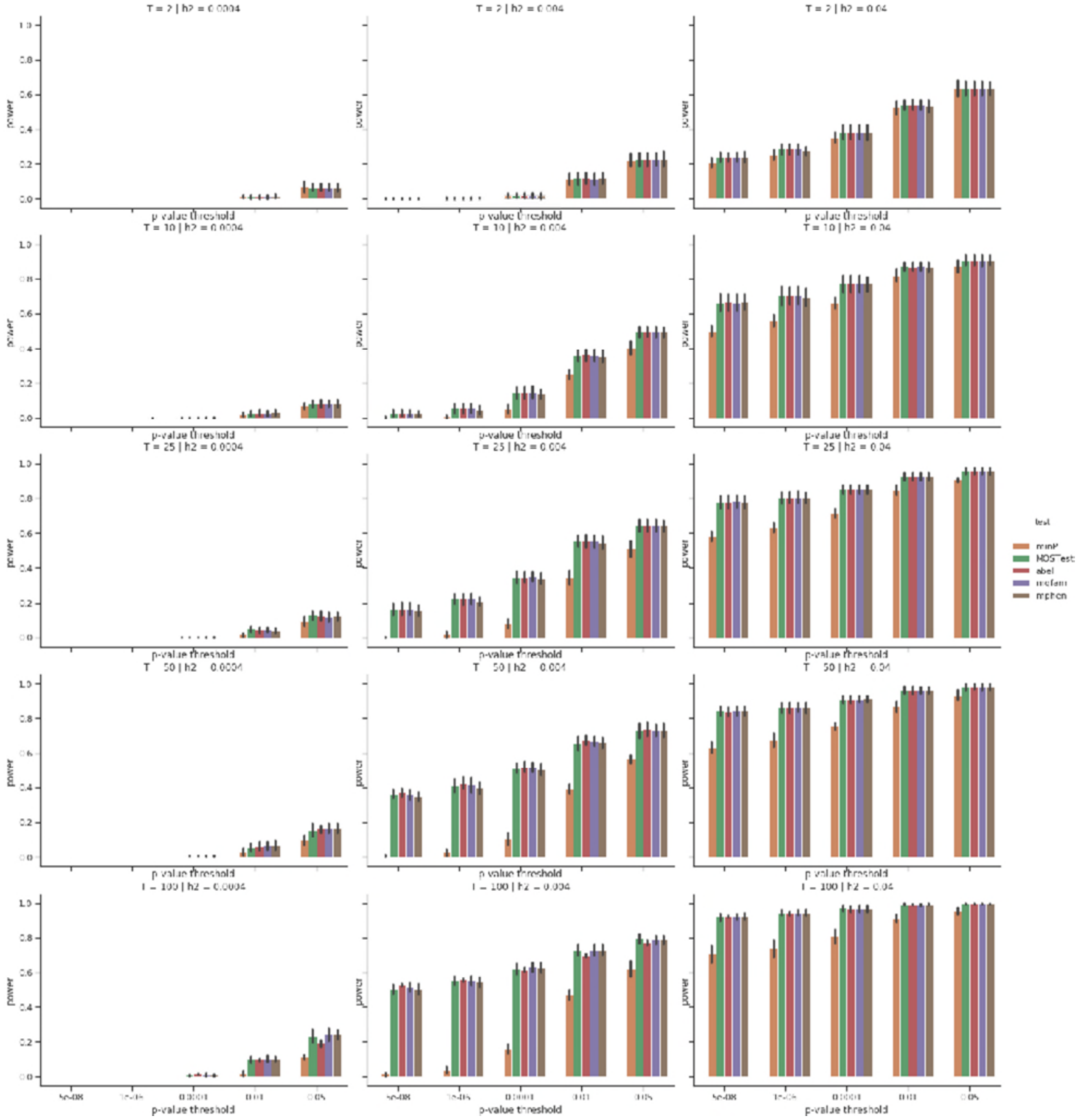

Supplementary Figure 2. Results from simulation of low to high number of total features included (T), from top to bottom panels, and low to high heritability (h2), from left to right panels. A) Type-I error on the y-axis**,** calculated under null (i.e. all chromosomes except chr21), across different p-value thresholds on the x-axis. **B)** Power to detect effects on the y-axis, as calculated from chromosome 21, across different p-value thresholds on the x-axis. The colours of the bars indicate the test, as shown in the legend. Appearance of the data bars and error bars is the same as for the previous figure.

A

tlowill1111111 11111111011.11111

p-val reshold

uethre=nold
T=0ll=10

lll#111whilli0111.140,111111

p-valu res old
T=101,10

ii11O11111: ii01111

p-value
reshold T201l1

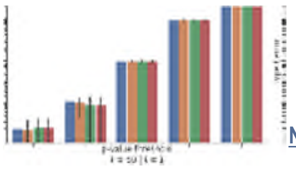

NJ, 011,11111„„0"11111„01111111

T=1001,1 p-value reshold T=1001,2 T=1001,0 p-value reshold T= 100 l = 10 p-value reshold p-value reshold

1:00,„„,„111111F.d$_{o}$gliti.„,1111111F„„,,,1111111

B

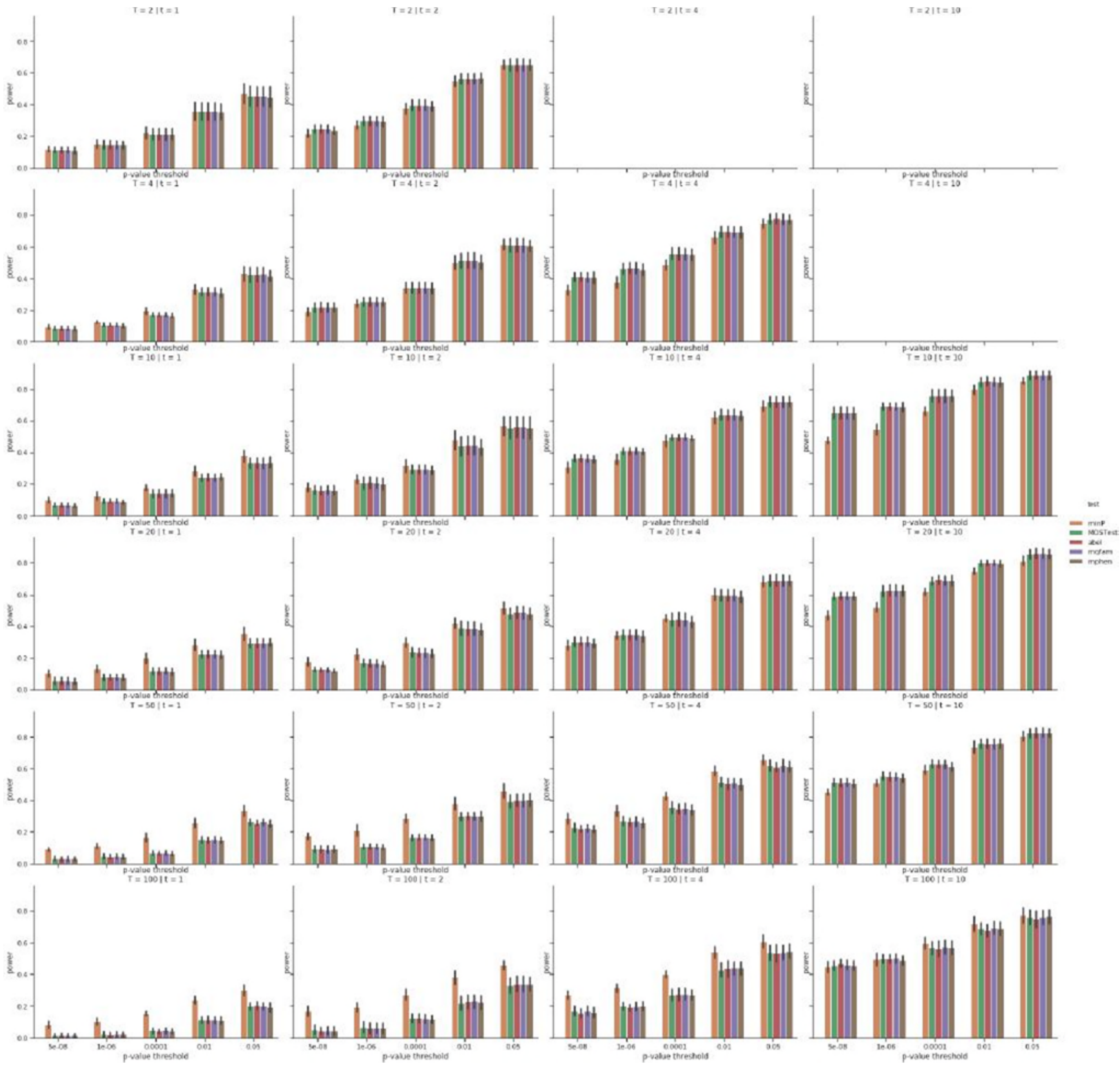

Supplementary Figure 3. Results from simulation of low to high number of total features included (T), from top to bottom panels, and low to high number of features that share genetic effects (t), from left to right panels. A) Type-I error on the y-axis**,** calculated under null (i.e. all chromosomes except chr21), across different p-value thresholds on the x-axis. **B)** Power to detect effects on the y-axis, as calculated from chromosome 21, across different p-value thresholds on the x-axis. The colours of the bars indicate the test, as shown in the legend. Appearance of the data bars and error bars is the same as for the previous figure.

A

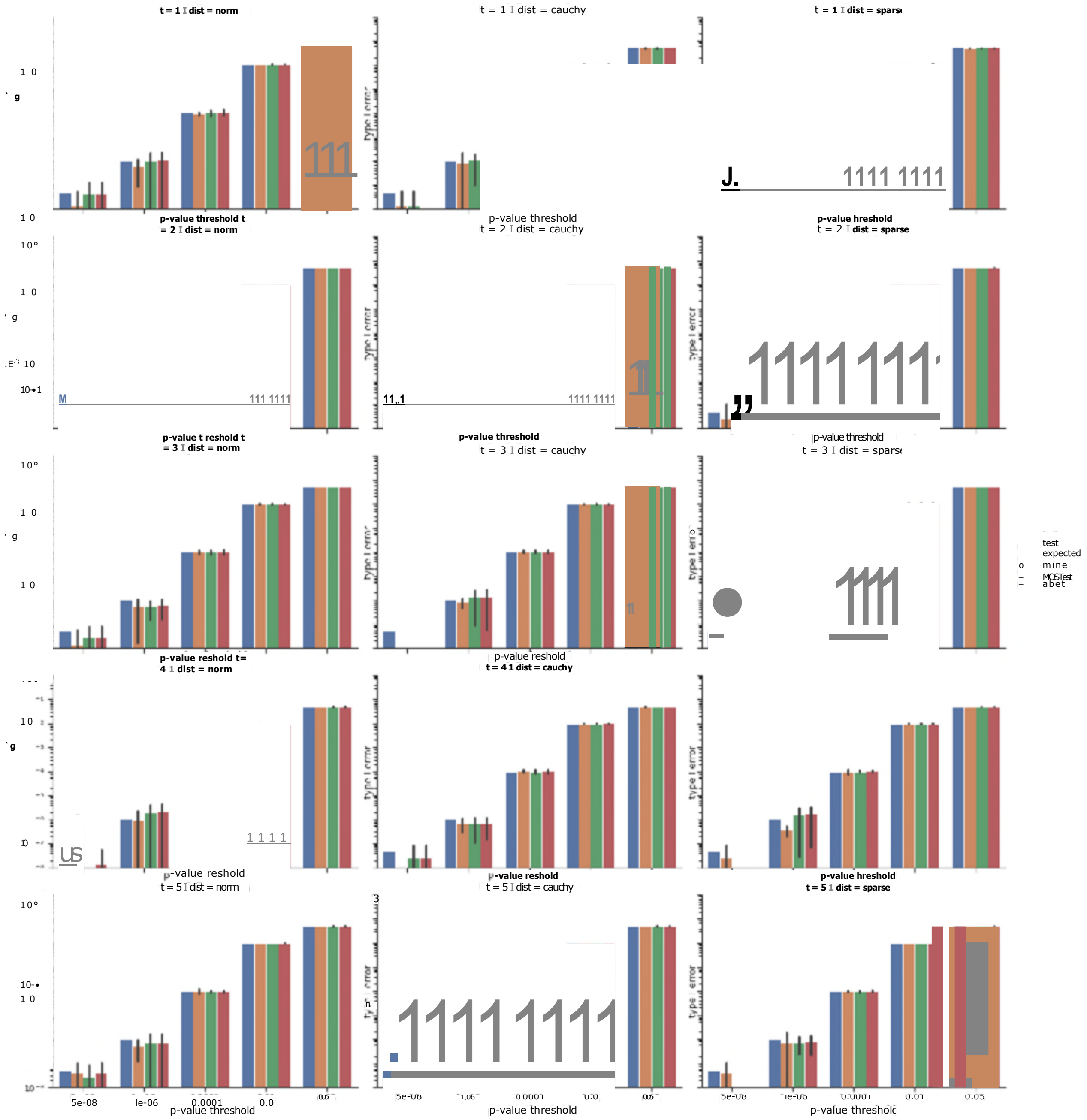
t = 1 | dist = norm
t = 1 | dist = cauchy
t = 1 | dist = sparse
t = 2 | dist = norm
t = 2 | dist = cauchy
t = 2 | dist = sparse
t = 3 | dist = norm
t = 3 | dist = cauchy
t = 3 | dist = sparse
t = 4 | dist = norm
t = 4 | dist = cauchy
t = 5 | dist = norm
t = 5 | dist = cauchy
t = 5 | dist = sparse
p-value threshold
type I error
test
expected
mine
MOSTest
5e-08
1e-06
0.0001
0.01
0.05

B

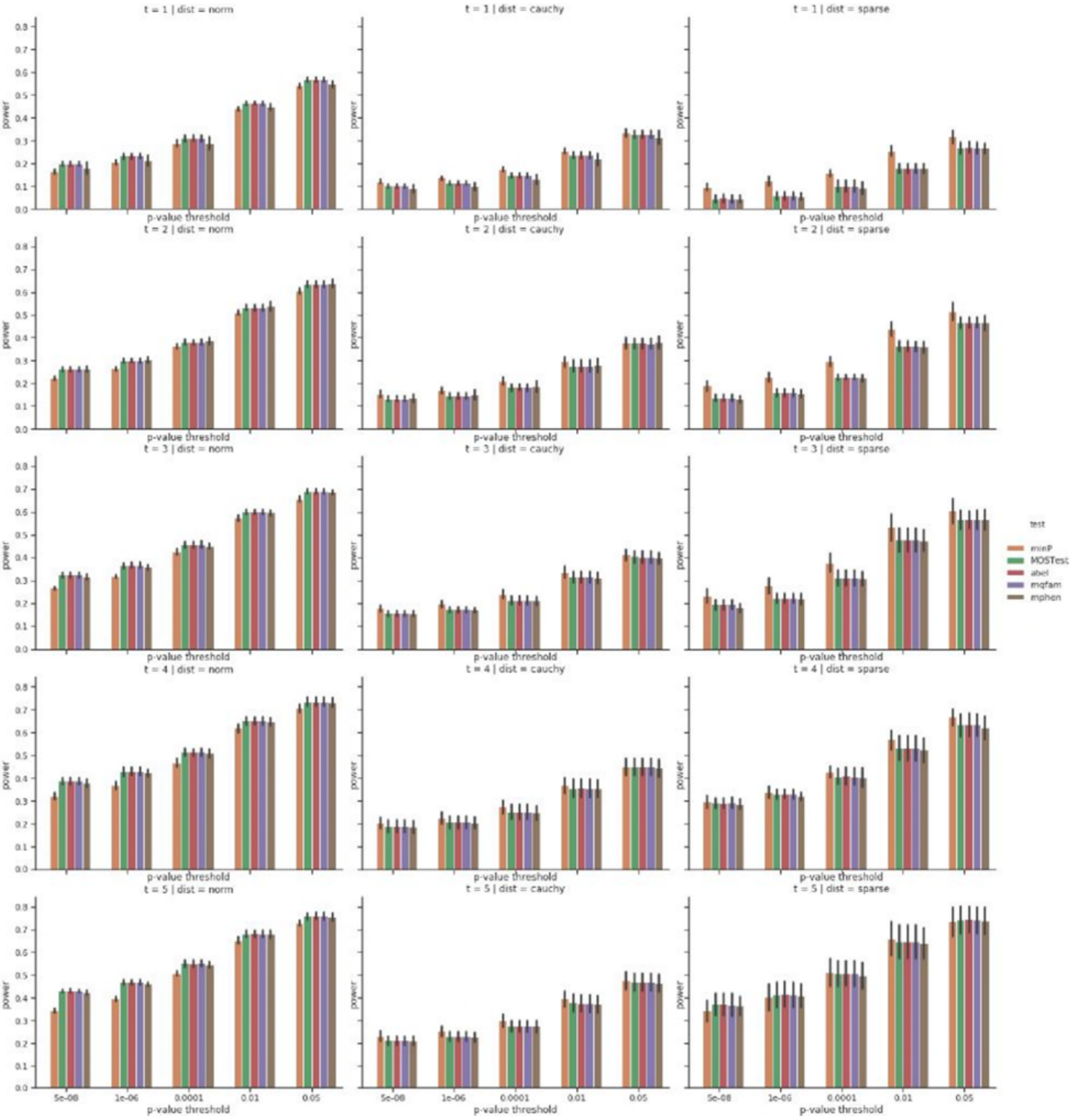

Supplementary Figure 4. Results from simulation of low to high number of features that share genetic effects (t), from top to bottom panels, with the genetic effect sizes having either a normal distribution, a Cauchy distribution, or sparse, from left to right panels. Total number of traits (T) is 25. A) Type-I error on the y-axis**,** calculated under null (i.e. all chromosomes except chr21), across different p-value thresholds on the x-axis. **B)** Power to detect effects on the y-axis, as calculated from chromosome 21, across different p-value thresholds on the x-axis. The colours of the bars indicate the test, as shown in the legend. Appearance of the data bars and error bars is the same as on the previous figure.

A

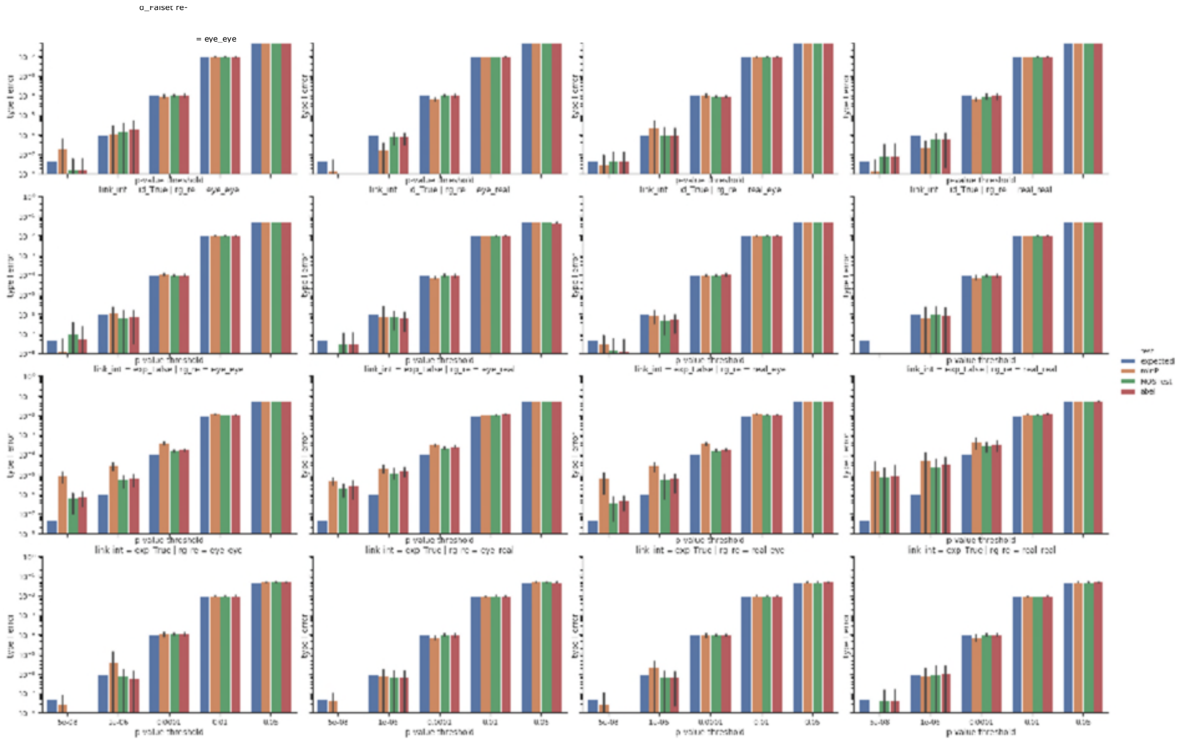

B

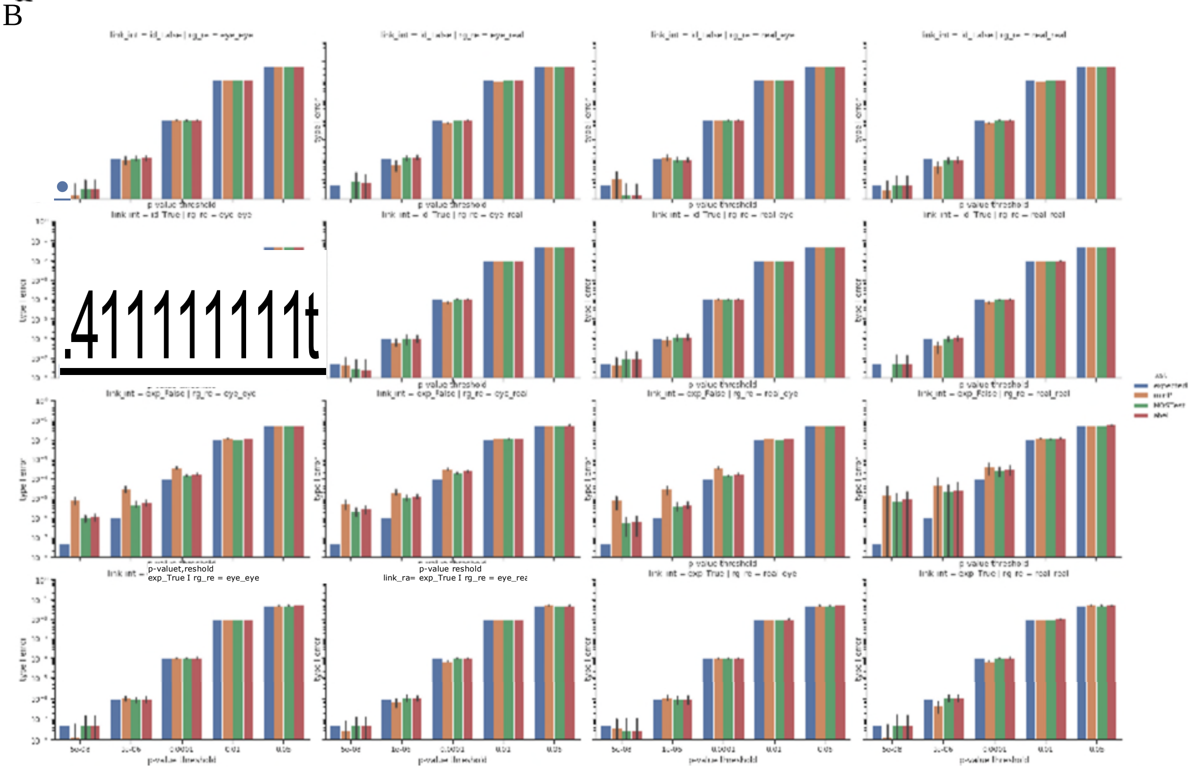

C

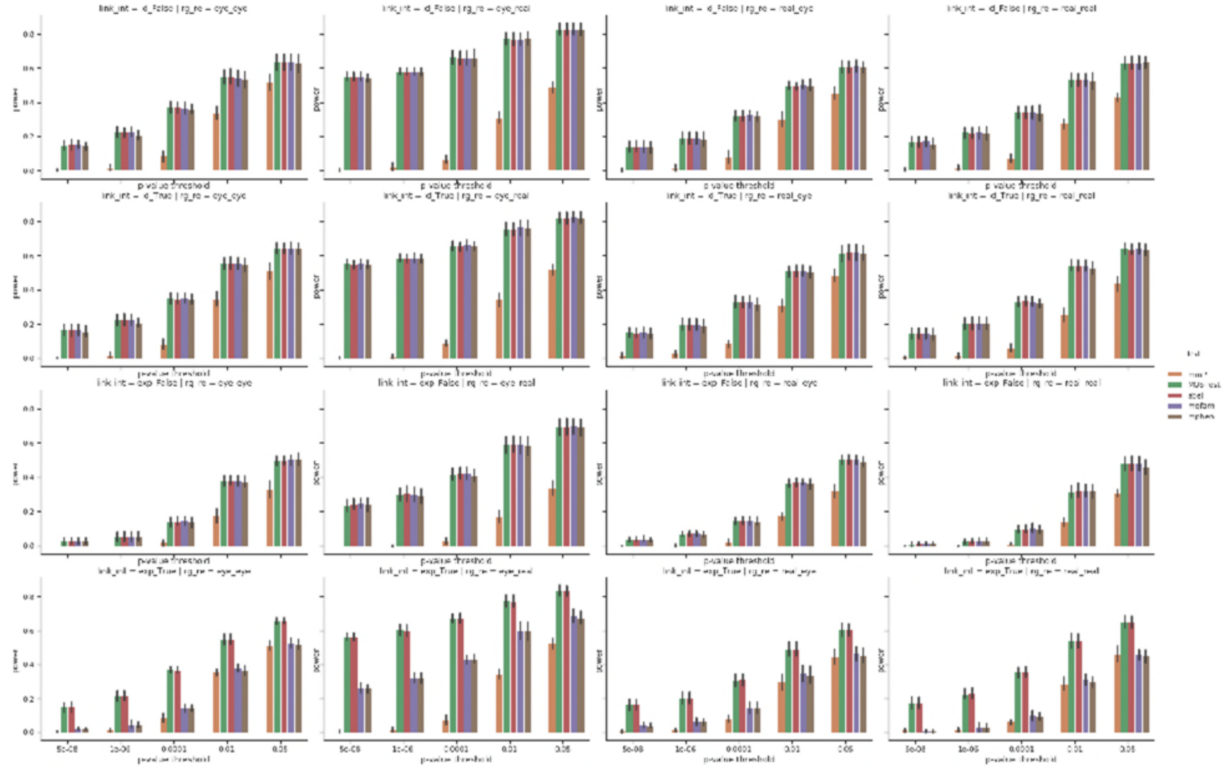

Supplementary Figure 5. Results from simulations across four dimensions: link ("id" or "exp") - link function applied to the phenotypes; int ("T" or "F") - whether to perform rank-based inverse-normal transformation; re ("eye" or "real") - covariance structure of the phenotype residuals; rg ("eye" or "real") - covariance structure of the genetic effects. A) Type-I error on the y-axis**,** calculated under null (i.e. all chromosomes except chr21), across different p-value thresholds on the x-axis. **B)** Type-I error on the y-axis**,** calculated under permutation on all chromosomes, across different p-value thresholds on the x-axis. **C)** Power to detect effects on the y-axis, as calculated from chromosome 21, across different p-value thresholds on the x-axis. The colours of the bars indicate the test, as shown in the legend. Appearance of the data bars and error bars is the same as on the previous figure.

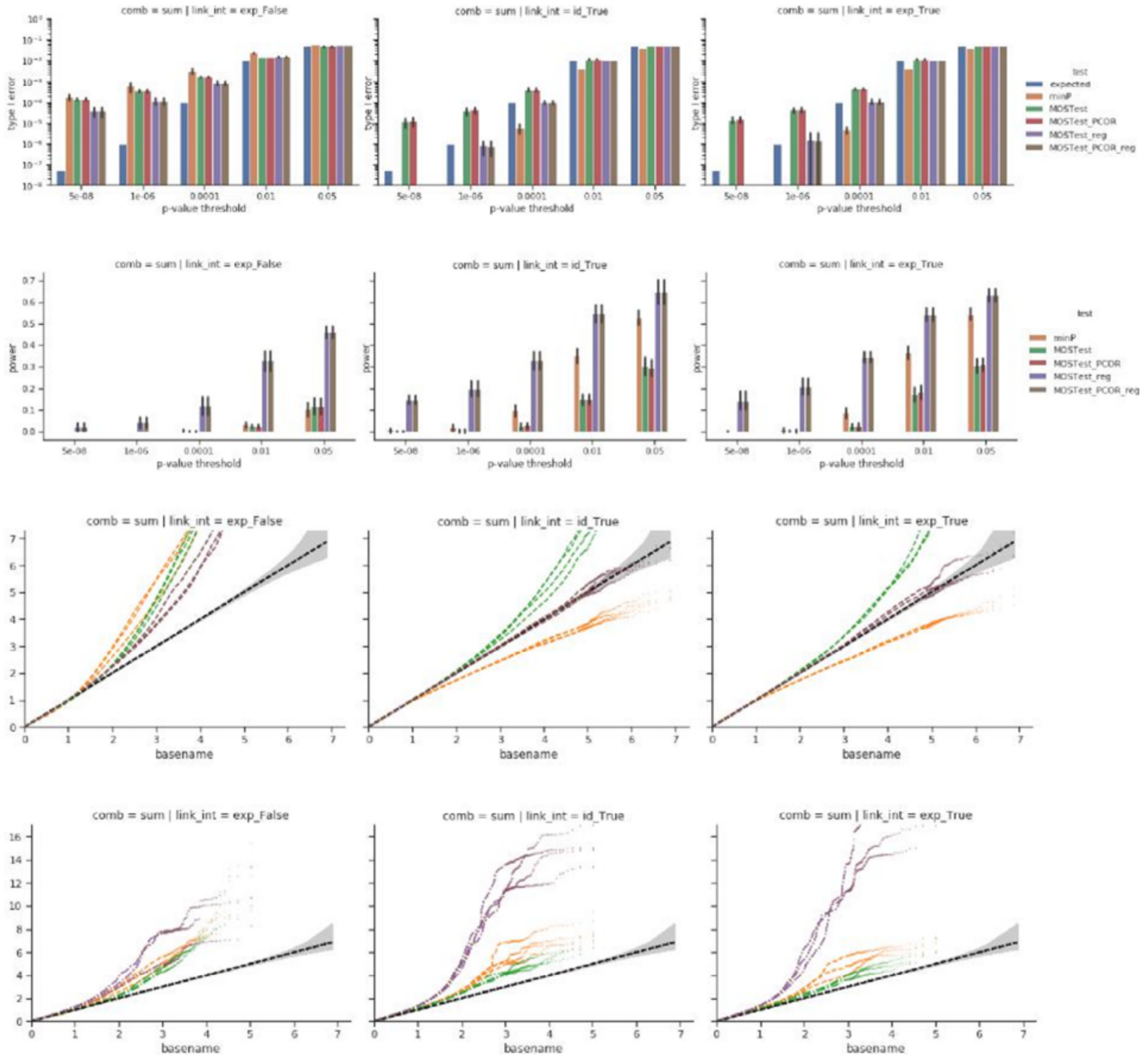

Supplementary Figure 6. Type-I error and power of MOSTest and min-P in the presence of linear combinations across features, and different combinations of "link" and "int" scenarios. Top row shows type-I error calculated under null (i.e. all chromosomes except chromosome 21); second row shows the power to detect effects, on chromosome 21; third row shows QQ plots under null; last row shows QQ plots for SNPs on chr21. "PCOR" indicates that the correlation matrix was calculated from phenotypes, while "GCOR" indicates the default MOSTest setting, which is to use correlation of z-scores under permutation. "reg" indicates that the correlation matrix was regularized by setting the 275 smallest eigenvalues to 0 (total number of features in this analysis is 25*24/2 = 300, i.e. all pairwise combinations of the original 25 features, therefore 275=300-25 gives the optimal number of eigenvalues to regularize). Appearance of the data bars and error bars is the same as on the previous figure. Grey shaded area on the QQ plots (third and last rows) is calculated as 95% confidence interval of the QQ plot curve under null, as described in https://genome.sph.umich.edu/wiki/Code_Sample:_Generating_QQ_Plots_in_R

A

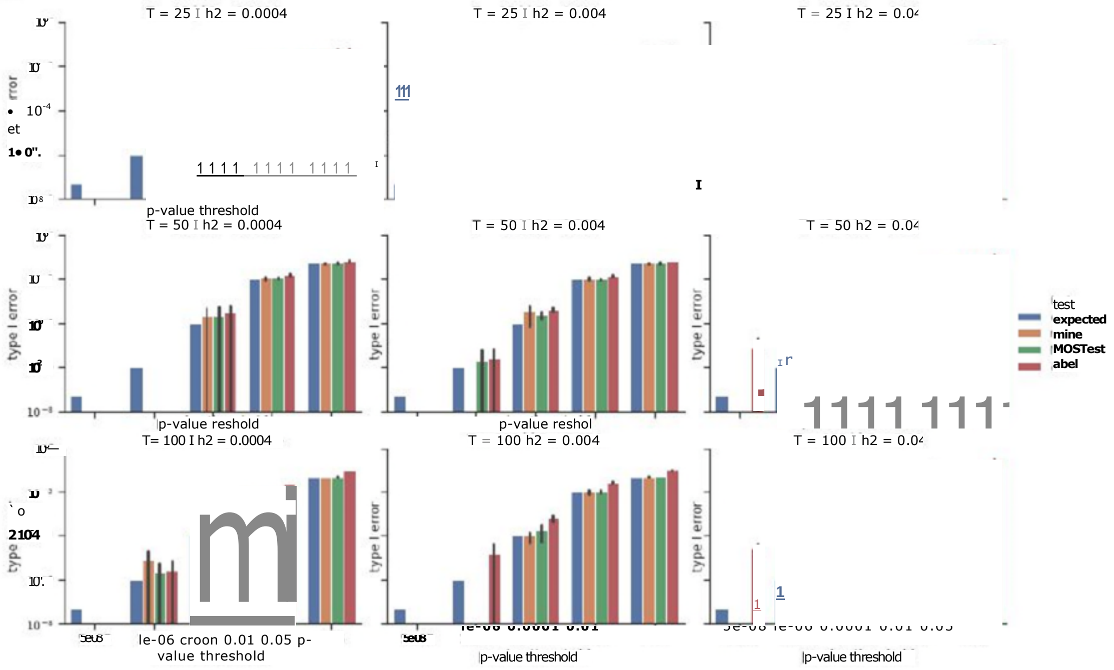
T = 25 I h2 = 0.0004
T = 25 I h2 = 0.004
T = 50 I h2 = 0.0004
T = 50 I h2 = 0.004
T = 100 I h2 = 0.0004
T = 100 h2 = 0.004
type I error
p-value threshold
test
expected
mine
MOSTest
abel

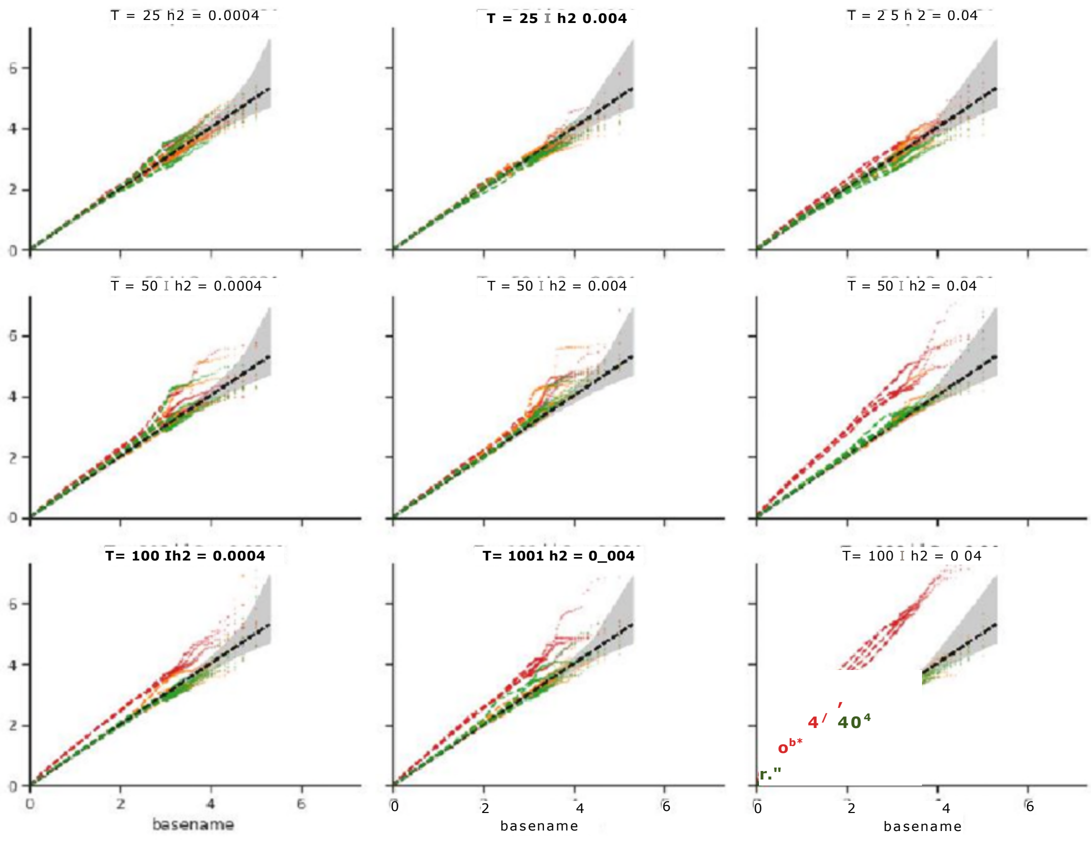
T = 25 h2 = 0.0004
T = 25 I h2 0.004
T = 50 I h2 = 0.0004
T = 50 I h2 = 0.004
T = 50 I h2 = 0.04
T= 100 Ih2 = 0.0004
basename

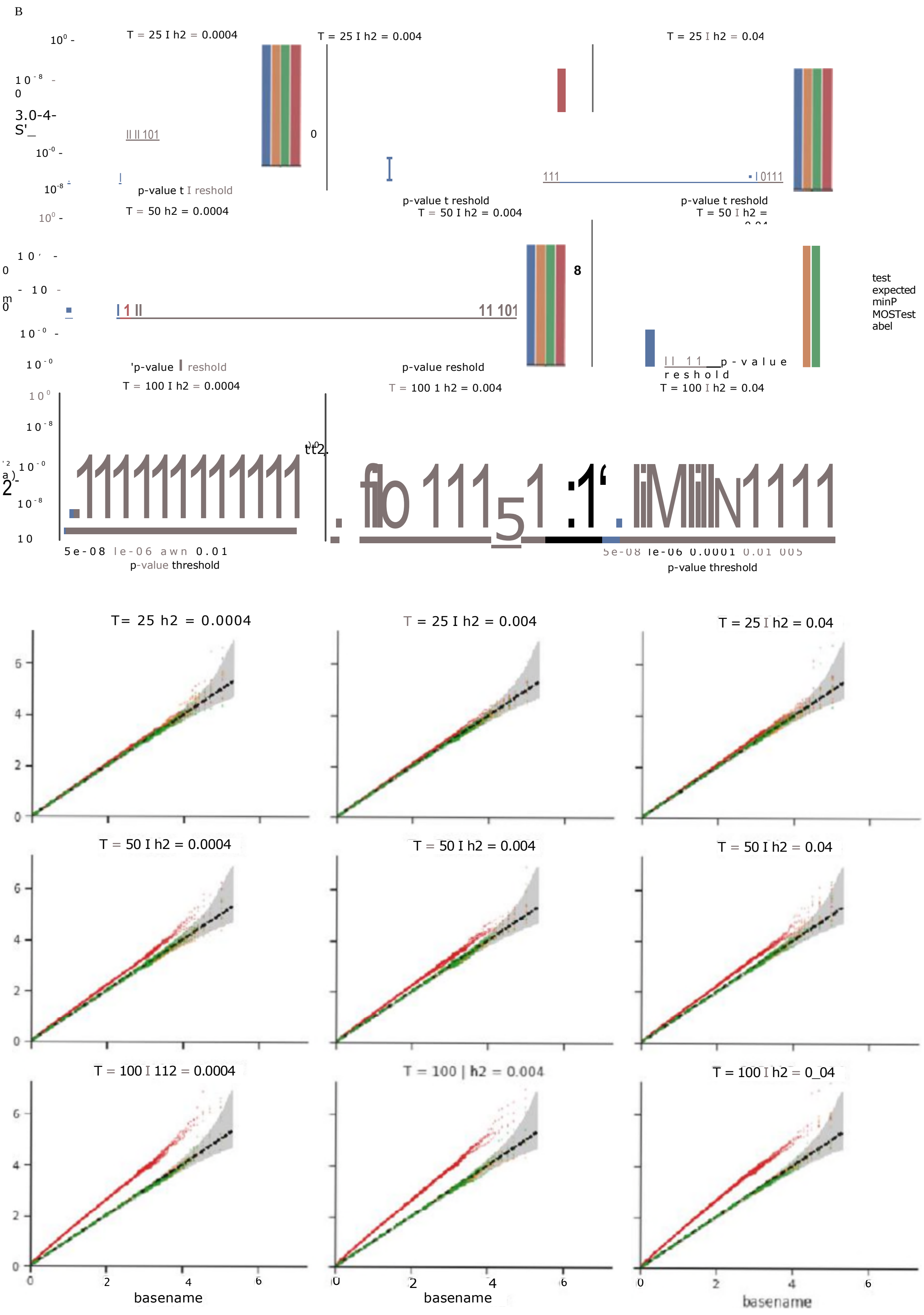
B
T = 25 I h2 = 0.0004
T = 25 I h2 = 0.004
T = 25 I h2 = 0.04
p-value t reshold
T = 50 h2 = 0.0004
T = 50 I h2 = 0.004
test
expected
minP
MOSTest
abel
T = 100 I h2 = 0.0004
T = 100 1 h2 = 0.004
T = 100 I h2 = 0.04
p-value threshold
T= 25 h2 = 0.0004
T = 25 I h2 = 0.004
T = 25 I h2 = 0.04
T = 50 I h2 = 0.0004
T = 50 I h2 = 0.004
T = 50 I h2 = 0.04
T = 100 I 112 = 0.0004
T = 100 | h2 = 0.004
T = 100 I h2 = 0_04
basename
basename
basename

C

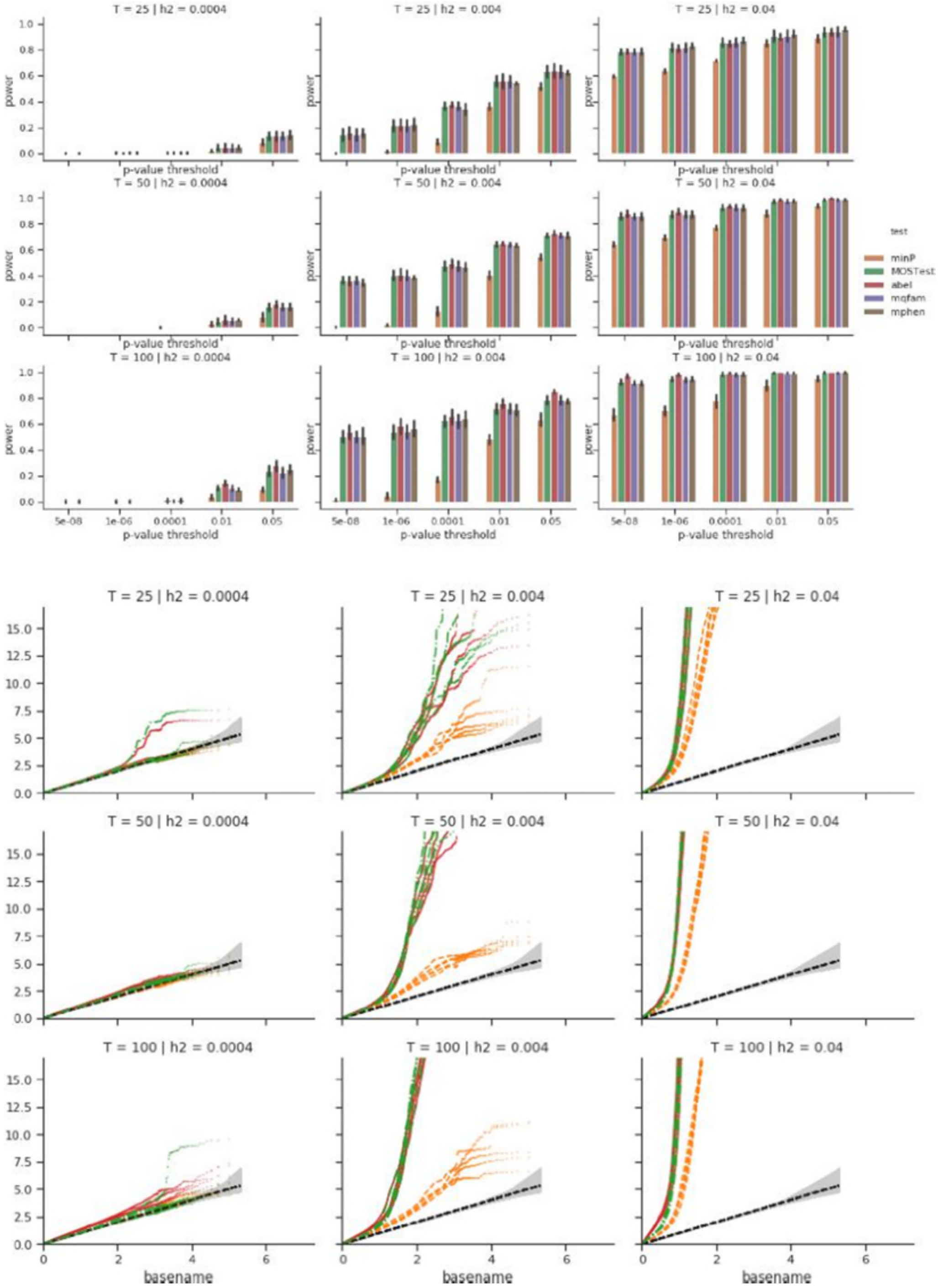

Supplementary Figure 7. Simulation results across two chromosomes (chr21 and chr22), showing inflation in MultiABEL test for large number of total traits in the analysis. Simulation parameters are the same as in Supplementary Figure 2. **A**) Under null (chr 22). B) under permutation. C) Power (chr 21). Under A), we observe a few cases with incorrect type-I error of the MultiABEL test, particularly with large number of traits (T>50). This issue did not manifest itself in whole-genome simulations with all 22 chromosomes, suggesting that MultiABEL require a sufficiently large number of genetic variants to correctly estimate phenotypic correlation structure. Appearance of the data bars, error bars and QQ plots is the same as on the previous figure.

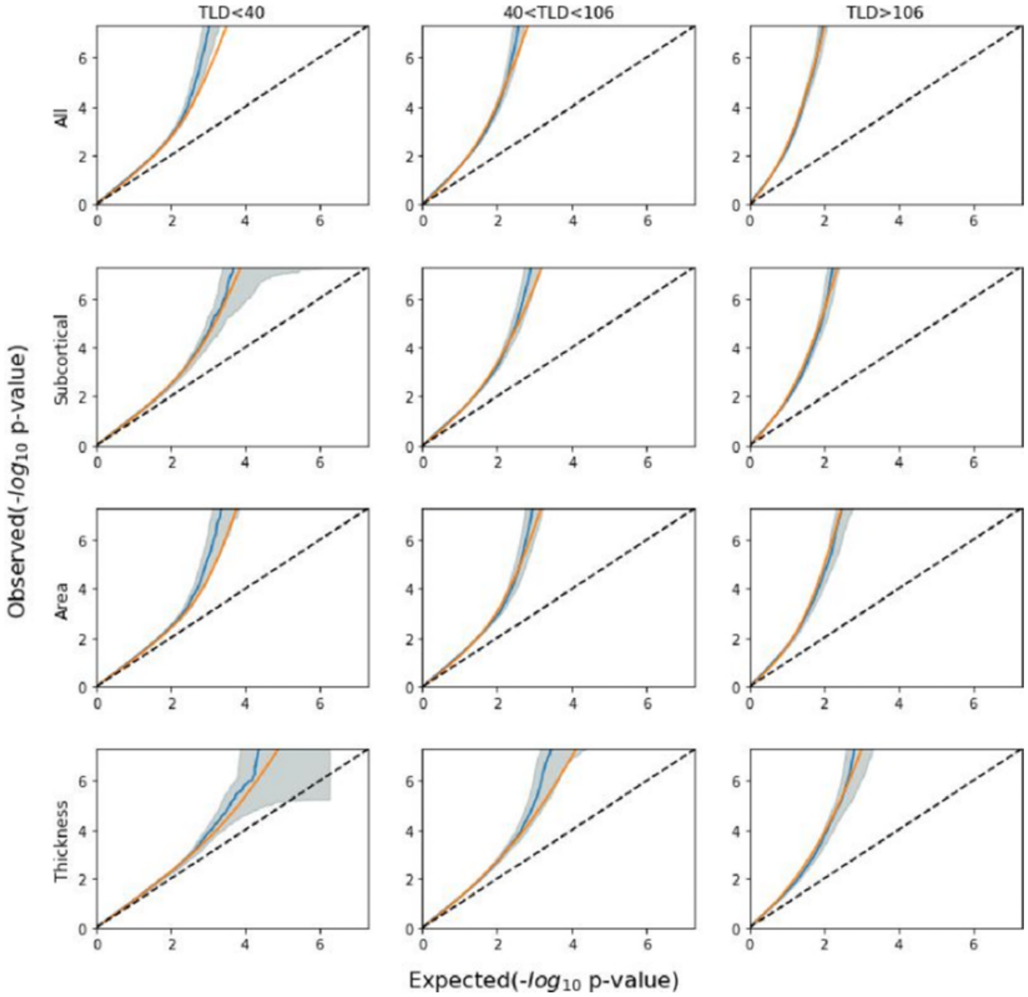

Supplementary Figure 8. QQ plots for subsets of SNPs, showing observed MOSTest p-values (in blue) and MiXeR model prediction (in orange). Rows correspond to feature sets (All, Subcortical, Area and Thickness). For each feature set, SNPs were partitioned into 3 groups according to their total LD score (TLD), constrained to common SNPs with a minor allele frequency above 0.20. The model was fit only once, so that all model predictions are based on the same set of parameters. Observed QQ plots show a stronger GWAS signal for SNPs with higher LD score. Model's prediction follows the same pattern, indicating that model correctly captures the dependency of association statistics on the Total LD score.

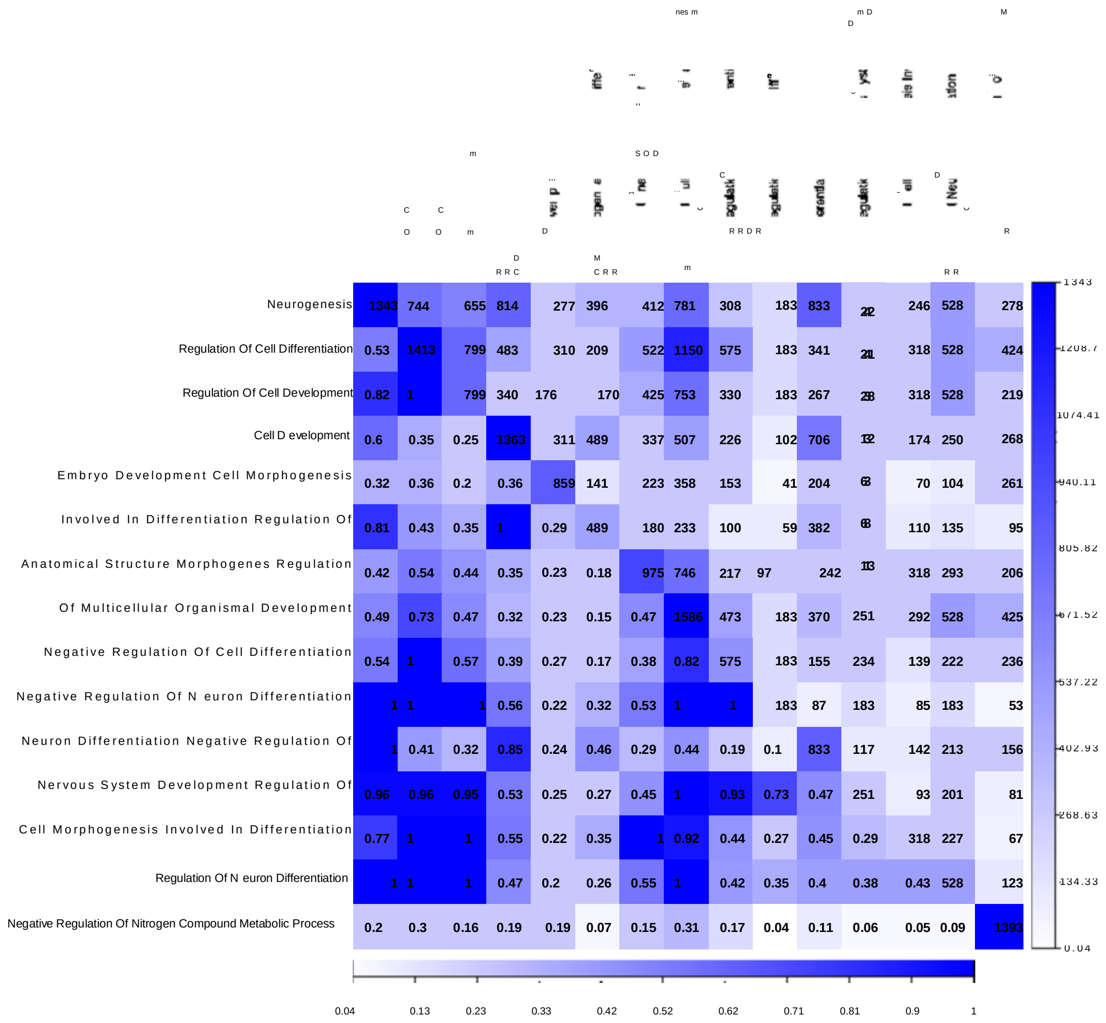

Supplementary Figure 9. Heatmap of the gene overlap between the reported genetic pathways. The diagonal indicates the number of genes in each reported genetic pathway, the upper triangle shows the number of genes shared between the pathways, and the lower triangle indicates the number of genes shared between the pathways divided by the total number of genes in the pathway on each row.

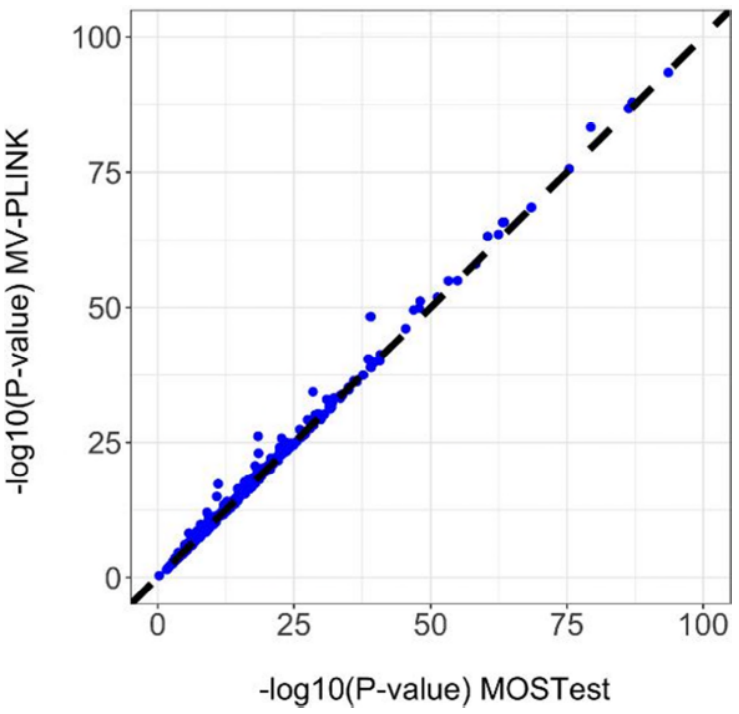

Supplementary Figure 10. Comparison between MQFAM and MOSTest for a selected set of 356 SNPs. This reveals similar statistical power (high correlation, r=0.9976, between MQFAM and MOSTest -log10(p-values), with median of 14.16 for MOSTest versus 14.40 for MQFAM).

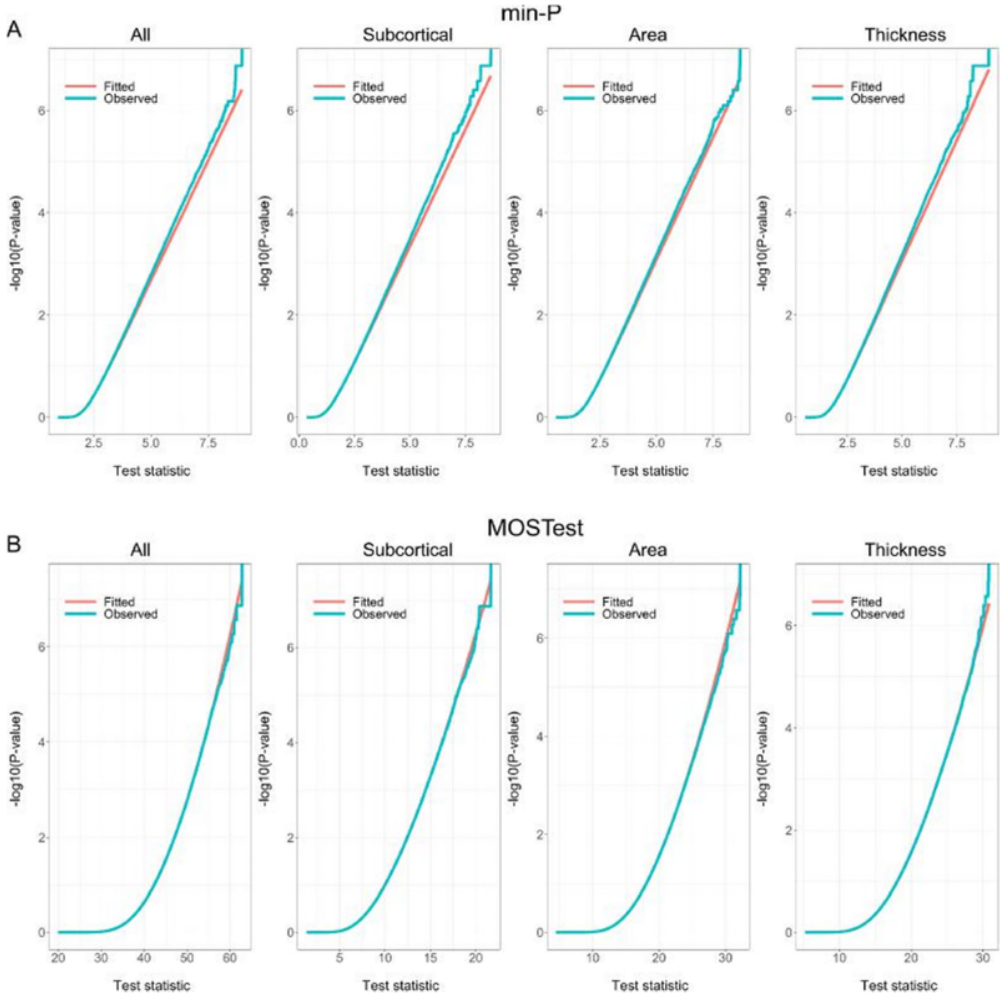

Supplementary Figure 11. P-value distributions under the null. Each subplot shows an empirical distribution of the min-P (top row) and MOSTest (bottom row) test statistics under null (calculated via permutations), along with p-value calculated from the test. Columns correspond to different sets of phenotypes included in the analysis. OX axis show the actual value of the test statistic: -log10(min-P), for the min-P test, and X = zl()1 z for MOSTest. The "Observed" plot shows empirical distribution of the test statistic; "Fitted" plot shows p-values calculated from gamma(a,b) distribution (MOSTest) and Beta(a,b) distribution (min-P) after fitting the two parameters to the observed data. Coincidence of the "Fitted" and "Observed" plots indicate that under null MOSTest p-values are uniformly distributed, as expected for a statistical test with well-calibrated type I error.

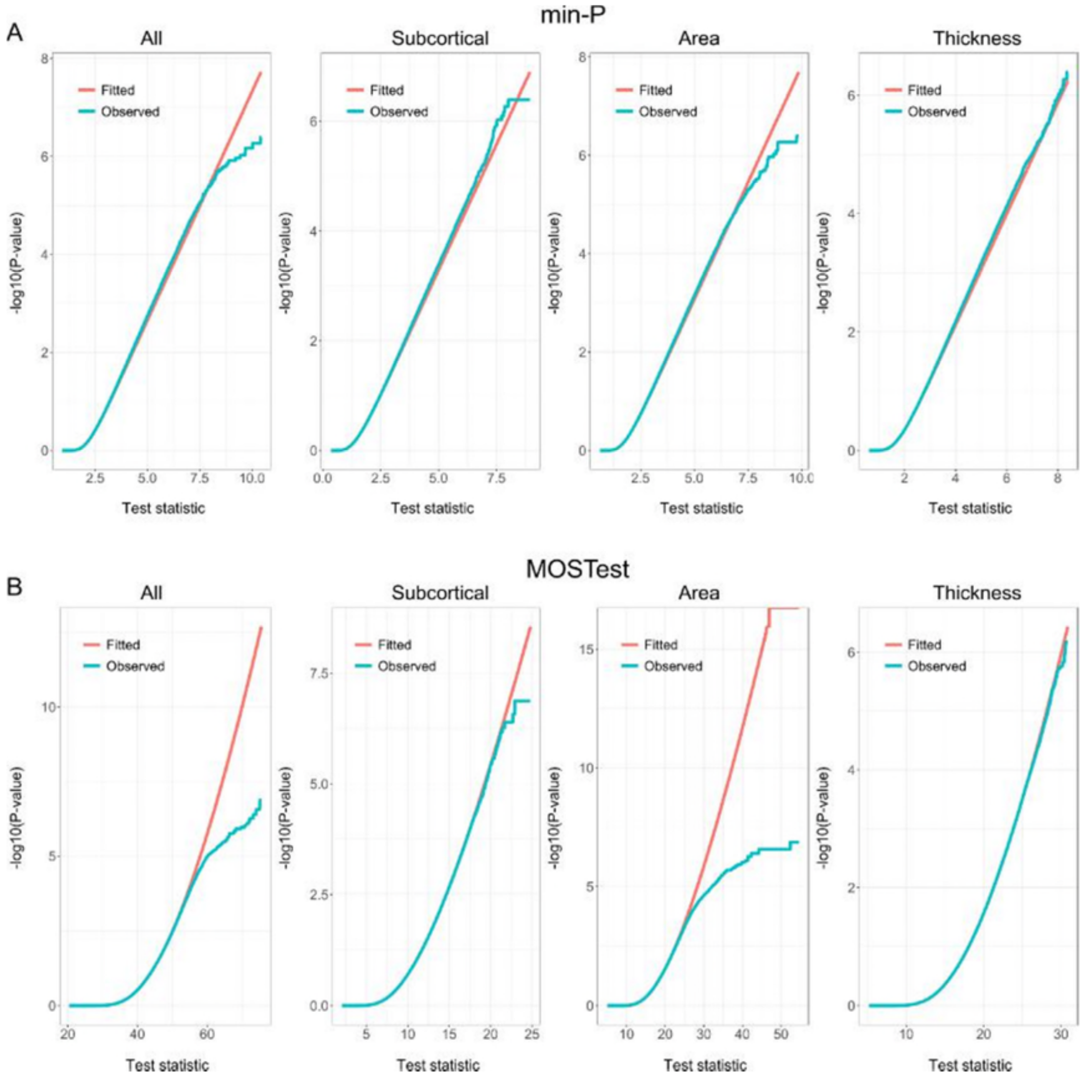

Supplementary Figure 12. Distribution of min-P and MOSTest p-values under permutation, without applying rank-based inverse normal transformation. This shows large deflection in the tails of the distribution. Appearance as in Supplementary Figure 11.

Supplementary Table 1.

Regional brain morphology outcome measures, per subset, included in the study.

Subcortical Volumes	Surface Area & Thickness
1. Lateral Ventricle	1. Bankssts
2. Inferior Lateral Ventricle	2. Caudalanteriorcingulate
3. Cerebellum White Matter	3. Caudalmiddlefrontal
4. Cerebellum Cortex	4. Cuneus
5. Thalamus Proper	5. Entorhinal
6. Caudate	6. Fusiform
7. Putamen	7. Inferiorparietal
8. Pallidum	8. Inferiortemporal
9. 3rd Ventricle*	9. Isthmuscingulate
10. 4th Ventricle*	10. Lateraloccipital
11. Brain Stem*	11. Lateralorbitofrontal
12. Hippocampus	12. Lingual
13. Amygdala	13. Medialorbitofrontal
14. Accumbens Area	14. Middletemporal
15. Ventral Diencephalon	15. Parahippocampal
16. Choroid Plexus	16. Paracentral
17. 5th Ventricle*	17. Parsopercularis
18. CC Posterior*	18. Parsorbitalis
19. CC Mid Posterior*	19. Parstriangularis
20. CC Central*	20. Pericalcarine
21. CC Mid Anterior*	21. Postcentral
22. CC Anterior*	22. Posteriorcingulate
	23. Precentral
	24. Precuneus
	25. Rostralanteriorcingulate
	26. Rostralmiddlefrontal
	27. Superiorfrontal
	28. Superiorparietal
	29. Superiortemporal
	30. Supramarginal
	31. Frontalpole
	32. Temporalpole
	33. Transversetemporal
	34. Insula

*Note: * not bilateral*

Supplementary Table 2.

Number of whole-genome significant SNPs, independent SNPs and independent loci discovered, per test and per feature set.

Test	Feature set	# significant SNPs	# independent SNPs	# loci discovered
MOSTest	All	93461	1425	347
MOSTest	Subcortical	46937	607	177
MOSTest	Surface area	33756	538	139
MOSTest	Cortical thickness	20443	229	71
min-P	All	24203	357	115
min-P	Subcortical	13194	228	90
min-P	Surface area	12650	182	53
min-P	Cortical thickness	9305	81	24

Supplementary Table 3.

The conditioning number of the variance-covariance matrix (R), parameters of the Beta distribution (in min-P test) and gamma distribution (in MOSTest).

	#features	cond(R)	Beta_a	Beta_b	gamma_a	gamma_b
All	171	2237.48	0.936	134.872	85.755	1.994
Area	68	1025.877	0.953	57.583	33.935	2.005
Thickness	68	191.599	0.938	53.641	34.049	1.998
Subcortical	35	132.63	0.913	20.977	17.484	2.002

Supplementary Table 4.

Parameters validated in simulations. Due to exponential number of combinations, only 76 informative combinations were tested (with 10 repetitions for each combination).

	Description	Tested values
T	Total number of traits in the analysis (same as K parameter in the main text)	1,2,4,10,20,25,50,100
t	Number of traits that share genetic effects	1,2,3,4,5,10,25,50,100
nc	Number of causal variants, drawn from chr21	10, 100, 1000
dist	Distribution of genetic effects	norm - standard normal distribution, cauchy - Cauchy distribution, sparse - standard normal distribution for "t" out of "T" features, other traits not heritable
re	Covariance structure of the phenotype residuals, i.e. "eps" in "y=G beta + eps"	eye - identity matrix (uncorrelated phenotypes) real - covariance informed by real phenotypes (first T subcortical volumes from the main text analysis)
rg	Covariance structure of the genetic effects, i.e. "beta" in "y = G beta + eps"	eye, real (as defined above)
h2	Heritability of traits	.0004, .004, .04, 0.4 (as causal effects are always allocated to chr21, heritability h2=.004 results in similar effect sizes as h2=0.291 for SNPs drawn across all chromosomes)
comb	Linear combination of traits	none – keeps original features sum – replaces the set of T features with T(T-1)/2 features, calculated as pairwise sums of all pairs from T
link	Link function, applied to the resulting phenotype	id – identity function (no transformation) exp – exponent (results in highly non-linear phenotypes with and heavy tail distribution)
int	Whether to perform rank-based inverse-normal transformation on all features	T, F
eig	For MOSTest, "eig" indicates the parameter of spectral regularization of the phenotype covariance matrix	0 (no regularization) 275 (best parameter for 25*24/2=300 features, produced from T=25 due to pairwise combination of all features)

Supplementary Table 5.

The list of 76 scenarios validated in simulations.

T	t	nc	dist	rg	re	h2	comb	link	int	SFig	GWAS	MOSTest	abel	mqfam	mphen
2	1	100	sparse	eye	eye	.04	none	id	T	3	237.1	0.6	2	0	4.1
2	2	100	norm	eye	eye	.0004	none	id	T	2	275.7	0.6	2	0	8.4
2	2	100	norm	eye	eye	.004	none	id	T	2	259	0.5	2	0	10.7
2	2	100	norm	eye	eye	.04	none	id	T	2	259	0.5	2	0	10.4
2	2	100	sparse	eye	eye	.04	none	id	T	3	243.3	0.6	2	0	4.1
4	1	100	sparse	eye	eye	.04	none	id	T	3	240.6	0.6	2.7	0.1	4.8
4	2	100	sparse	eye	eye	.04	none	id	T	3	233.9	0.6	2.7	0.1	4.6
4	4	100	sparse	eye	eye	.04	none	id	T	3	269.5	0.6	2.9	0.1	5
10	1	100	sparse	eye	eye	.04	none	id	T	3	245.4	0.7	4.8	0.4	6.9
10	2	100	sparse	eye	eye	.04	none	id	T	3	237.1	0.6	4.9	0.4	6.2
10	4	100	sparse	eye	eye	.04	none	id	T	3	264.6	0.7	5.4	0.4	6.2
10	10	100	norm	eye	eye	.0004	none	id	T	2	272.3	0.7	5.4	0.5	14.3
10	10	100	norm	eye	eye	.004	none	id	T	2	280.6	0.7	4.9	0.4	16.8
10	10	100	norm	eye	eye	.04	none	id	T	2	280.8	0.7	4.9	0.4	14.6
10	10	100	sparse	eye	eye	.04	none	id	T	3	260.6	0.7	4.9	0.4	7
20	1	100	sparse	eye	eye	.04	none	id	T	3	257	0.8	8.7	1.6	10.2
20	2	100	sparse	eye	eye	.04	none	id	T	3	251.3	0.9	8.5	1.6	9.7
20	4	100	sparse	eye	eye	.04	none	id	T	3	257.1	0.9	9.7	1.5	9.8
20	10	100	sparse	eye	eye	.04	none	id	T	3	269.1	0.9	9.1	1.4	13.5
25	1	100	cauchy	eye	eye	.04	none	id	T	4	281.7	0.9	11.5	77.8	162.6
25	1	100	norm	eye	eye	.04	none	id	T	4	297.6	1	11.8	72.4	155
25	1	100	sparse	eye	eye	.04	none	id	T	3,4	281.9	0.9	11.3	2.2	10.7
25	2	100	cauchy	eye	eye	.04	none	id	T	4	264.4	1	11.7	47.1	167.8
25	2	100	norm	eye	eye	.04	none	id	T	4	295.2	1	12.2	38.3	139.3
25	2	100	sparse	eye	eye	.04	none	id	T	3,4	264.8	1	12.1	2.3	12.6
25	3	100	cauchy	eye	eye	.04	none	id	T	4	259.3	1	12.2	26.8	172.5
25	3	100	norm	eye	eye	.04	none	id	T	4	264.4	1	12.2	26.7	144.7
25	3	100	sparse	eye	eye	.04	none	id	T	3,4	259.4	1	11.9	2.2	12.7
25	4	100	cauchy	eye	eye	.04	none	id	T	4	299.1	0.9	12.2	21.7	114.6
25	4	100	norm	eye	eye	.04	none	id	T	4	274.5	1	11.8	18.6	118.9
25	4	100	sparse	eye	eye	.04	none	id	T	3,4	317.8	0.9	12.5	2.9	21.8
25	5	100	cauchy	eye	eye	.04	none	id	T	4	252.3	0.9	12.1	18.2	65.9
25	5	100	norm	eye	eye	.04	none	id	T	4	289.3	0.9	12.3	21.1	95.5
25	5	100	sparse	eye	eye	.04	none	id	T	3,4	252.4	0.9	11.8	2.9	12.5
25	25	10	norm	eye	eye	.0004	none	id	T	1	248.6	1	12.5	0.2	1.3
25	25	10	norm	eye	eye	.004	none	id	T	1	243.5	1.1	12.6	0.2	1.2
25	25	10	norm	eye	eye	.04	none	id	T	1	248.9	1.1	11.8	0.3	1.4
25	25	100	norm	eye	eye	.0004	none	id	T	1,2	276.1	0.9	11.5	2.9	26
25	25	100	norm	eye	eye	.004	none	exp	F	5	253.5	1	12.8	2.6	26.8
25	25	100	norm	eye	eye	.004	none	exp	T	5	253.4	0.9	12.8	3.7	25.8
25	25	100	norm	eye	eye	.004	none	id	F	5	267.4	1	12.8	3.4	19.1
25	25	100	norm	eye	eye	.004	none	id	T	1,2,5	286.1	0.9	9.9	2.3	24.8
25	25	100	norm	eye	eye	.004	sum	exp	F	6	319.3	6.9	18.6		
[illegible]	[illegible]	[illegible]	[illegible]	[illegible]	[illegible]	.004	sum	exp	T	6	369.9	7.1	16.8		
						.004	sum	id	F	6	305	7	0		

25	25	100	norm	eye	eye	.004	sum	id	T	6	315.6	6	18.7		
25	25	100	norm	eye	eye	.04	none	id	T	1,2	276.1	0.9	11.8	2.6	25.5
25	25	100	norm	eye	real	.004	none	exp	F	5	266.8	1	13.4	3.1	27.3
25	25	100	norm	eye	real	.004	none	exp	T	5	257.1	0.9	11.3	3.1	23.9
25	25	100	norm	eye	real	.004	none	id	F	5	267	1	13.3	3	29.5
25	25	100	norm	eye	real	.004	none	id	T	5	266.7	1	13	3.1	25
25	25	100	norm	real	eye	.004	none	exp	F	5	264.6	1	13	2.8	26.8
25	25	100	norm	real	eye	.004	none	exp	T	5	259.8	0.9	13.1	3.7	31.4
25	25	100	norm	real	eye	.004	none	id	F	5	254.8	1	12.1	3.7	23.4
25	25	100	norm	real	eye	.004	none	id	T	5	254.7	0.9	11.6	3.6	23.8
25	25	100	norm	real	real	.004	none	exp	F	5	272.4	1	12.4	2.5	22.9
25	25	100	norm	real	real	.004	none	exp	T	5	272.5	0.9	12.4	4.7	26.1
25	25	100	norm	real	real	.004	none	id	F	5	259.7	0.9	12.6	3	28
25	25	100	norm	real	real	.004	none	id	T	5	267.6	1	12.5	3.3	31.6
25	25	1000	norm	eye	eye	.0004	none	id	T	1	248	1	12.5	32.5	98.6
25	25	1000	norm	eye	eye	.004	none	id	T	1	256.1	1	12.7	30.7	180.5
25	25	1000	norm	eye	eye	.04	none	id	T	1	247.6	1	12.8	28.4	120.6
50	1	100	sparse	eye	eye	.04	none	id	T	3	259.7	1.3	25.1	17.6	19.2
50	2	100	sparse	eye	eye	.04	none	id	T	3	257.4	1.3	23.2	11.7	16.6
50	4	100	sparse	eye	eye	.04	none	id	T	3	244	1.3	22.5	12.5	17
50	10	100	sparse	eye	eye	.04	none	id	T	3	271.6	1.4	25.9	18.5	23.2
50	50	100	norm	eye	eye	.0004	none	id	T	2	289.6	1.3	21.7	19.8	32.9
50	50	100	norm	eye	eye	.004	none	id	T	2	289.6	1.3	20.6	14.5	27.5
50	50	100	norm	eye	eye	.04	none	id	T	2	284.5	1.3	20.3	15.5	17.6
100	1	100	sparse	eye	eye	.04	none	id	T	3	251.1	2	55.7	50.6	37.1
100	2	100	sparse	eye	eye	.04	none	id	T	3	273.6	2.1	67.2	53.3	44.5
100	4	100	sparse	eye	eye	.04	none	id	T	3	253.4	2.1	60.1	60.2	37.8
100	10	100	sparse	eye	eye	.04	none	id	T	3	287.2	2.3	61.4	60.9	63.6
100	100	100	norm	eye	eye	.0004	none	id	T	2	323.6	2.3	46.8	72.3	90.4
100	100	100	norm	eye	eye	.004	none	id	T	2	323.7	2.3	50.2	69.7	87.8
100	100	100	norm	eye	eye	.04	none	id	T	2	293.4	2.2	46.4	60.6	90.2

*Legend: The ´GWAS´, ´MOSTest´, ´abel´, ´mqfam´ and ´mphen´ columns give median runtime in minutes across 10 repetitions of the experiment. The ´GWAS´ column indicates the runtime of univariate GWAS across all traits and SNPs, as implemented in MOSTest software, including with and without genotype permutation. ´MOSTest´ indicates the combined runtime of the MOSTest and min-P tests, including time spent in loading summary statistics from binary matlab files. ´abel´ indicates runtime of MultiABEL software, including time needed to load summary statistics from a text file. ´mqfam´ and ´mphen´ indicate time needed for the MQ-FAM and MultiPhen tools to compute p-values in the power analyses, i.e. for causal variants only (the number of causal variants is, approximately, "nc*T/t" variants when dist=norm or dist=cauchy; and "nc" variants when dist=sparse).*

Supplementary Table 6.
Genomic inflation analysis with LD Score Regression intercept (from partitioned LDSC 1kG phase3 reference), confirming no confounding effects (stratification, cryptic relatedness) in MOSTest p-values.

Feature set	Test	LambdaGC	Intercept from LDSC
All	min-P	1.6524	0.9864 (0.0096)
All	MOSTest	2.0217	0.9247 (0.015)
Subcortical	min-P	1.5511	1.0062 (0.0086)
Subcortical	MOSTest	1.7179	0.9742 (0.0106)
Area	min-P	1.453	0.9913 (0.0093)
Area	MOSTest	1.5733	0.9748 (0.0112)
Thickness	min-P	1.4034	1.0085 (0.0068)
Thickness	MOSTest	1.5254	0.9952 (0.0085)
