# Supplementary material for: Understanding the genetic determinants of the brain with MOSTest
Source: Nat Commun. 2020 Jul 14;11:3512. doi: 10.1038/s41467-020-17368-1 (PMC7360598; doi:10.1038/s41467-020-17368-1)
Supplement: Supplementary file 3 — Description of Additional Supplementary Files [file 41467_2020_17368_MOESM3_ESM.pdf]

Descriptions of Additional Supplementary Files

Supplementary Data 1-3

Description: Tables listing information on the lead SNP for each discovered genome-wide significant locus for the analyses of all features combined. The cells listing the p-values for both MOSTest and min-P in the discovery sample are color-coded with a scale running from yellow (least significant) to red (most significant). The cells listing the p-values in the replication sample are color-coded such that green indicates the SNP had a nominally significant effect, while grey indicates the SNP had a non-significant effect. The "# regions" column indicates the number of univariate measures the SNP had a genome-wide significant effect on, in the discovery sample. The "Regions" column lists which regions were significant whereby "A" codes for area, "T" for thickness, "V" for subcortical volume, "L" for left hemisphere, "R" for right hemisphere; the region numbering is listed in Supplementary Table 1. Further abbreviations: Chr=chromosome; BP=base pair; SNP=single nucleotide polymorphism; repl.=replication. Supplementary Data 1 lists the loci that were genome-wide significant through both the MOSTest and the minP approach, Supplementary Data 2 lists the loci that were genome-wide significant only through MOSTest, Supplementary Data 3 lists the loci that were genome-wide significant only through min-P.

Supplementary Data 4-11

Description: Tables listing information on the lead SNP for every discovered genome-wide significant locus for each of the feature subsets, through either the MOSTest or min-P approach. Abbreviations: SNP=single nucleotide polymorphism; A1=allele 1; MAF=minor allele frequency; Chr=chromosome; BP=base pair.

Supplementary Data 12

Description: Table listing each genome-wide significant gene identified by MOSTest on all features combined, using MAGMA. The "Pathways" column indicates in which genetic pathways this gene is included, with the number indicating the row number of the pathways as listed in Supplementary Data 13. Abbreviations: Chr=chromosome; BP=base pair; SNP=single nucleotide polymorphism.

Supplementary Data 13

Description: Table listing each Bonferroni-corrected significant genetic pathway identified by MOSTest on all features combined, using MAGMA. Abbreviation: Std.= Standard.
