# Supplementary material for: Understanding the genetic determinants of the brain with MOSTest
Source: Nat Commun. 2020 Jul 14;11:3512. doi: 10.1038/s41467-020-17368-1 (PMC7360598; doi:10.1038/s41467-020-17368-1)
Supplement: Supplementary file 4 — Supplementary Data 1 [file 41467_2020_17368_MOESM4_ESM.html]

datatables
