# Supplementary figures and images for: Understanding the genetic determinants of the brain with MOSTest (part 2 of 2)
Source: Nat Commun. 2020 Jul 14;11:3512. doi: 10.1038/s41467-020-17368-1 (PMC7360598; doi:10.1038/s41467-020-17368-1)

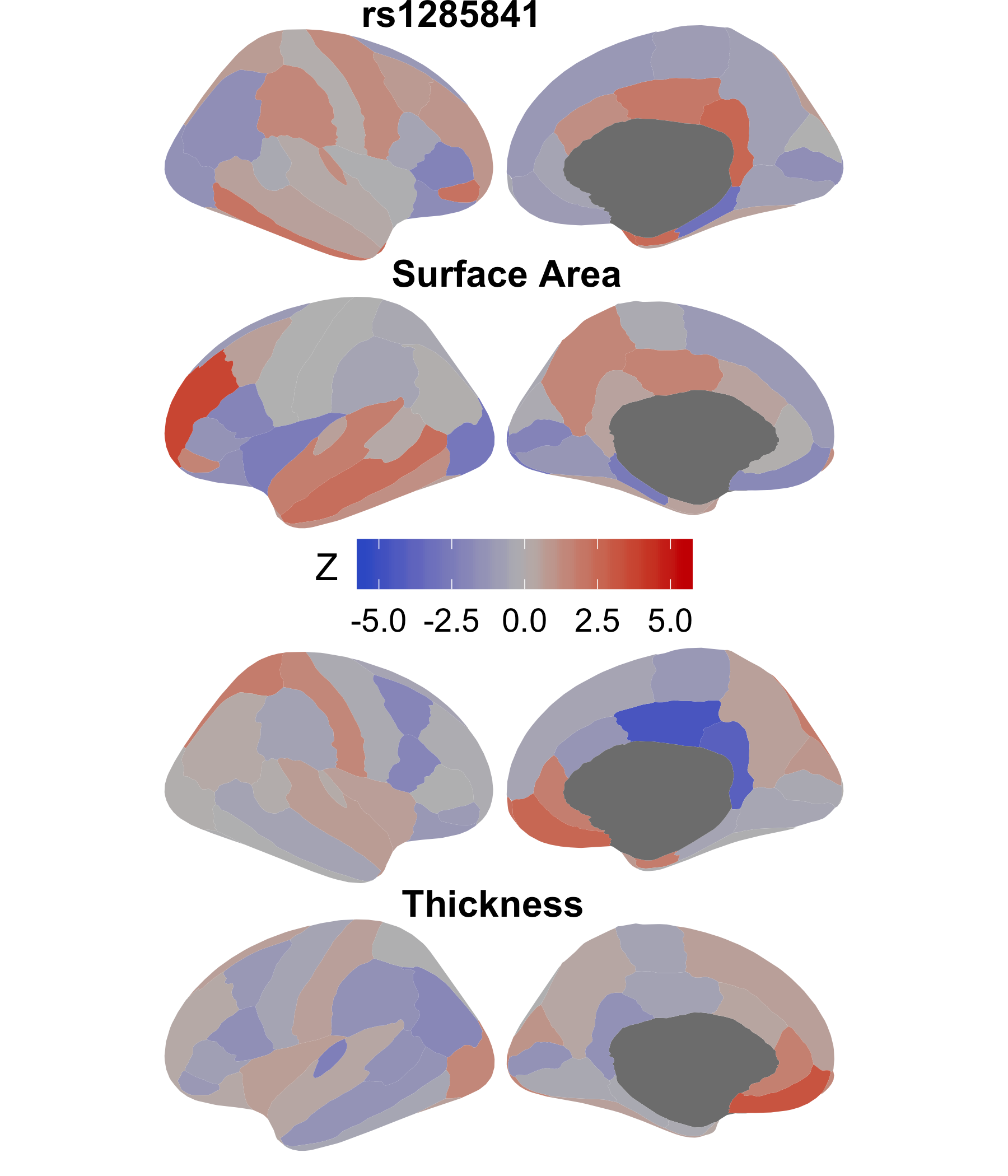

Supplement: Supplementary file 17 — Supplementary Data 14 [file 41467_2020_17368_MOESM17_ESM.gz › BrainMaps/most_aseg_vol/BrainMap028_rs1285841.png]

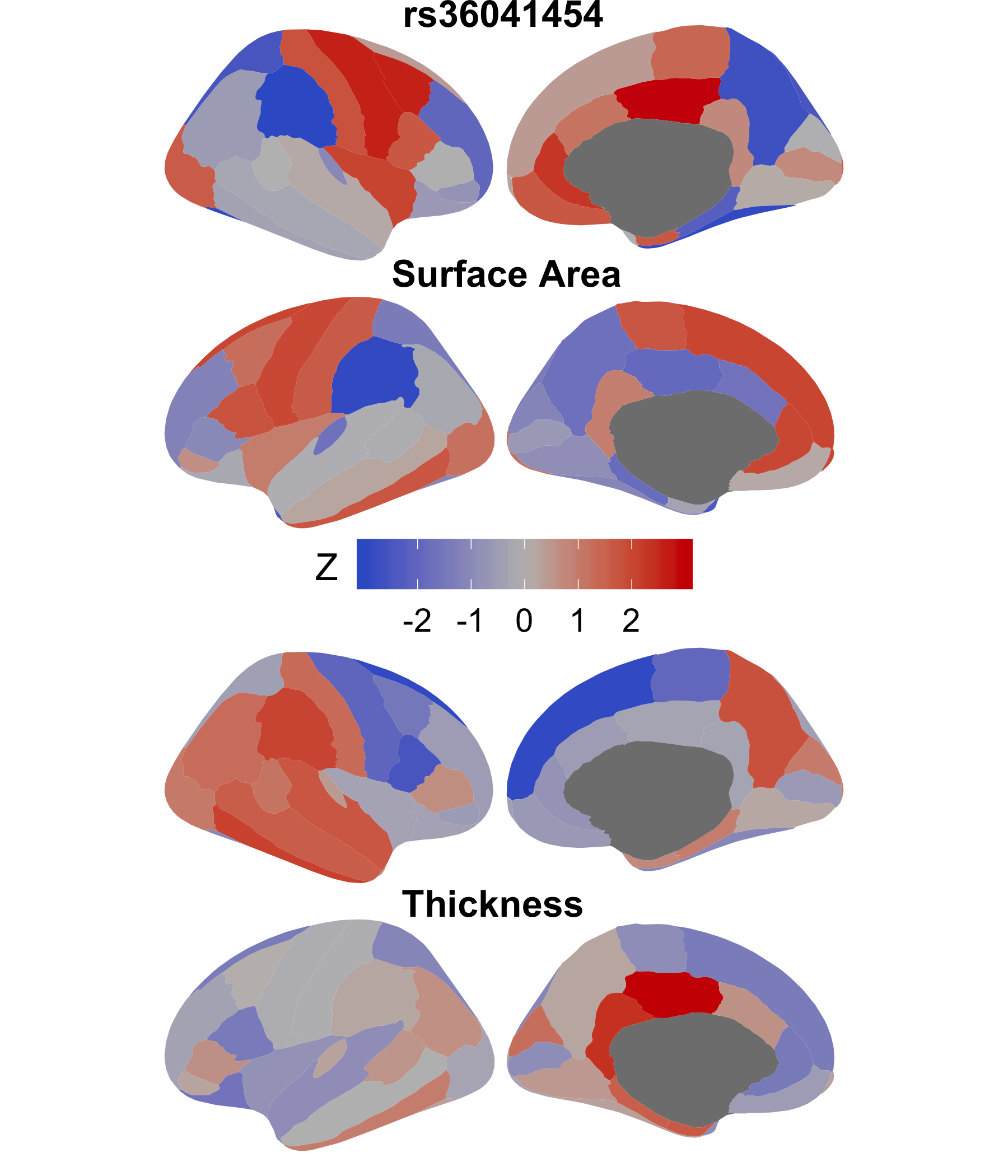

Supplement: Supplementary file 17 — Supplementary Data 14 [file 41467_2020_17368_MOESM17_ESM.gz › BrainMaps/most_aseg_vol/BrainMap171_rs36041454.png]

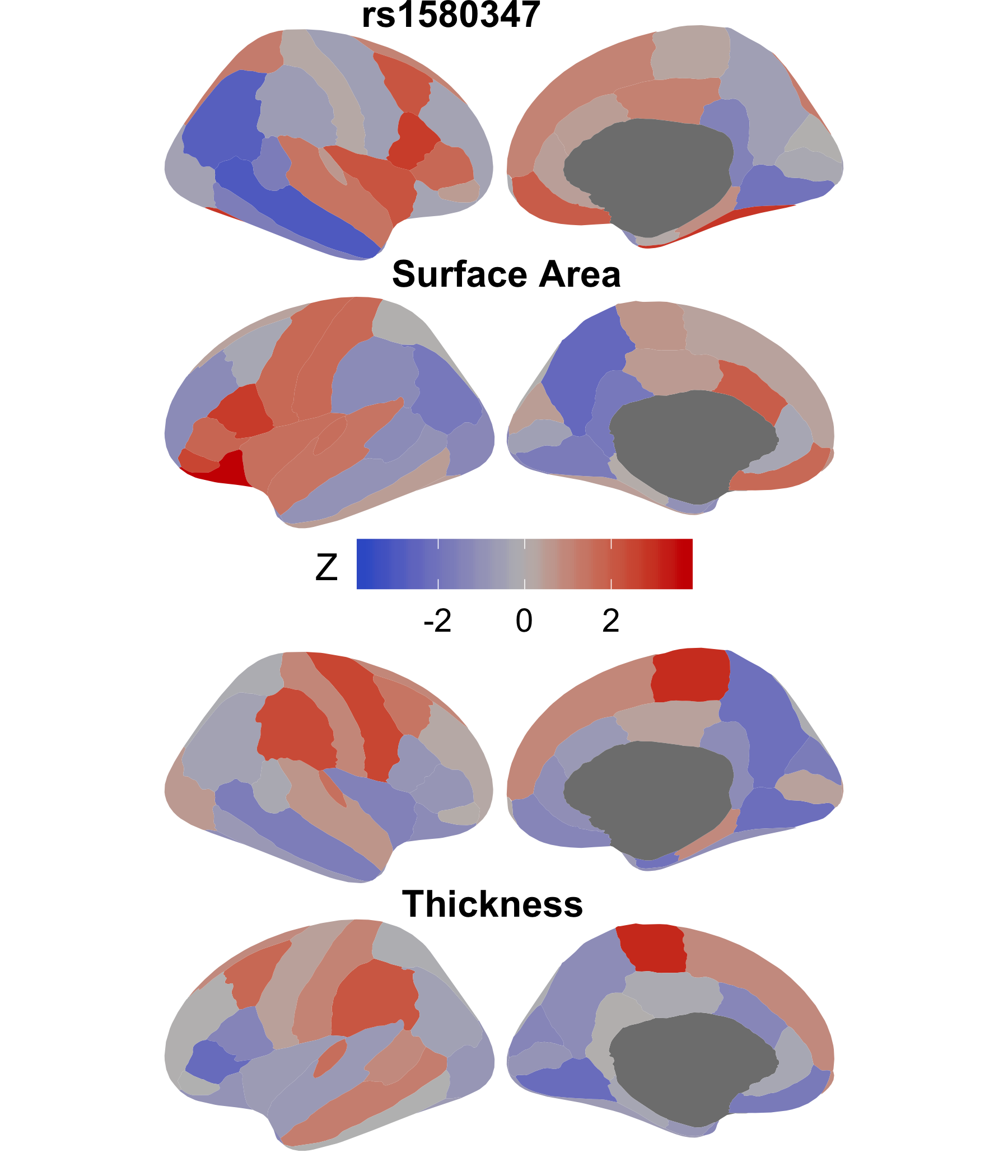

Supplement: Supplementary file 17 — Supplementary Data 14 [file 41467_2020_17368_MOESM17_ESM.gz › BrainMaps/most_aseg_vol/BrainMap155_rs1580347.png]

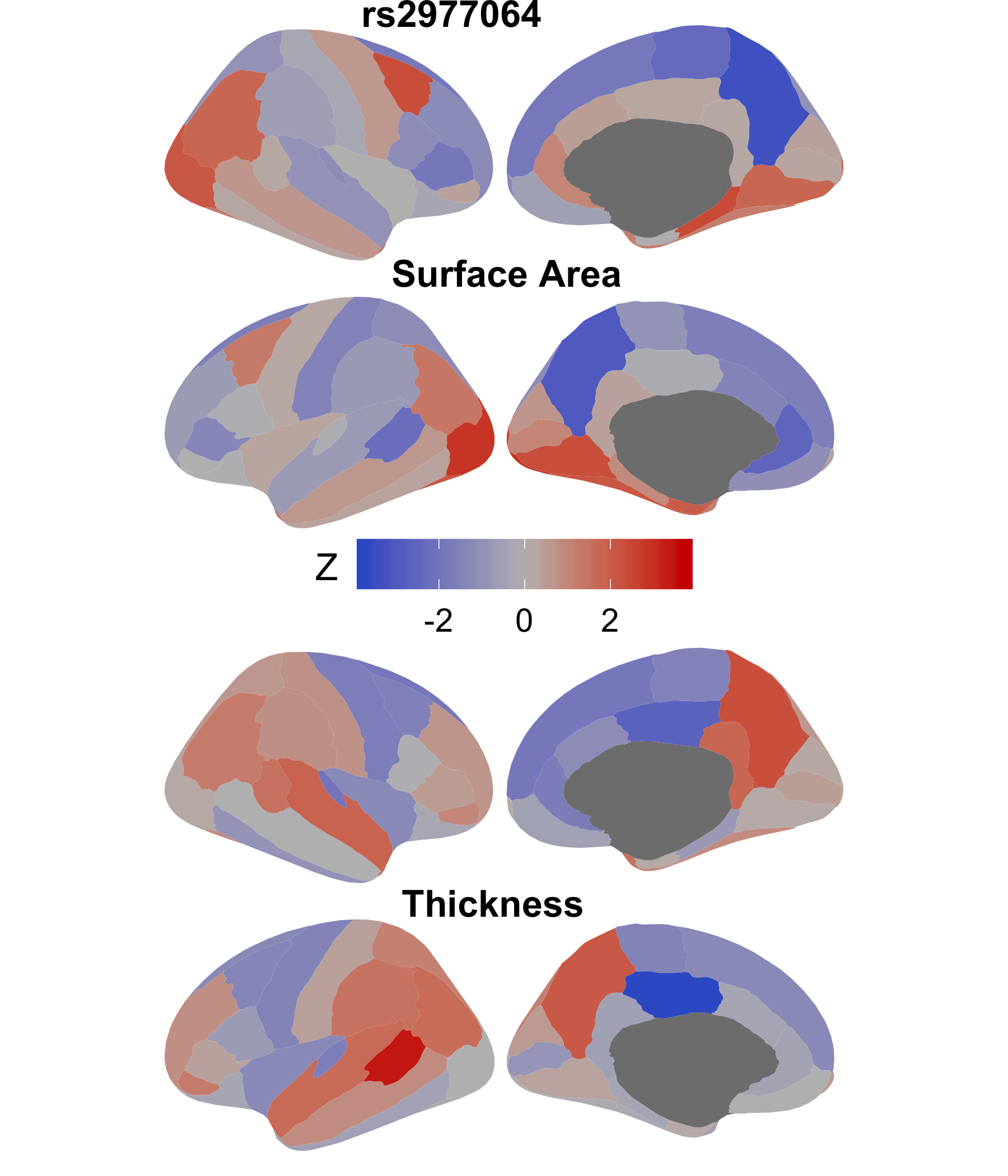

Supplement: Supplementary file 17 — Supplementary Data 14 [file 41467_2020_17368_MOESM17_ESM.gz › BrainMaps/most_aseg_vol/BrainMap111_rs2977064.png]

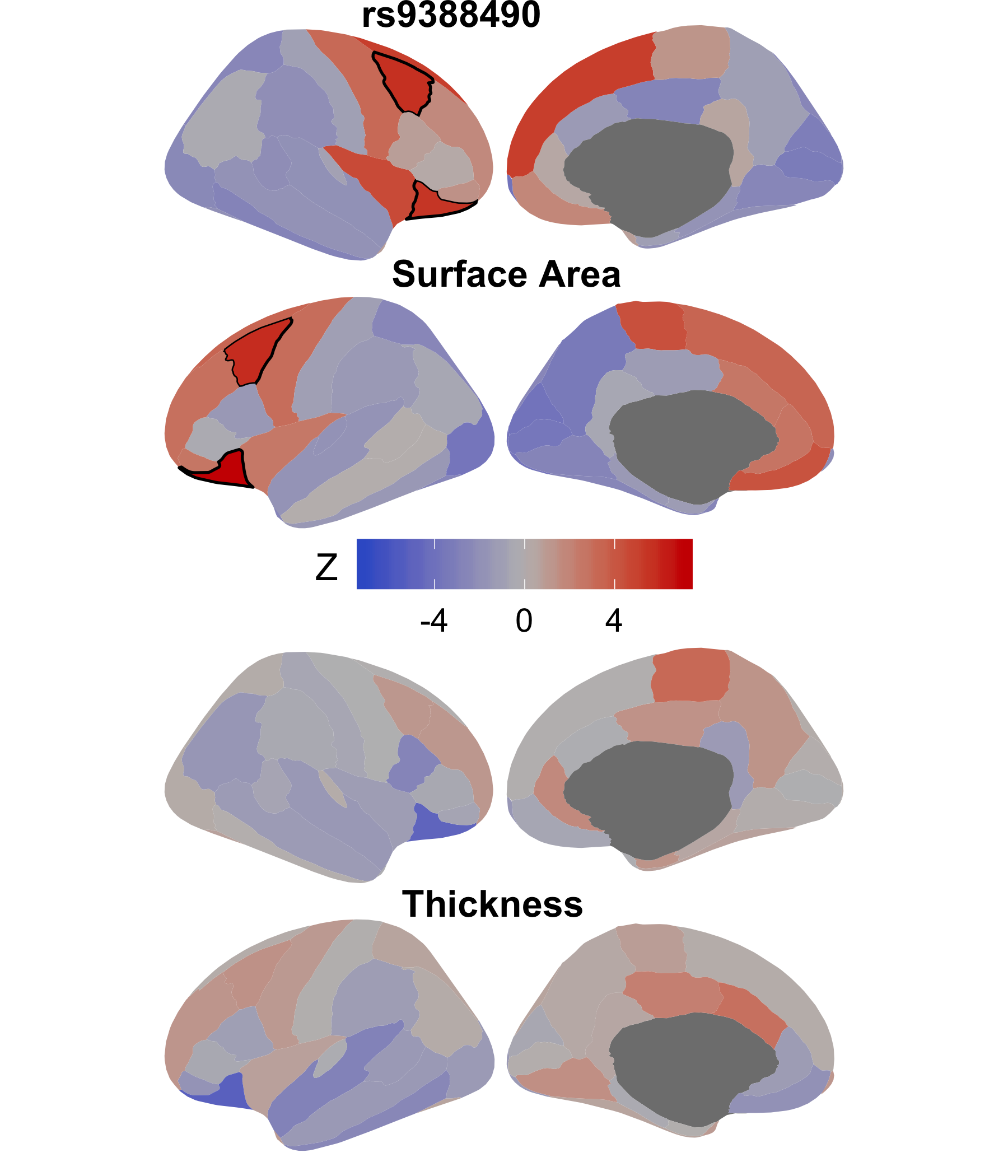

Supplement: Supplementary file 17 — Supplementary Data 14 [file 41467_2020_17368_MOESM17_ESM.gz › BrainMaps/most_aseg_vol/BrainMap054_rs9388490.png]

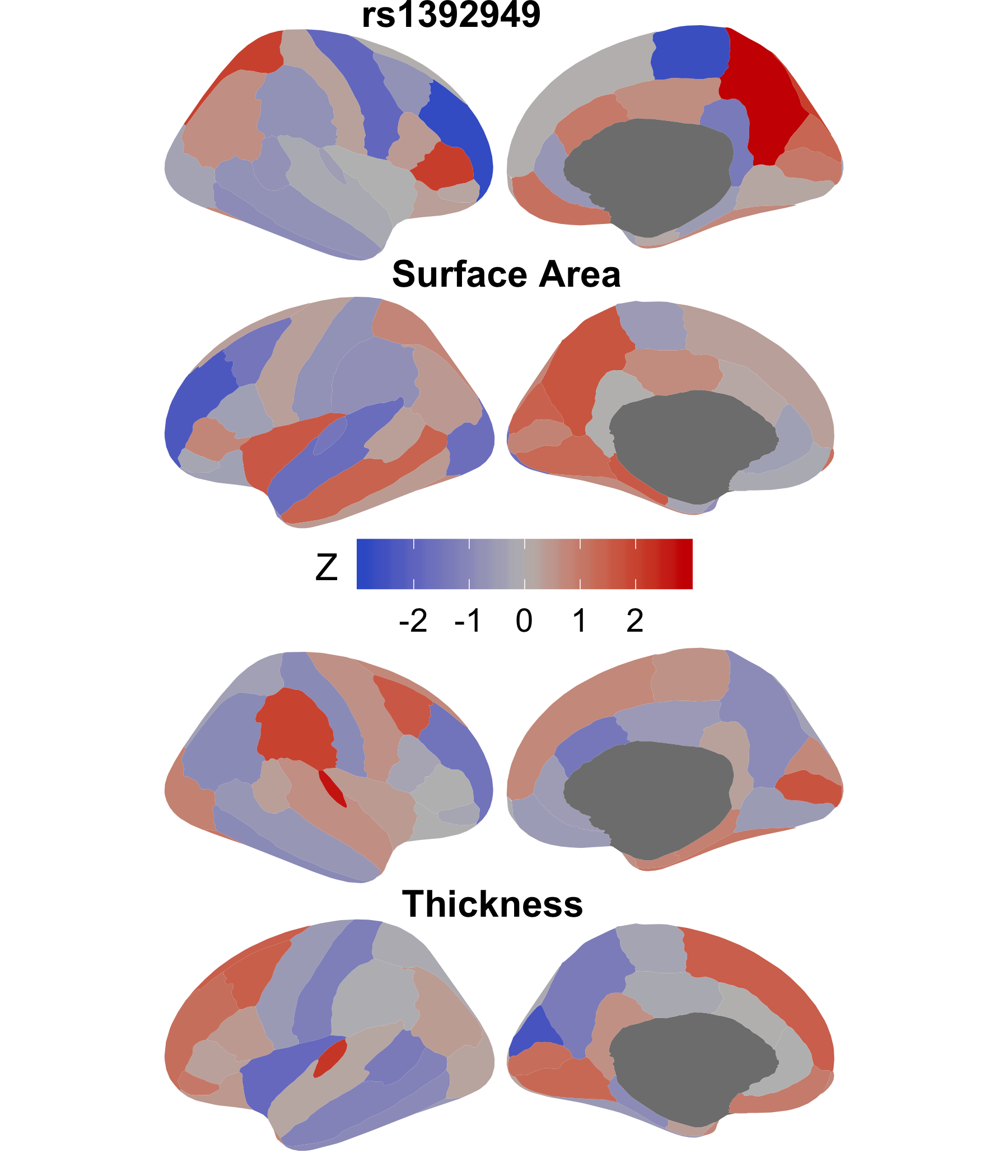

Supplement: Supplementary file 17 — Supplementary Data 14 [file 41467_2020_17368_MOESM17_ESM.gz › BrainMaps/most_aseg_vol/BrainMap106_rs1392949.png]

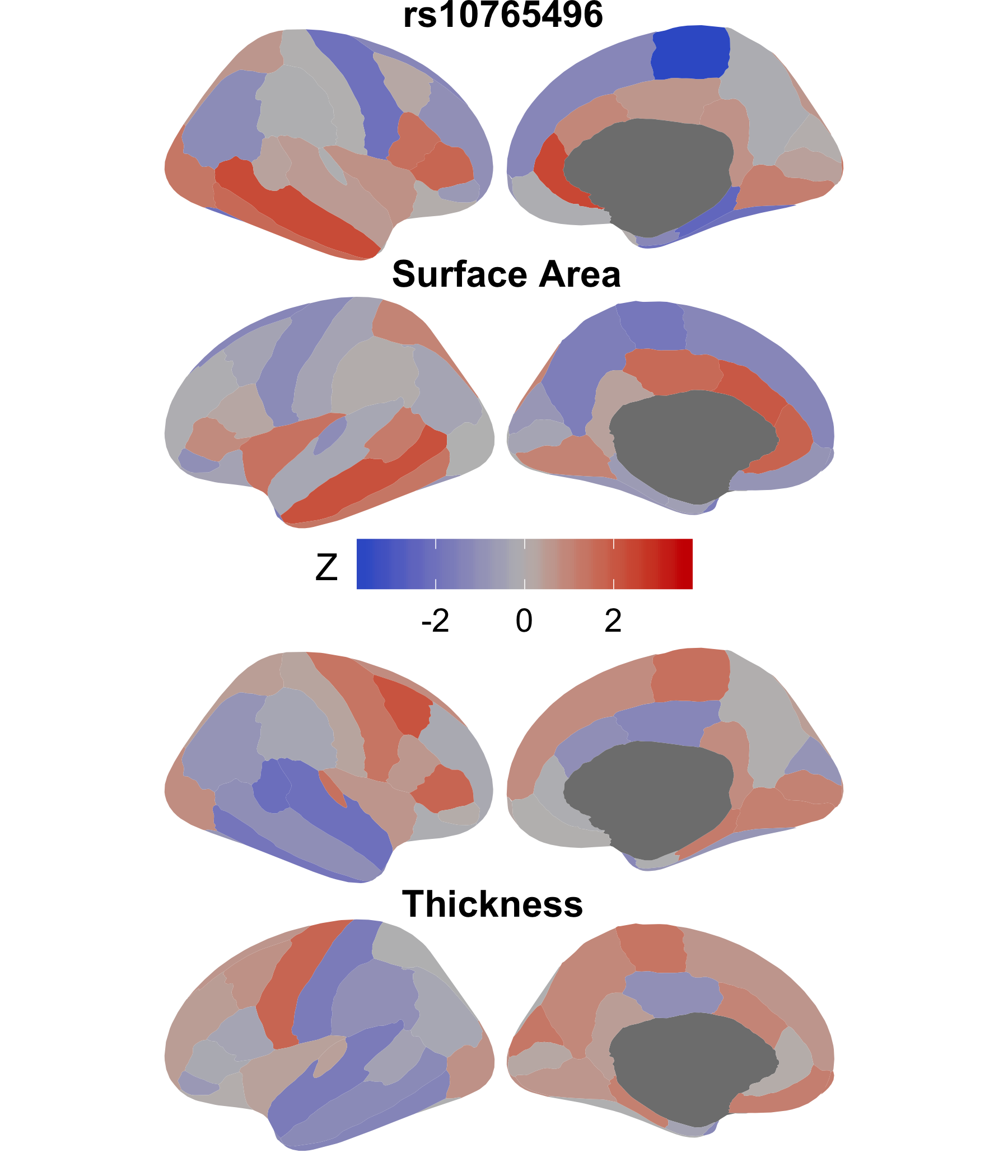

Supplement: Supplementary file 17 — Supplementary Data 14 [file 41467_2020_17368_MOESM17_ESM.gz › BrainMaps/most_aseg_vol/BrainMap164_rs10765496.png]

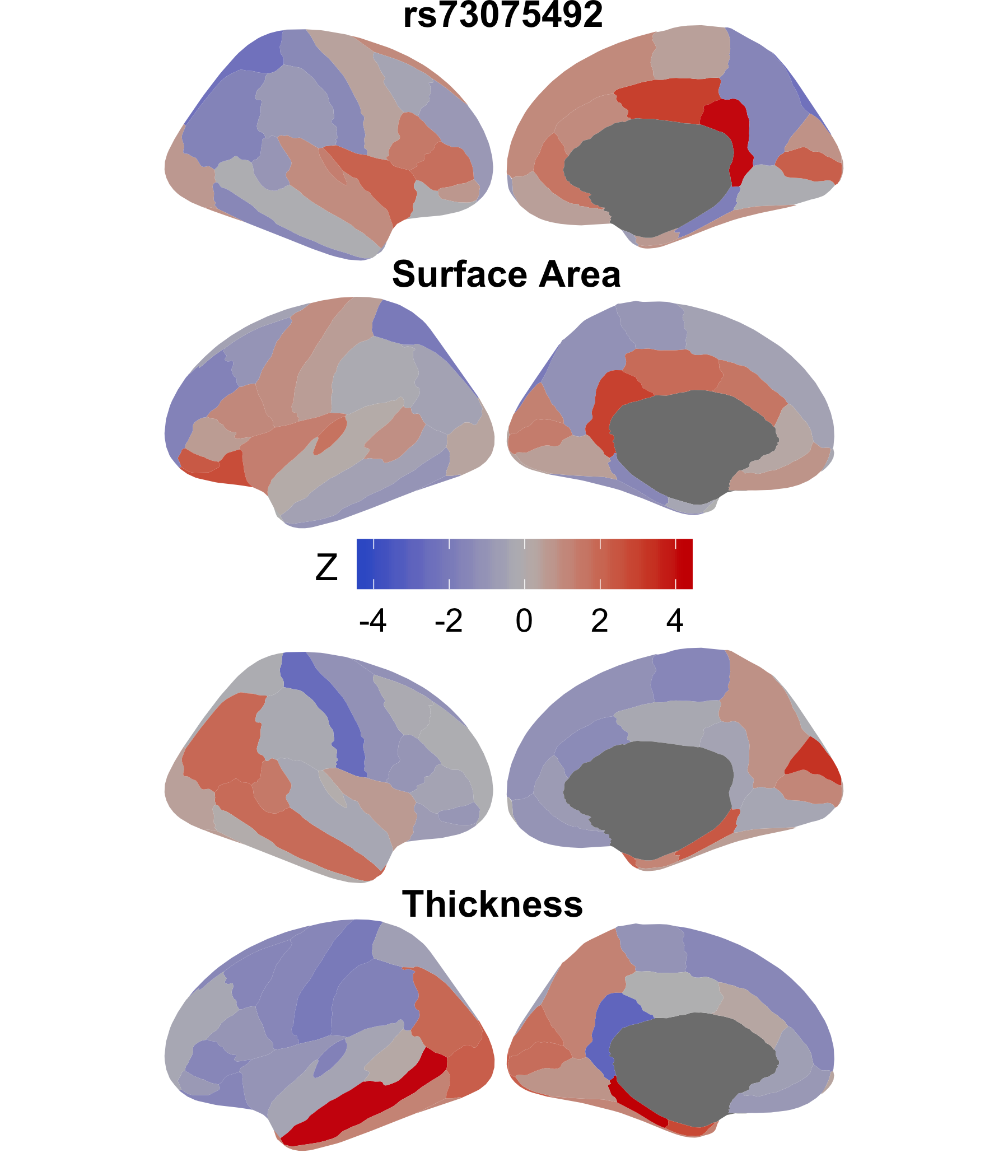

Supplement: Supplementary file 17 — Supplementary Data 14 [file 41467_2020_17368_MOESM17_ESM.gz › BrainMaps/most_aseg_vol/BrainMap161_rs73075492.png]

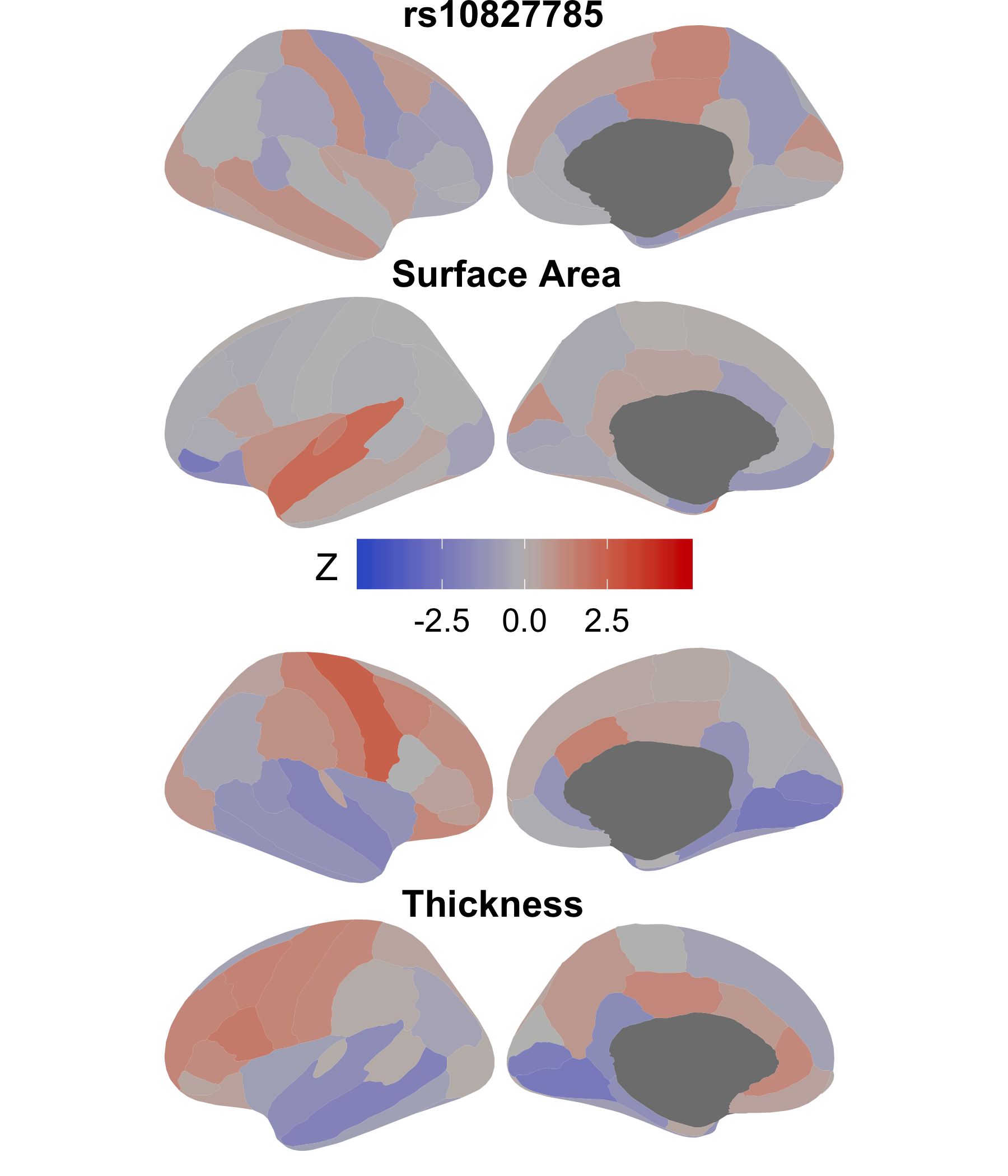

Supplement: Supplementary file 17 — Supplementary Data 14 [file 41467_2020_17368_MOESM17_ESM.gz › BrainMaps/most_aseg_vol/BrainMap079_rs10827785.png]

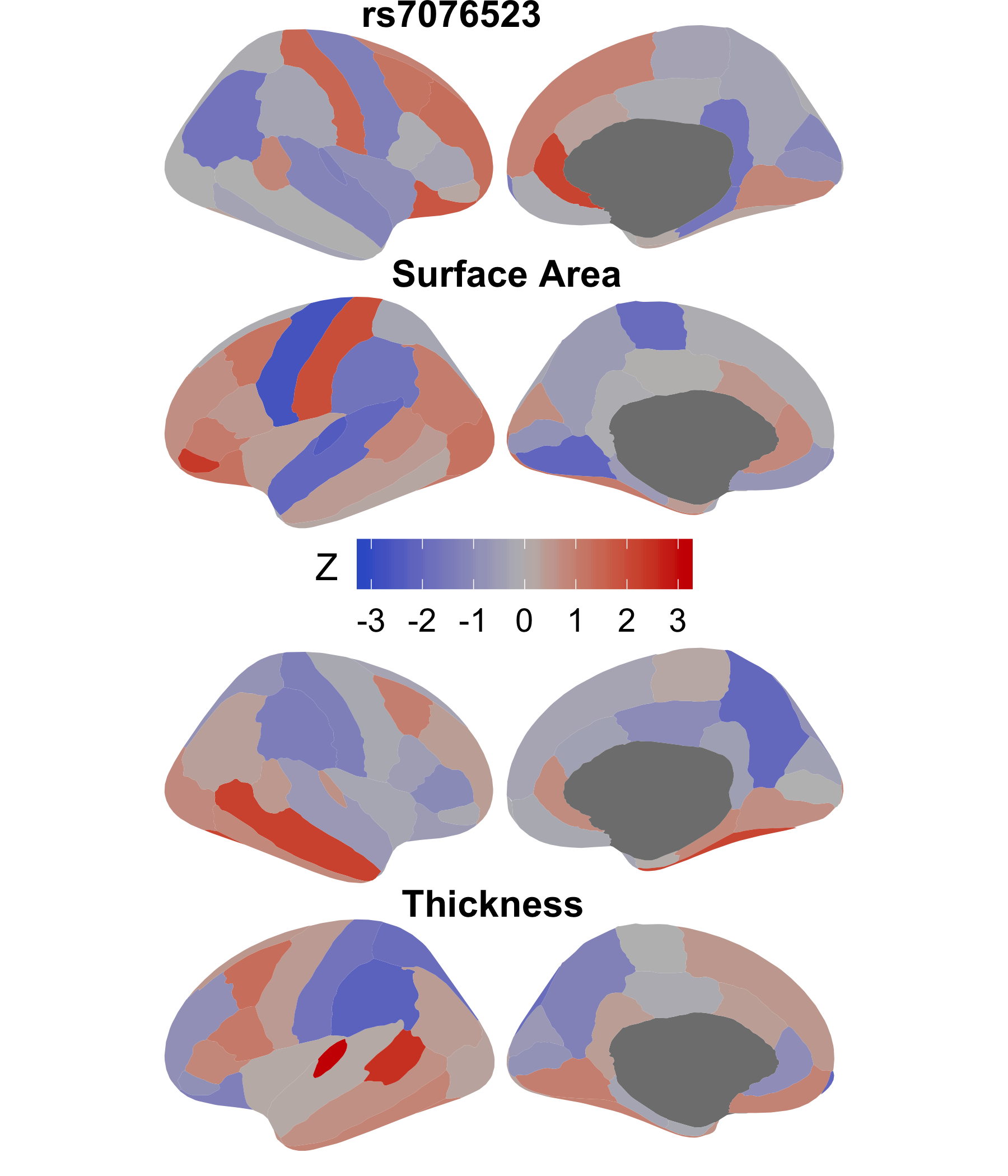

Supplement: Supplementary file 17 — Supplementary Data 14 [file 41467_2020_17368_MOESM17_ESM.gz › BrainMaps/most_aseg_vol/BrainMap165_rs7076523.png]

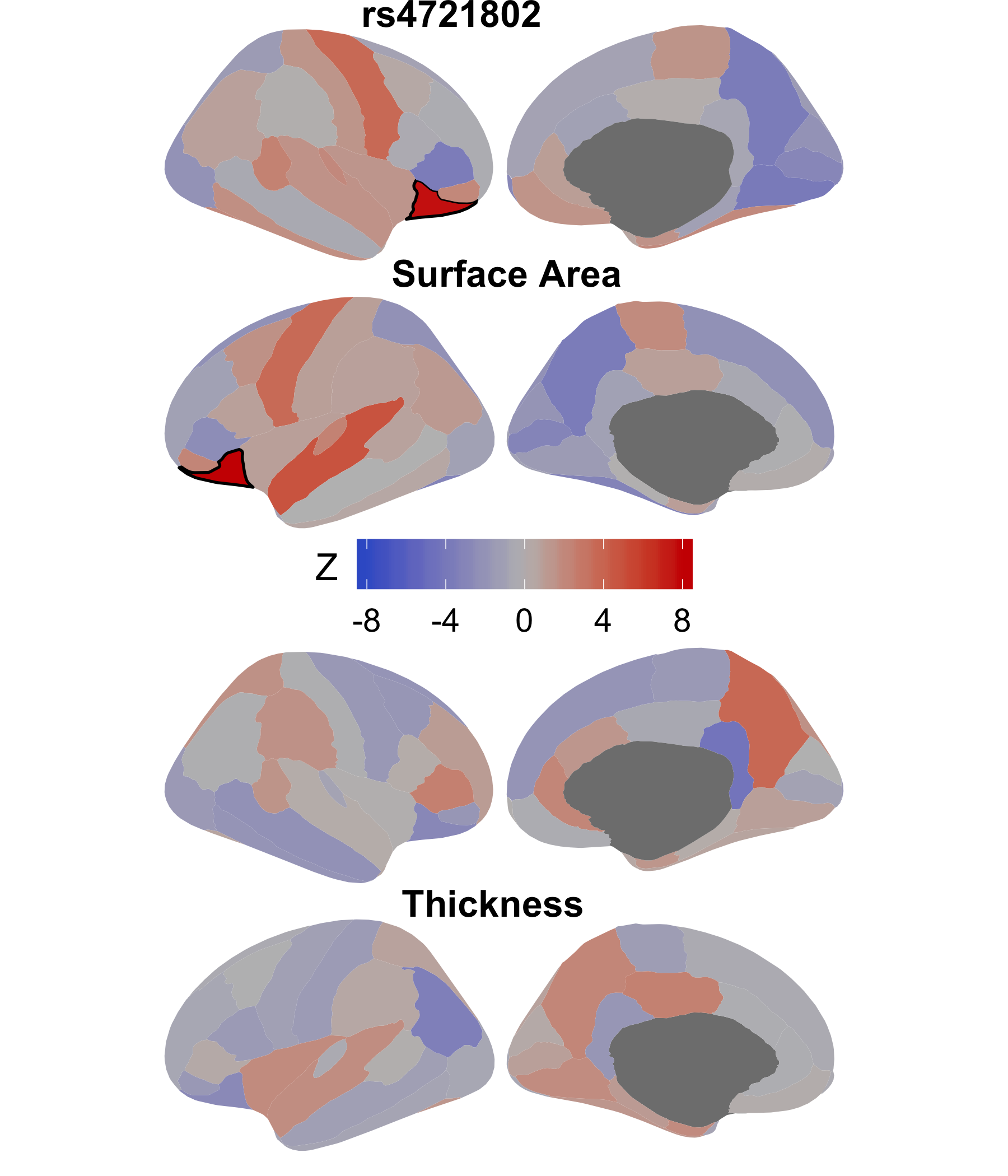

Supplement: Supplementary file 17 — Supplementary Data 14 [file 41467_2020_17368_MOESM17_ESM.gz › BrainMaps/most_aseg_vol/BrainMap114_rs4721802.png]

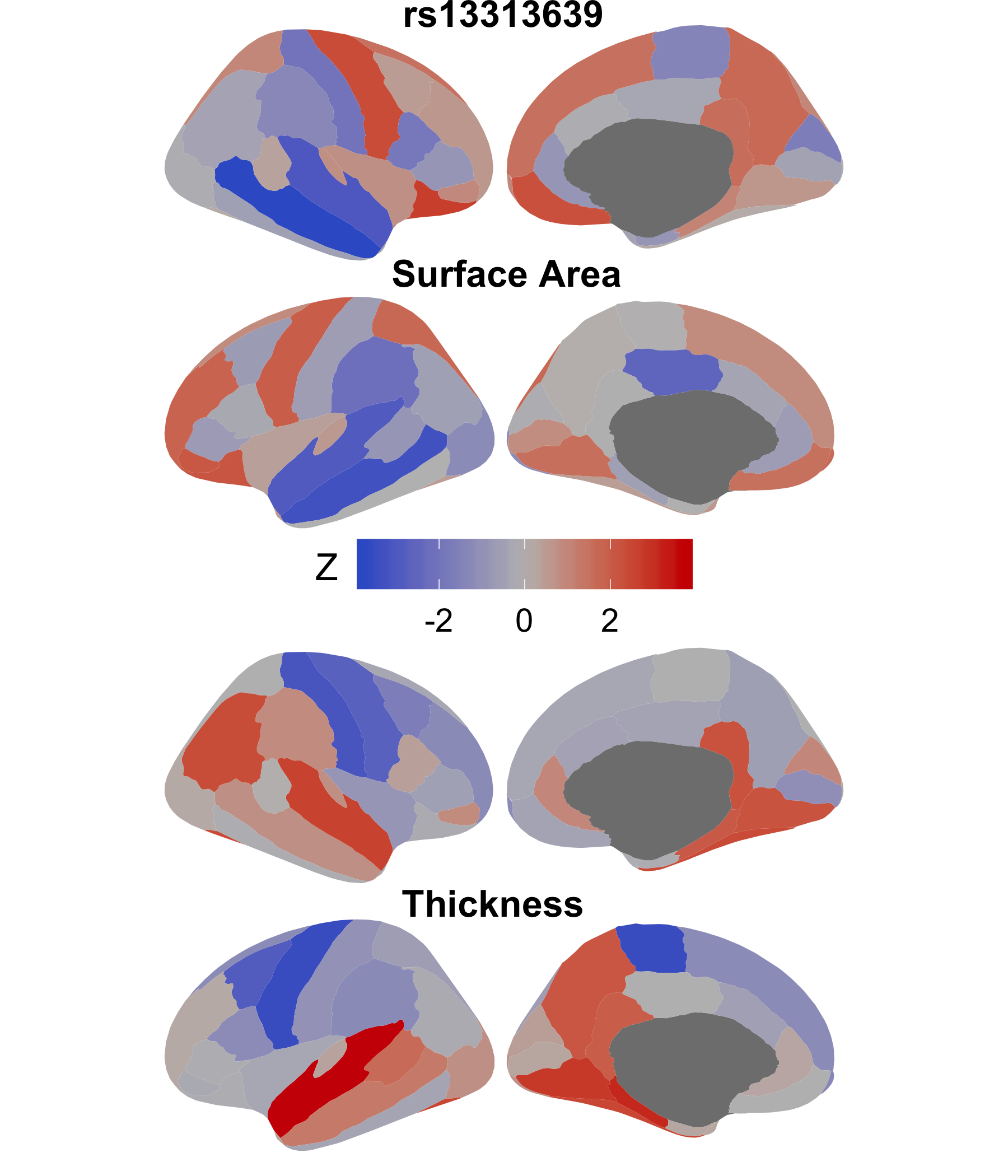

Supplement: Supplementary file 17 — Supplementary Data 14 [file 41467_2020_17368_MOESM17_ESM.gz › BrainMaps/most_aseg_vol/BrainMap092_rs13313639.png]

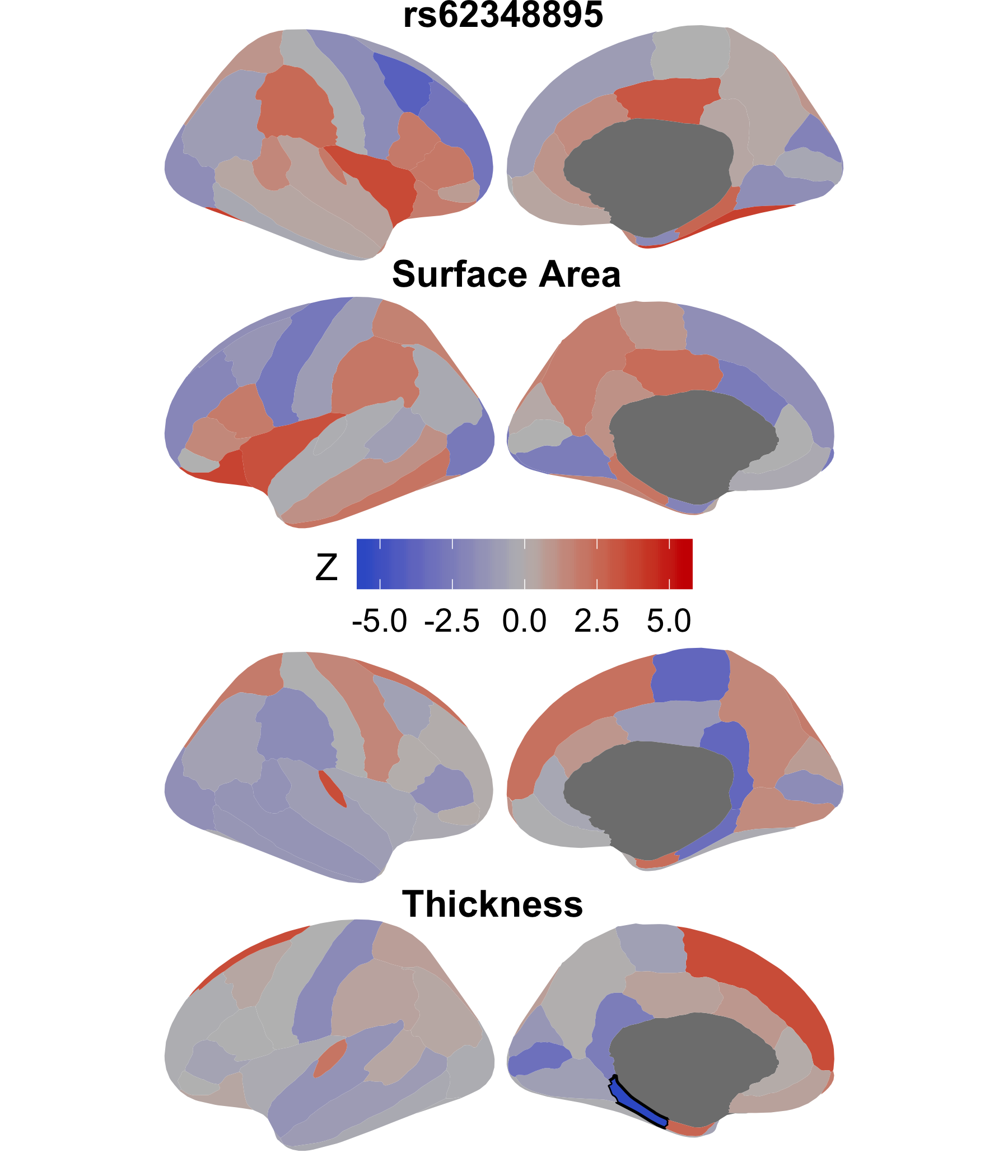

Supplement: Supplementary file 17 — Supplementary Data 14 [file 41467_2020_17368_MOESM17_ESM.gz › BrainMaps/most_aseg_vol/BrainMap074_rs62348895.png]

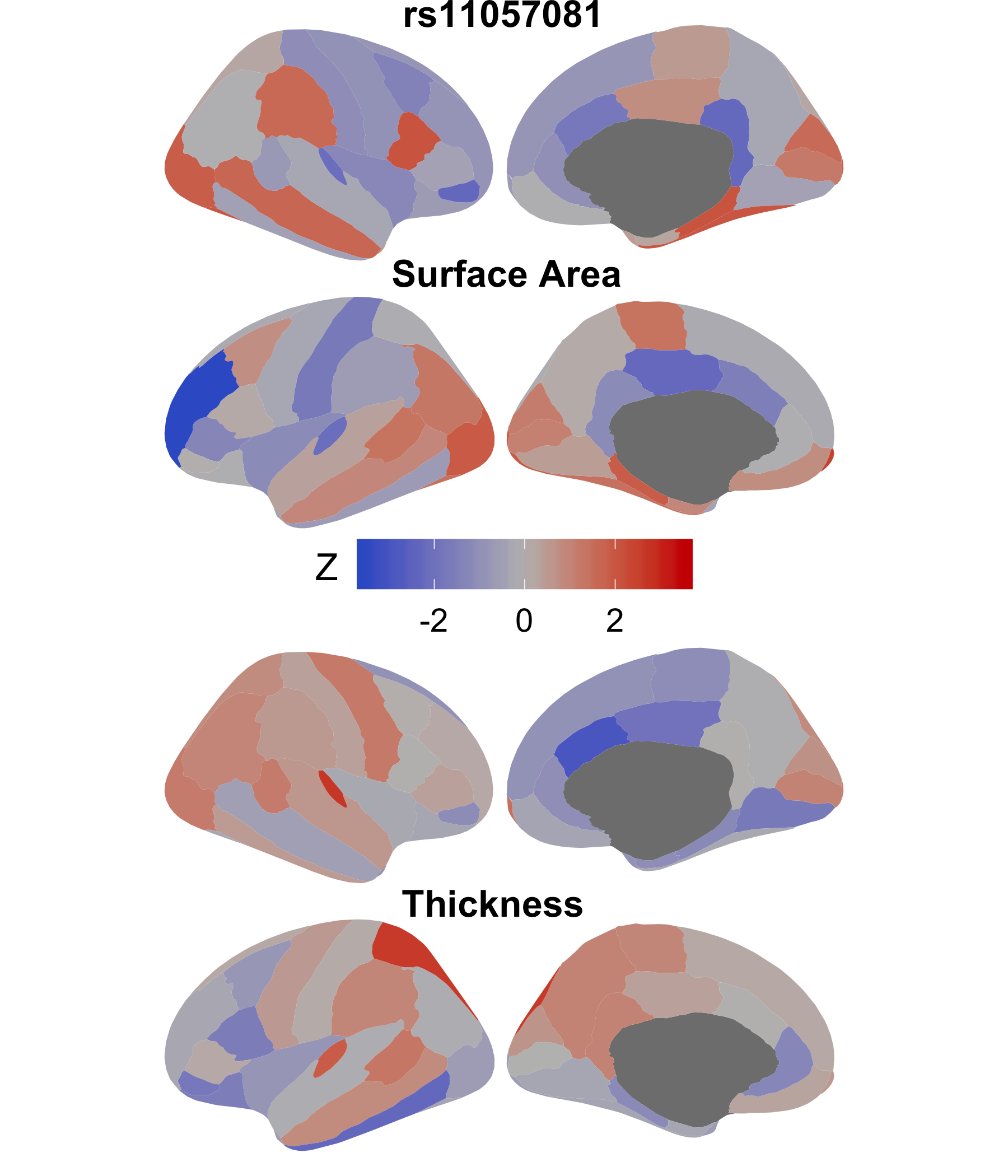

Supplement: Supplementary file 17 — Supplementary Data 14 [file 41467_2020_17368_MOESM17_ESM.gz › BrainMaps/most_aseg_vol/BrainMap070_rs11057081.png]

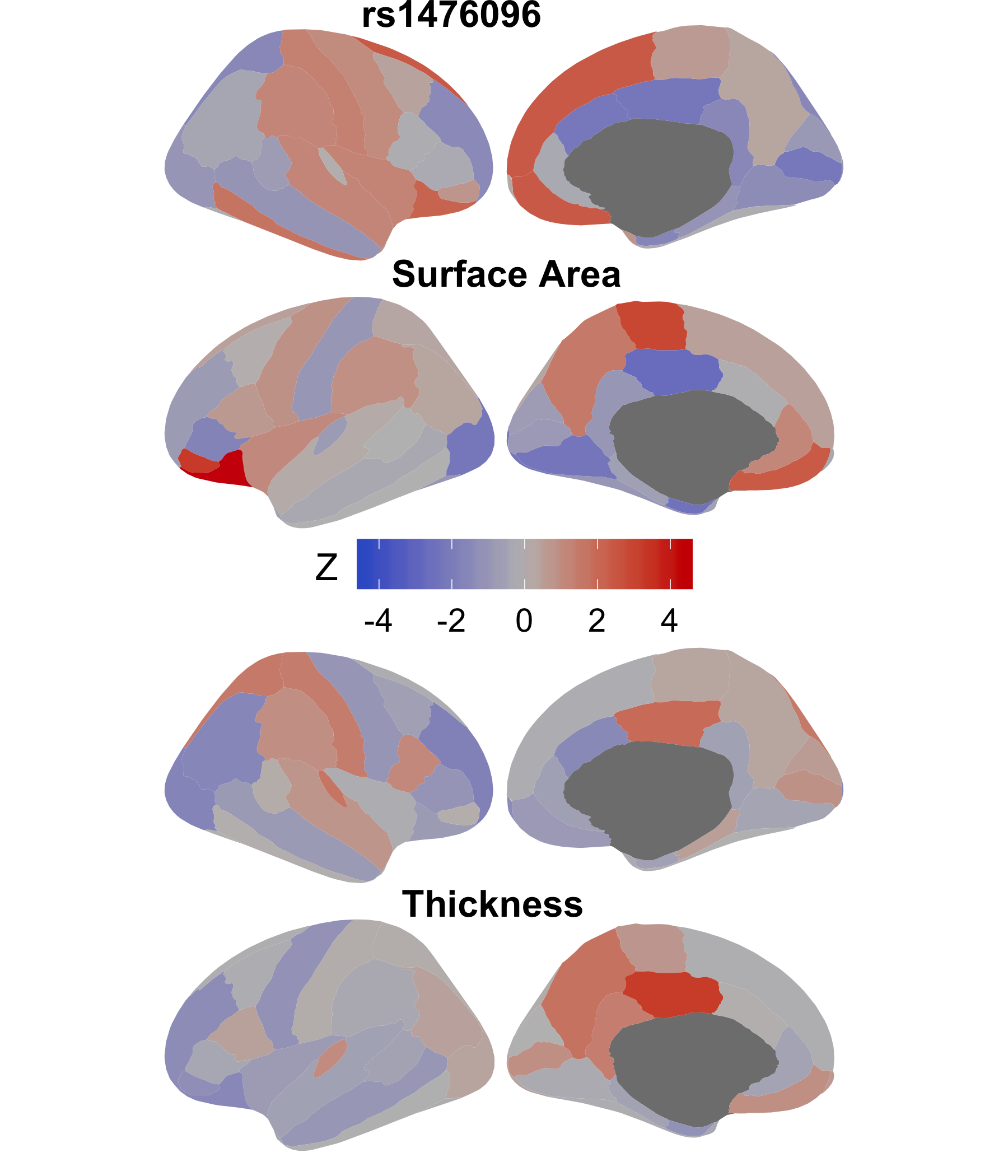

Supplement: Supplementary file 17 — Supplementary Data 14 [file 41467_2020_17368_MOESM17_ESM.gz › BrainMaps/most_aseg_vol/BrainMap033_rs1476096.png]

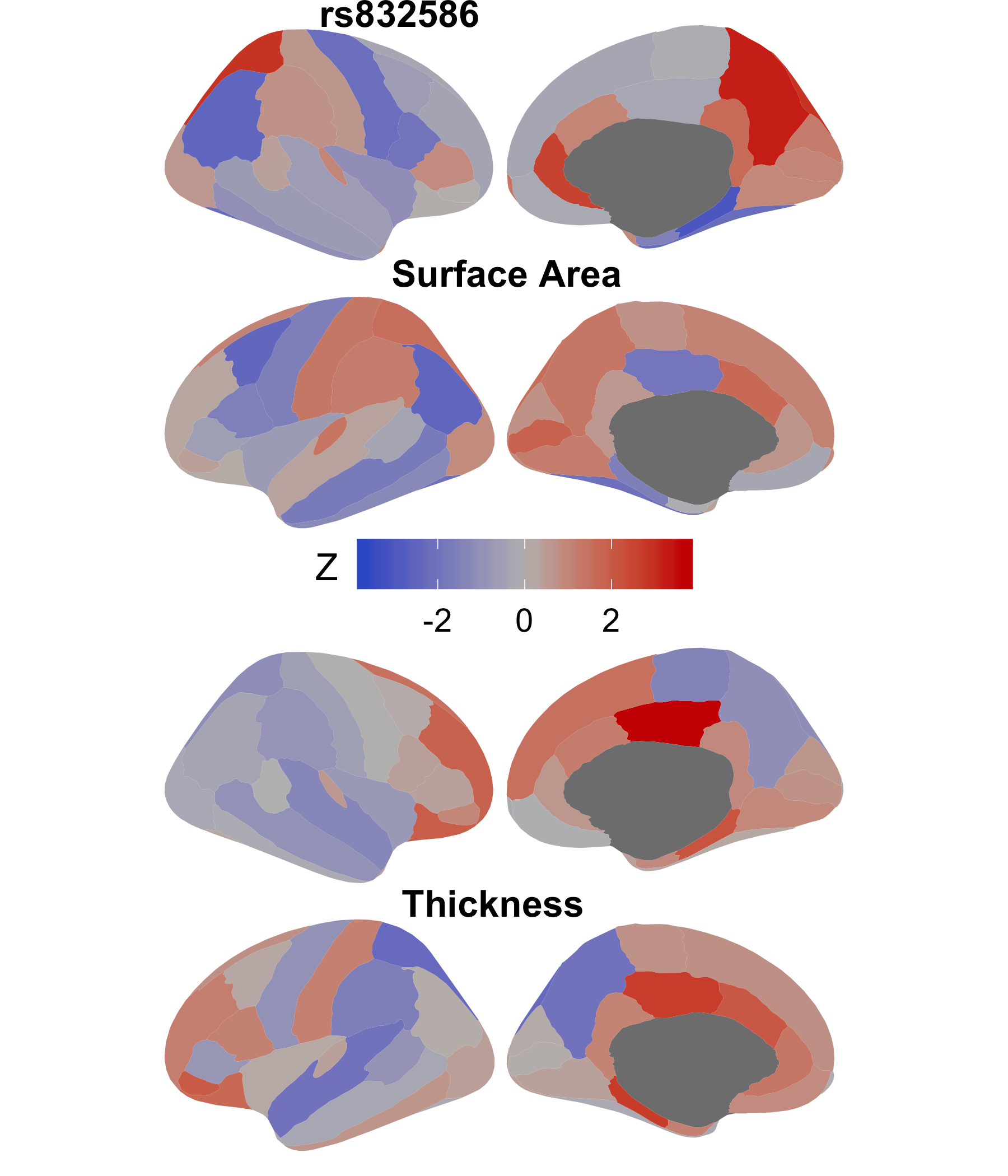

Supplement: Supplementary file 17 — Supplementary Data 14 [file 41467_2020_17368_MOESM17_ESM.gz › BrainMaps/most_aseg_vol/BrainMap136_rs832586.png]

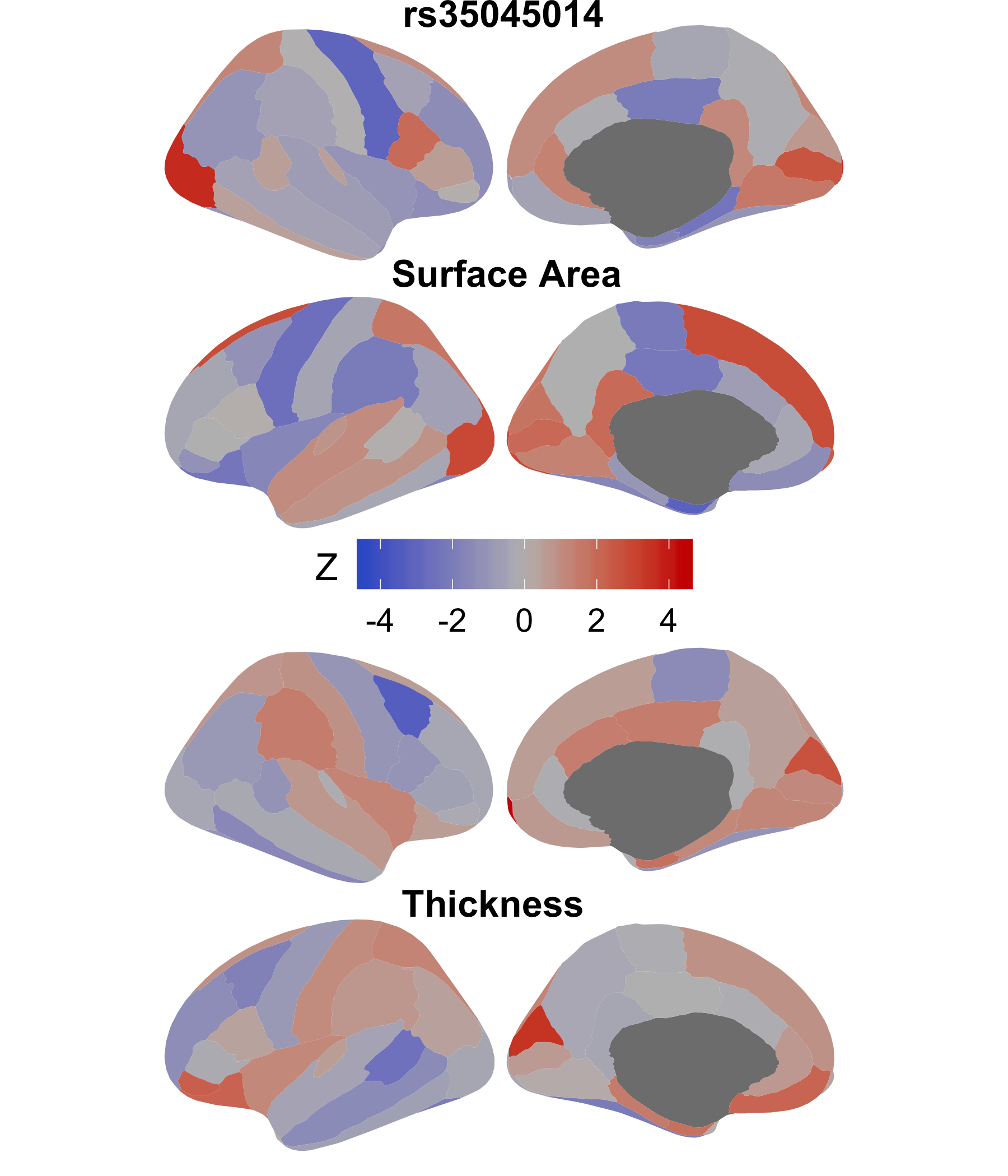

Supplement: Supplementary file 17 — Supplementary Data 14 [file 41467_2020_17368_MOESM17_ESM.gz › BrainMaps/most_aseg_vol/BrainMap107_rs35045014.png]

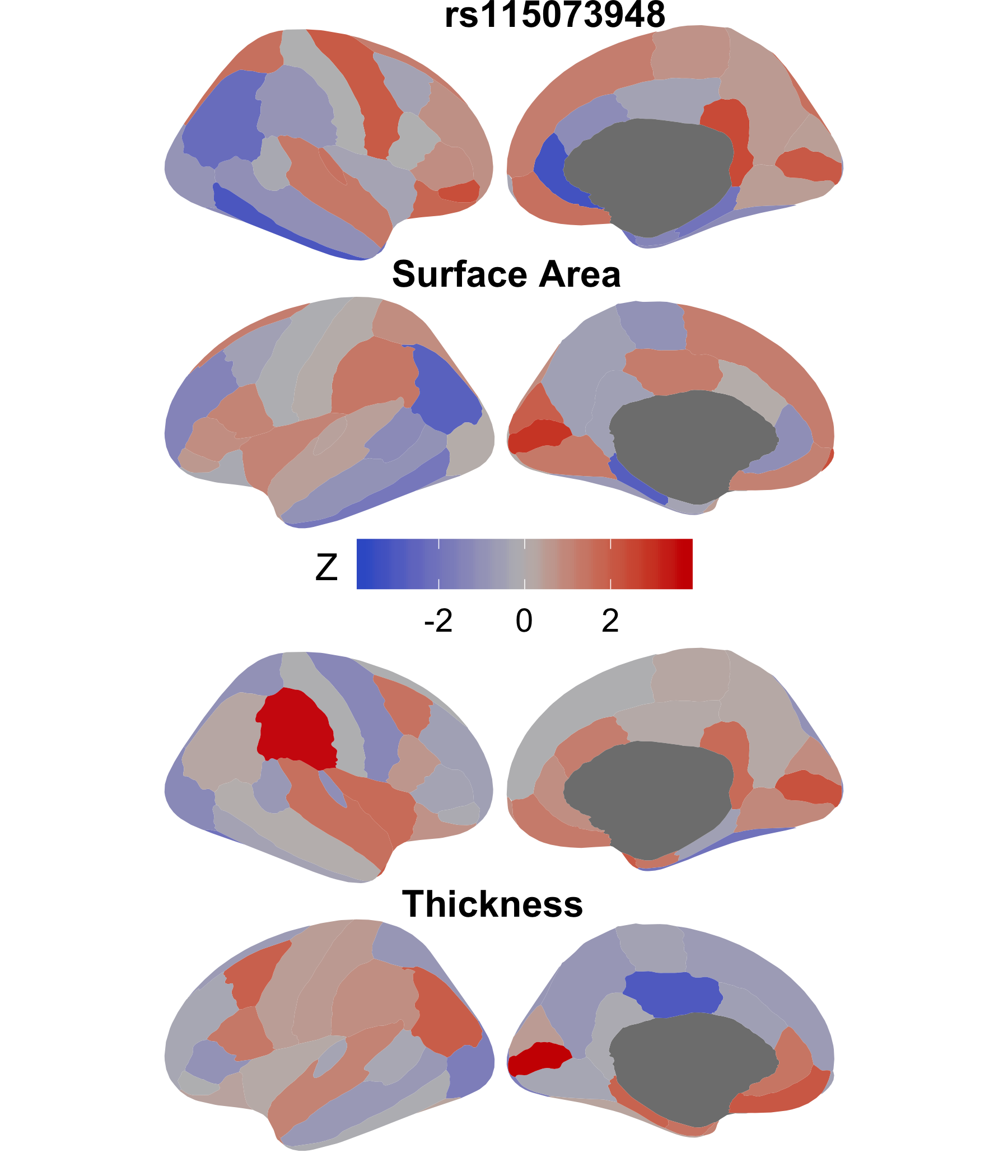

Supplement: Supplementary file 17 — Supplementary Data 14 [file 41467_2020_17368_MOESM17_ESM.gz › BrainMaps/most_aseg_vol/BrainMap173_rs115073948.png]

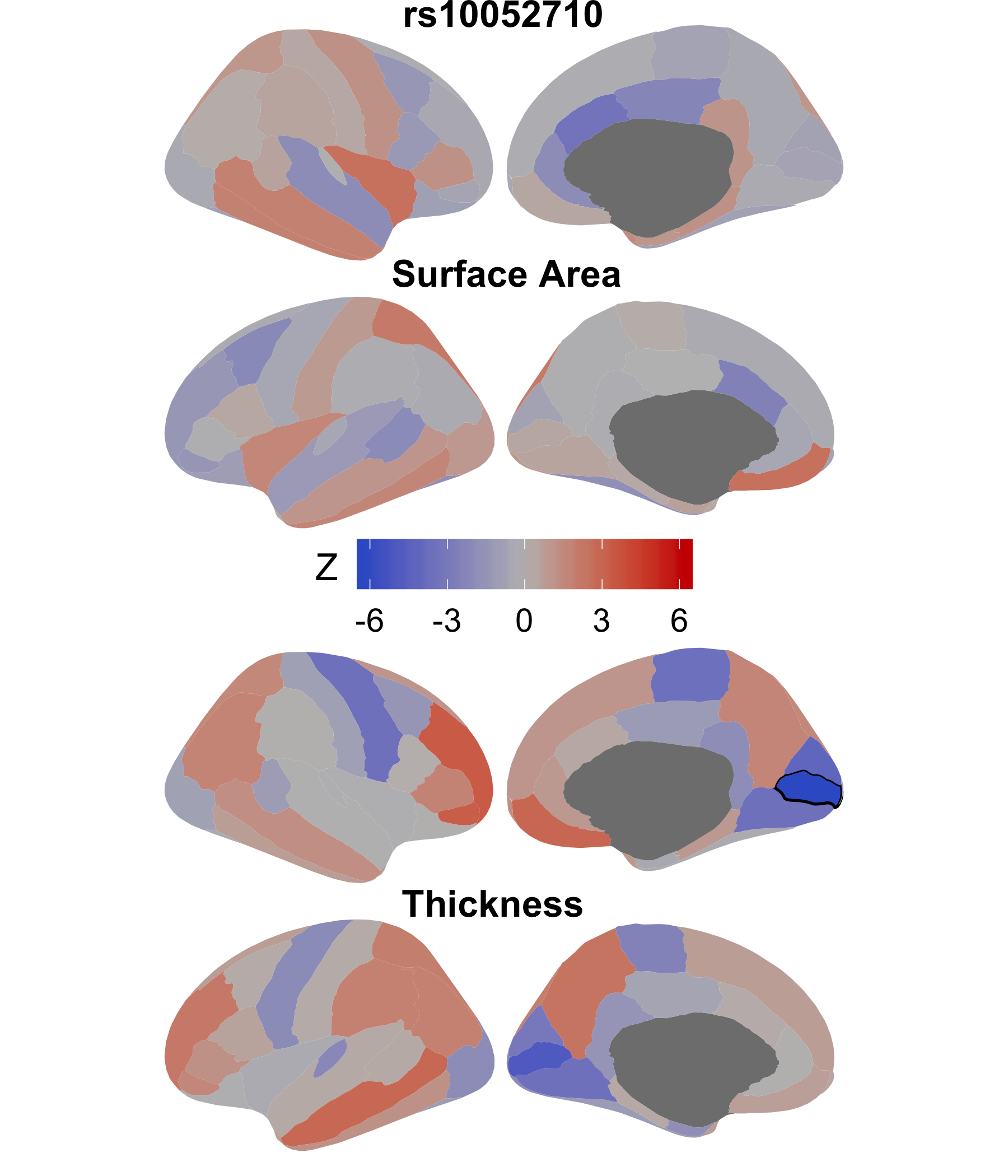

Supplement: Supplementary file 17 — Supplementary Data 14 [file 41467_2020_17368_MOESM17_ESM.gz › BrainMaps/most_aseg_vol/BrainMap072_rs10052710.png]

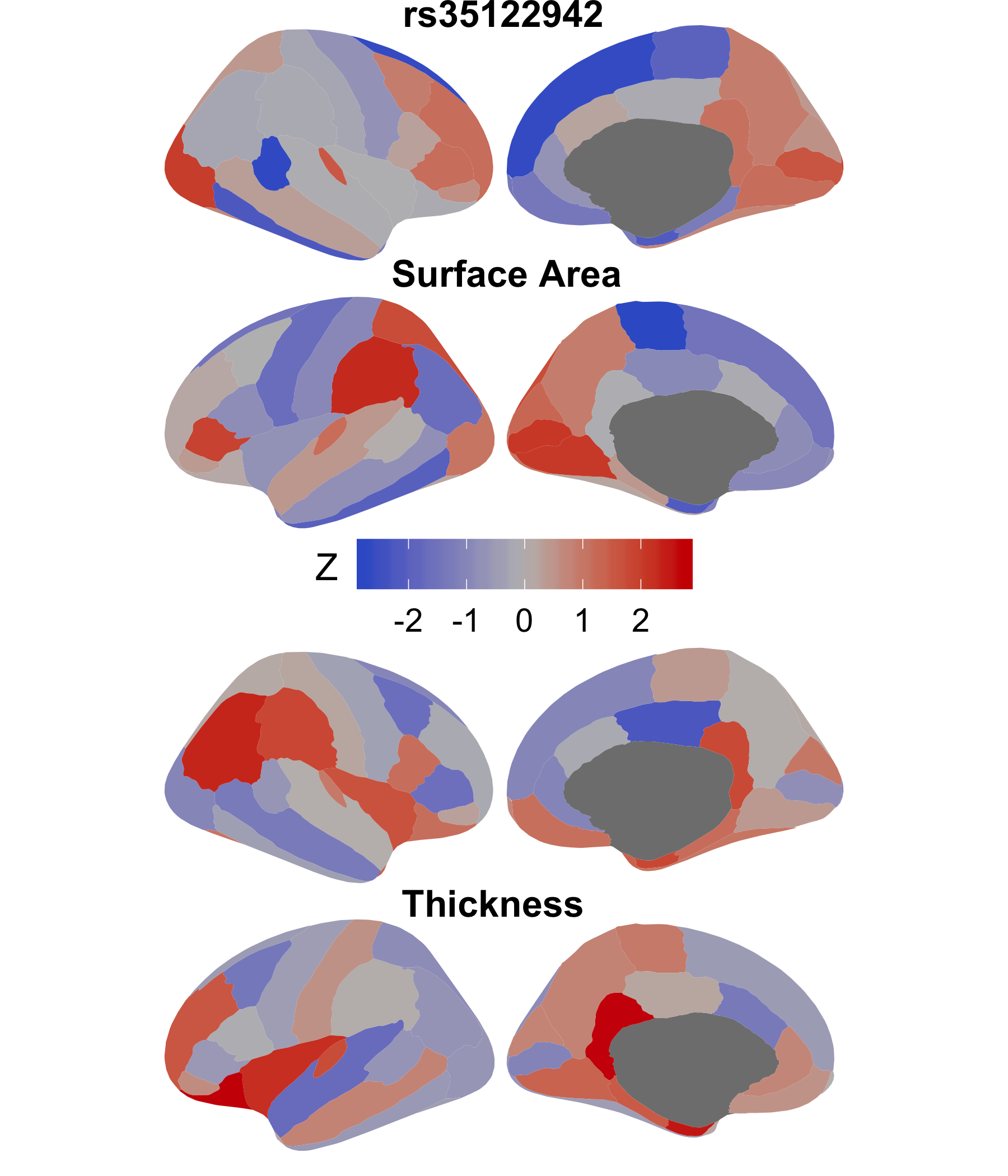

Supplement: Supplementary file 17 — Supplementary Data 14 [file 41467_2020_17368_MOESM17_ESM.gz › BrainMaps/most_aseg_vol/BrainMap080_rs35122942.png]

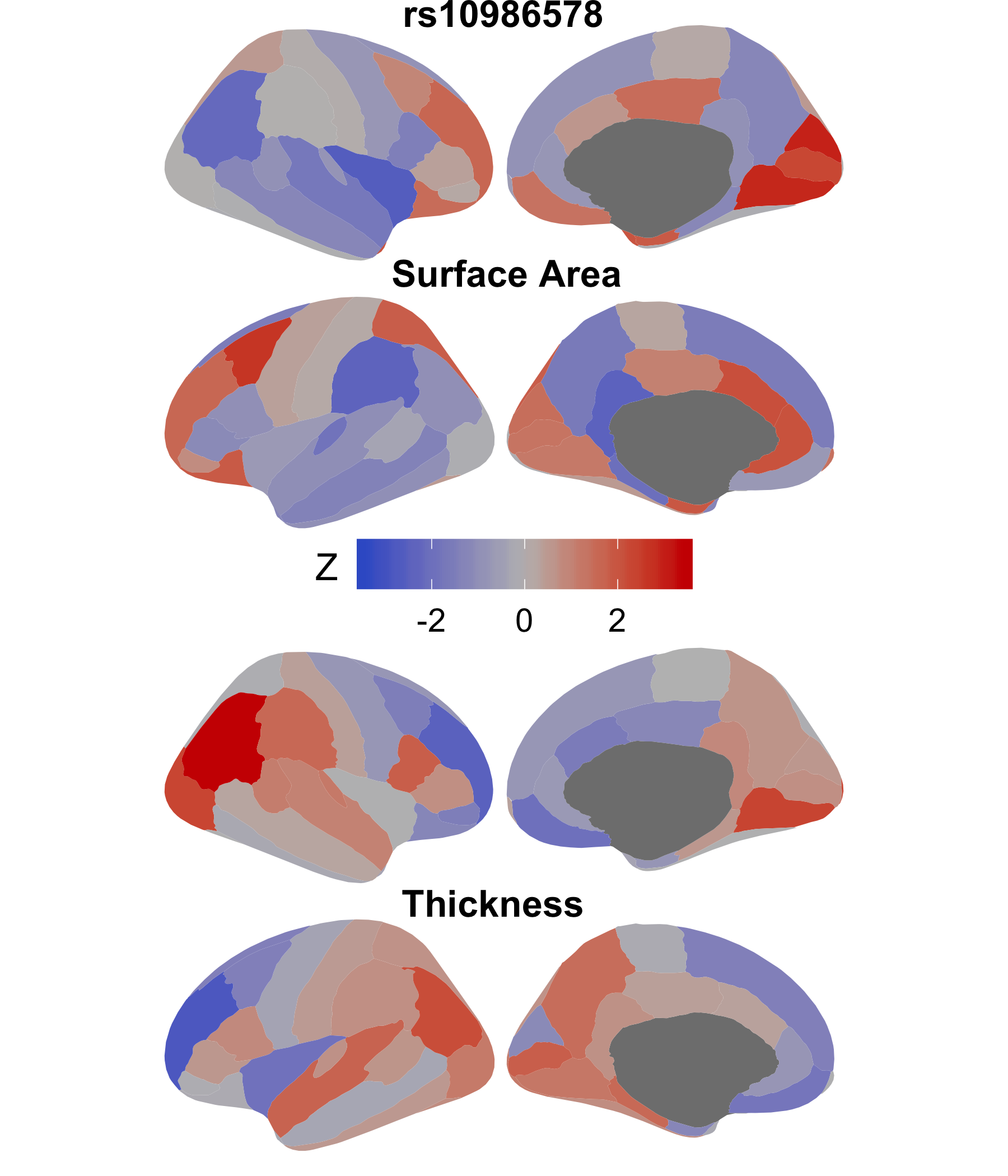

Supplement: Supplementary file 17 — Supplementary Data 14 [file 41467_2020_17368_MOESM17_ESM.gz › BrainMaps/most_aseg_vol/BrainMap140_rs10986578.png]

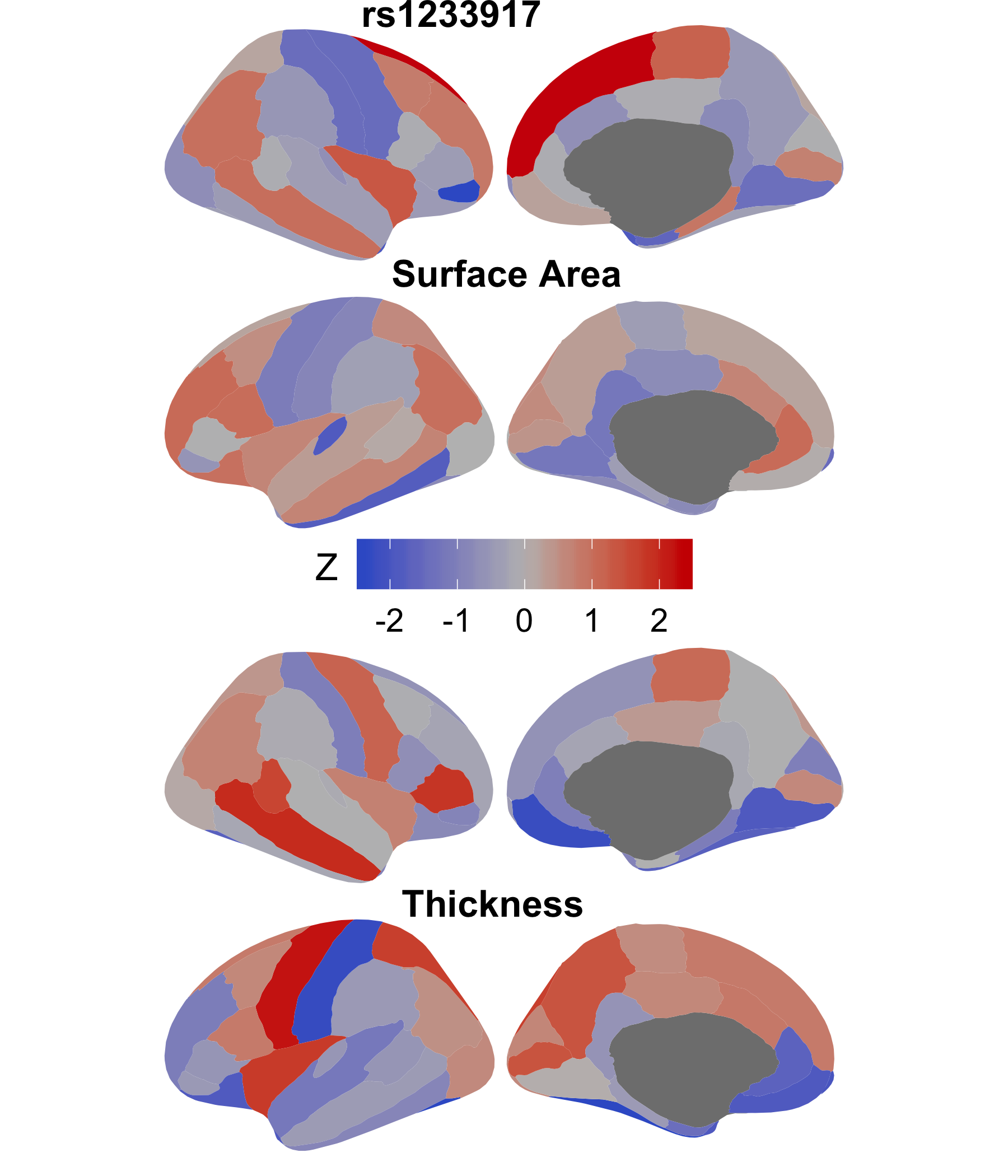

Supplement: Supplementary file 17 — Supplementary Data 14 [file 41467_2020_17368_MOESM17_ESM.gz › BrainMaps/most_aseg_vol/BrainMap030_rs1233917.png]

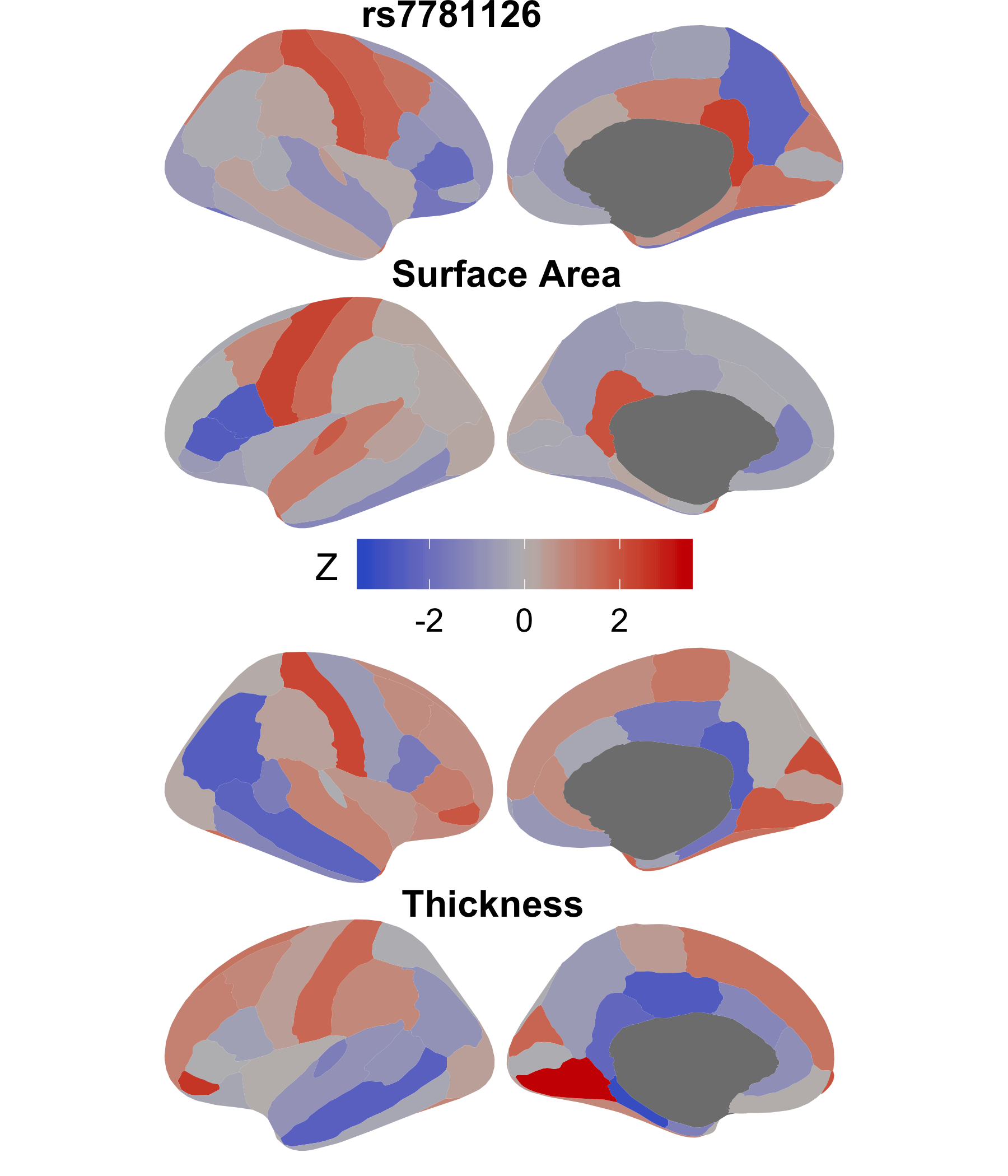

Supplement: Supplementary file 17 — Supplementary Data 14 [file 41467_2020_17368_MOESM17_ESM.gz › BrainMaps/most_aseg_vol/BrainMap126_rs7781126.png]

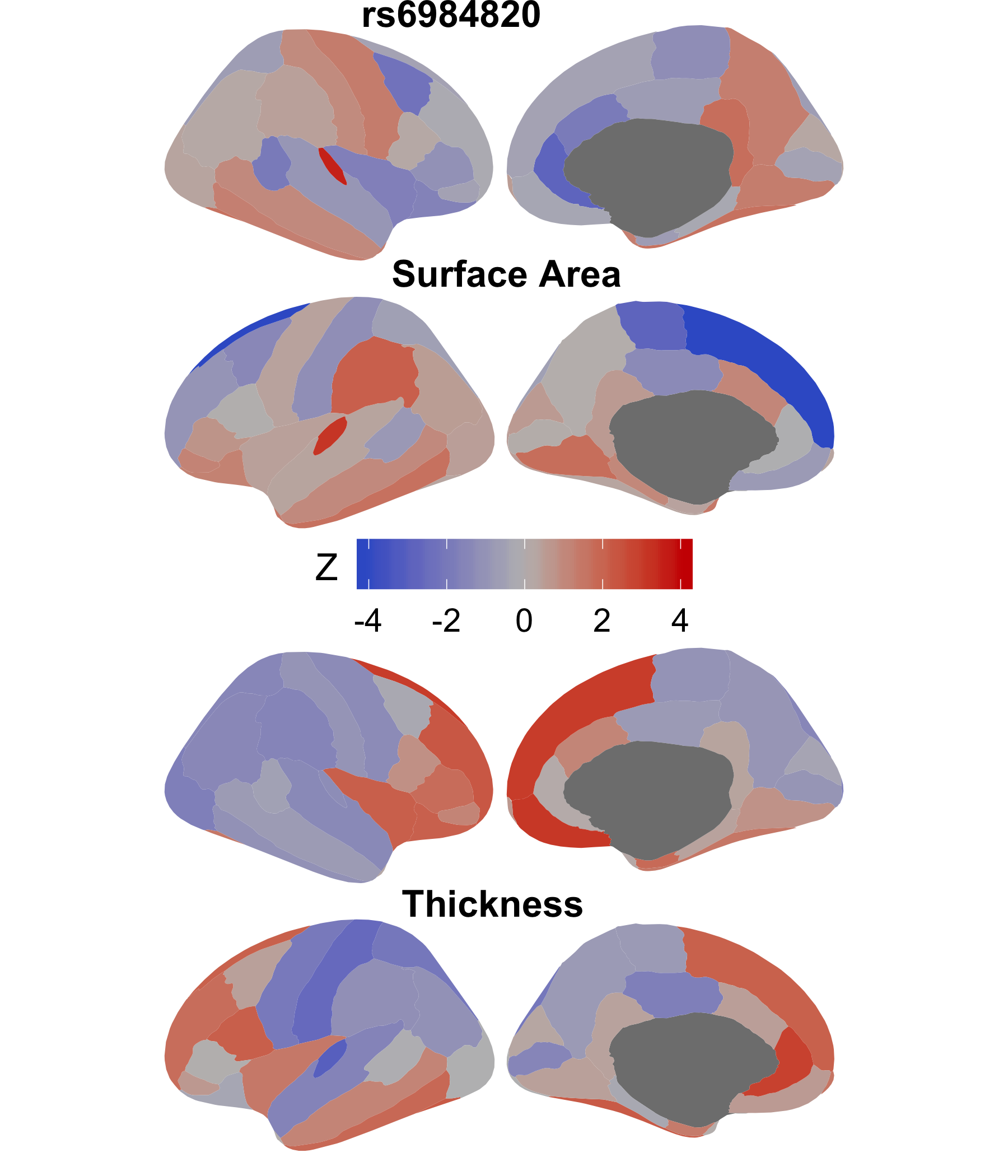

Supplement: Supplementary file 17 — Supplementary Data 14 [file 41467_2020_17368_MOESM17_ESM.gz › BrainMaps/most_aseg_vol/BrainMap169_rs6984820.png]

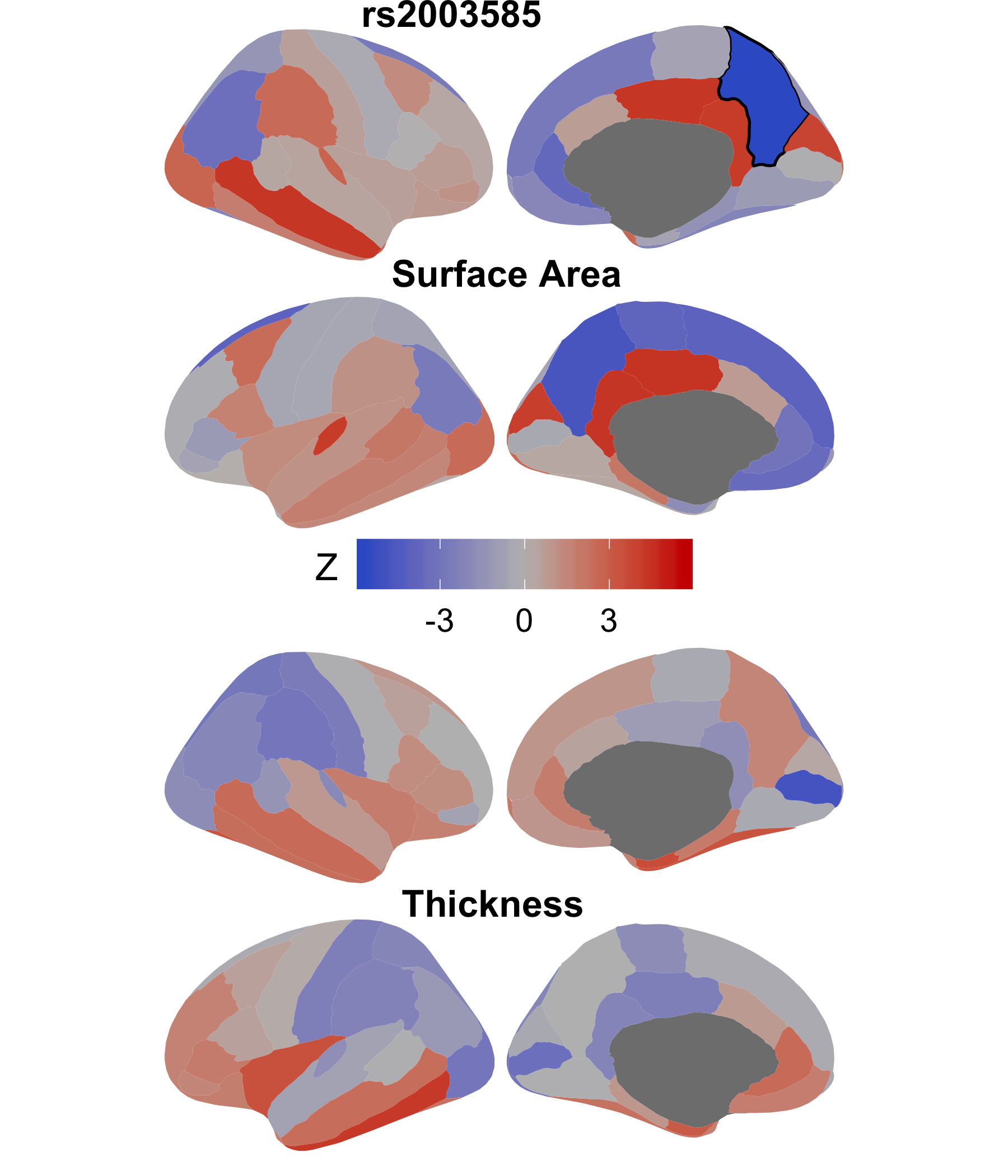

Supplement: Supplementary file 17 — Supplementary Data 14 [file 41467_2020_17368_MOESM17_ESM.gz › BrainMaps/most_aseg_vol/BrainMap029_rs2003585.png]

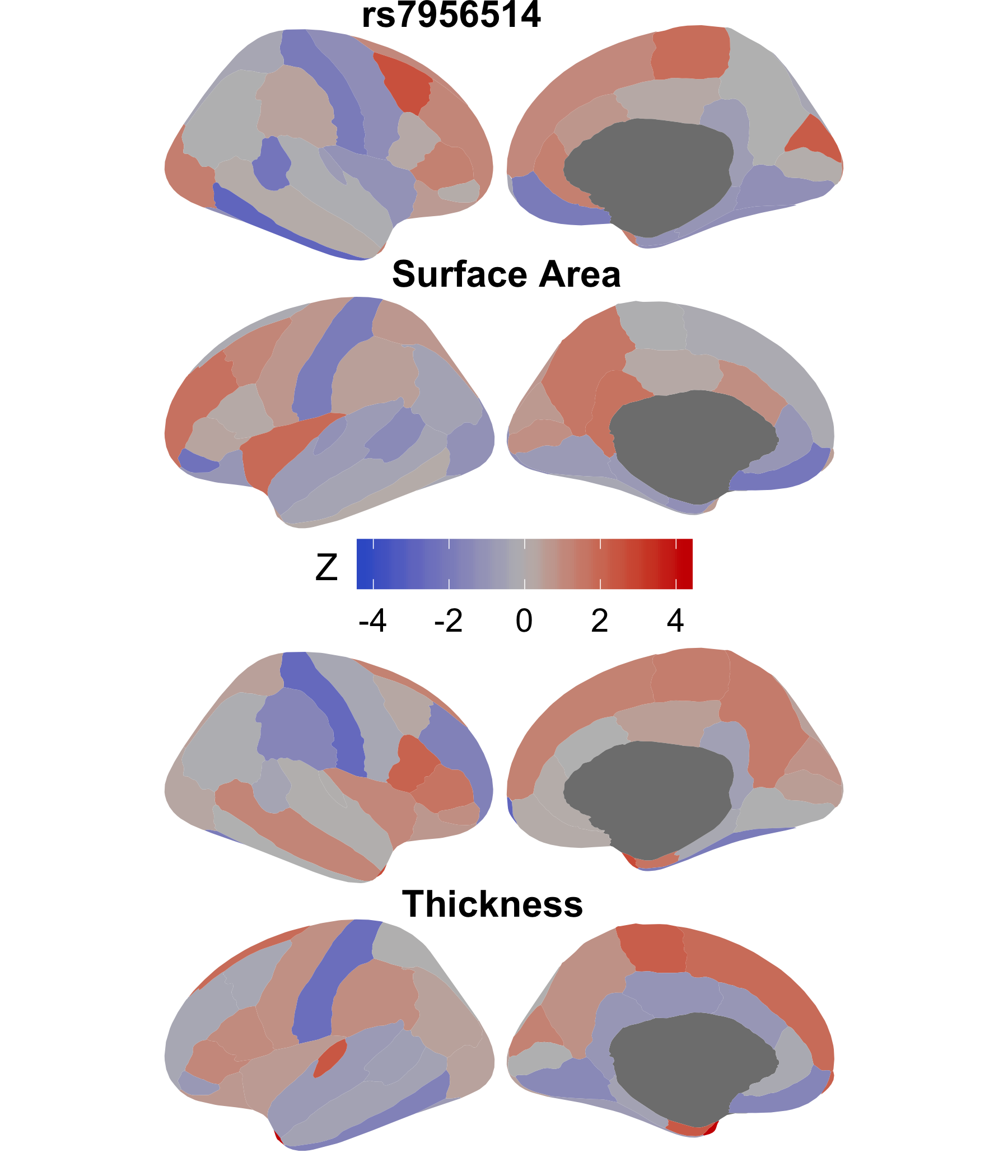

Supplement: Supplementary file 17 — Supplementary Data 14 [file 41467_2020_17368_MOESM17_ESM.gz › BrainMaps/most_aseg_vol/BrainMap122_rs7956514.png]

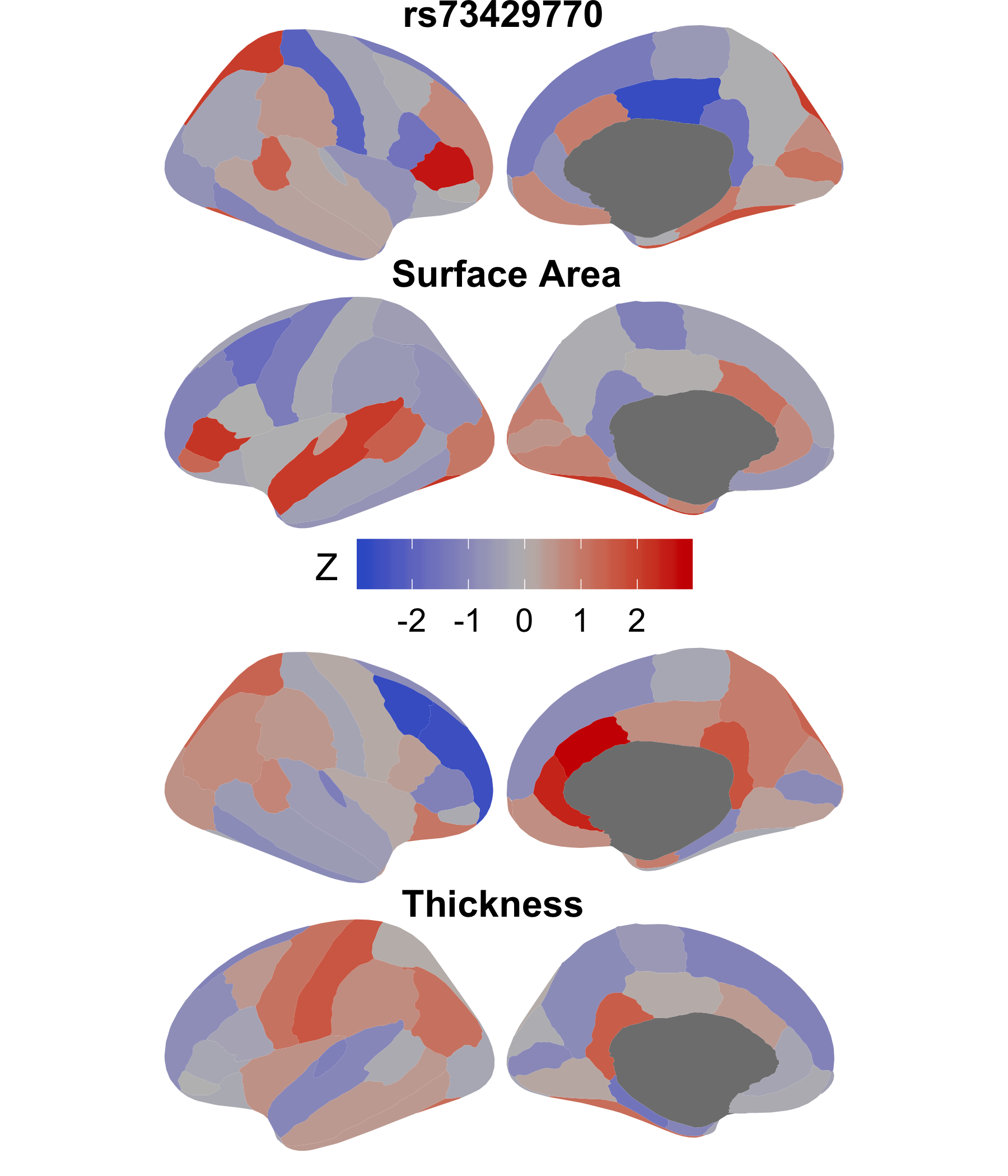

Supplement: Supplementary file 17 — Supplementary Data 14 [file 41467_2020_17368_MOESM17_ESM.gz › BrainMaps/most_aseg_vol/BrainMap050_rs73429770.png]

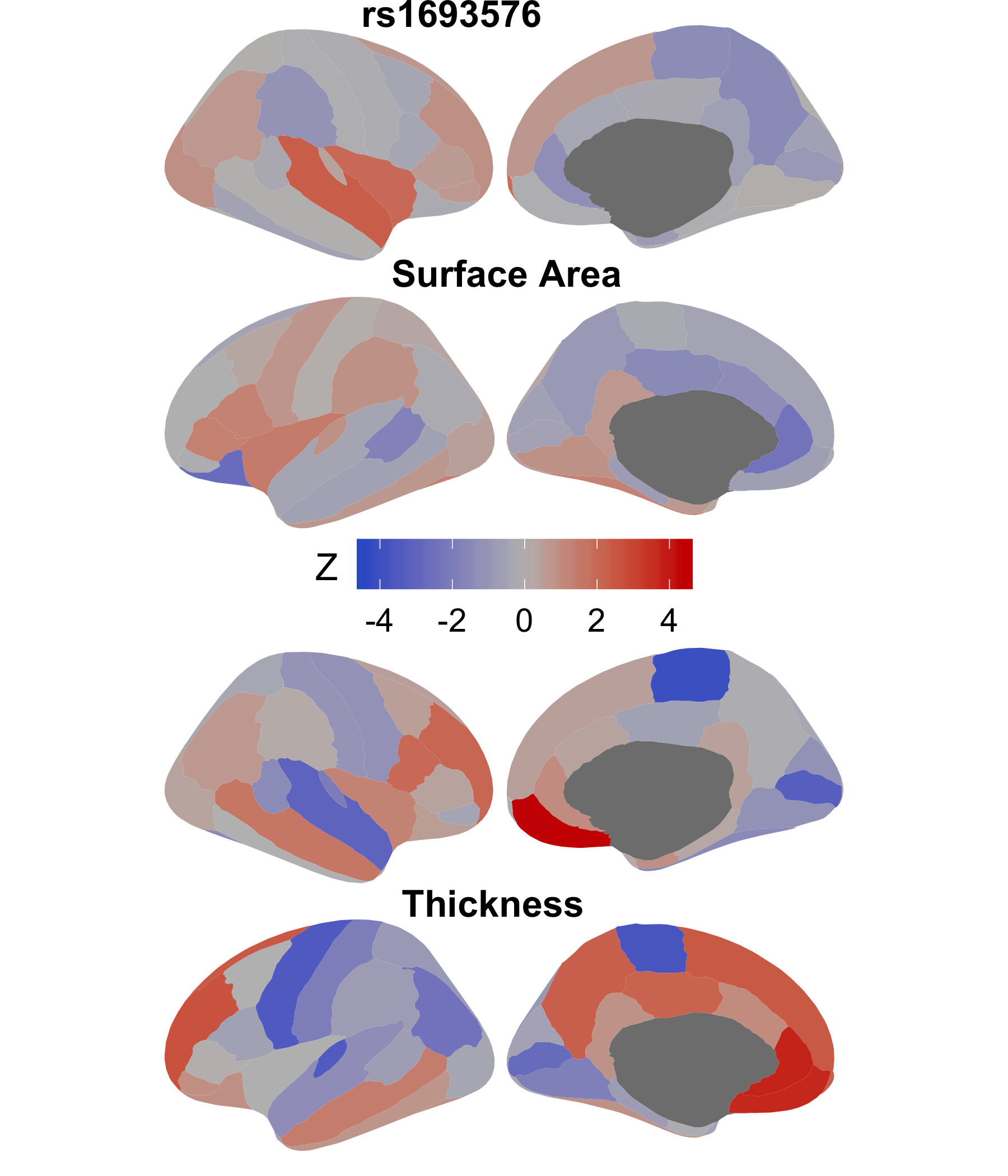

Supplement: Supplementary file 17 — Supplementary Data 14 [file 41467_2020_17368_MOESM17_ESM.gz › BrainMaps/most_aseg_vol/BrainMap037_rs1693576.png]

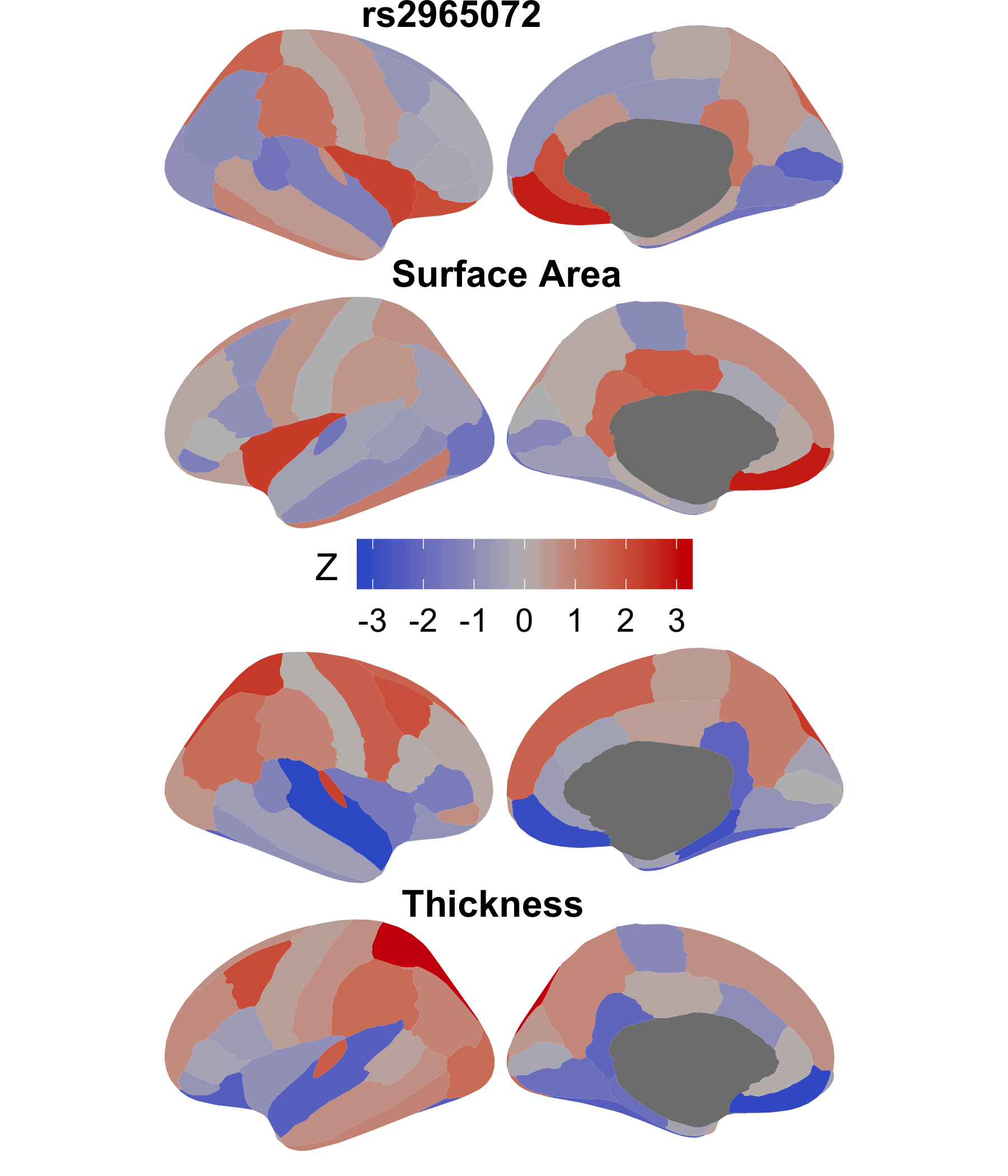

Supplement: Supplementary file 17 — Supplementary Data 14 [file 41467_2020_17368_MOESM17_ESM.gz › BrainMaps/most_aseg_vol/BrainMap095_rs2965072.png]

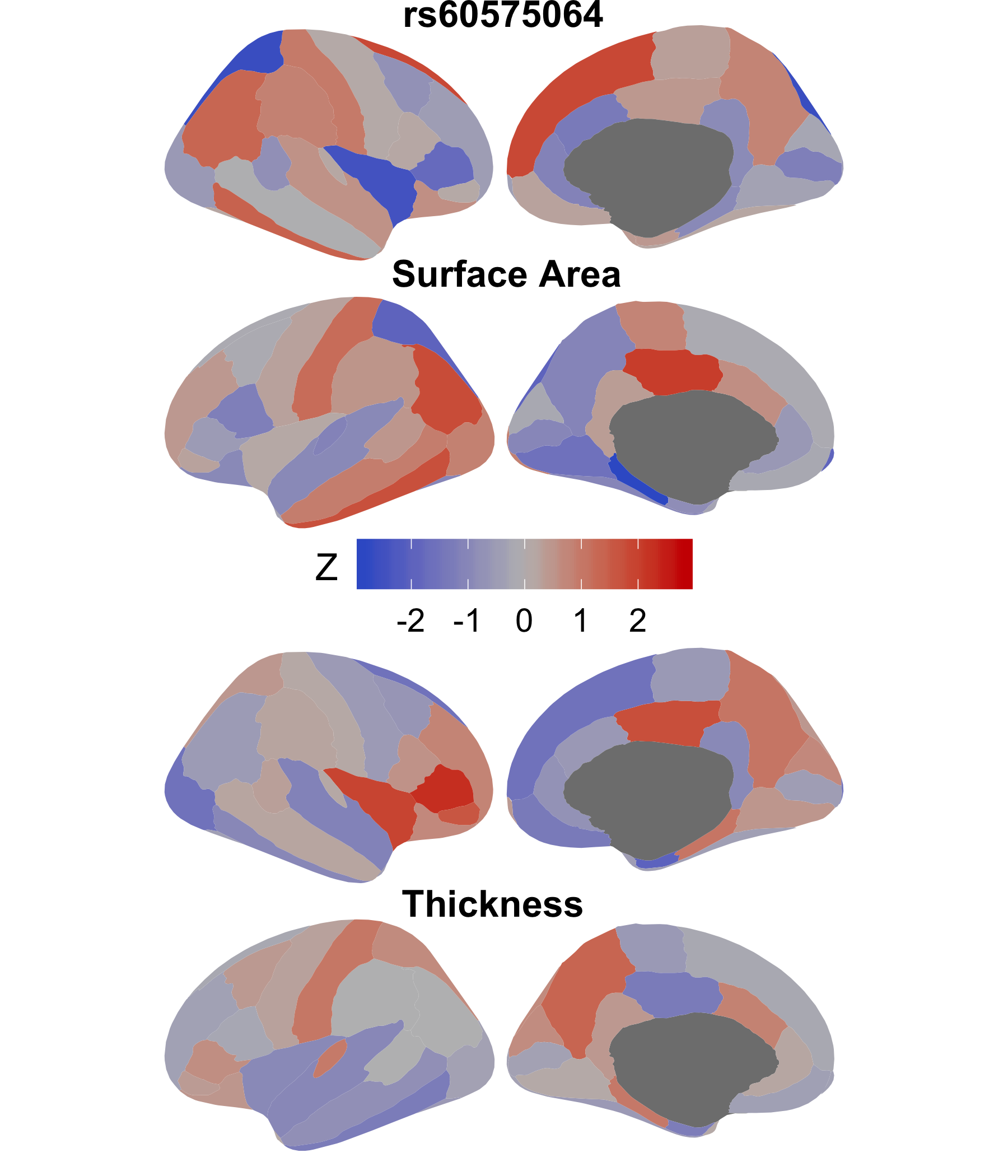

Supplement: Supplementary file 17 — Supplementary Data 14 [file 41467_2020_17368_MOESM17_ESM.gz › BrainMaps/most_aseg_vol/BrainMap008_rs60575064.png]

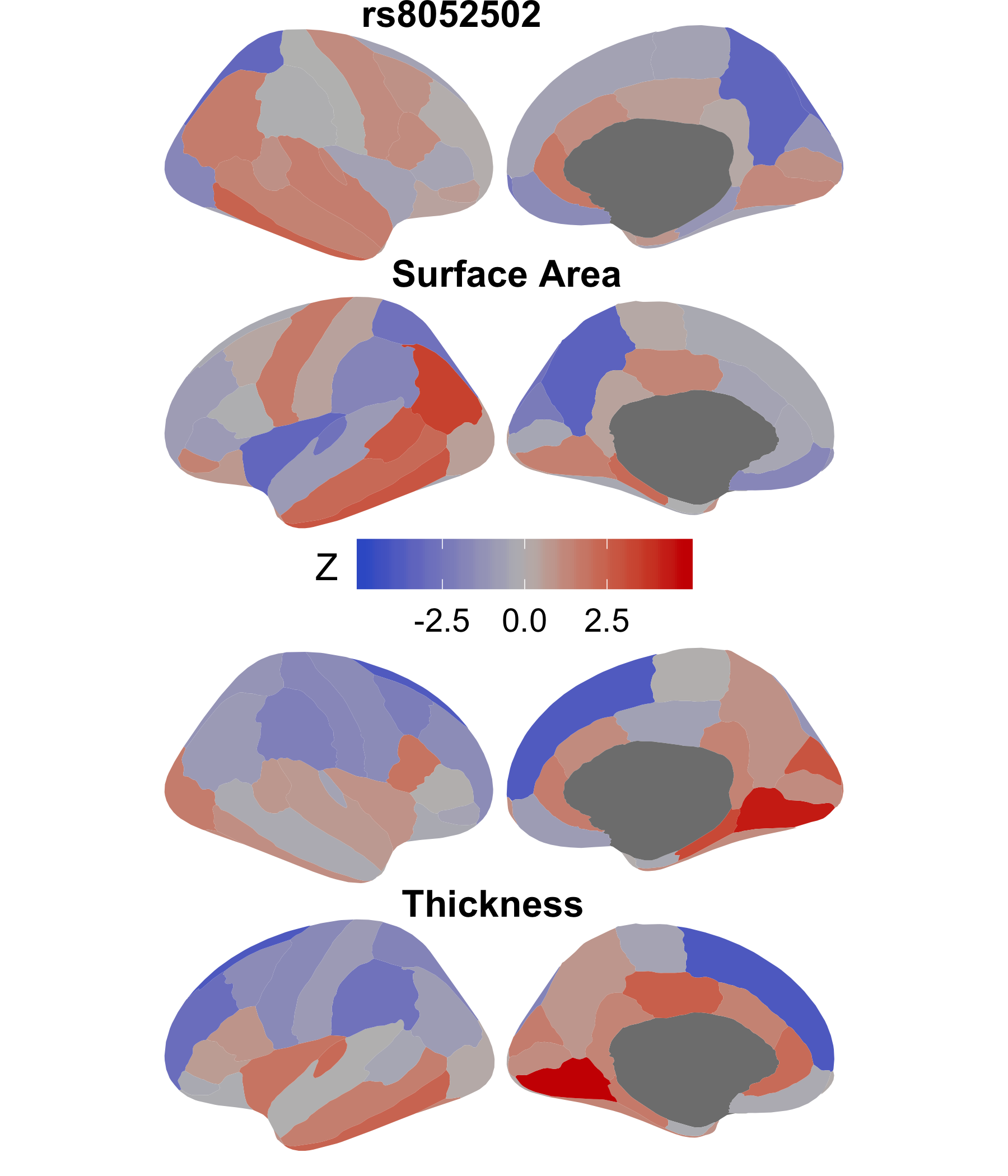

Supplement: Supplementary file 17 — Supplementary Data 14 [file 41467_2020_17368_MOESM17_ESM.gz › BrainMaps/most_aseg_vol/BrainMap148_rs8052502.png]

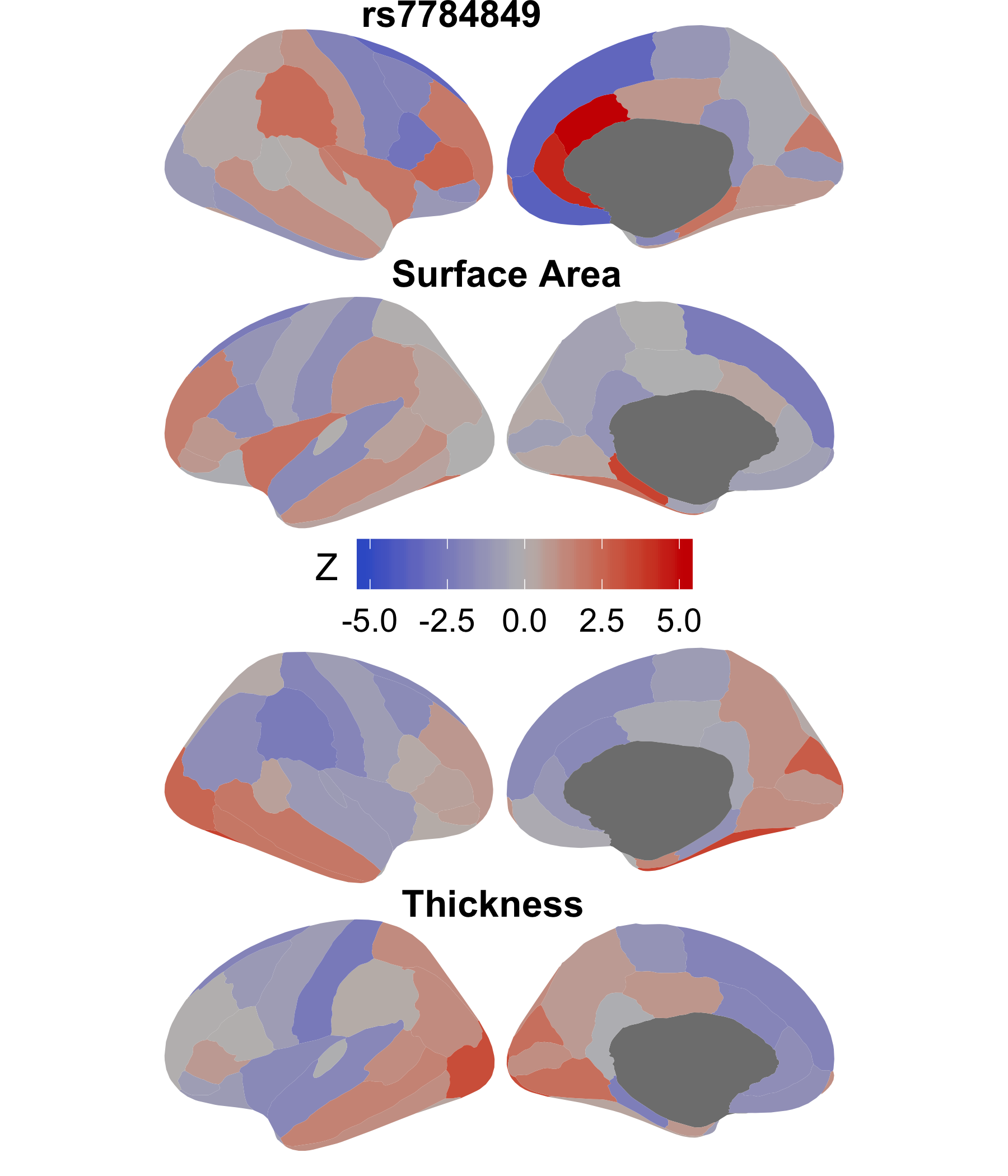

Supplement: Supplementary file 17 — Supplementary Data 14 [file 41467_2020_17368_MOESM17_ESM.gz › BrainMaps/most_aseg_vol/BrainMap143_rs7784849.png]

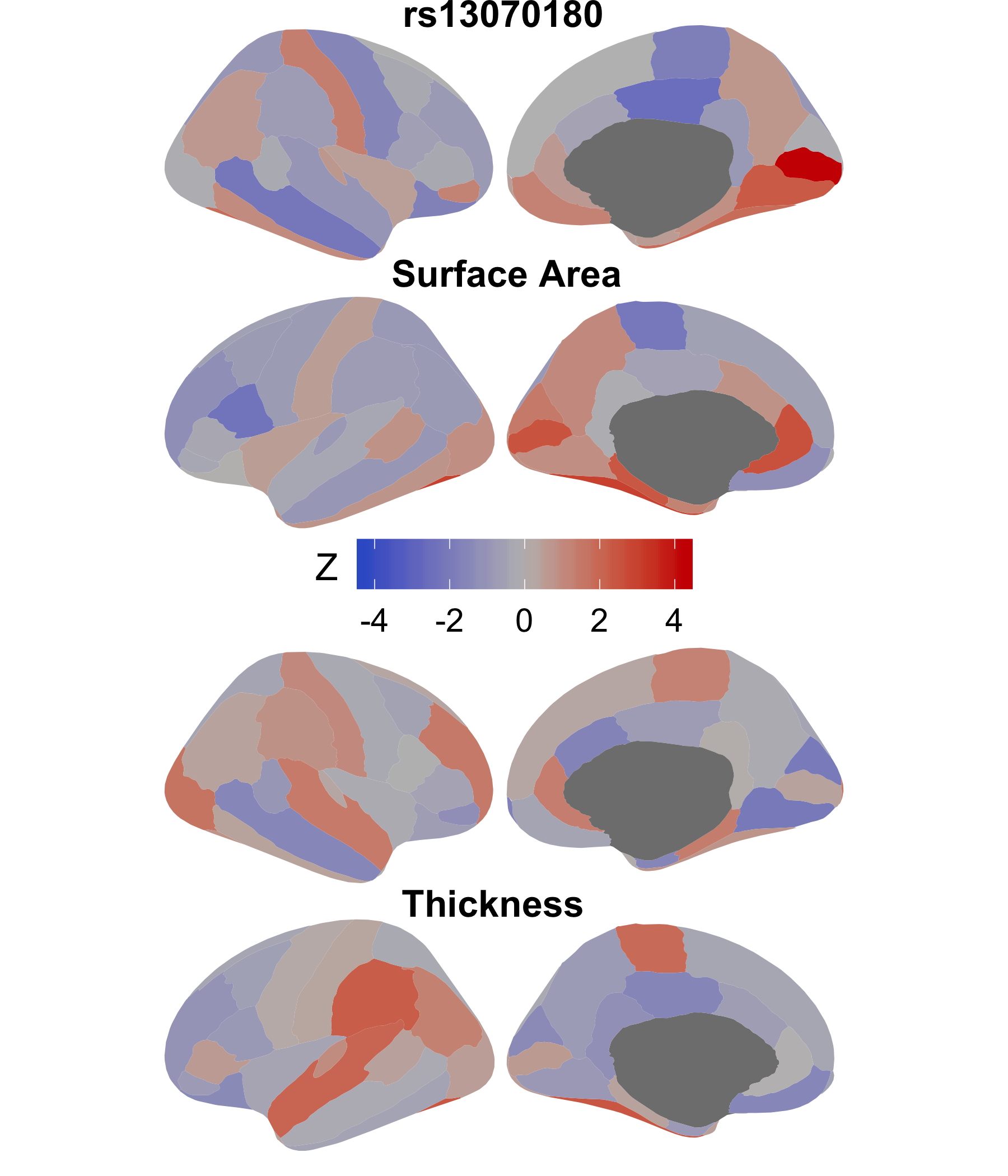

Supplement: Supplementary file 17 — Supplementary Data 14 [file 41467_2020_17368_MOESM17_ESM.gz › BrainMaps/most_aseg_vol/BrainMap088_rs13070180.png]

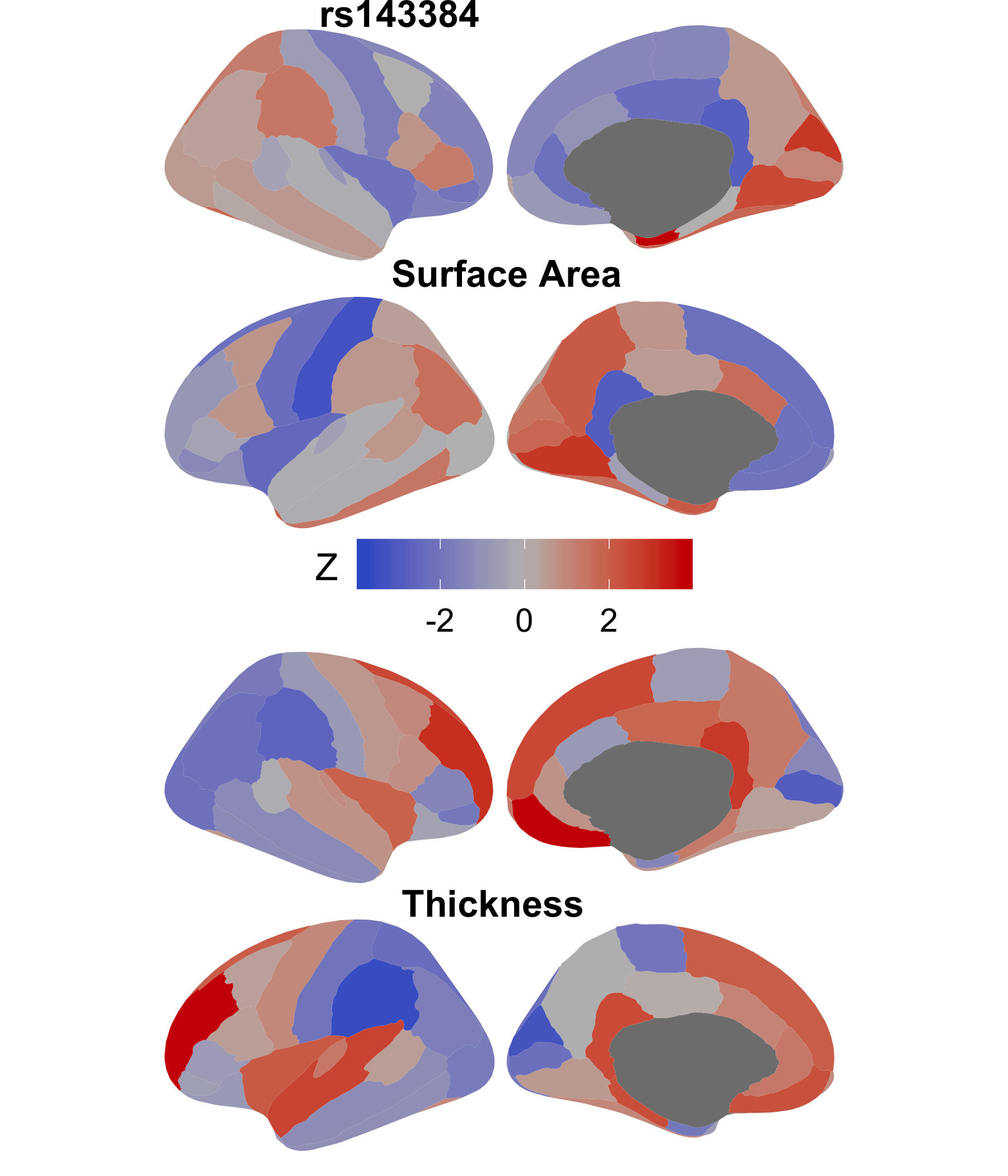

Supplement: Supplementary file 17 — Supplementary Data 14 [file 41467_2020_17368_MOESM17_ESM.gz › BrainMaps/most_aseg_vol/BrainMap098_rs143384.png]

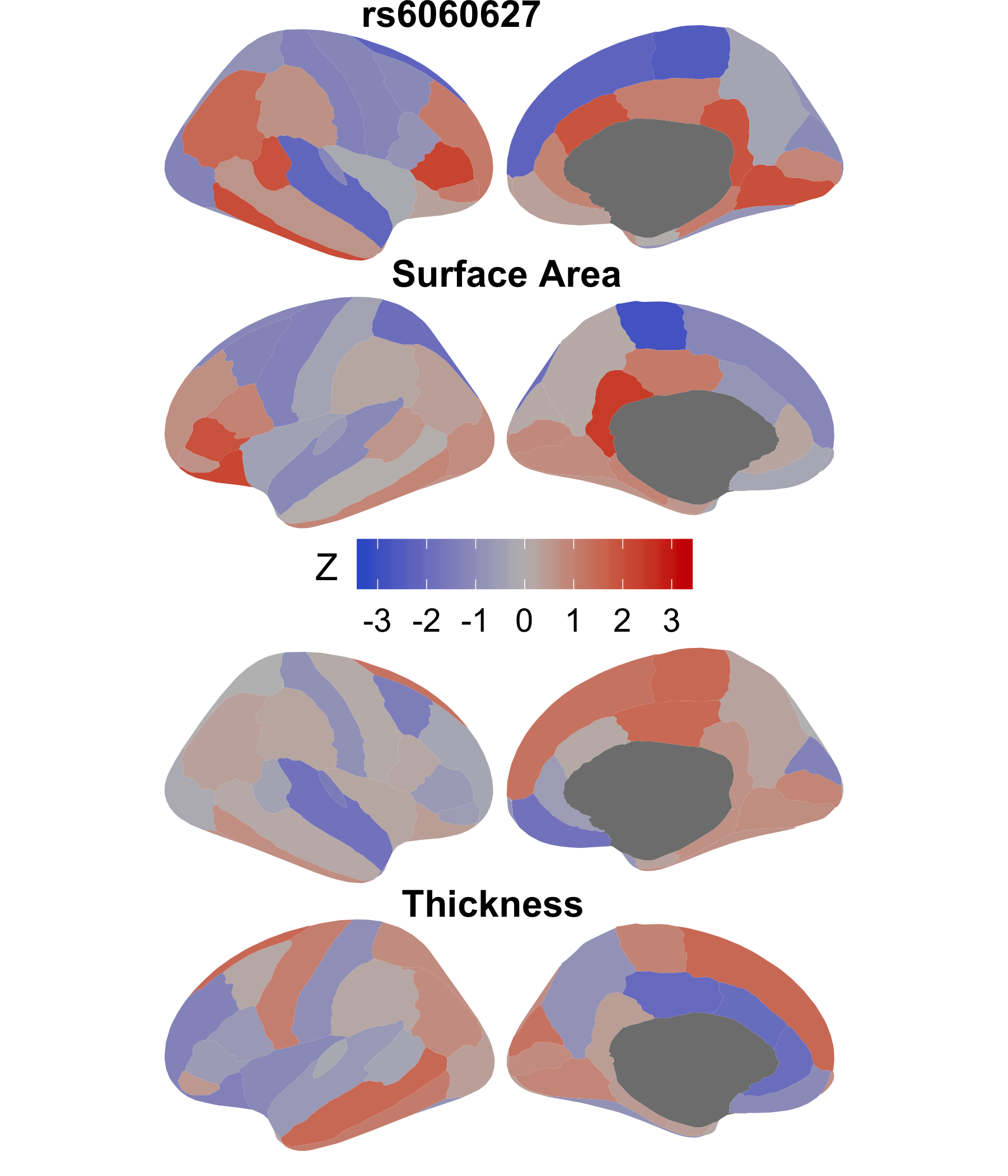

Supplement: Supplementary file 17 — Supplementary Data 14 [file 41467_2020_17368_MOESM17_ESM.gz › BrainMaps/most_aseg_vol/BrainMap018_rs6060627.png]

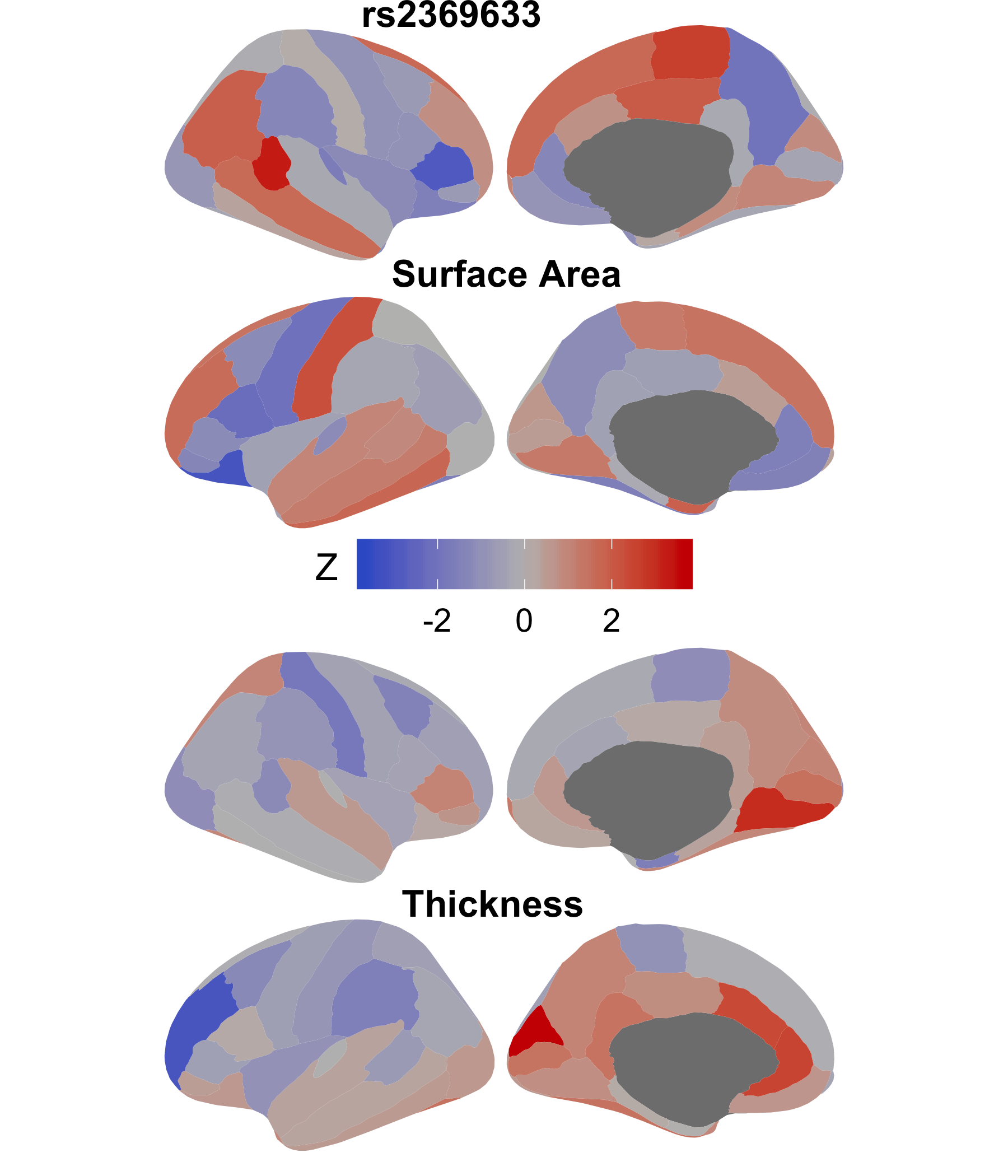

Supplement: Supplementary file 17 — Supplementary Data 14 [file 41467_2020_17368_MOESM17_ESM.gz › BrainMaps/most_aseg_vol/BrainMap163_rs2369633.png]

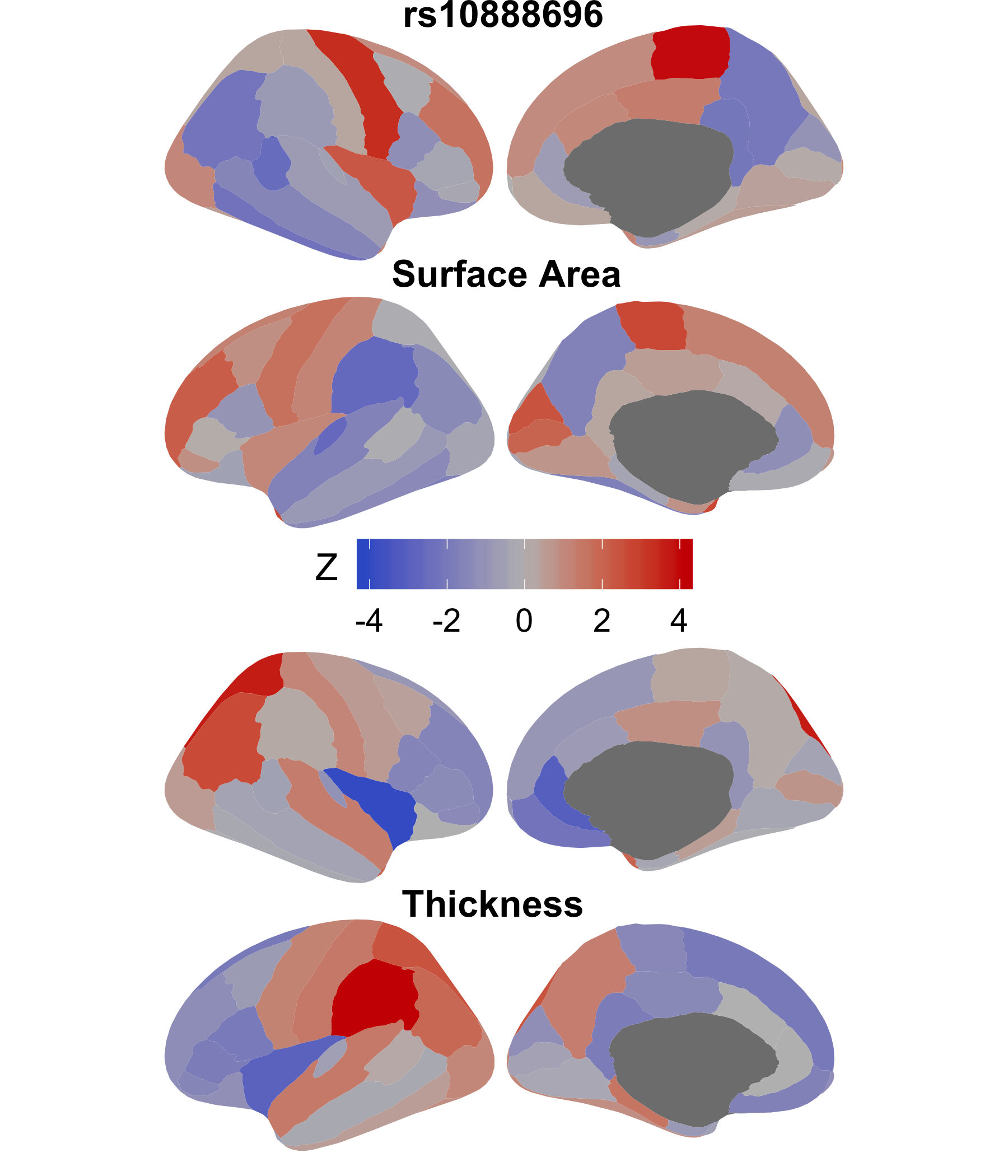

Supplement: Supplementary file 17 — Supplementary Data 14 [file 41467_2020_17368_MOESM17_ESM.gz › BrainMaps/most_aseg_vol/BrainMap021_rs10888696.png]

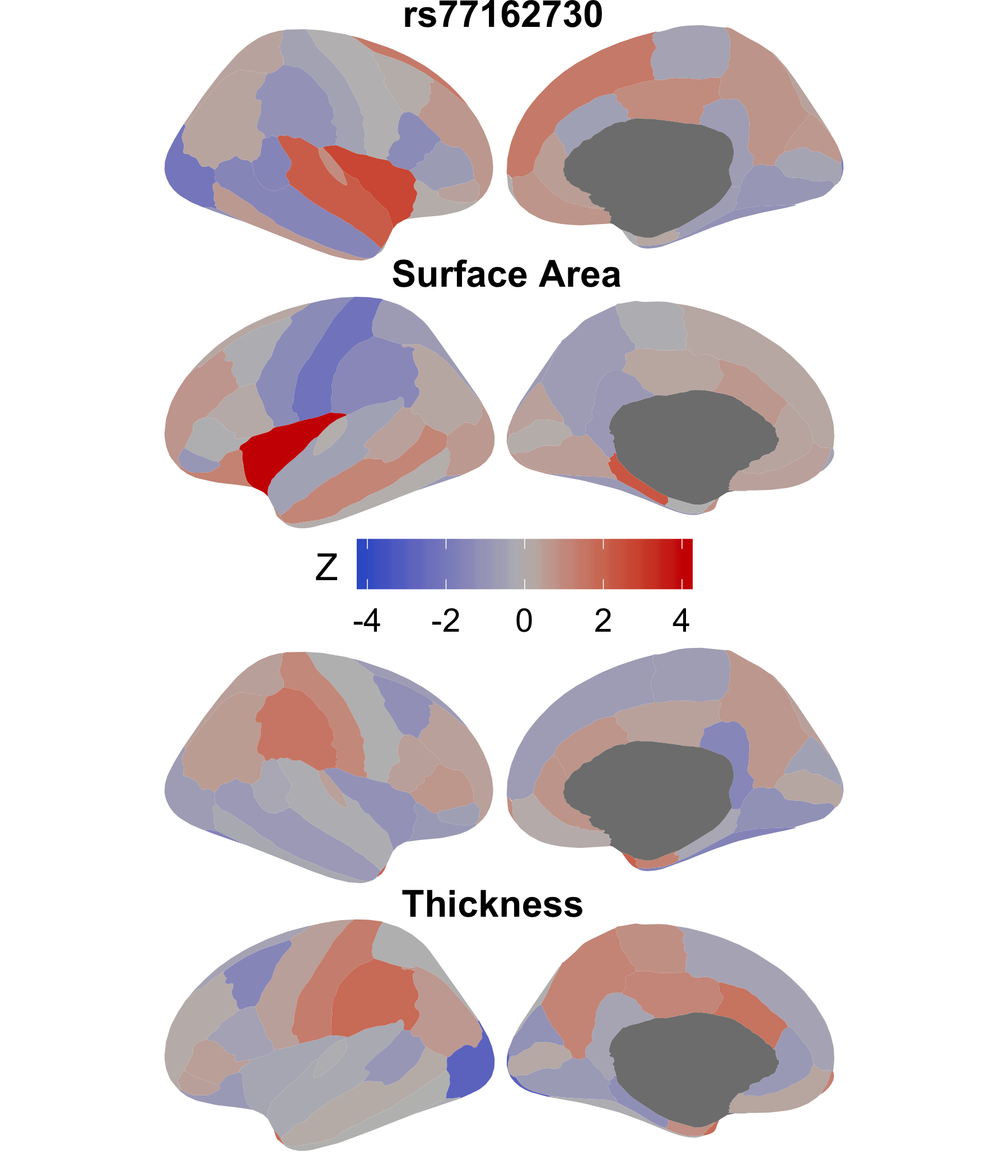

Supplement: Supplementary file 17 — Supplementary Data 14 [file 41467_2020_17368_MOESM17_ESM.gz › BrainMaps/most_aseg_vol/BrainMap015_rs77162730.png]

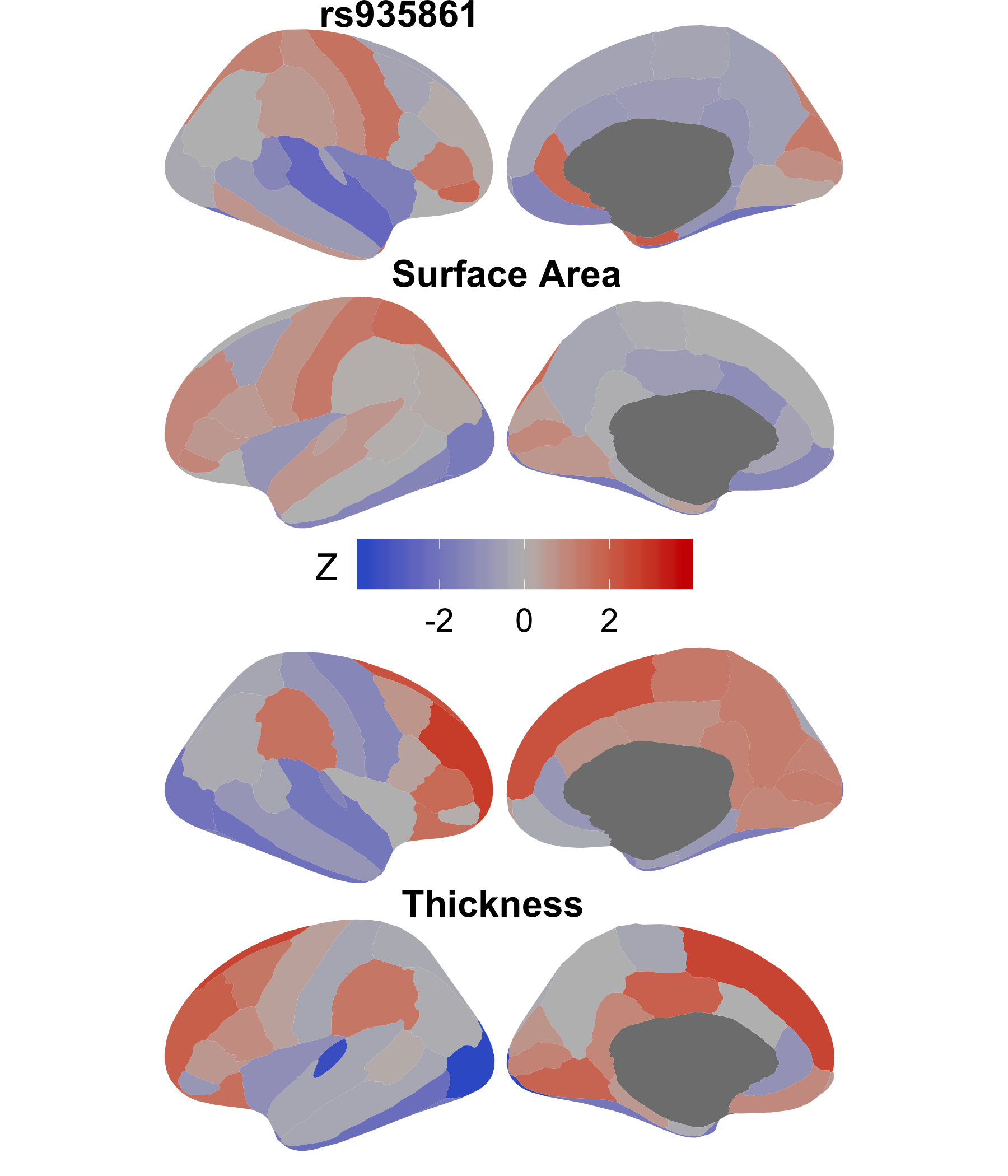

Supplement: Supplementary file 17 — Supplementary Data 14 [file 41467_2020_17368_MOESM17_ESM.gz › BrainMaps/most_aseg_vol/BrainMap102_rs935861.png]

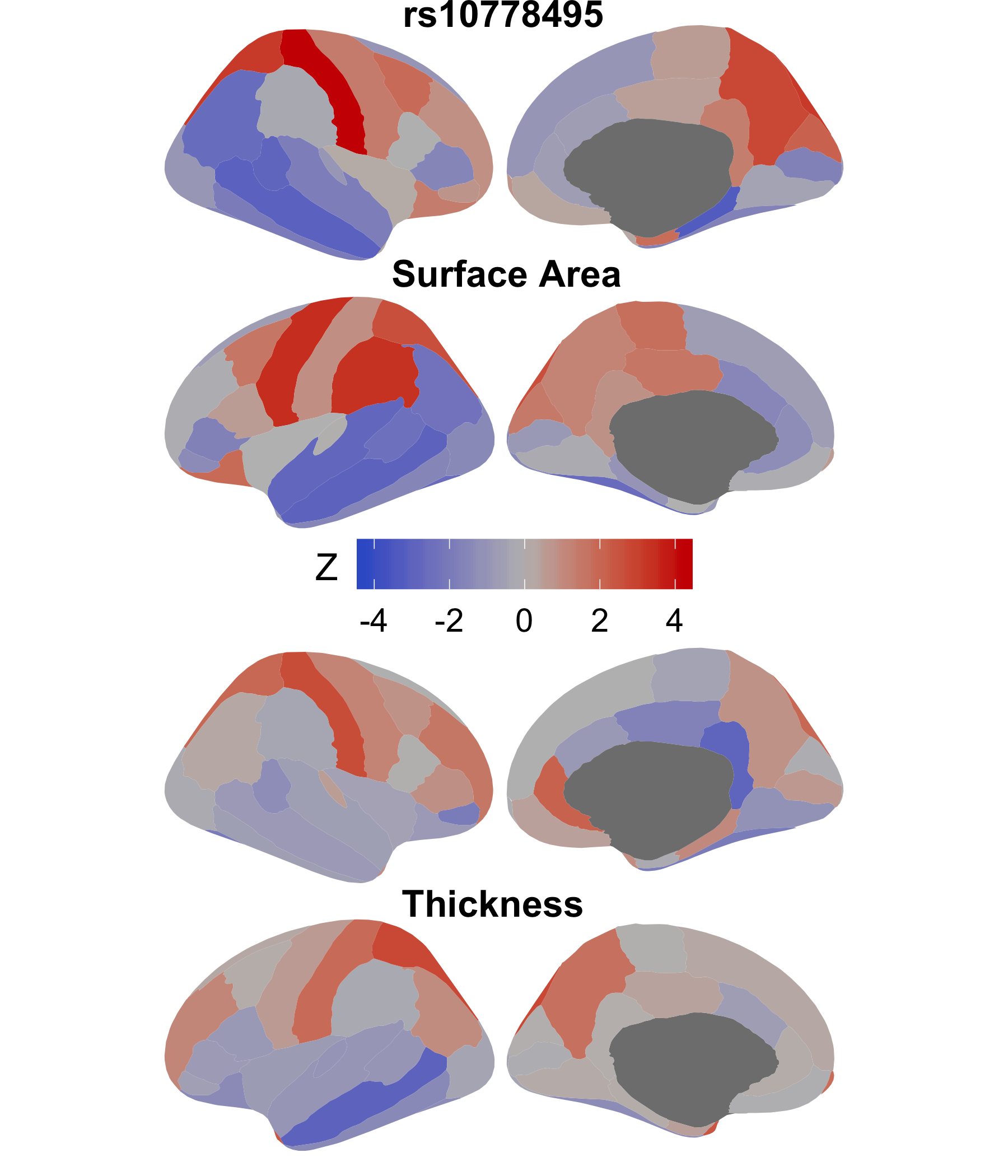

Supplement: Supplementary file 17 — Supplementary Data 14 [file 41467_2020_17368_MOESM17_ESM.gz › BrainMaps/most_aseg_vol/BrainMap036_rs10778495.png]

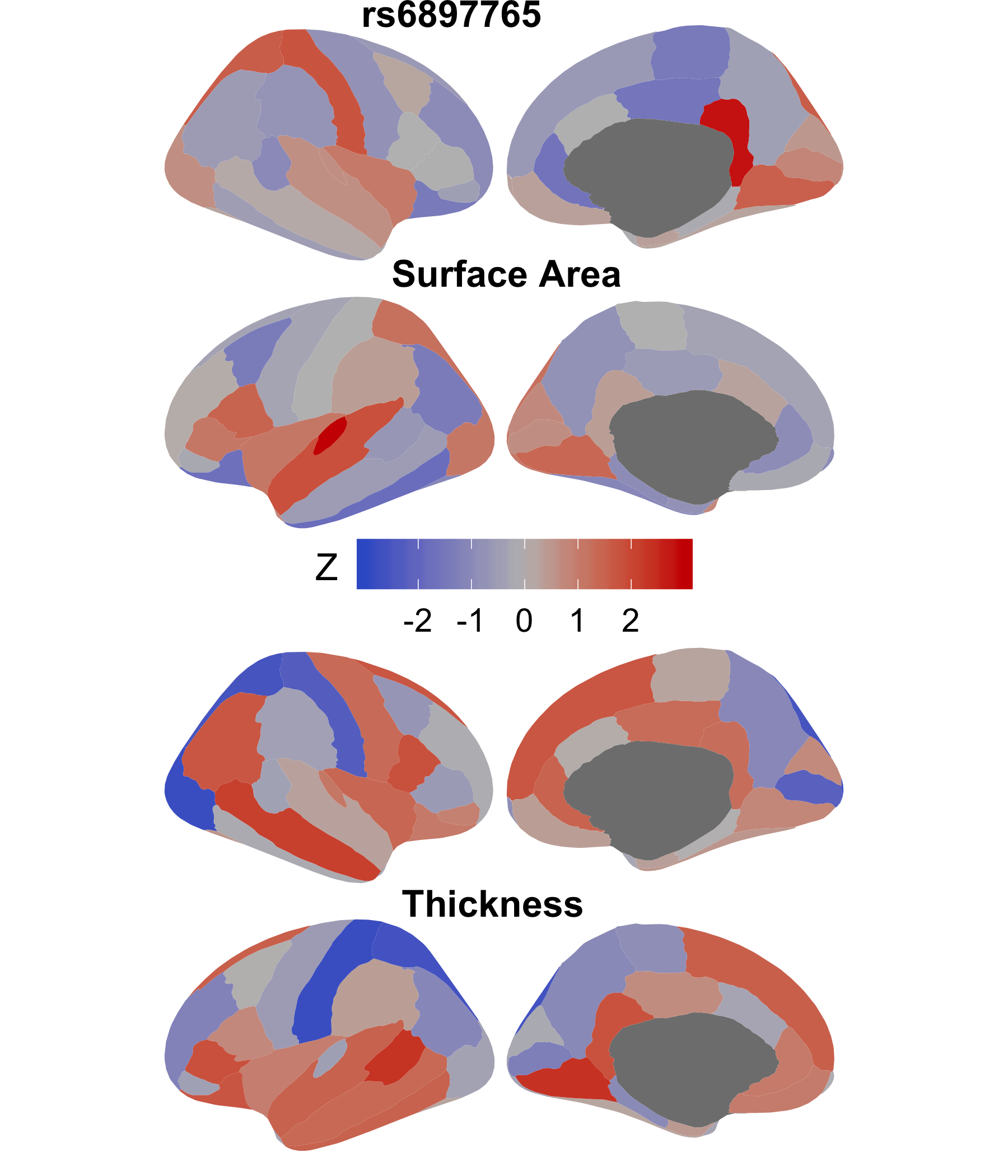

Supplement: Supplementary file 17 — Supplementary Data 14 [file 41467_2020_17368_MOESM17_ESM.gz › BrainMaps/most_aseg_vol/BrainMap032_rs6897765.png]

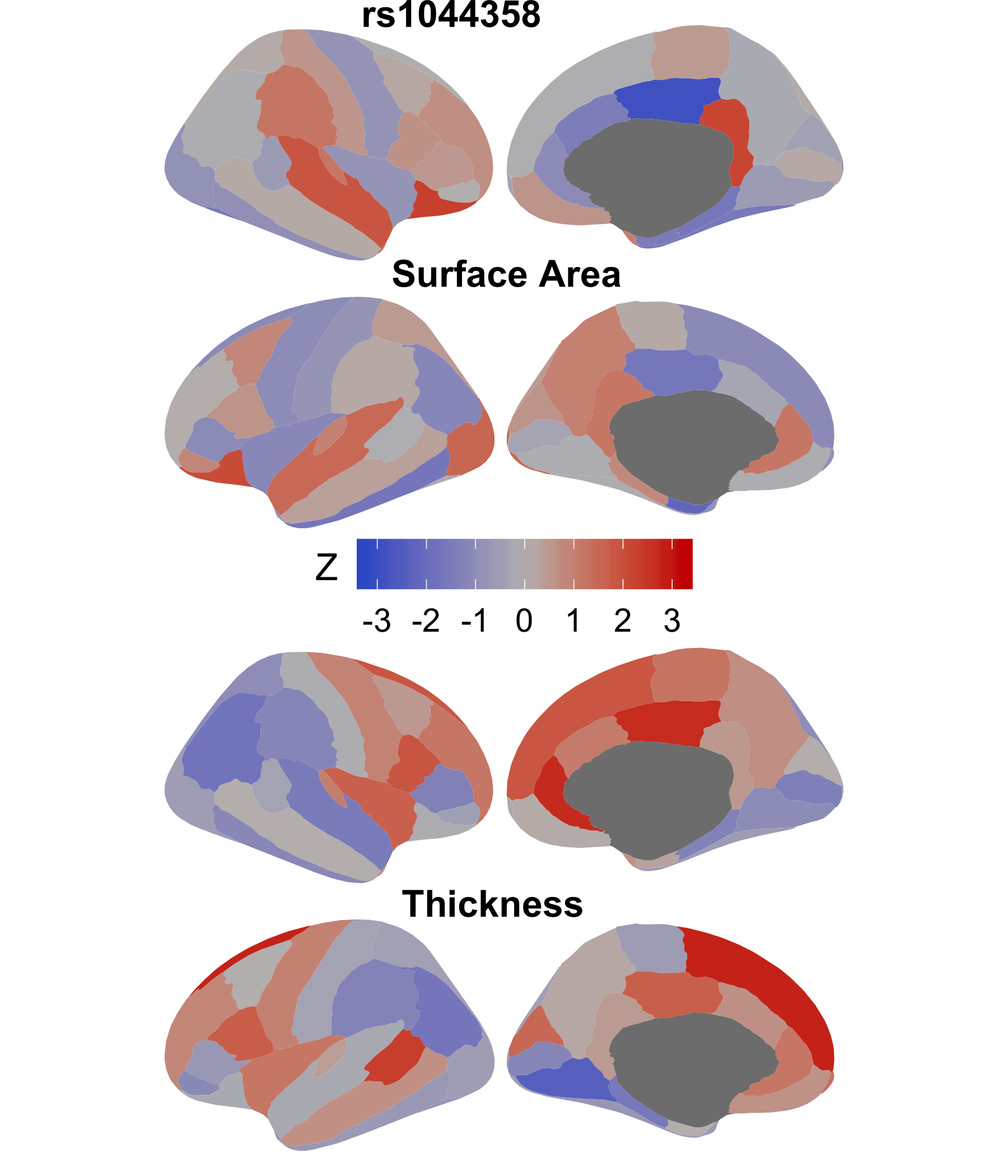

Supplement: Supplementary file 17 — Supplementary Data 14 [file 41467_2020_17368_MOESM17_ESM.gz › BrainMaps/most_aseg_vol/BrainMap134_rs1044358.png]

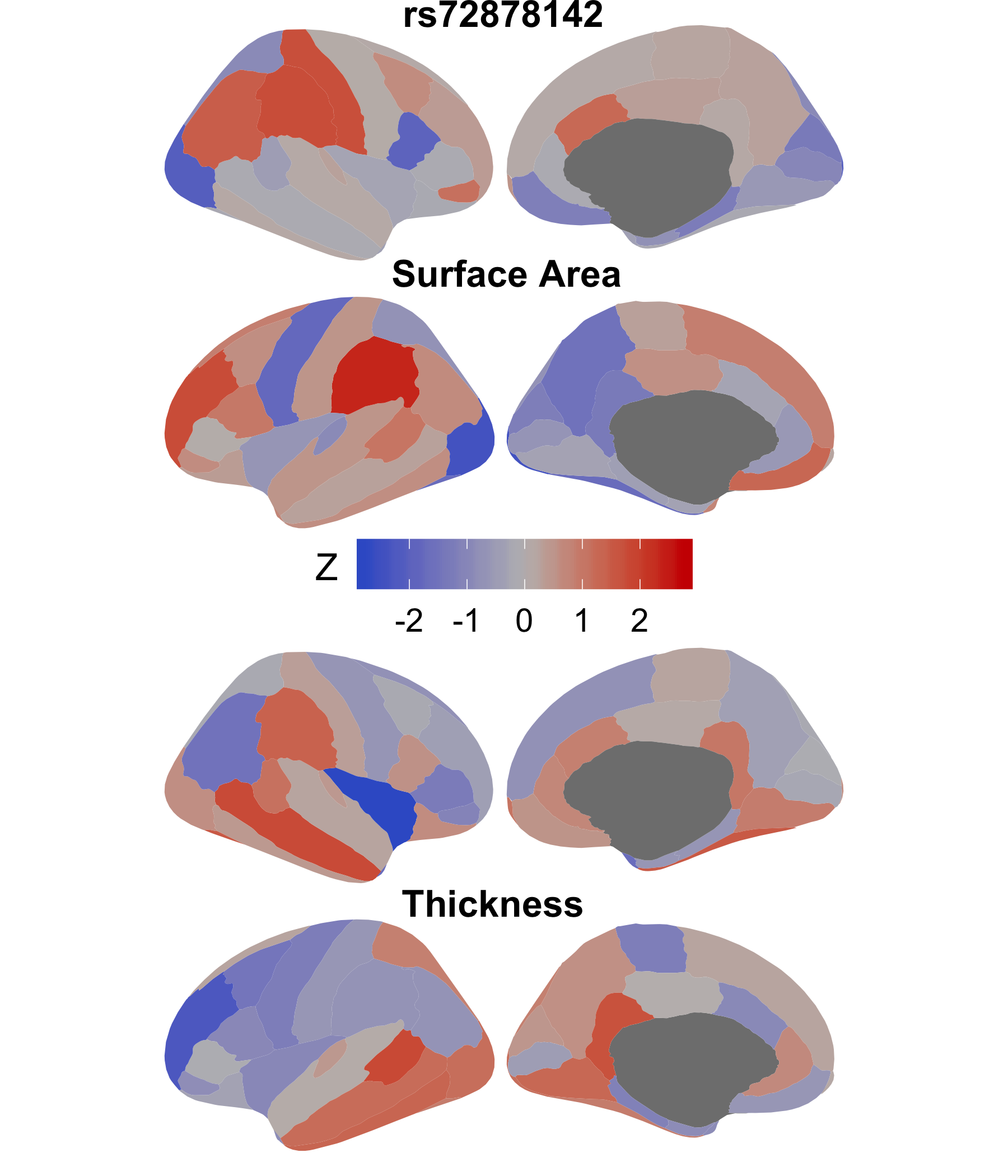

Supplement: Supplementary file 17 — Supplementary Data 14 [file 41467_2020_17368_MOESM17_ESM.gz › BrainMaps/most_aseg_vol/BrainMap090_rs72878142.png]

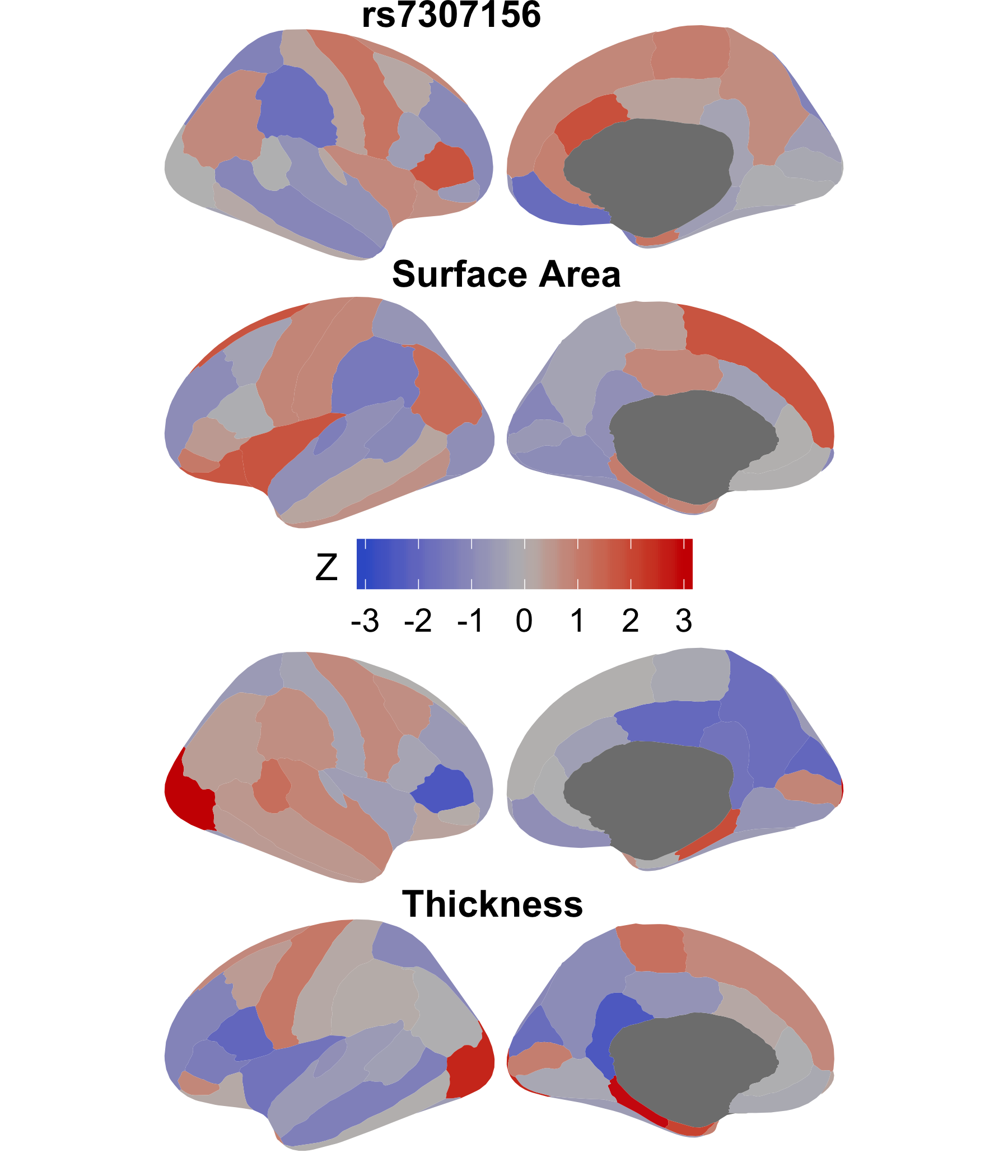

Supplement: Supplementary file 17 — Supplementary Data 14 [file 41467_2020_17368_MOESM17_ESM.gz › BrainMaps/most_aseg_vol/BrainMap109_rs7307156.png]

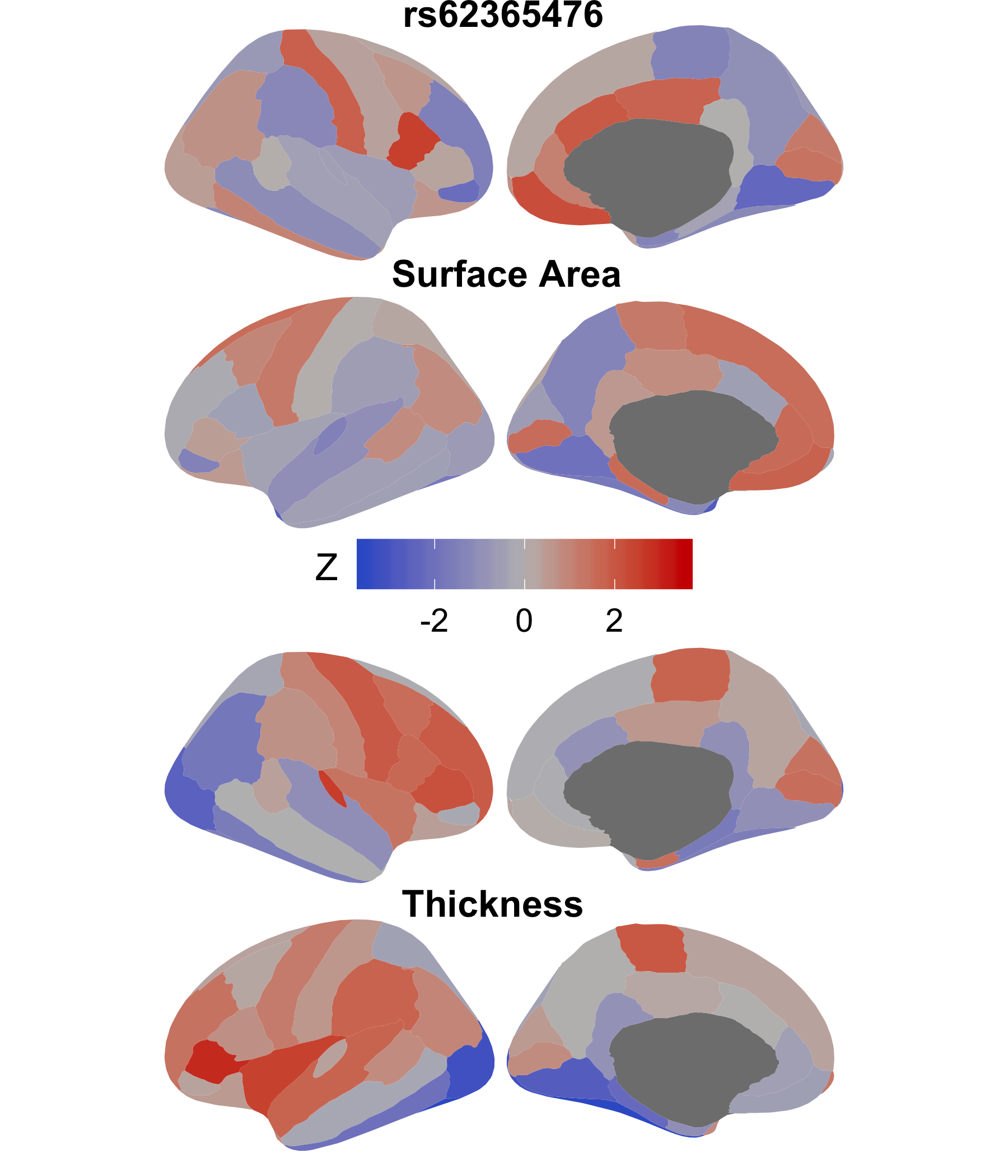

Supplement: Supplementary file 17 — Supplementary Data 14 [file 41467_2020_17368_MOESM17_ESM.gz › BrainMaps/most_aseg_vol/BrainMap058_rs62365476.png]

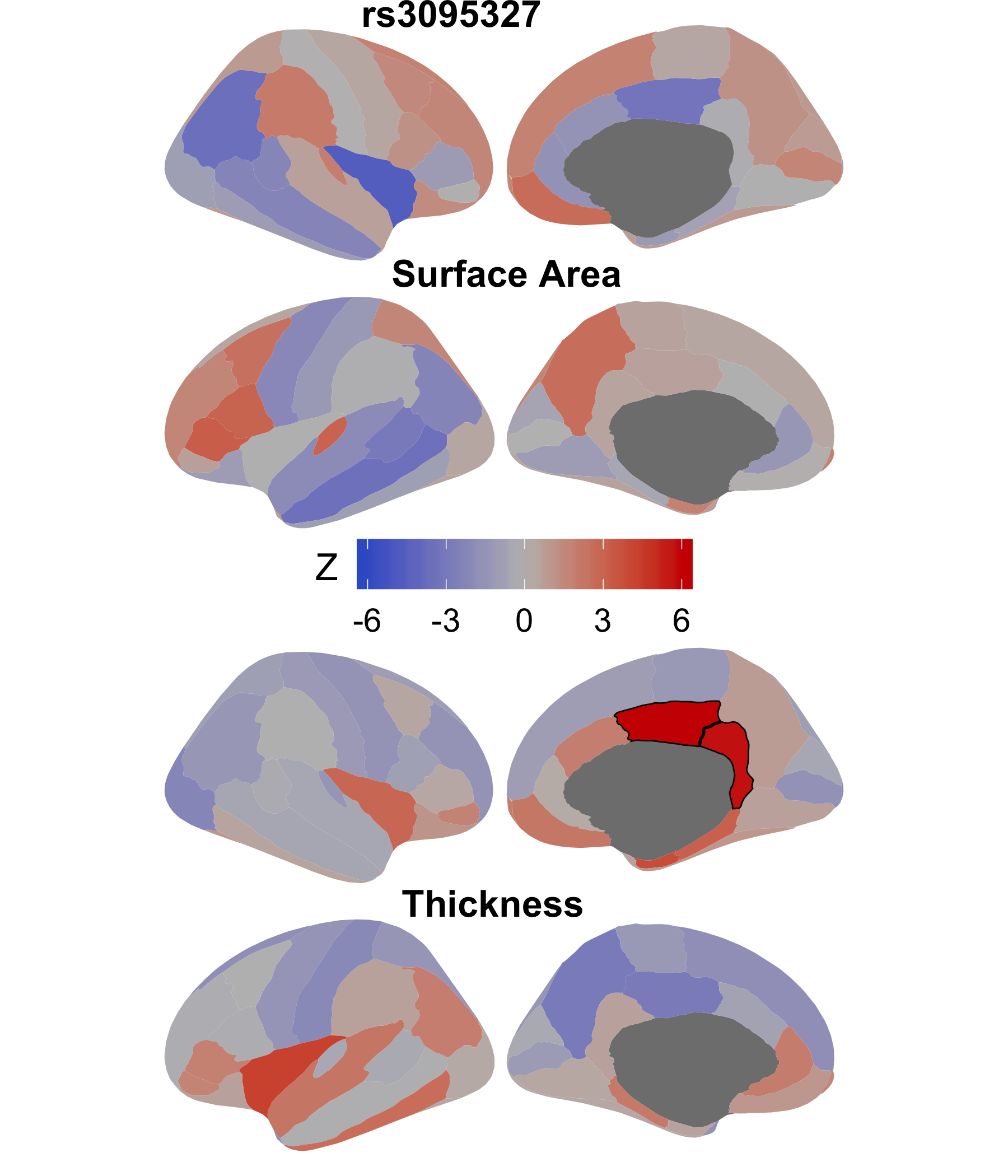

Supplement: Supplementary file 17 — Supplementary Data 14 [file 41467_2020_17368_MOESM17_ESM.gz › BrainMaps/most_aseg_vol/BrainMap047_rs3095327.png]

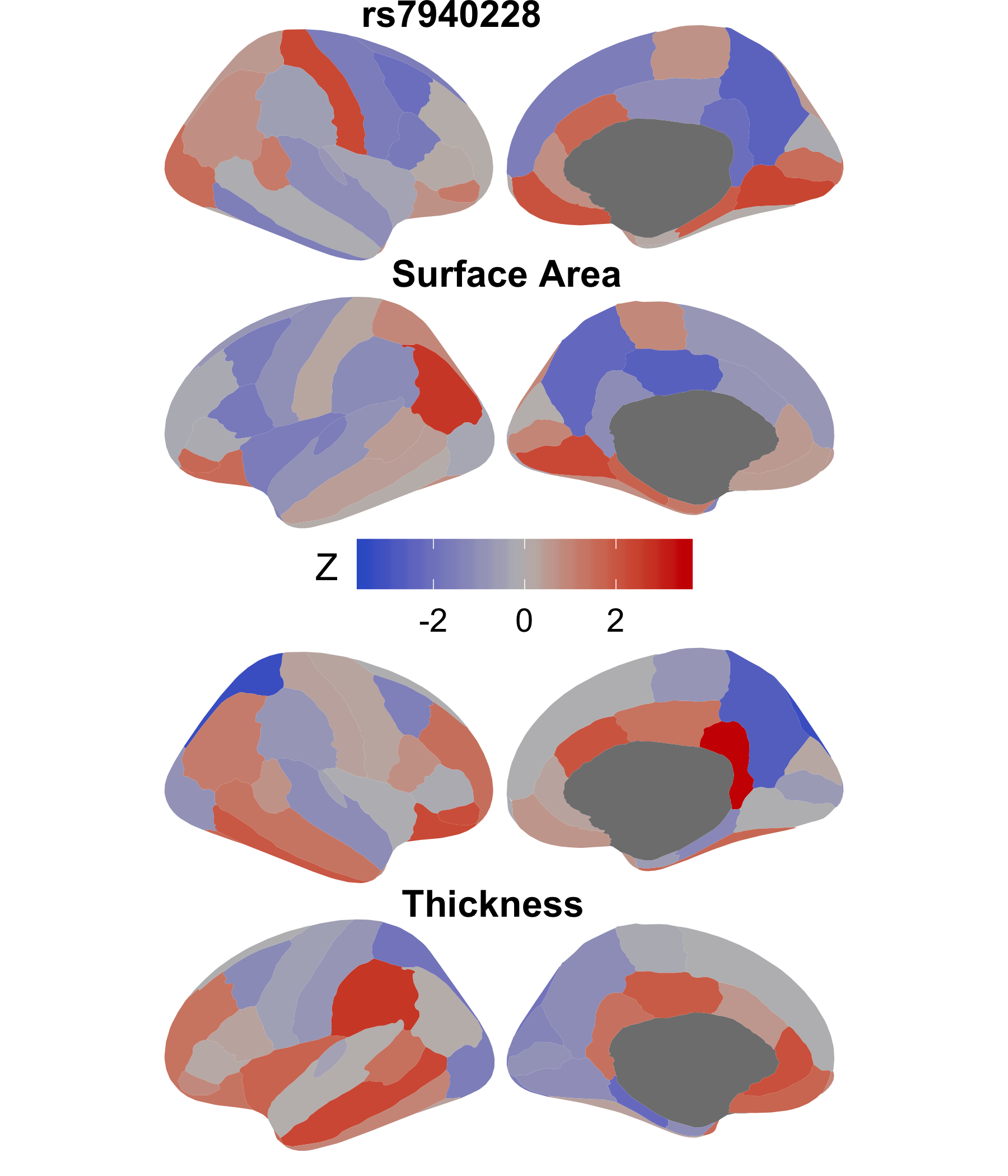

Supplement: Supplementary file 17 — Supplementary Data 14 [file 41467_2020_17368_MOESM17_ESM.gz › BrainMaps/most_aseg_vol/BrainMap121_rs7940228.png]

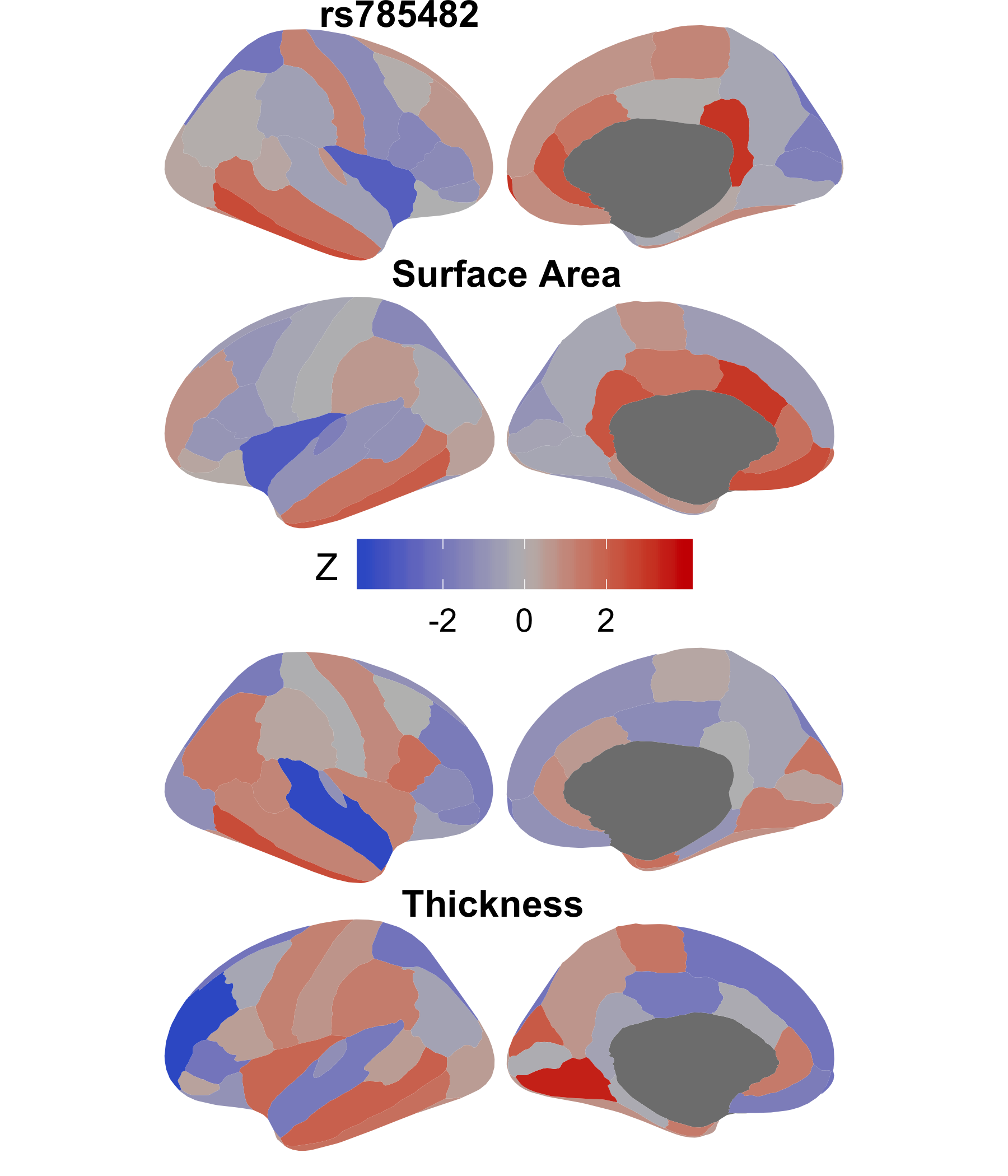

Supplement: Supplementary file 17 — Supplementary Data 14 [file 41467_2020_17368_MOESM17_ESM.gz › BrainMaps/most_aseg_vol/BrainMap159_rs785482.png]

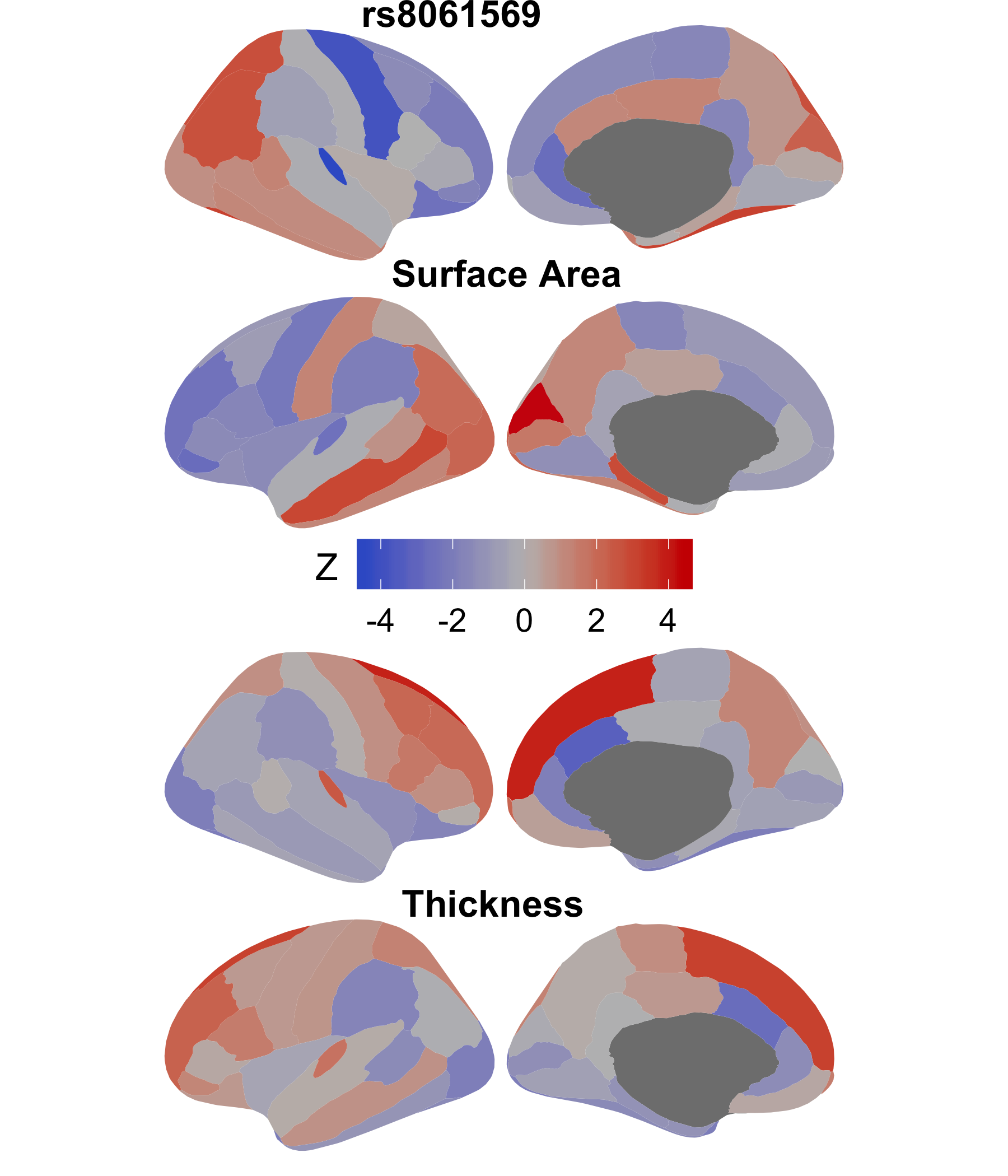

Supplement: Supplementary file 17 — Supplementary Data 14 [file 41467_2020_17368_MOESM17_ESM.gz › BrainMaps/most_aseg_vol/BrainMap006_rs8061569.png]

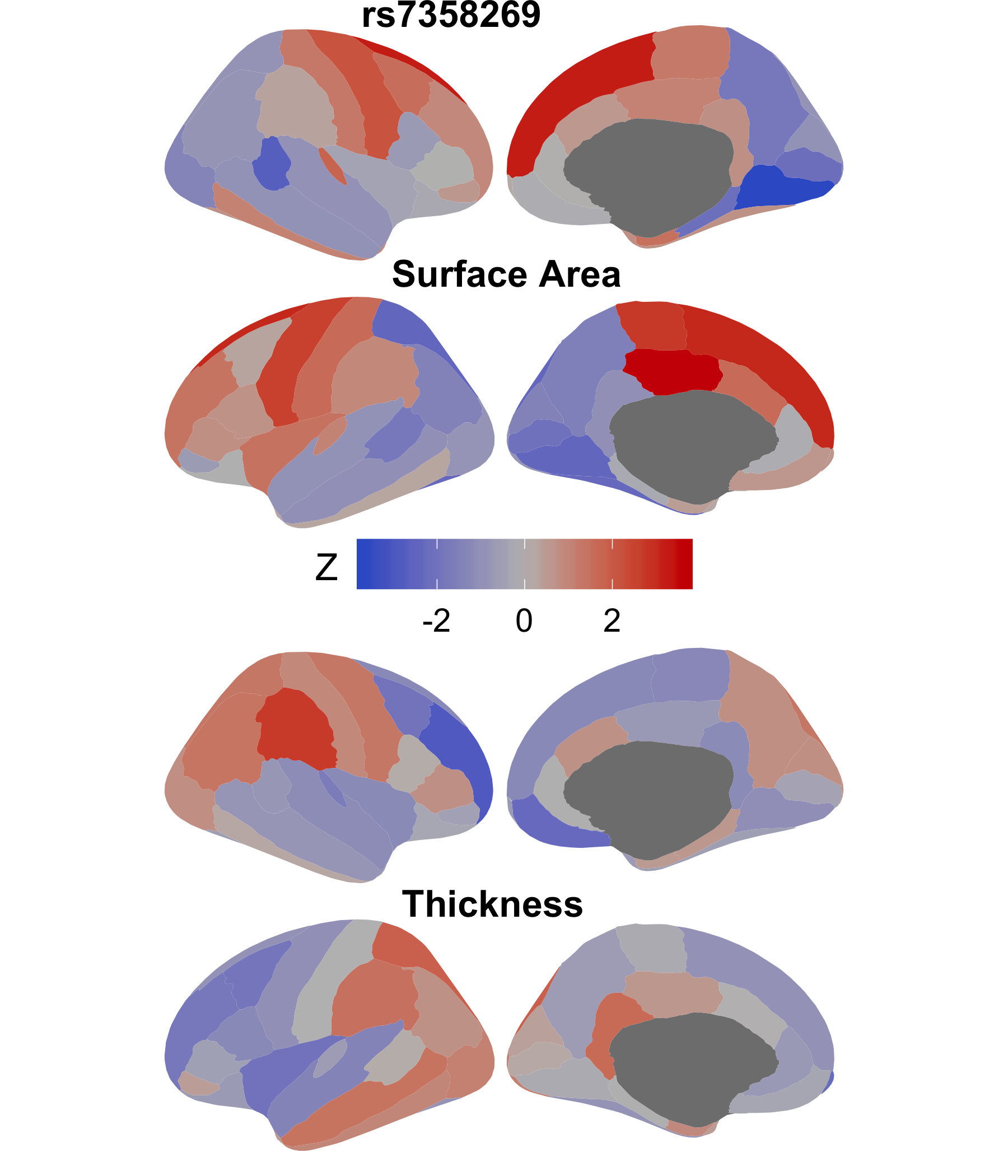

Supplement: Supplementary file 17 — Supplementary Data 14 [file 41467_2020_17368_MOESM17_ESM.gz › BrainMaps/most_aseg_vol/BrainMap060_rs7358269.png]

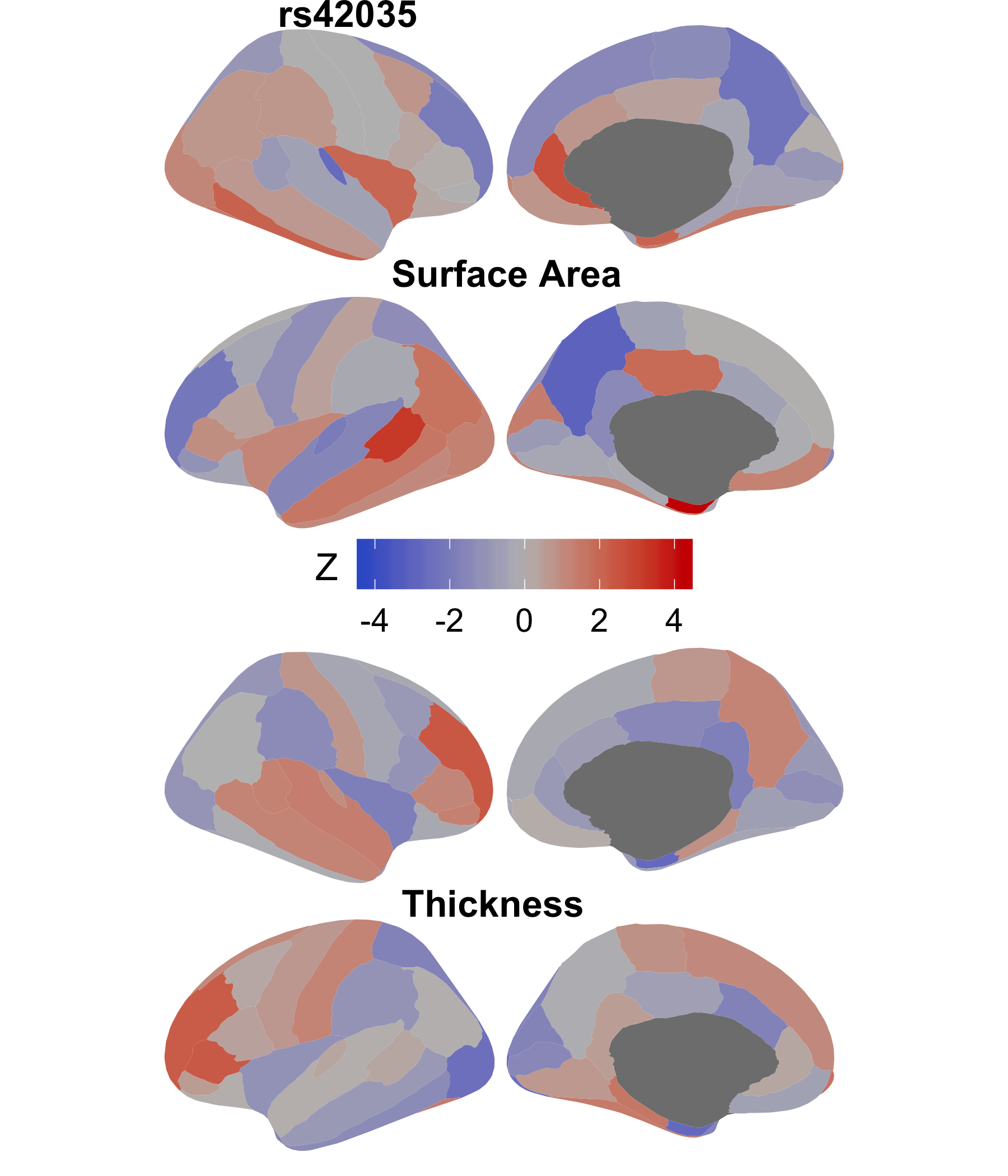

Supplement: Supplementary file 17 — Supplementary Data 14 [file 41467_2020_17368_MOESM17_ESM.gz › BrainMaps/most_aseg_vol/BrainMap093_rs42035.png]

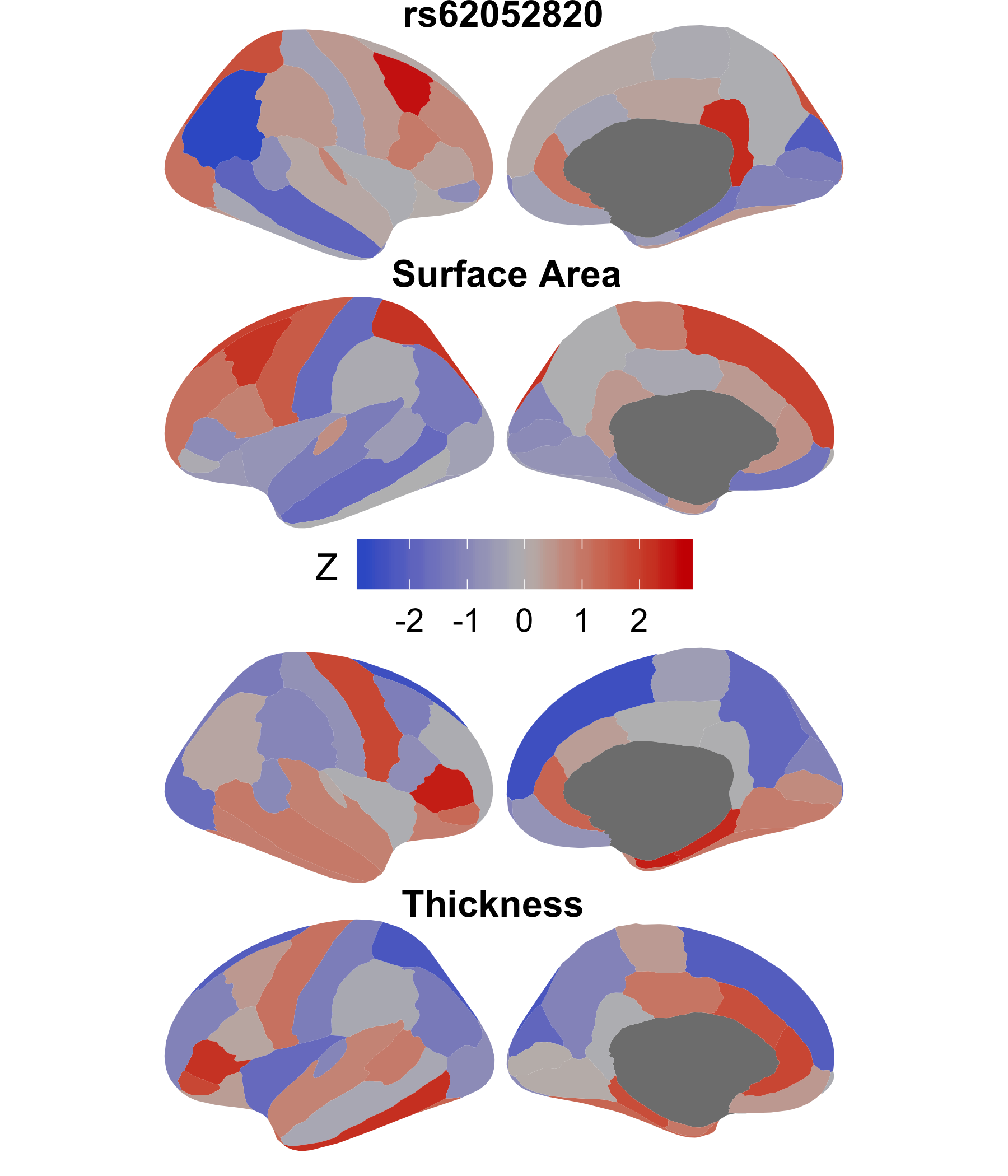

Supplement: Supplementary file 17 — Supplementary Data 14 [file 41467_2020_17368_MOESM17_ESM.gz › BrainMaps/most_aseg_vol/BrainMap158_rs62052820.png]

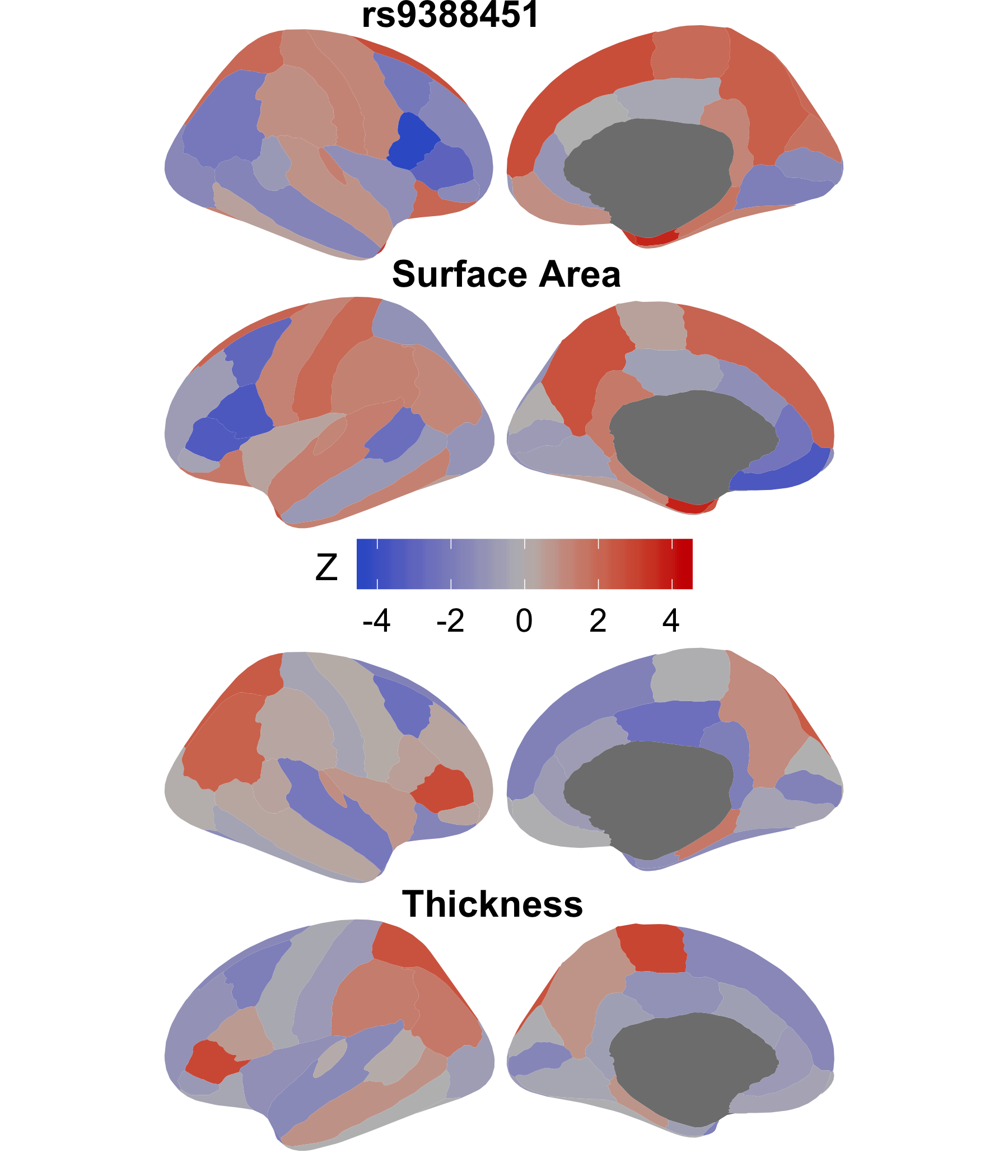

Supplement: Supplementary file 17 — Supplementary Data 14 [file 41467_2020_17368_MOESM17_ESM.gz › BrainMaps/most_aseg_vol/BrainMap130_rs9388451.png]

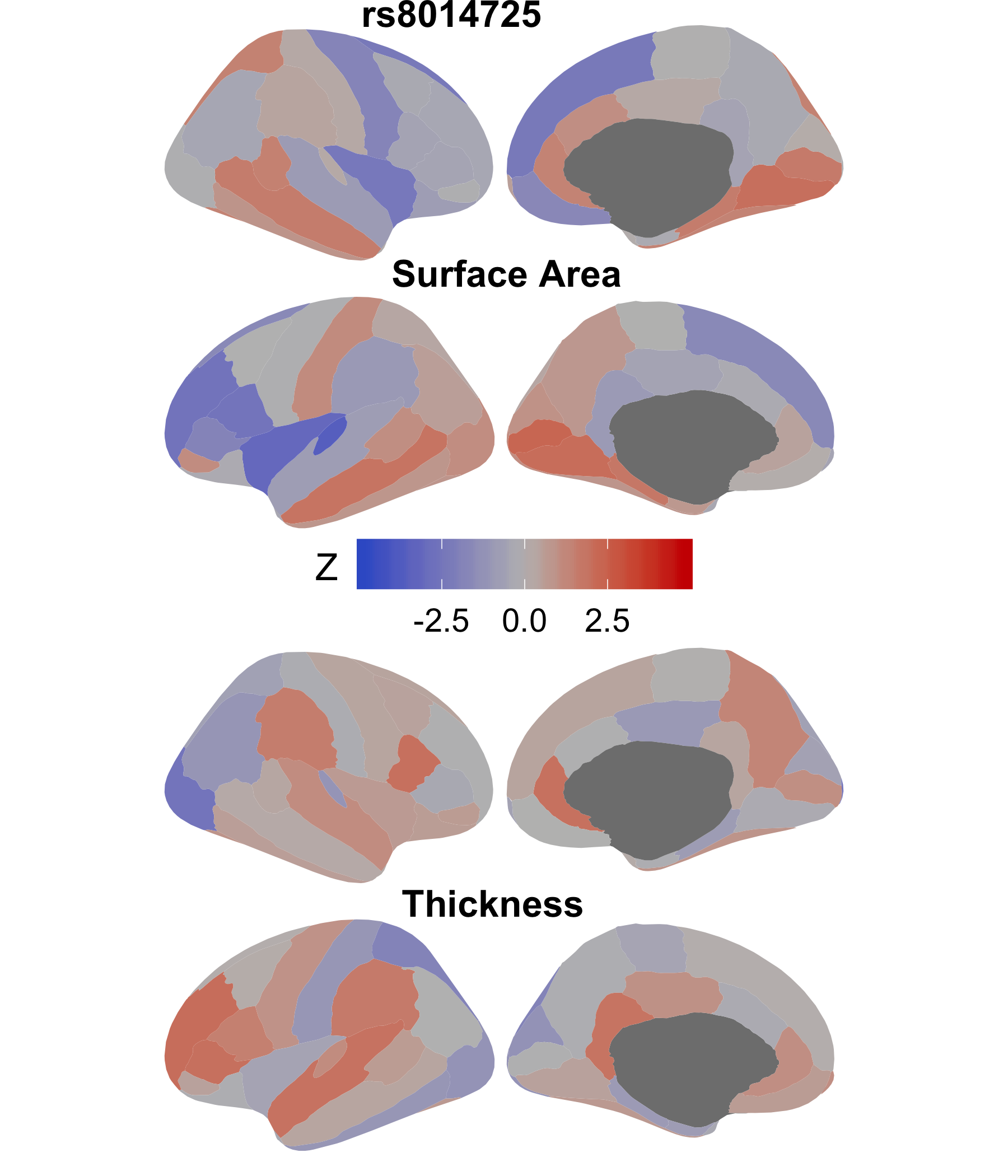

Supplement: Supplementary file 17 — Supplementary Data 14 [file 41467_2020_17368_MOESM17_ESM.gz › BrainMaps/most_aseg_vol/BrainMap011_rs8014725.png]

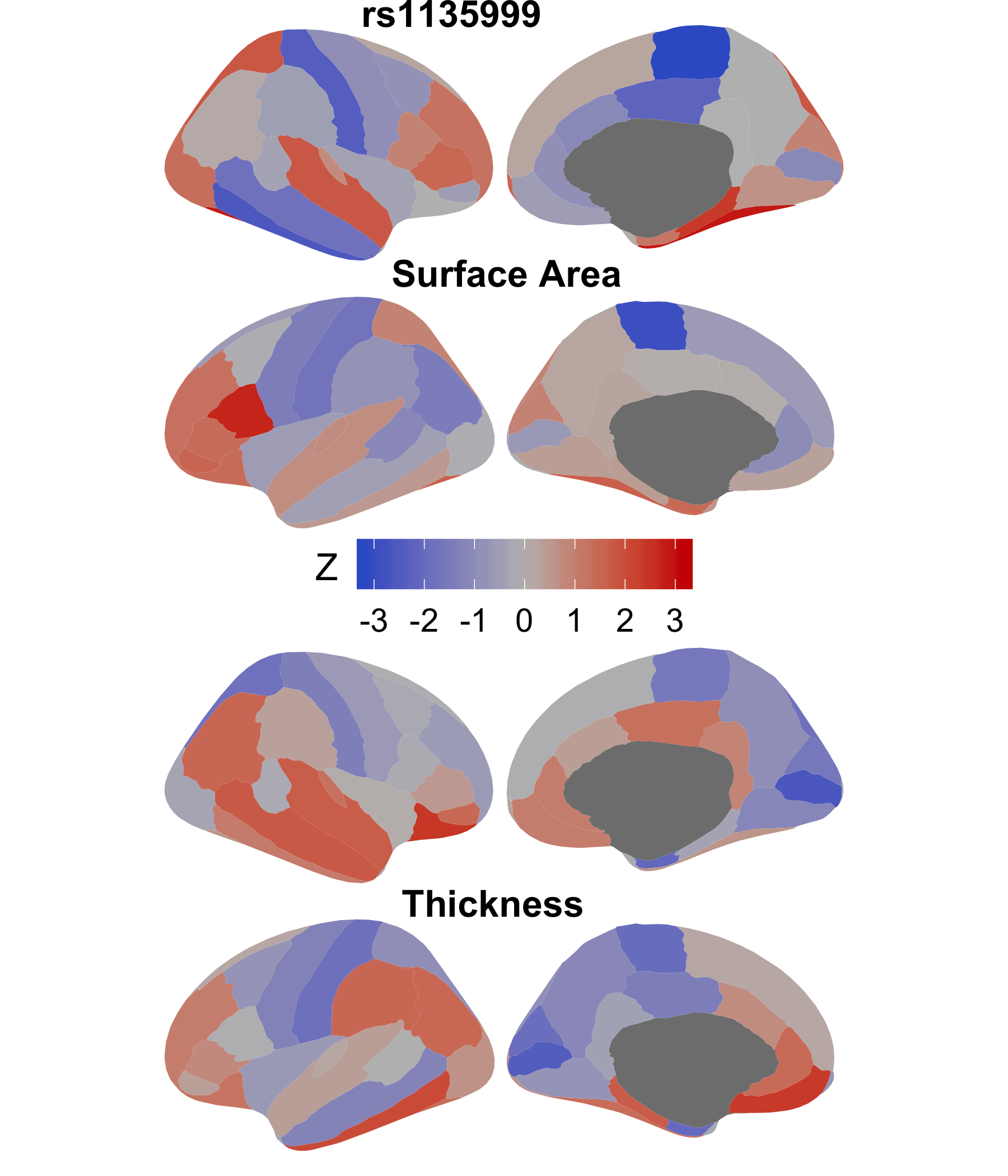

Supplement: Supplementary file 17 — Supplementary Data 14 [file 41467_2020_17368_MOESM17_ESM.gz › BrainMaps/most_aseg_vol/BrainMap125_rs1135999.png]

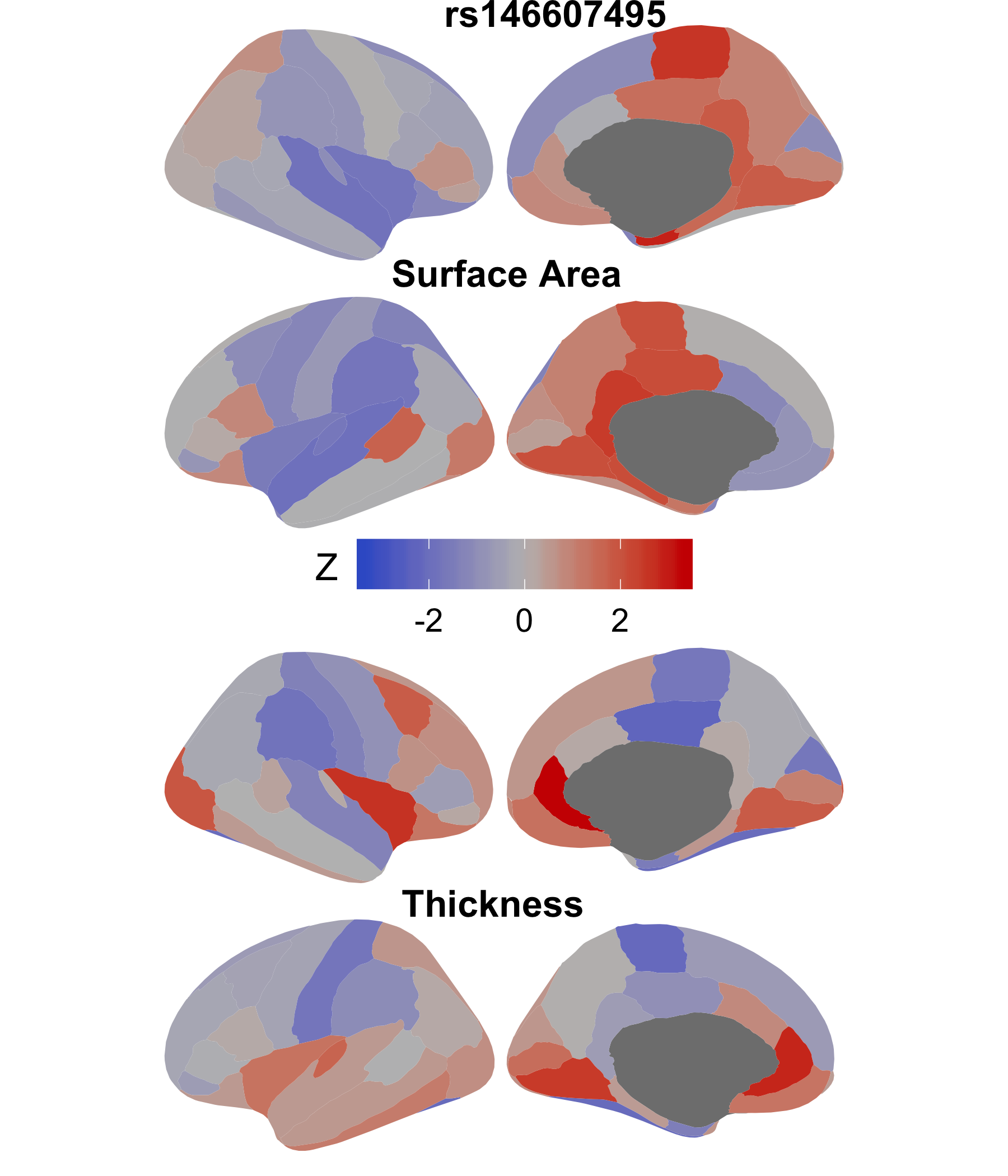

Supplement: Supplementary file 17 — Supplementary Data 14 [file 41467_2020_17368_MOESM17_ESM.gz › BrainMaps/most_aseg_vol/BrainMap007_rs146607495.png]

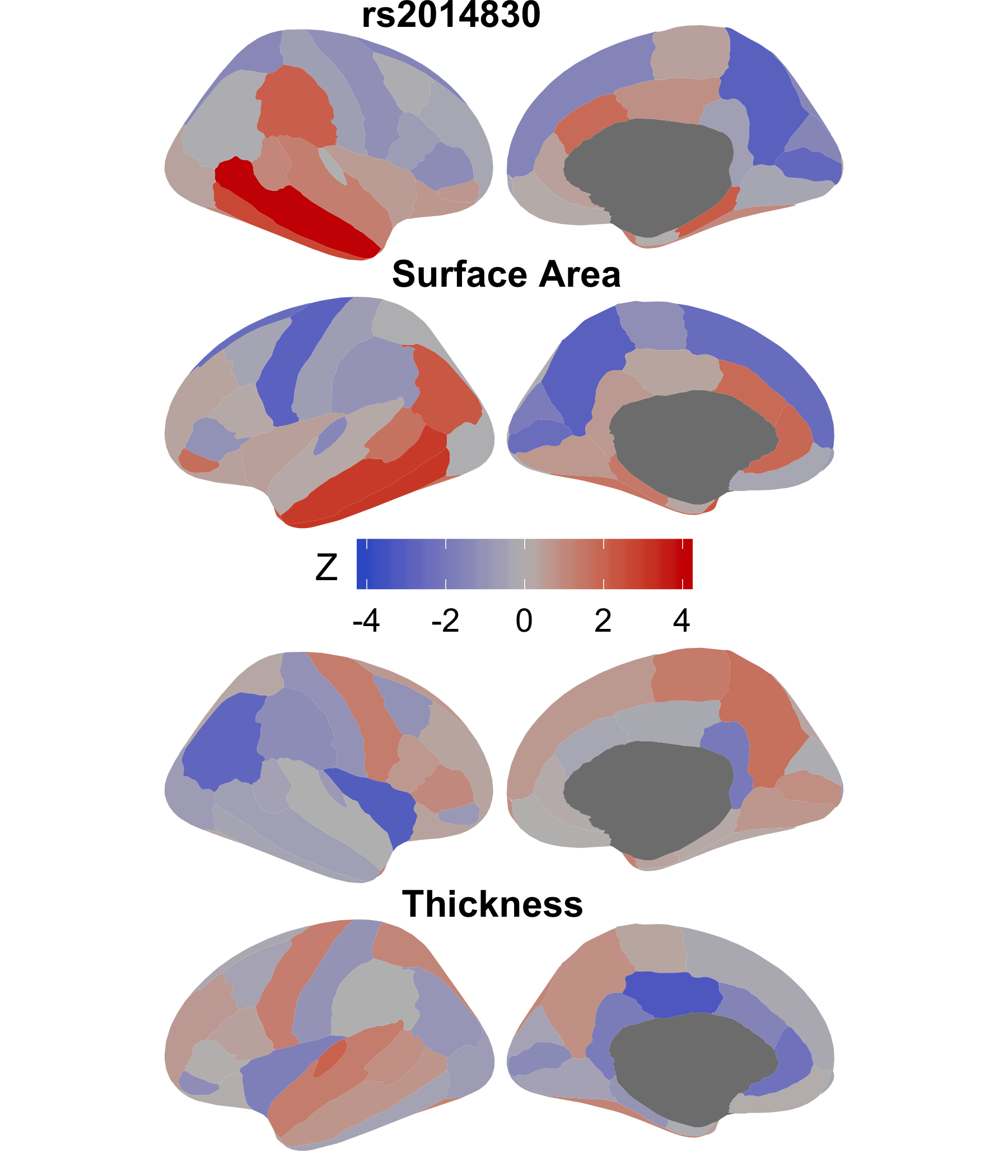

Supplement: Supplementary file 17 — Supplementary Data 14 [file 41467_2020_17368_MOESM17_ESM.gz › BrainMaps/most_aseg_vol/BrainMap067_rs2014830.png]

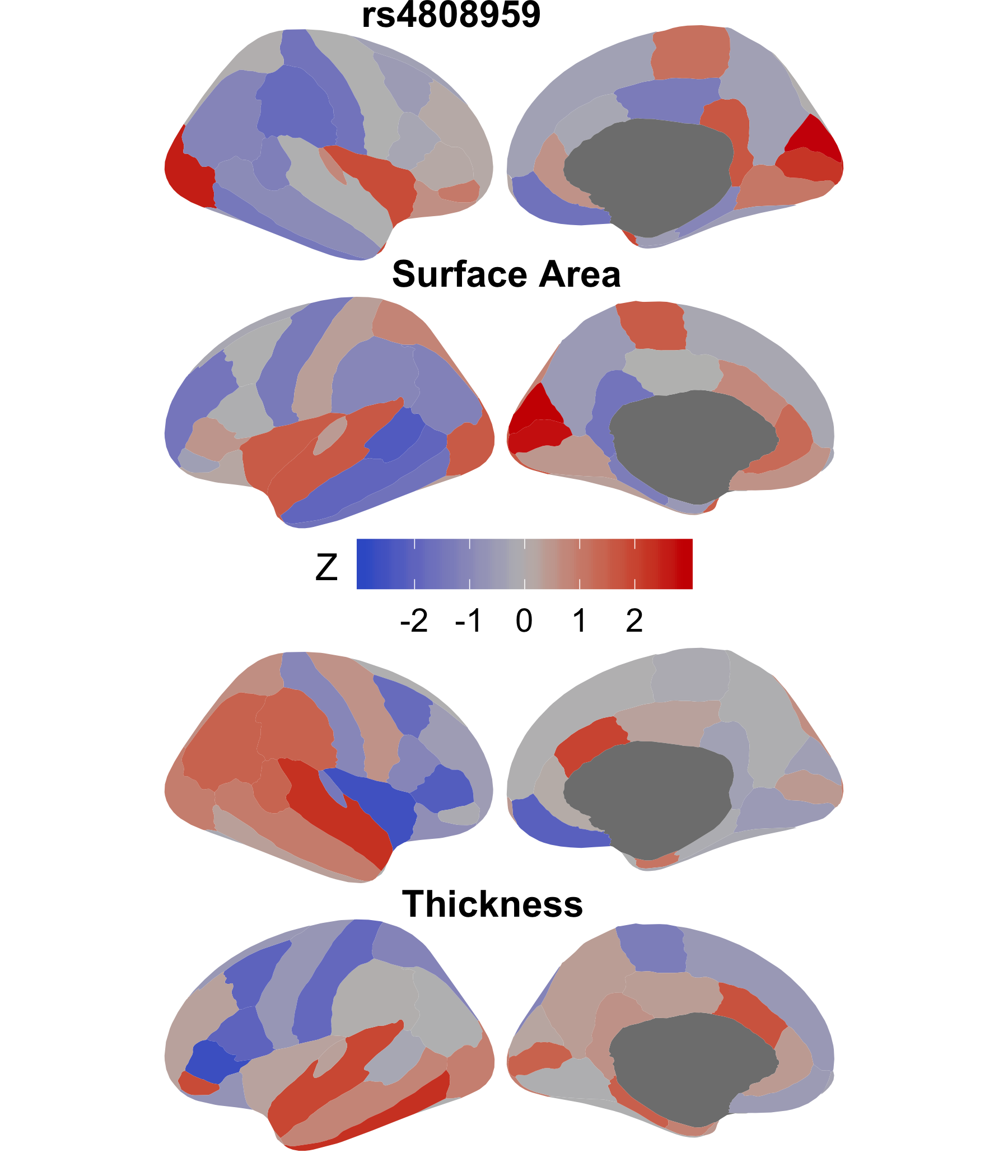

Supplement: Supplementary file 17 — Supplementary Data 14 [file 41467_2020_17368_MOESM17_ESM.gz › BrainMaps/most_aseg_vol/BrainMap139_rs4808959.png]

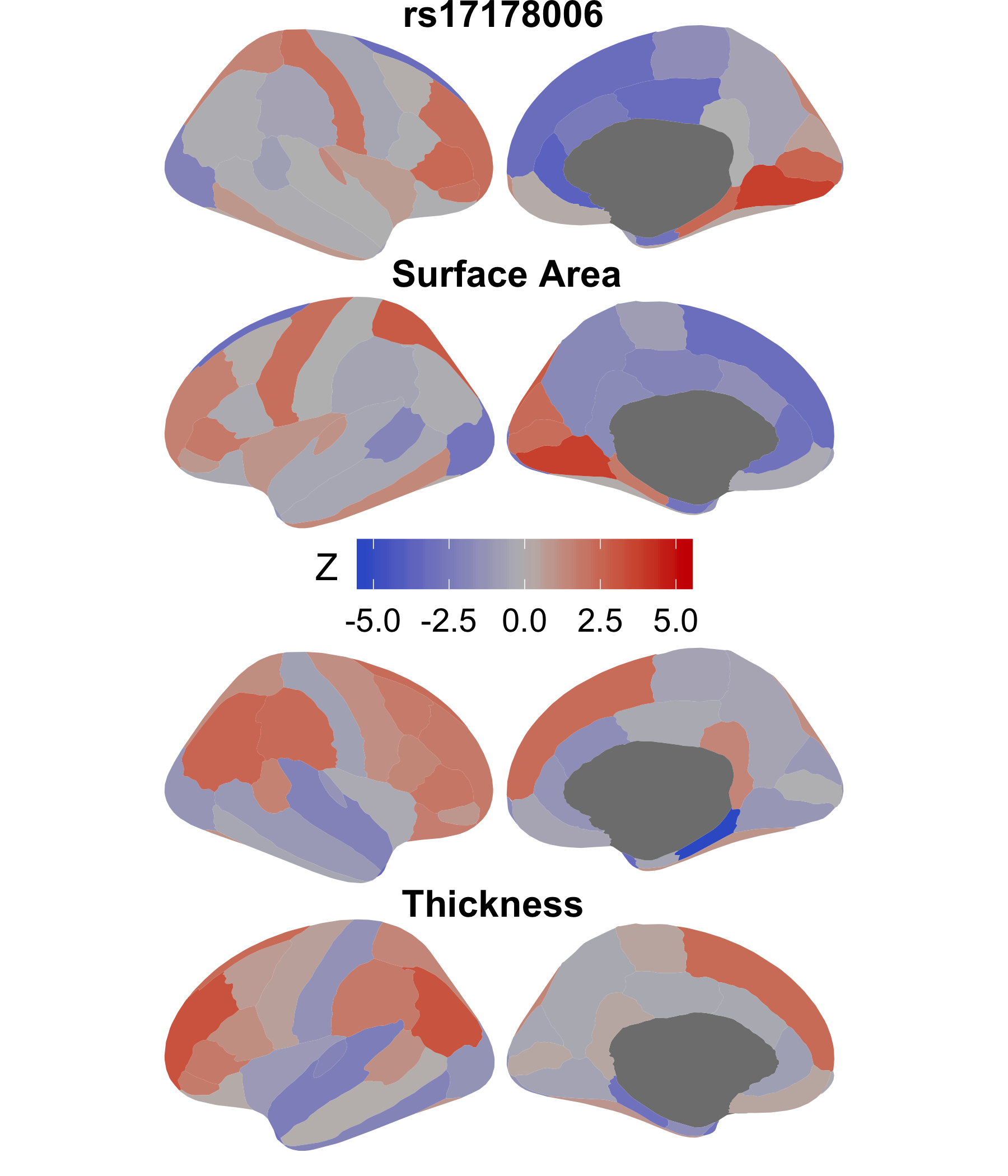

Supplement: Supplementary file 17 — Supplementary Data 14 [file 41467_2020_17368_MOESM17_ESM.gz › BrainMaps/most_aseg_vol/BrainMap009_rs17178006.png]

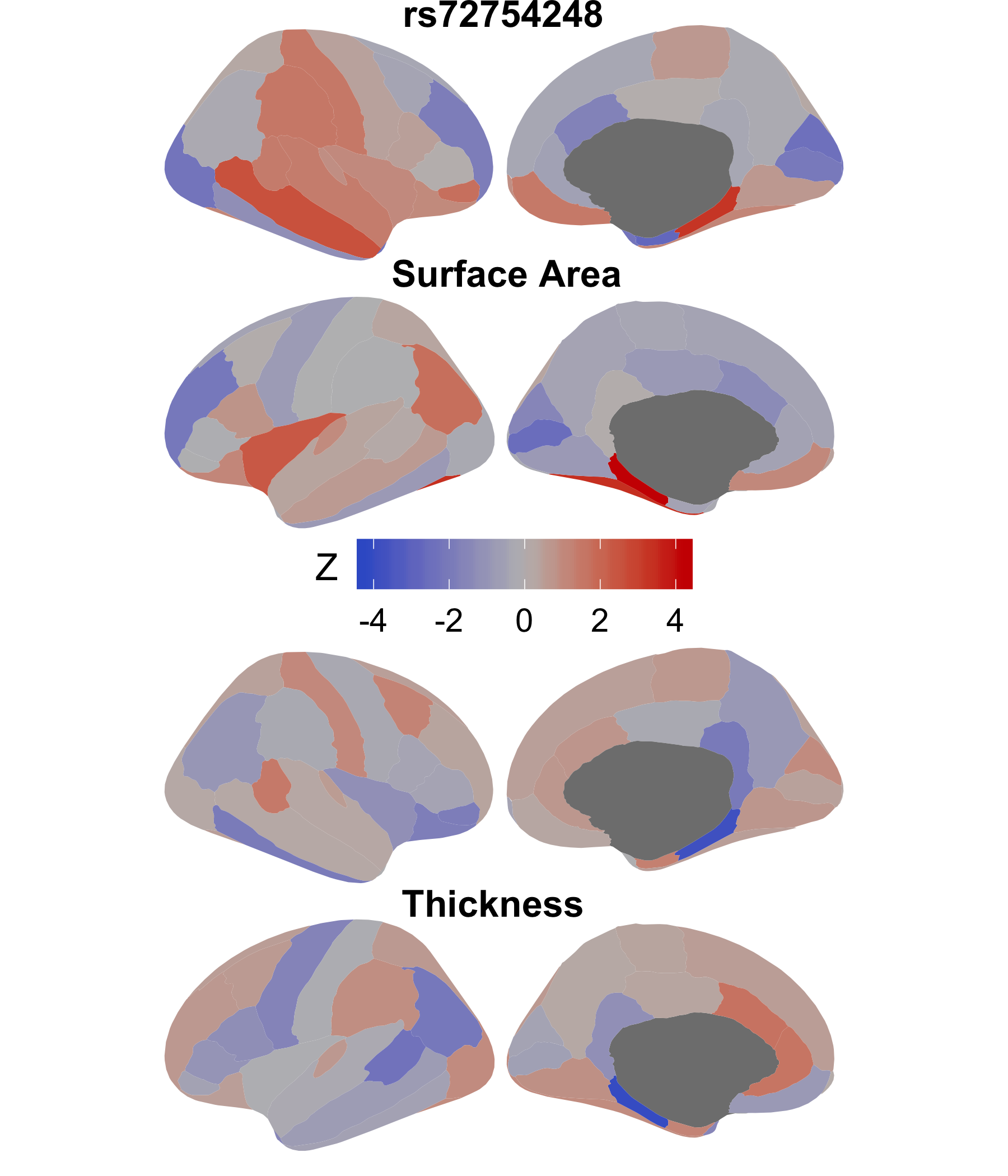

Supplement: Supplementary file 17 — Supplementary Data 14 [file 41467_2020_17368_MOESM17_ESM.gz › BrainMaps/most_aseg_vol/BrainMap005_rs72754248.png]

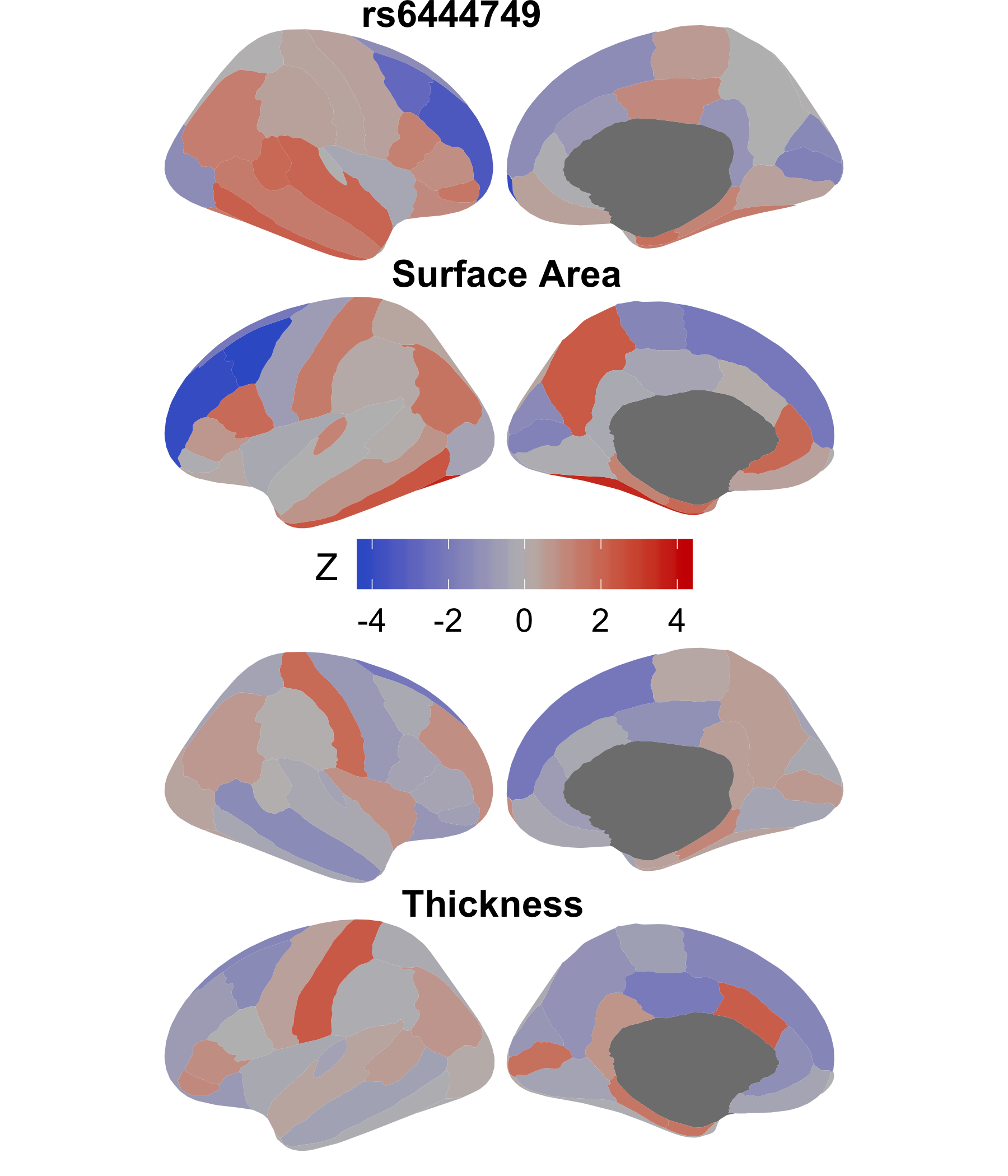

Supplement: Supplementary file 17 — Supplementary Data 14 [file 41467_2020_17368_MOESM17_ESM.gz › BrainMaps/most_aseg_vol/BrainMap116_rs6444749.png]

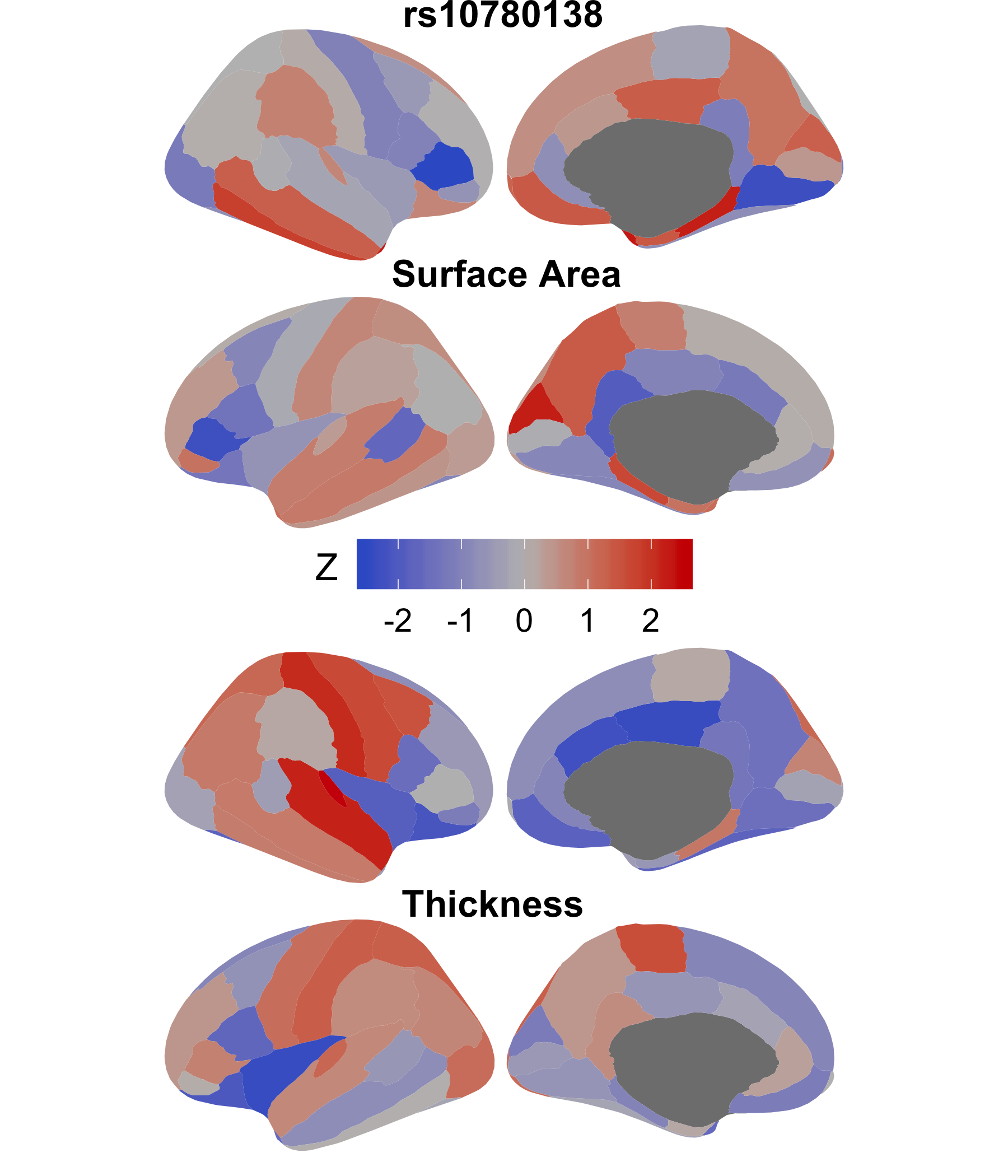

Supplement: Supplementary file 17 — Supplementary Data 14 [file 41467_2020_17368_MOESM17_ESM.gz › BrainMaps/most_aseg_vol/BrainMap176_rs10780138.png]

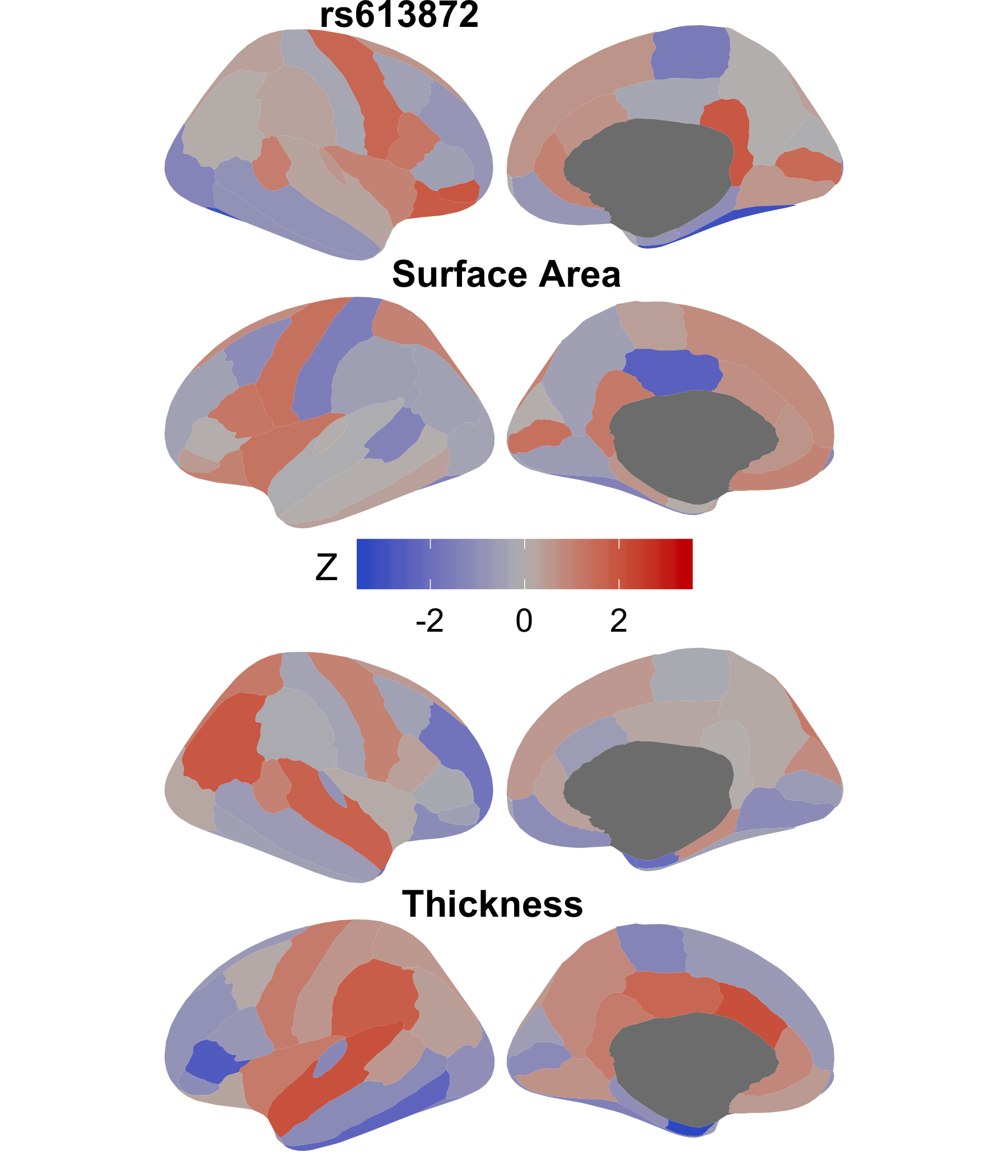

Supplement: Supplementary file 17 — Supplementary Data 14 [file 41467_2020_17368_MOESM17_ESM.gz › BrainMaps/most_aseg_vol/BrainMap142_rs613872.png]

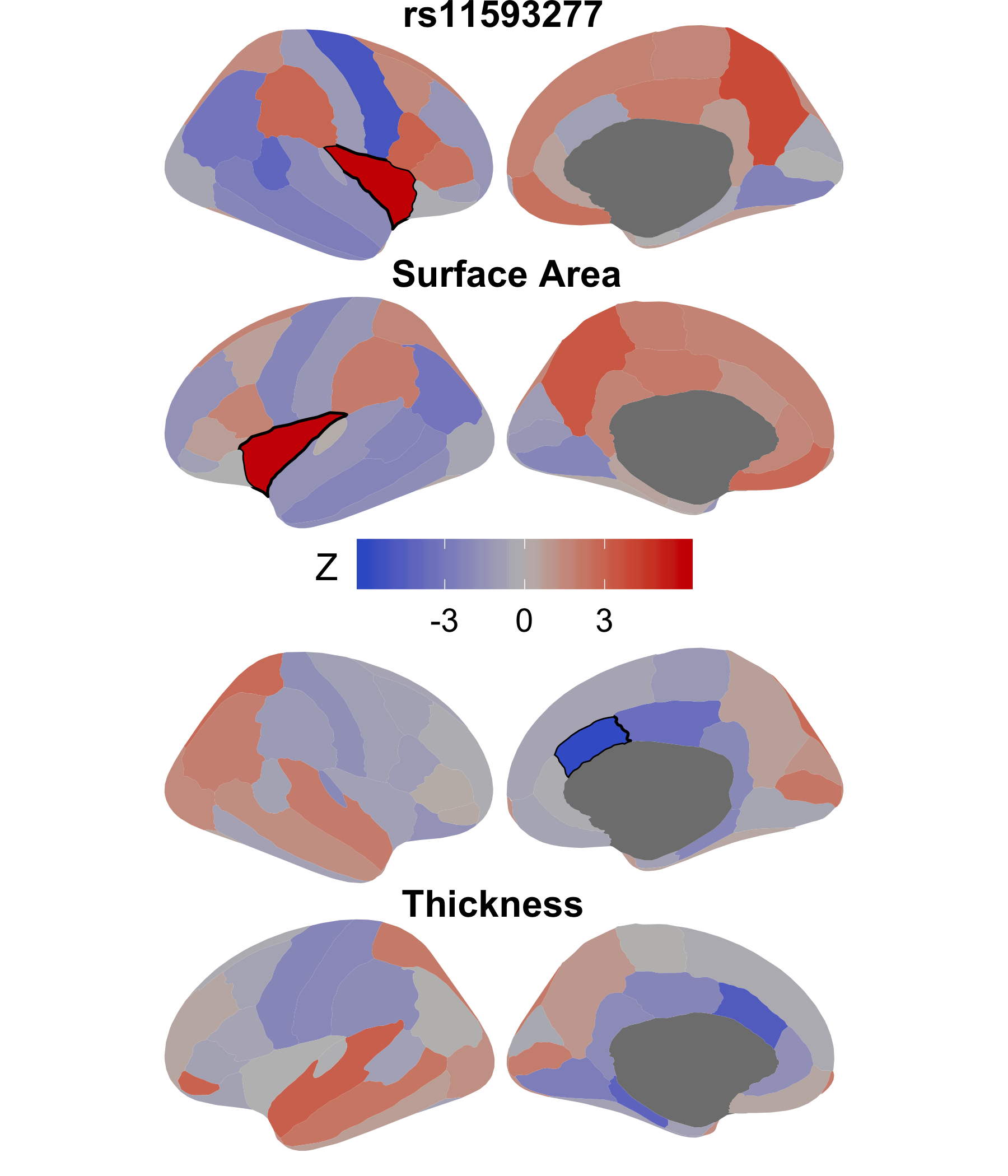

Supplement: Supplementary file 17 — Supplementary Data 14 [file 41467_2020_17368_MOESM17_ESM.gz › BrainMaps/most_aseg_vol/BrainMap071_rs11593277.png]

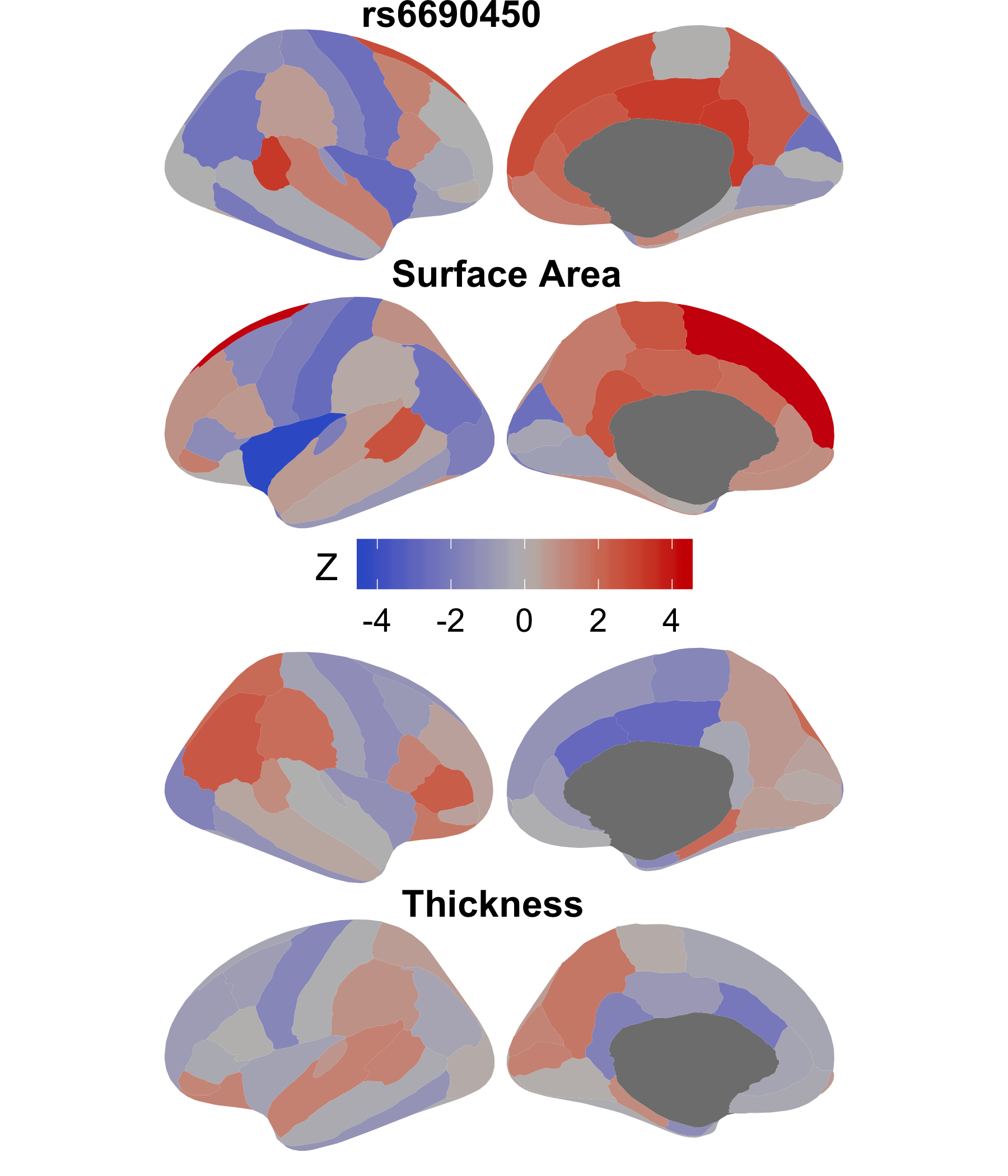

Supplement: Supplementary file 17 — Supplementary Data 14 [file 41467_2020_17368_MOESM17_ESM.gz › BrainMaps/most_aseg_vol/BrainMap076_rs6690450.png]

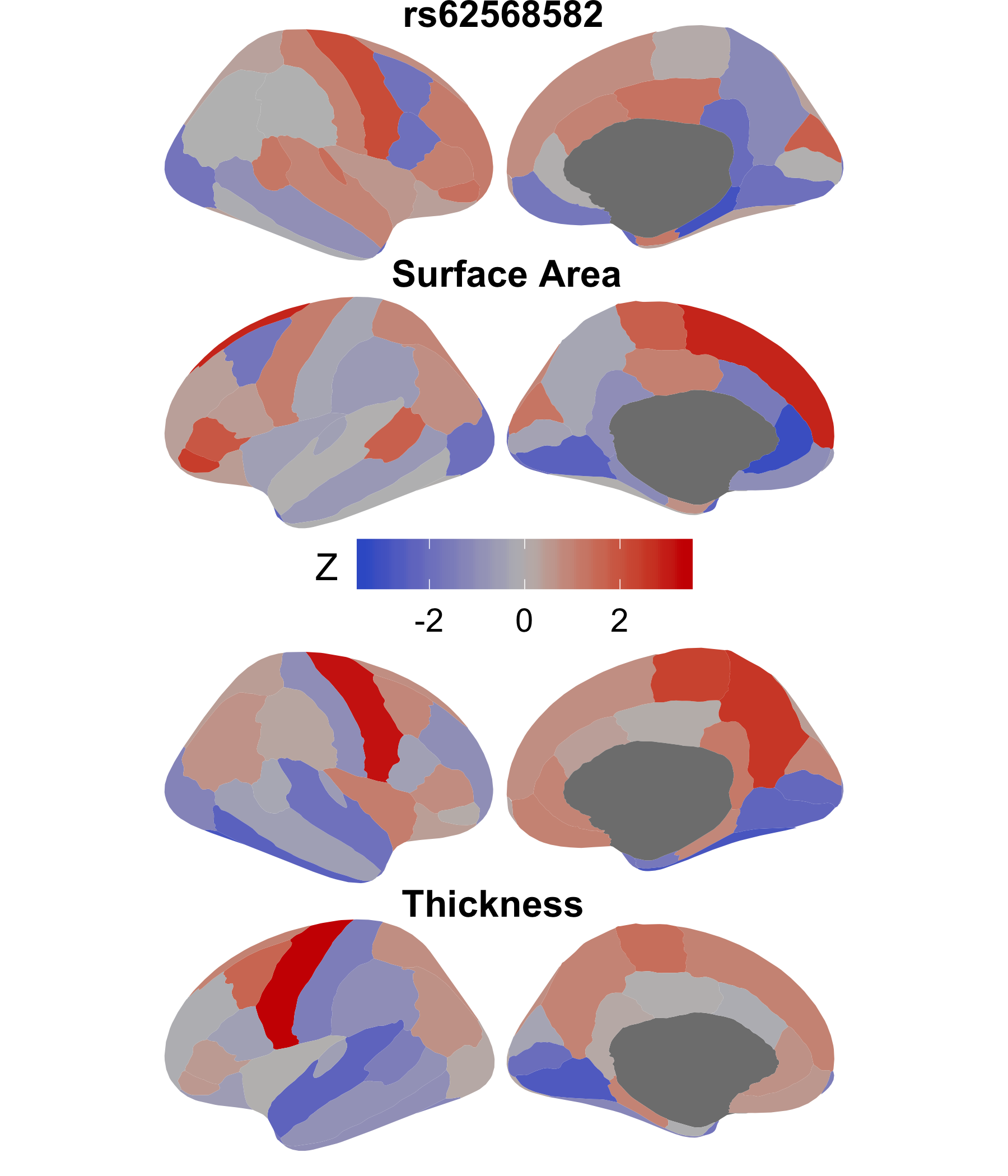

Supplement: Supplementary file 17 — Supplementary Data 14 [file 41467_2020_17368_MOESM17_ESM.gz › BrainMaps/most_aseg_vol/BrainMap177_rs62568582.png]

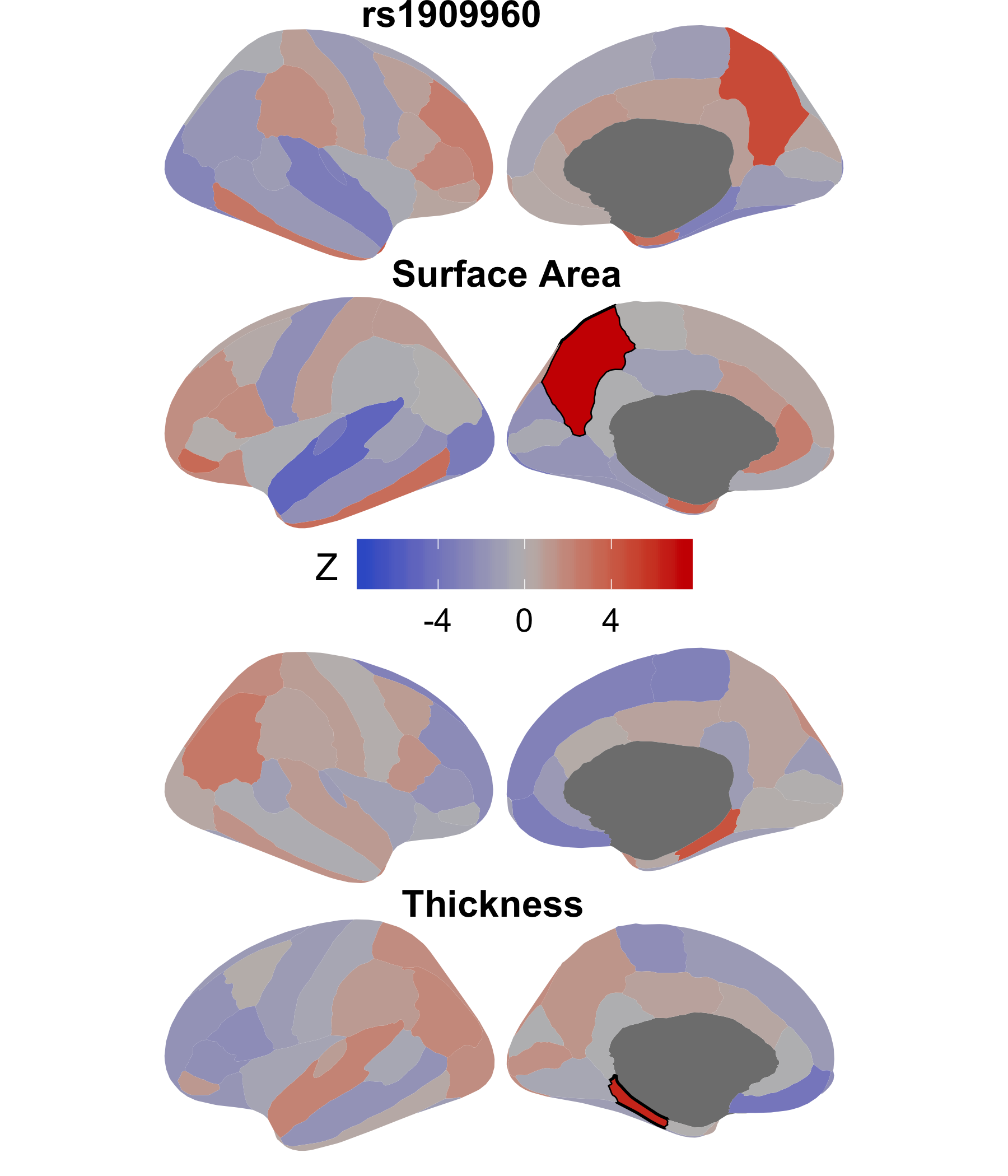

Supplement: Supplementary file 17 — Supplementary Data 14 [file 41467_2020_17368_MOESM17_ESM.gz › BrainMaps/most_aseg_vol/BrainMap002_rs1909960.png]

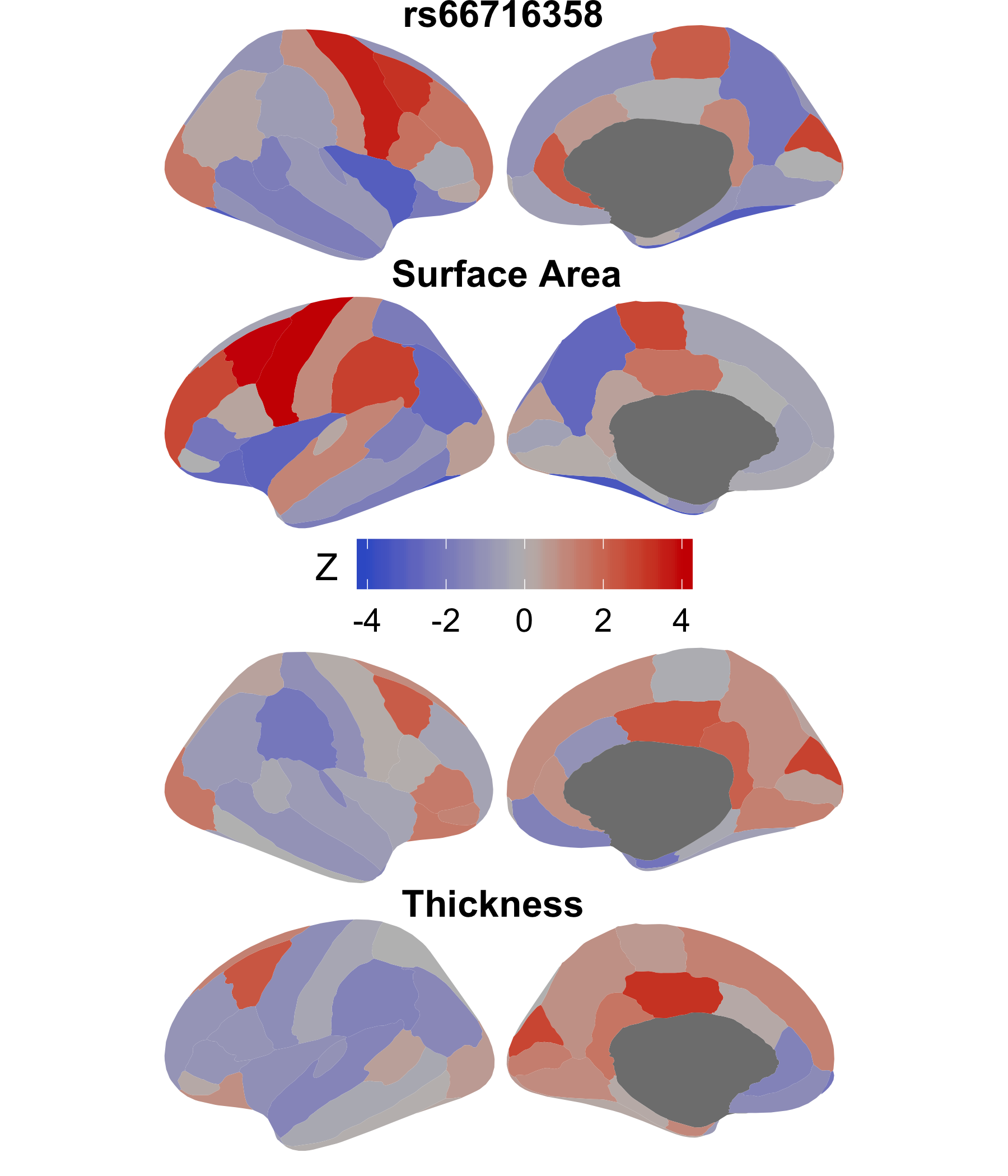

Supplement: Supplementary file 17 — Supplementary Data 14 [file 41467_2020_17368_MOESM17_ESM.gz › BrainMaps/most_aseg_vol/BrainMap064_rs66716358.png]

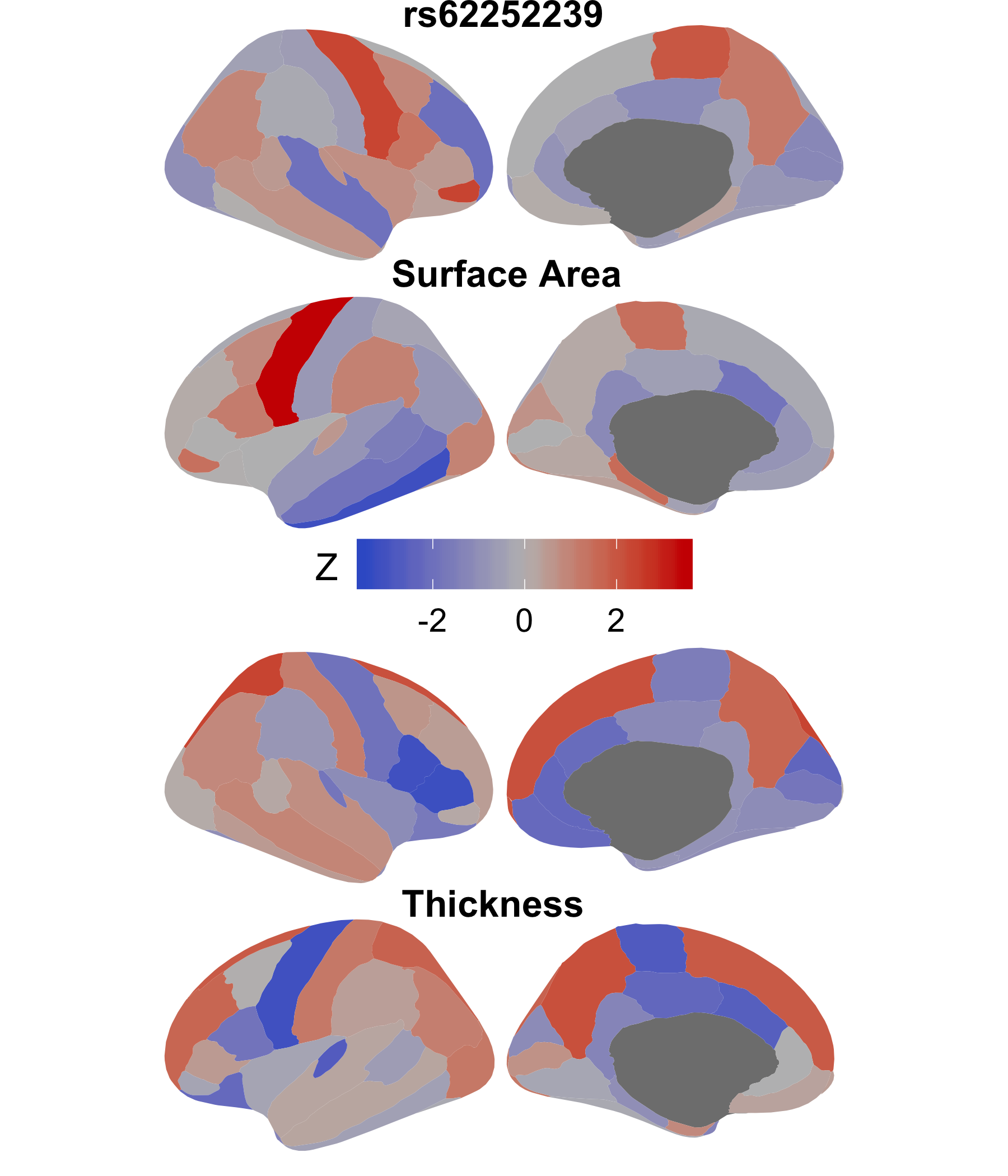

Supplement: Supplementary file 17 — Supplementary Data 14 [file 41467_2020_17368_MOESM17_ESM.gz › BrainMaps/most_aseg_vol/BrainMap131_rs62252239.png]

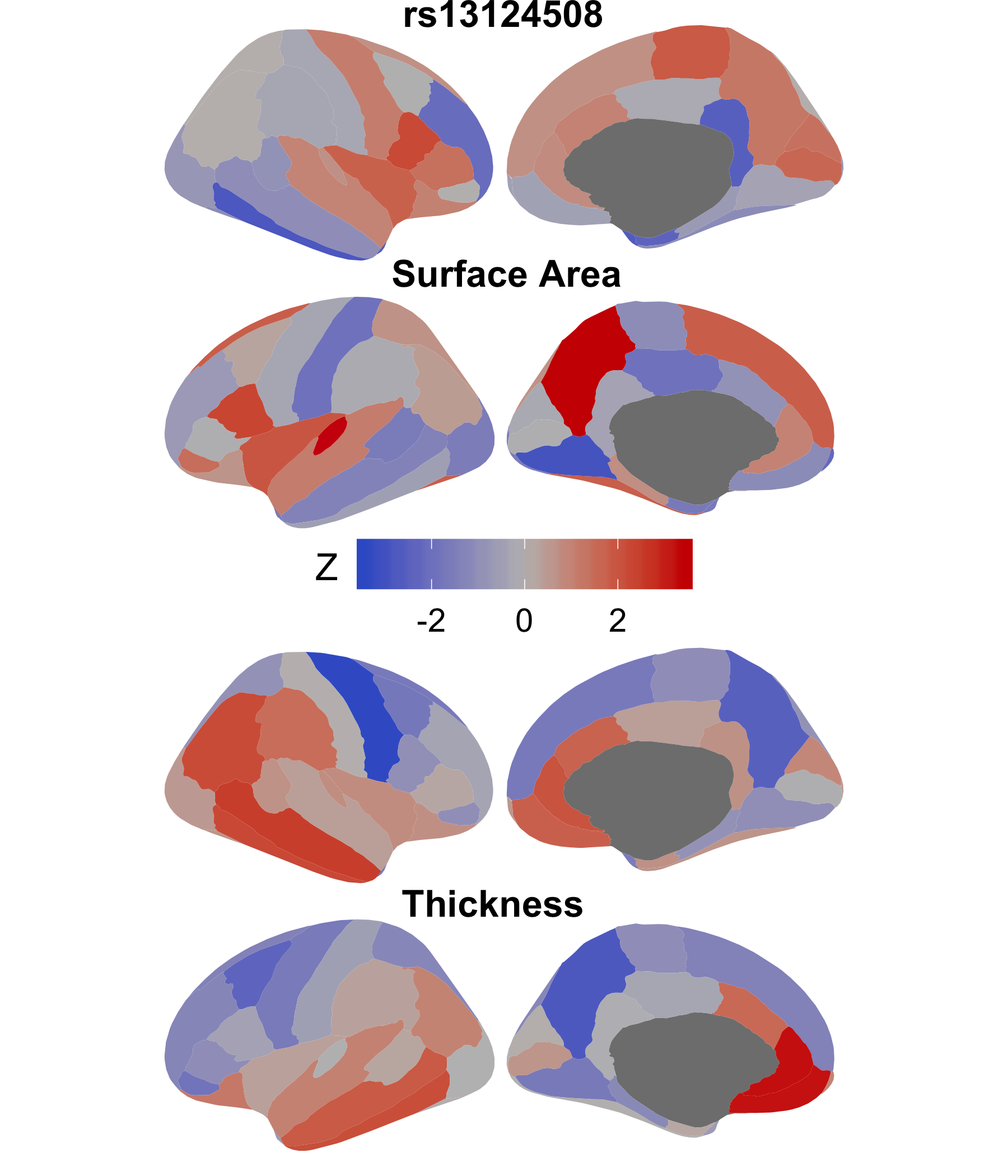

Supplement: Supplementary file 17 — Supplementary Data 14 [file 41467_2020_17368_MOESM17_ESM.gz › BrainMaps/most_aseg_vol/BrainMap094_rs13124508.png]

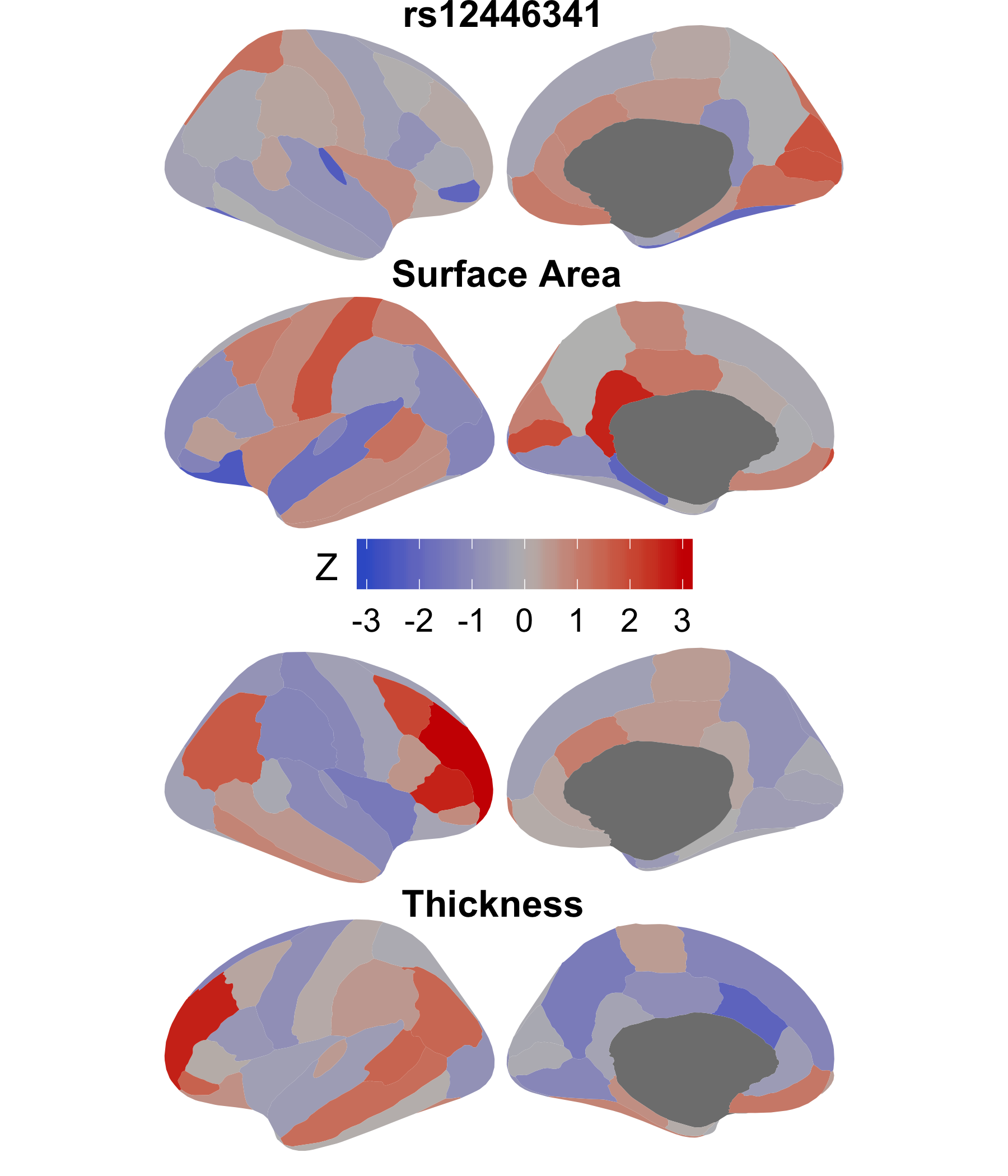

Supplement: Supplementary file 17 — Supplementary Data 14 [file 41467_2020_17368_MOESM17_ESM.gz › BrainMaps/most_aseg_vol/BrainMap083_rs12446341.png]

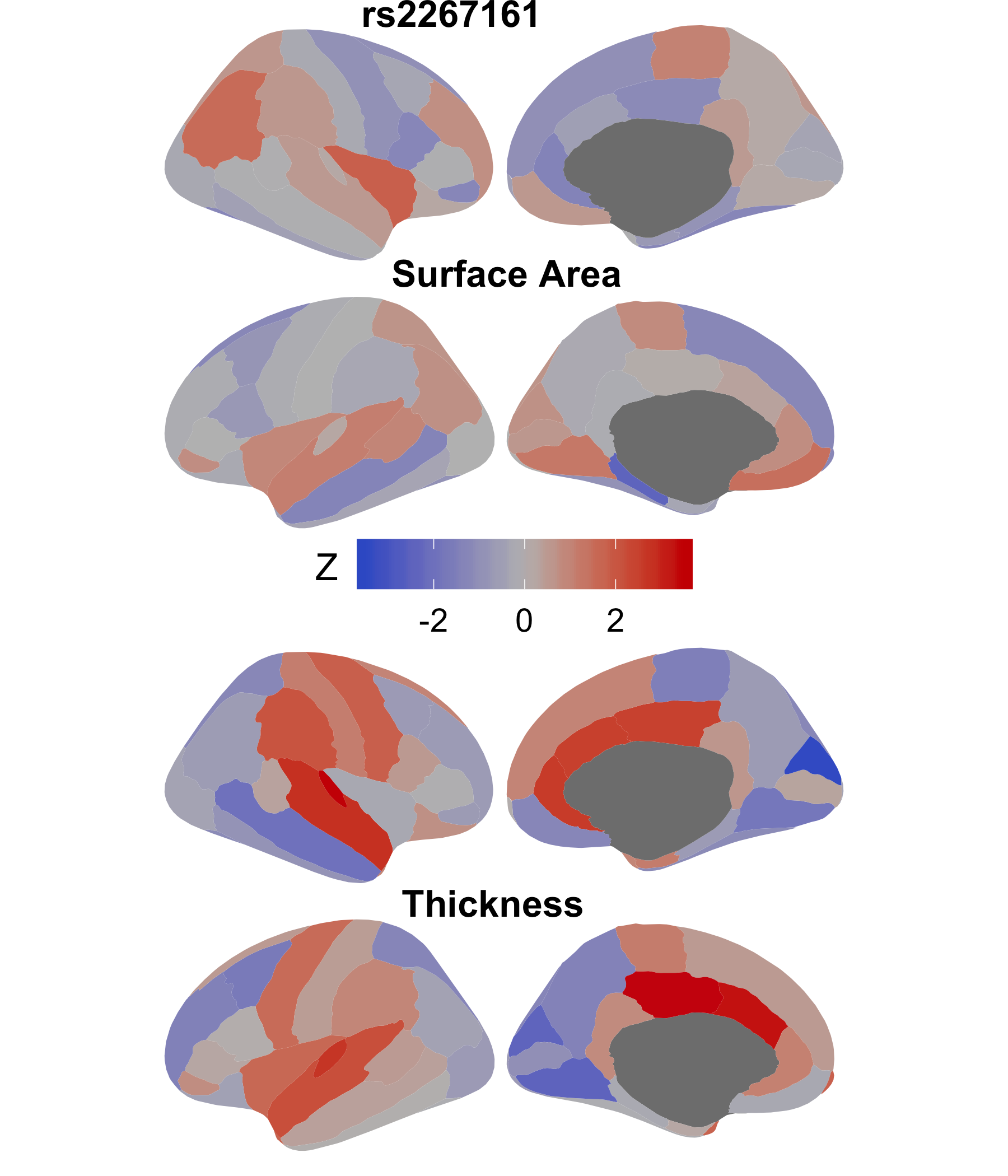

Supplement: Supplementary file 17 — Supplementary Data 14 [file 41467_2020_17368_MOESM17_ESM.gz › BrainMaps/most_aseg_vol/BrainMap053_rs2267161.png]

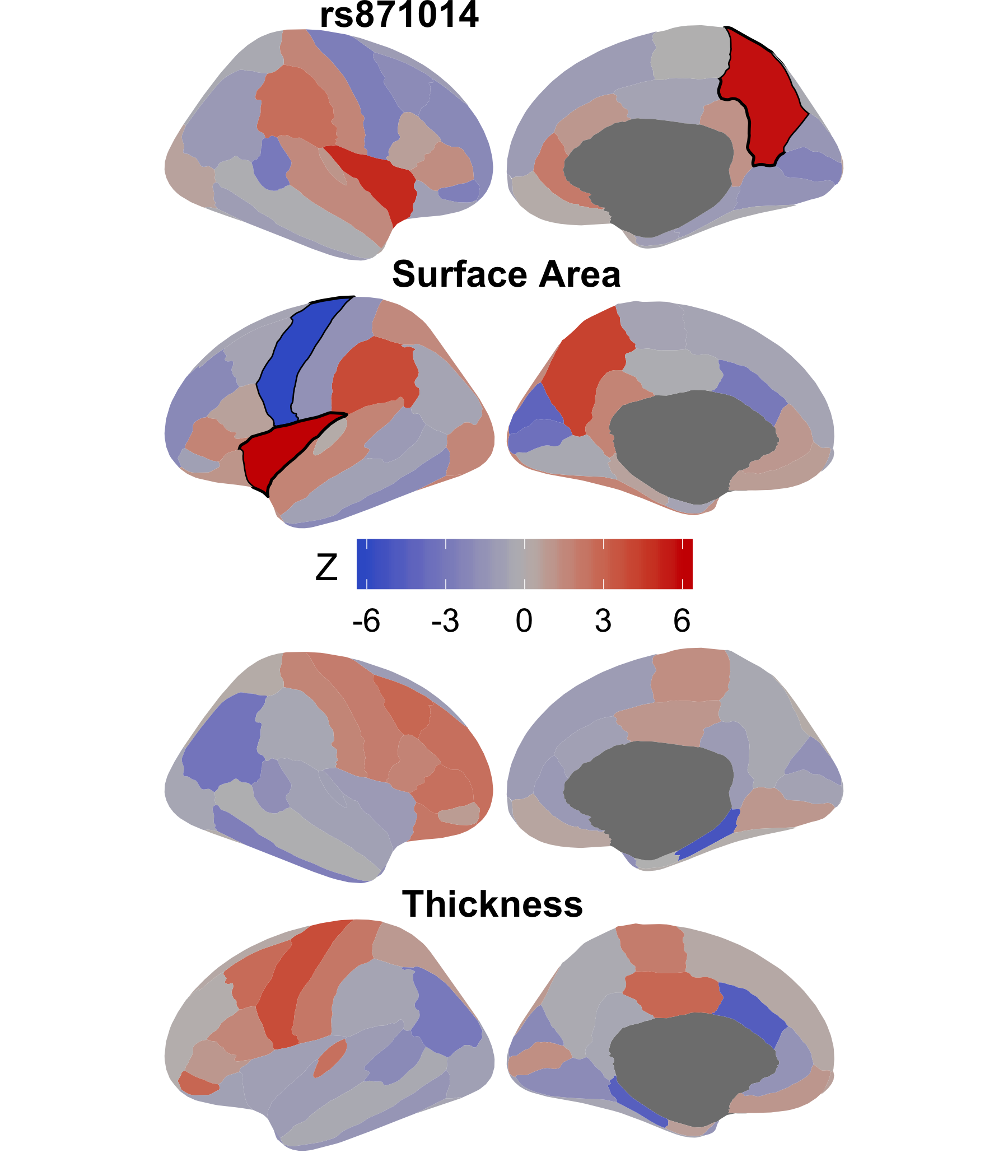

Supplement: Supplementary file 17 — Supplementary Data 14 [file 41467_2020_17368_MOESM17_ESM.gz › BrainMaps/most_aseg_vol/BrainMap062_rs871014.png]

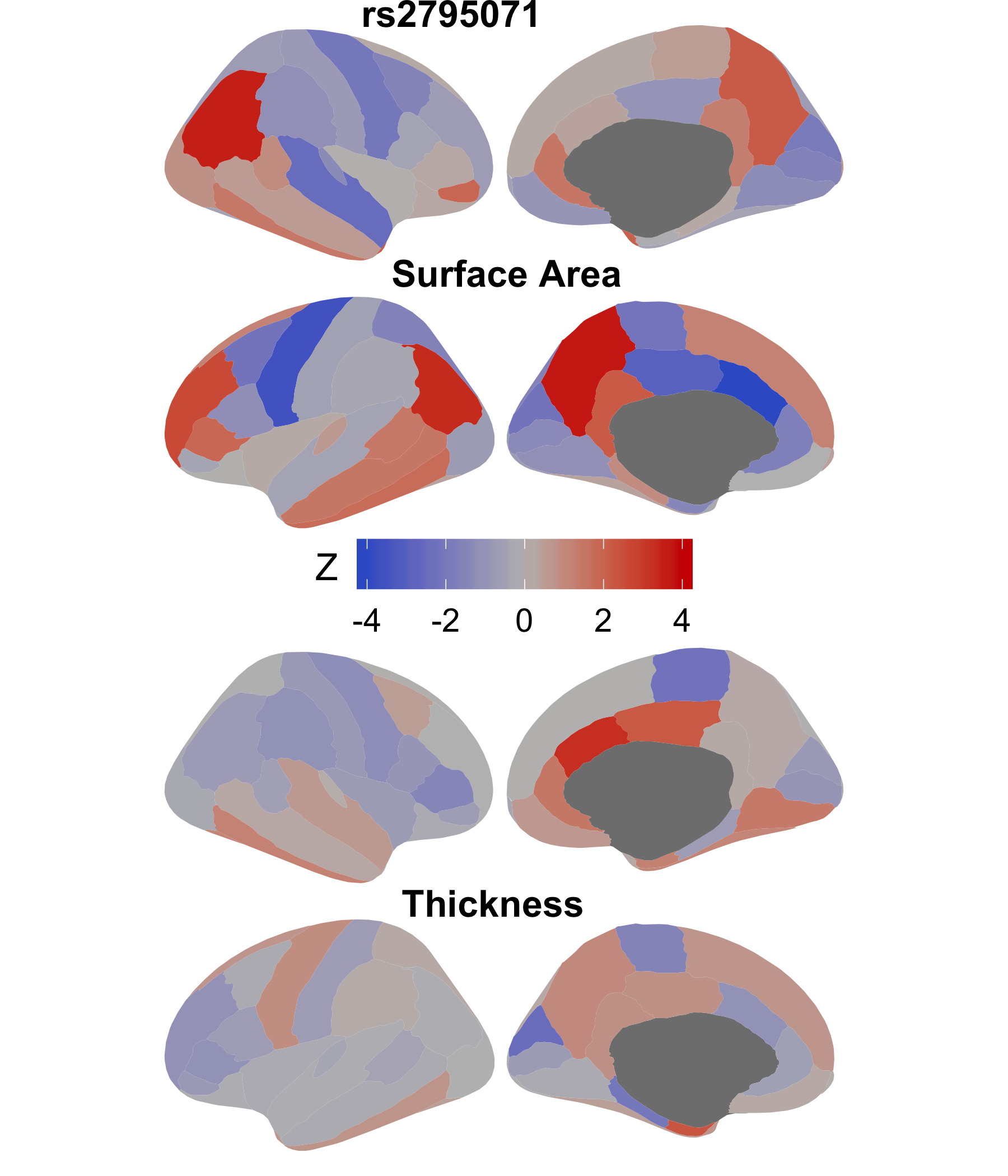

Supplement: Supplementary file 17 — Supplementary Data 14 [file 41467_2020_17368_MOESM17_ESM.gz › BrainMaps/most_aseg_vol/BrainMap174_rs2795071.png]

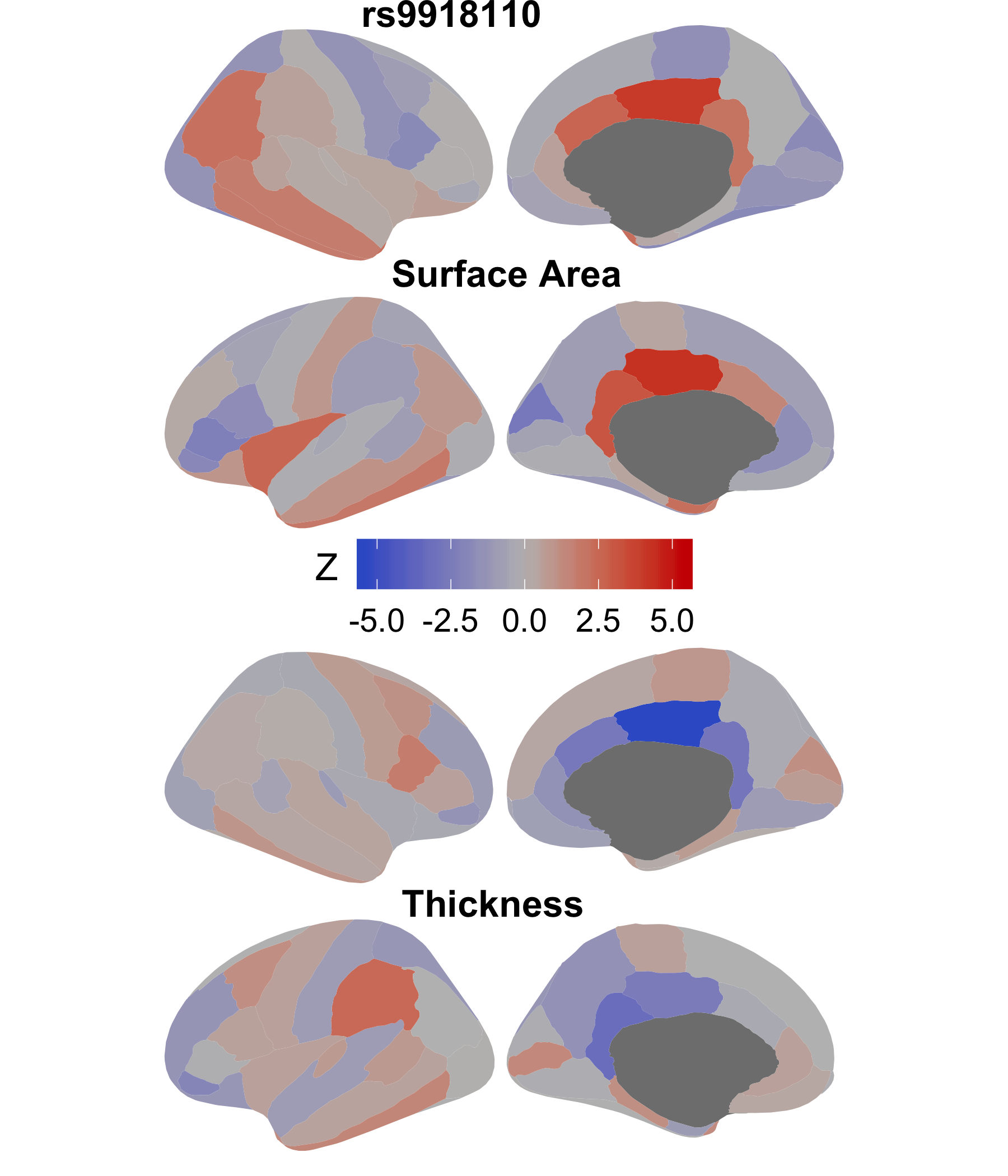

Supplement: Supplementary file 17 — Supplementary Data 14 [file 41467_2020_17368_MOESM17_ESM.gz › BrainMaps/most_aseg_vol/BrainMap087_rs9918110.png]

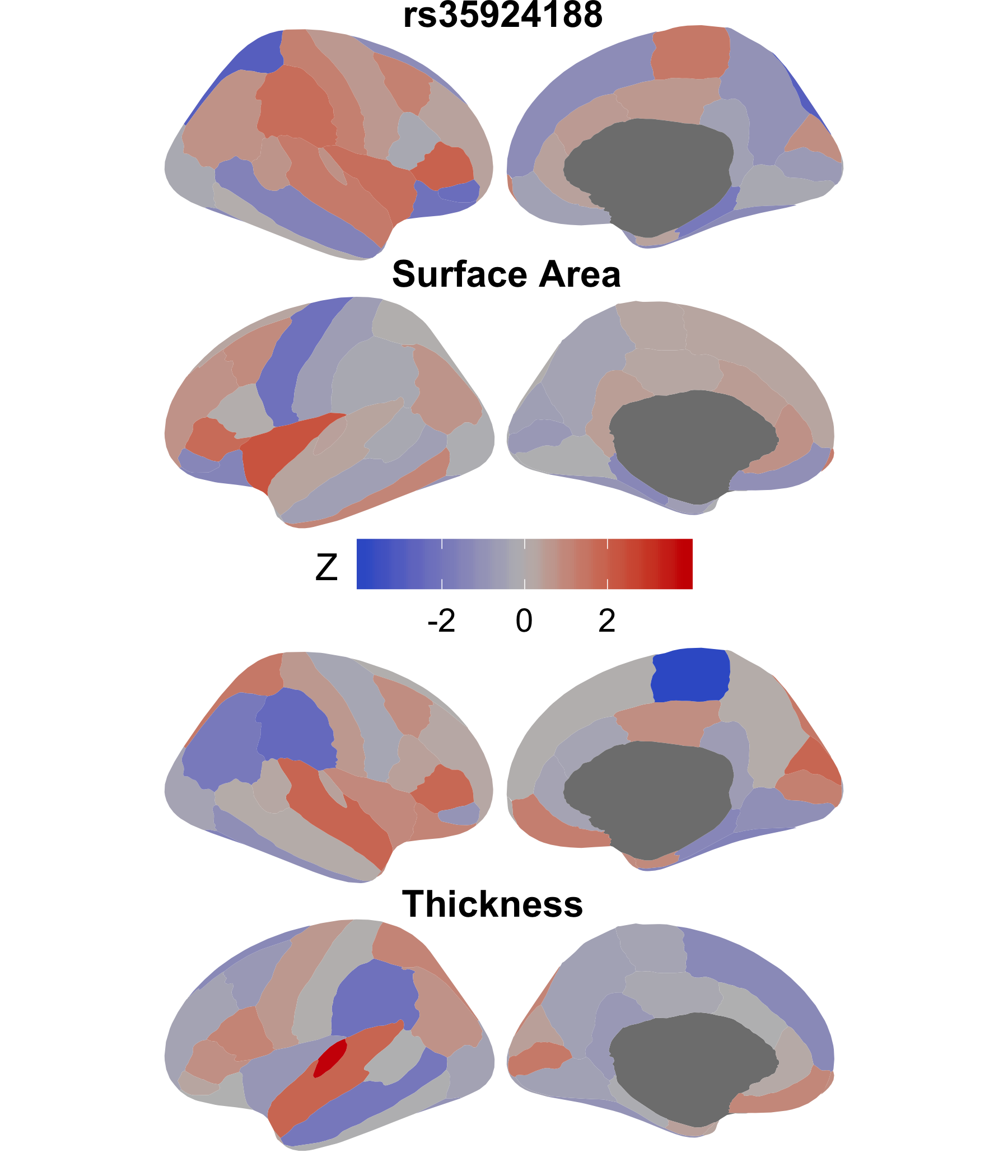

Supplement: Supplementary file 17 — Supplementary Data 14 [file 41467_2020_17368_MOESM17_ESM.gz › BrainMaps/most_aseg_vol/BrainMap101_rs35924188.png]

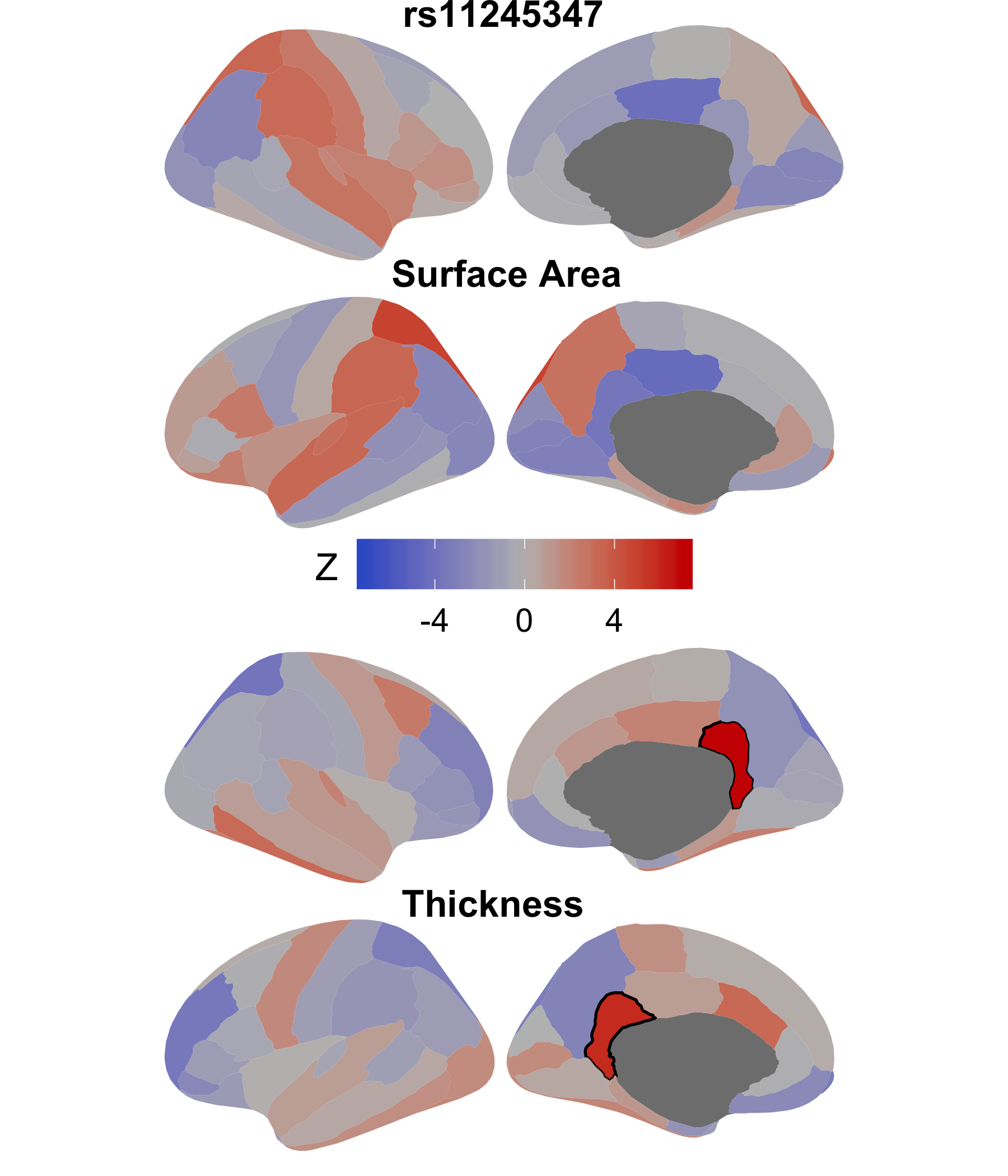

Supplement: Supplementary file 17 — Supplementary Data 14 [file 41467_2020_17368_MOESM17_ESM.gz › BrainMaps/most_aseg_vol/BrainMap004_rs11245347.png]

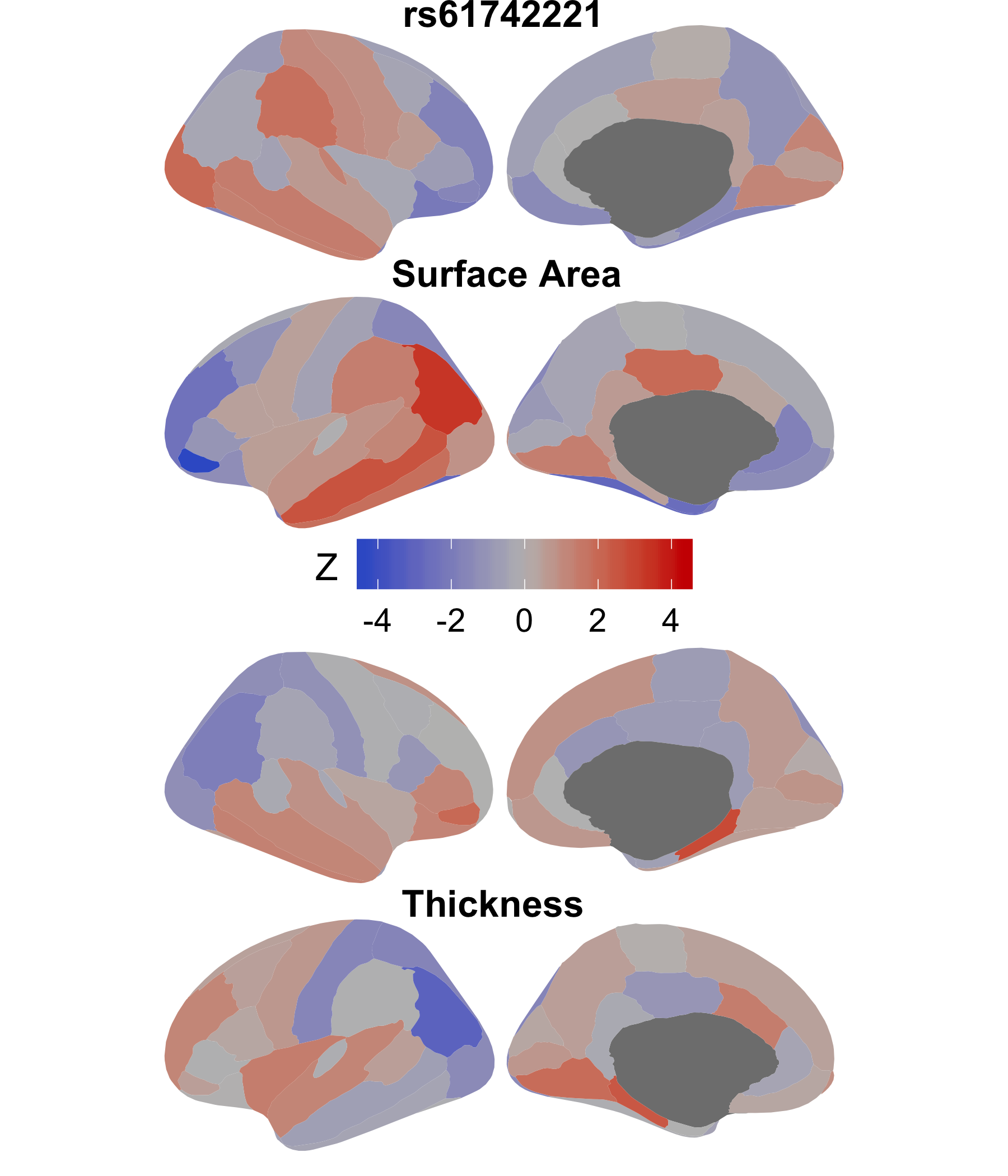

Supplement: Supplementary file 17 — Supplementary Data 14 [file 41467_2020_17368_MOESM17_ESM.gz › BrainMaps/most_aseg_vol/BrainMap160_rs61742221.png]

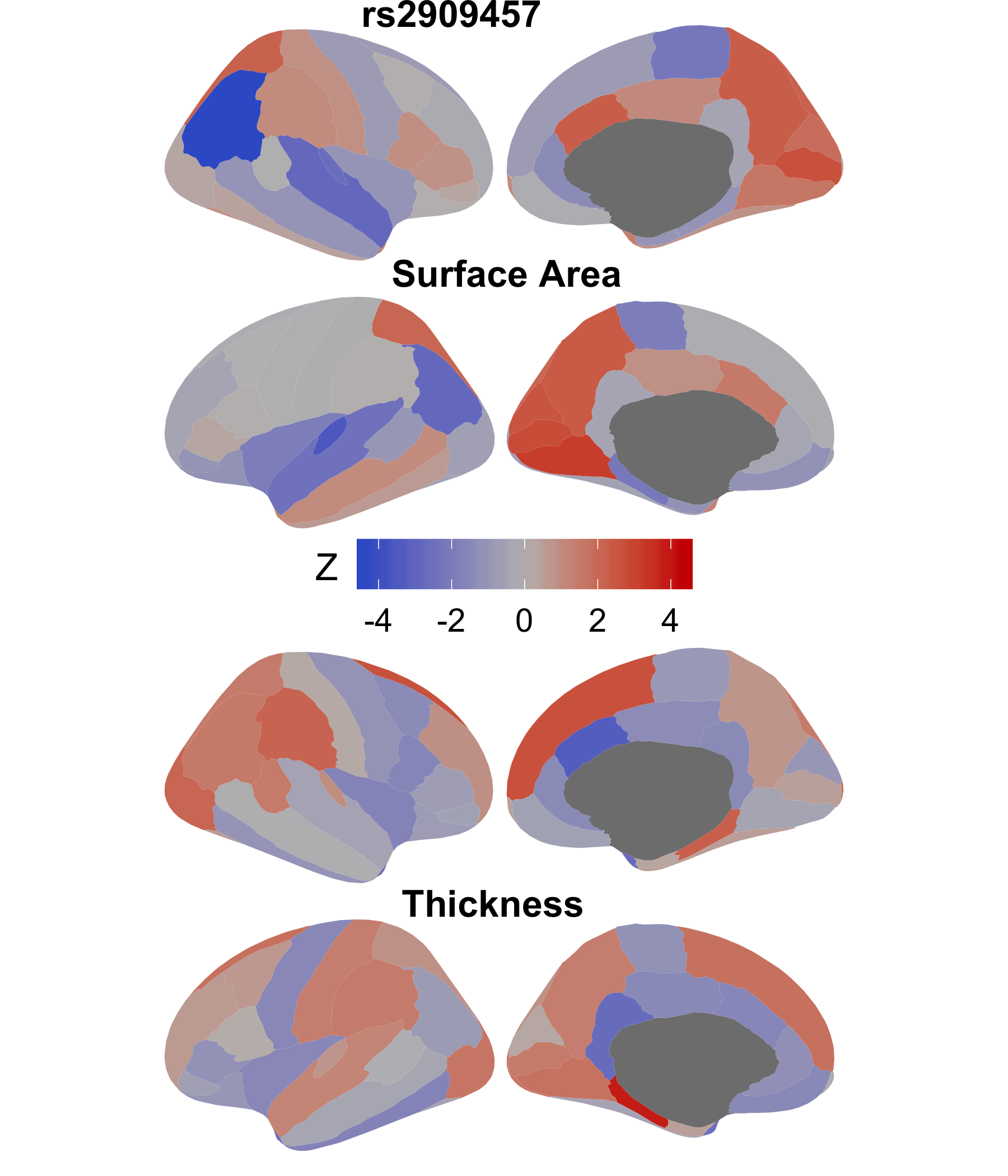

Supplement: Supplementary file 17 — Supplementary Data 14 [file 41467_2020_17368_MOESM17_ESM.gz › BrainMaps/most_aseg_vol/BrainMap020_rs2909457.png]

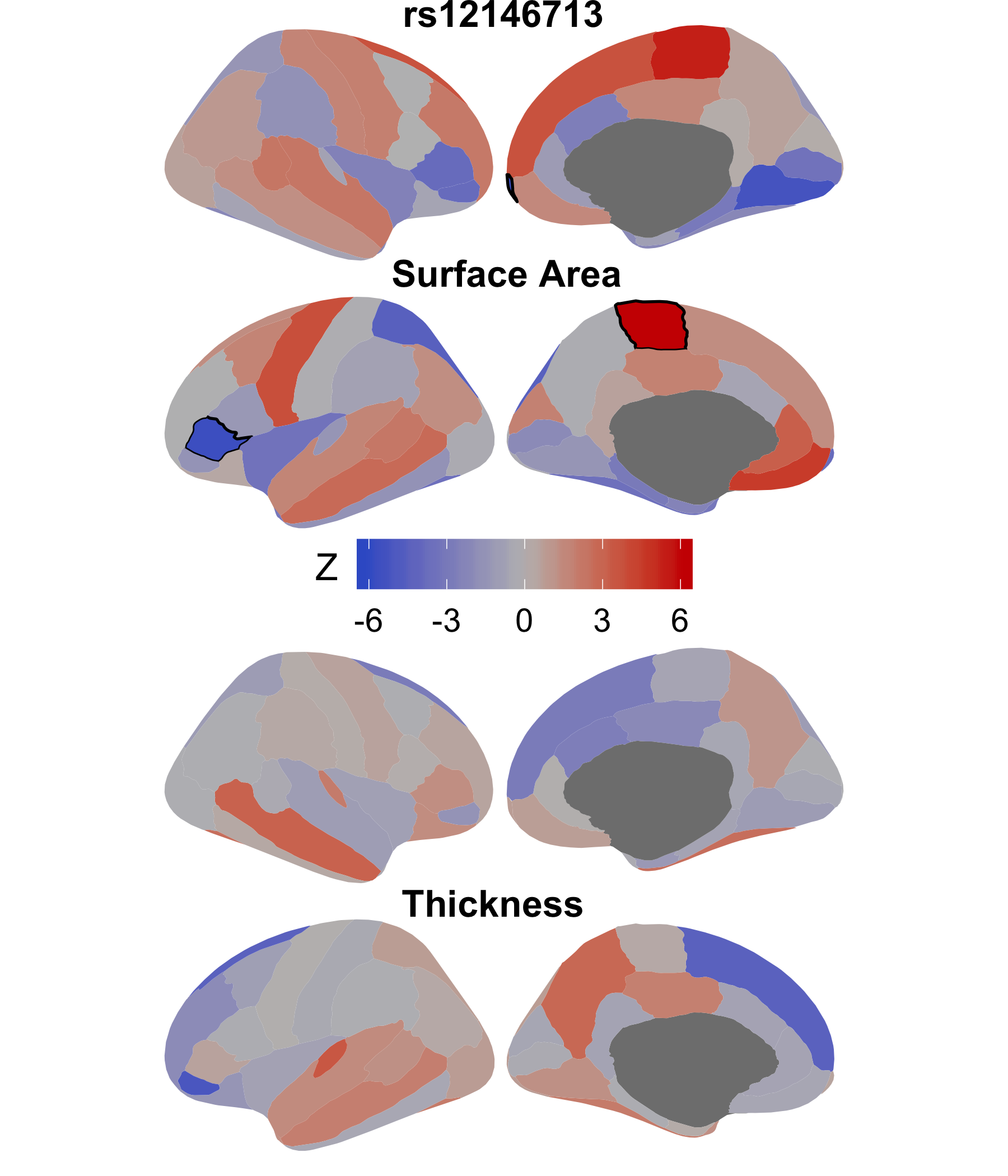

Supplement: Supplementary file 17 — Supplementary Data 14 [file 41467_2020_17368_MOESM17_ESM.gz › BrainMaps/most_aseg_vol/BrainMap013_rs12146713.png]

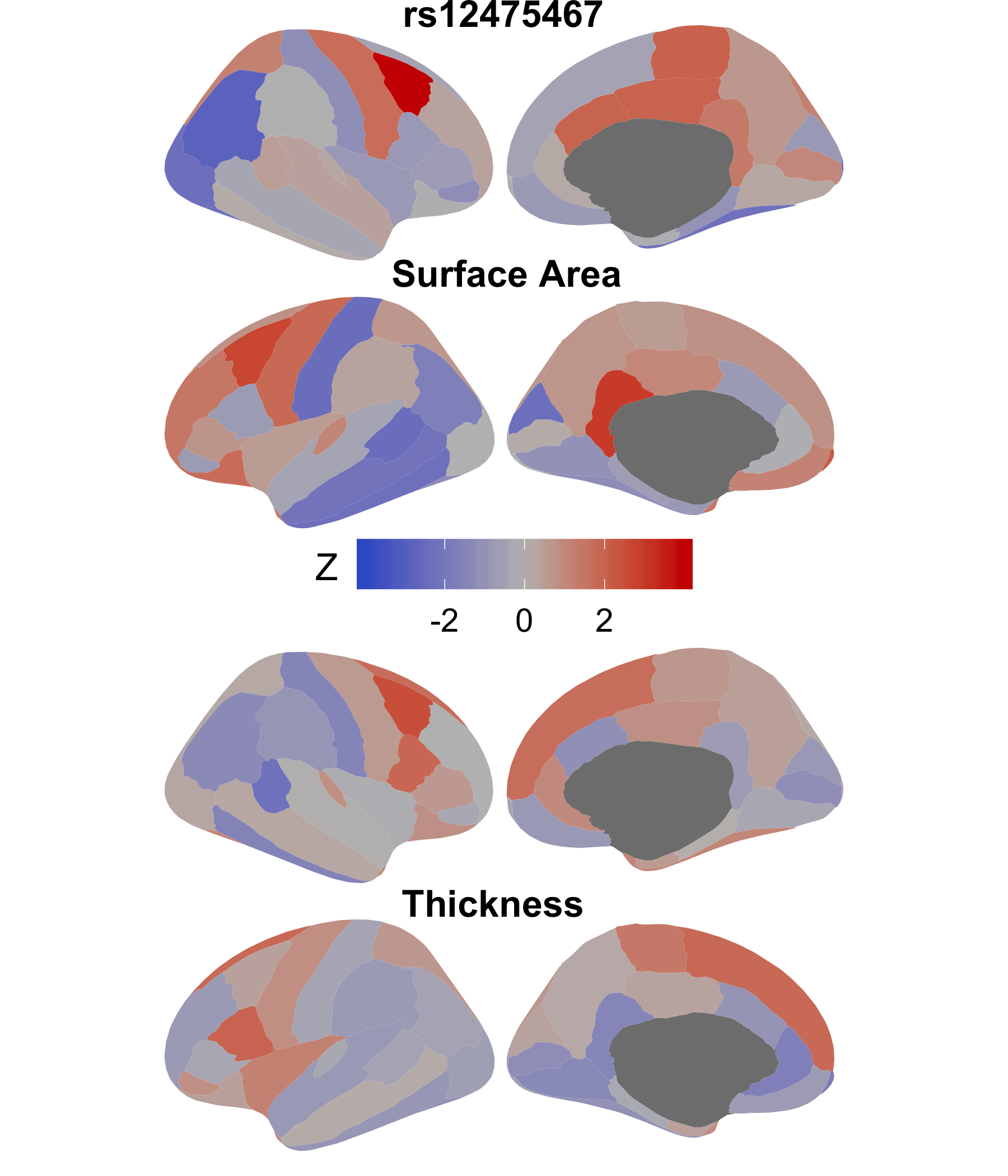

Supplement: Supplementary file 17 — Supplementary Data 14 [file 41467_2020_17368_MOESM17_ESM.gz › BrainMaps/most_aseg_vol/BrainMap168_rs12475467.png]

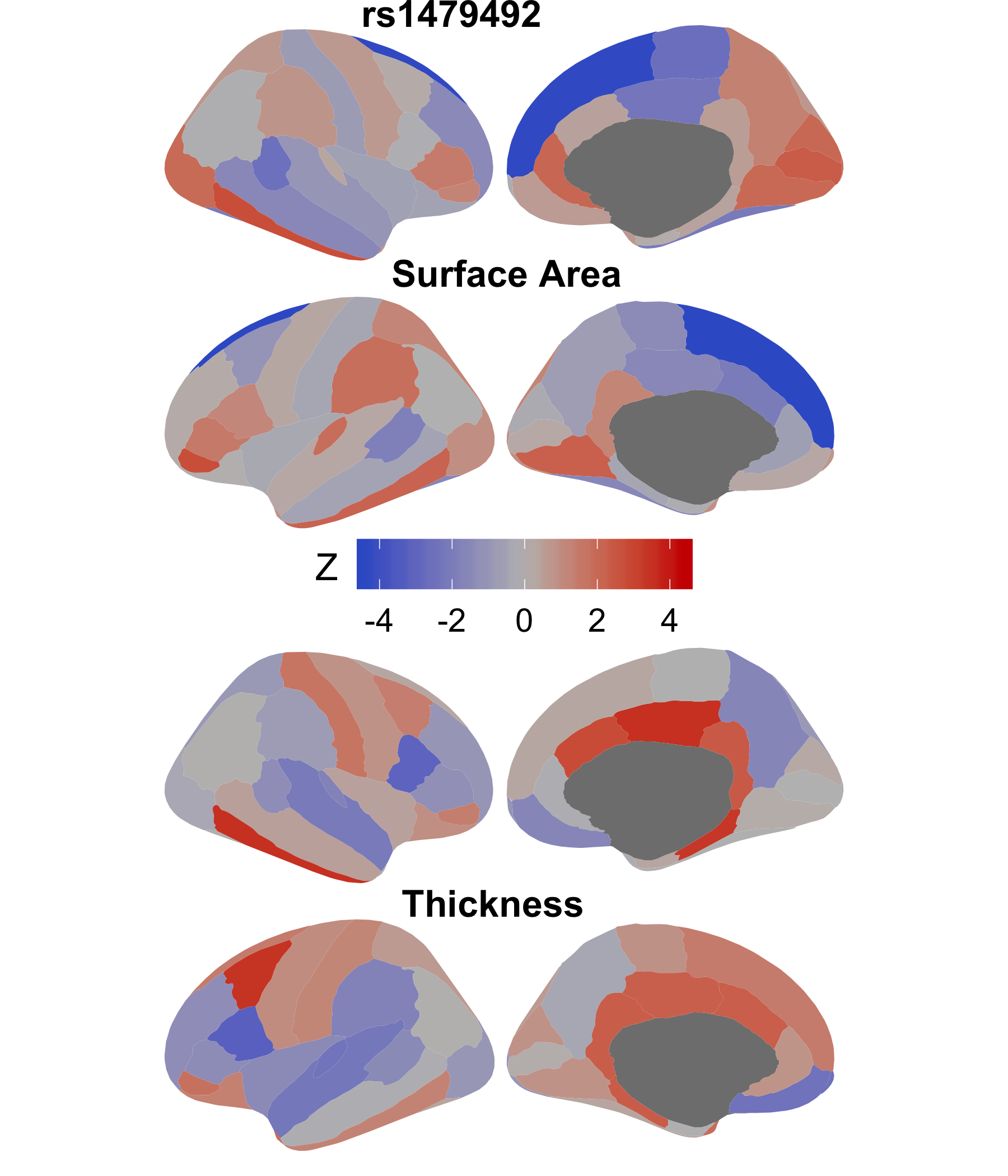

Supplement: Supplementary file 17 — Supplementary Data 14 [file 41467_2020_17368_MOESM17_ESM.gz › BrainMaps/most_aseg_vol/BrainMap019_rs1479492.png]

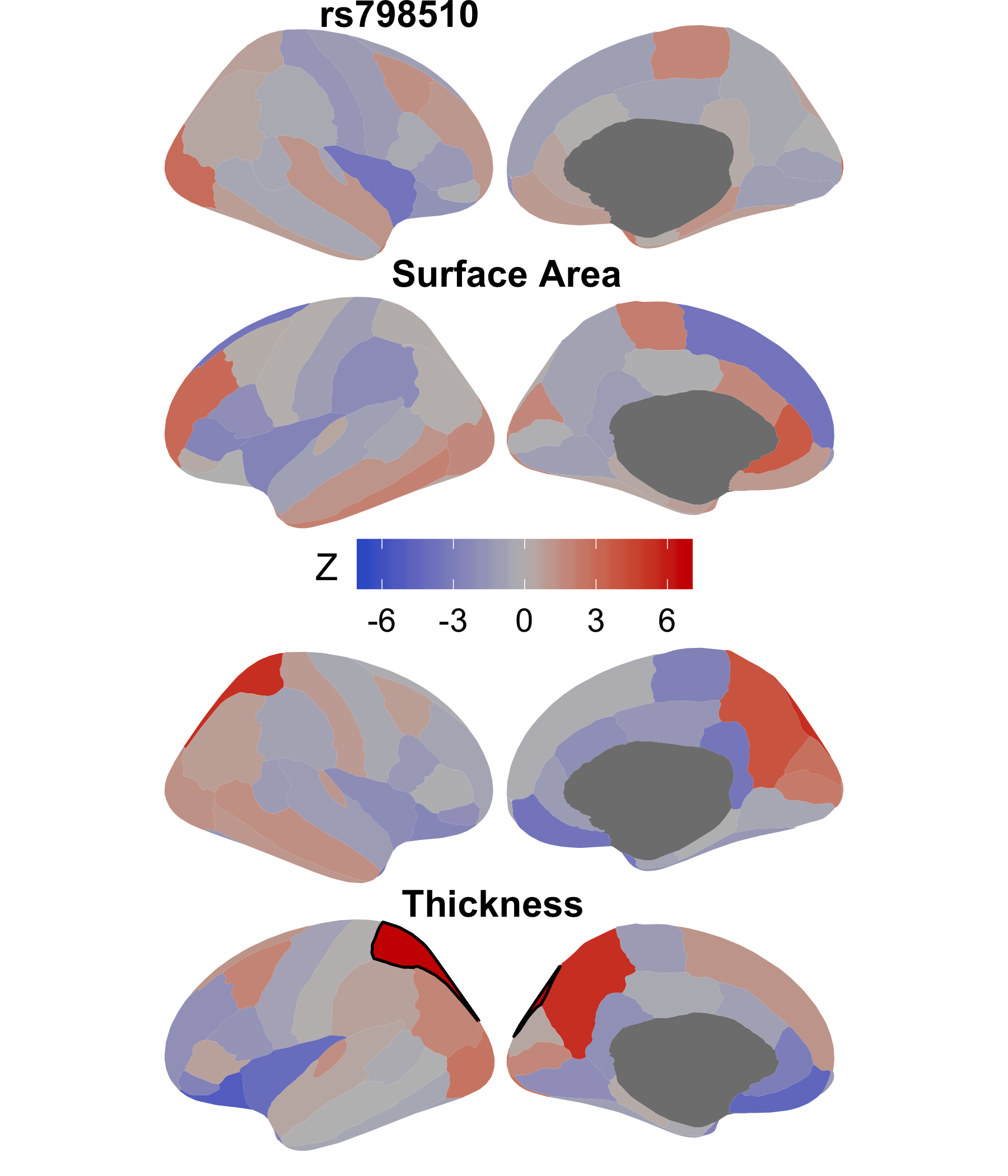

Supplement: Supplementary file 17 — Supplementary Data 14 [file 41467_2020_17368_MOESM17_ESM.gz › BrainMaps/most_aseg_vol/BrainMap051_rs798510.png]

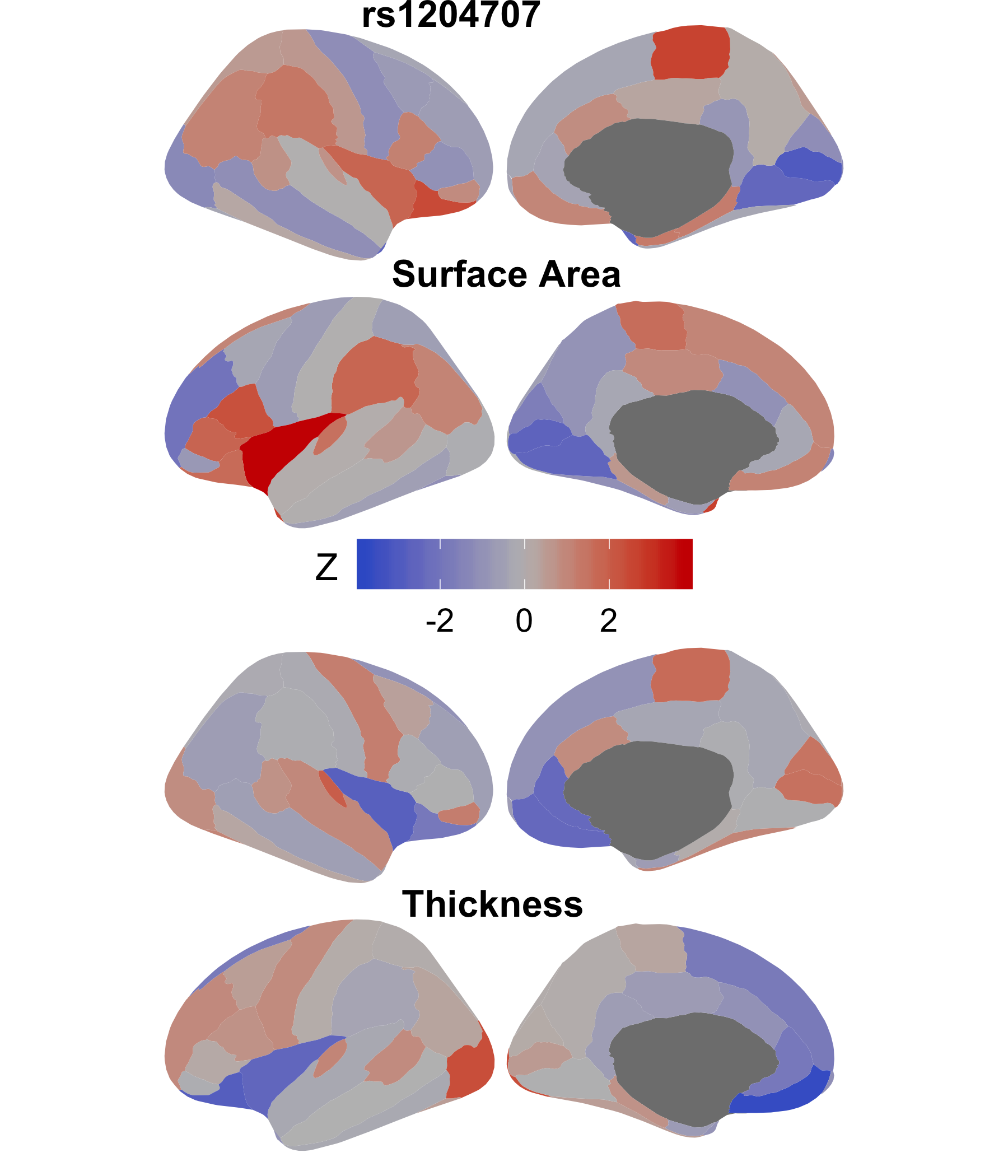

Supplement: Supplementary file 17 — Supplementary Data 14 [file 41467_2020_17368_MOESM17_ESM.gz › BrainMaps/most_aseg_vol/BrainMap145_rs1204707.png]

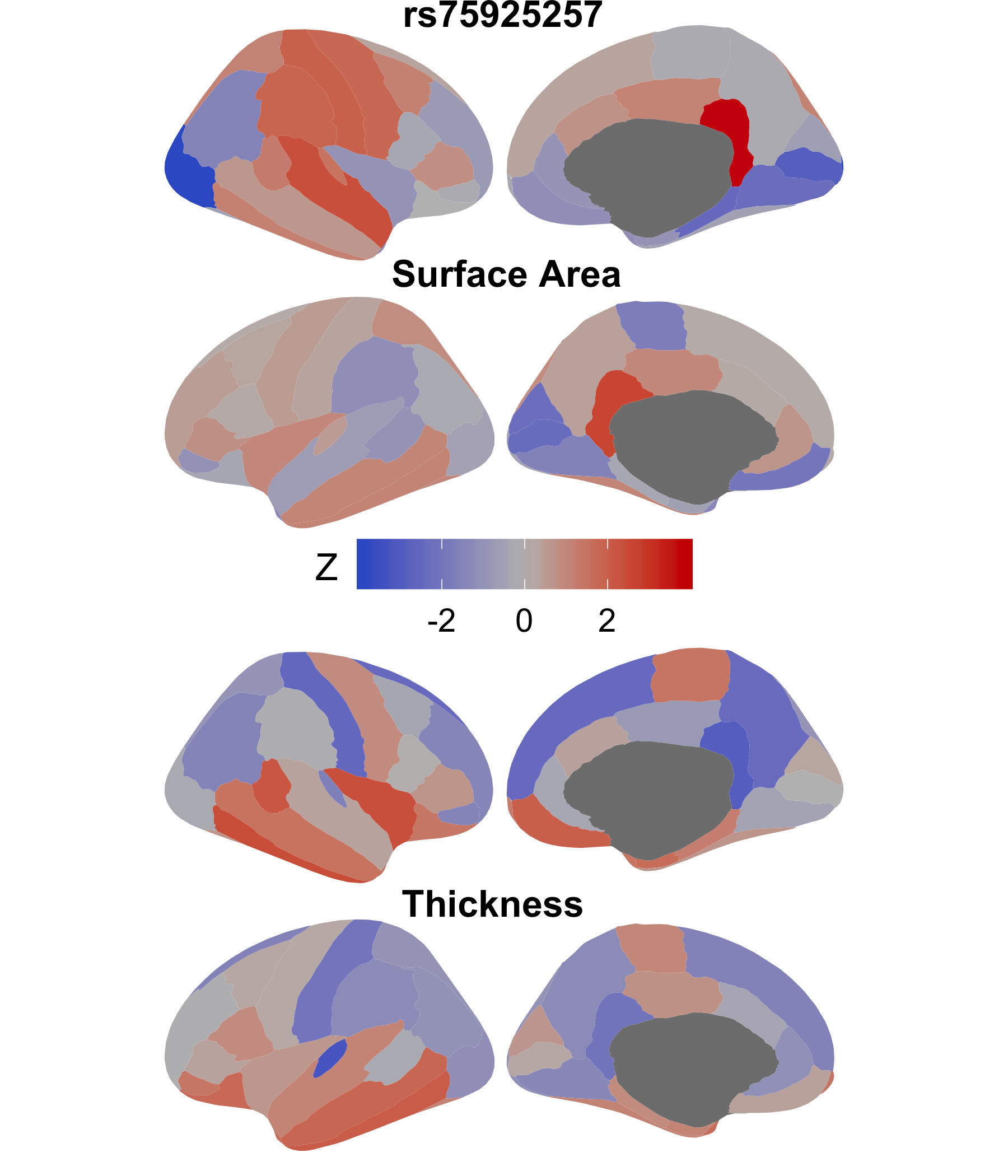

Supplement: Supplementary file 17 — Supplementary Data 14 [file 41467_2020_17368_MOESM17_ESM.gz › BrainMaps/most_aseg_vol/BrainMap034_rs75925257.png]

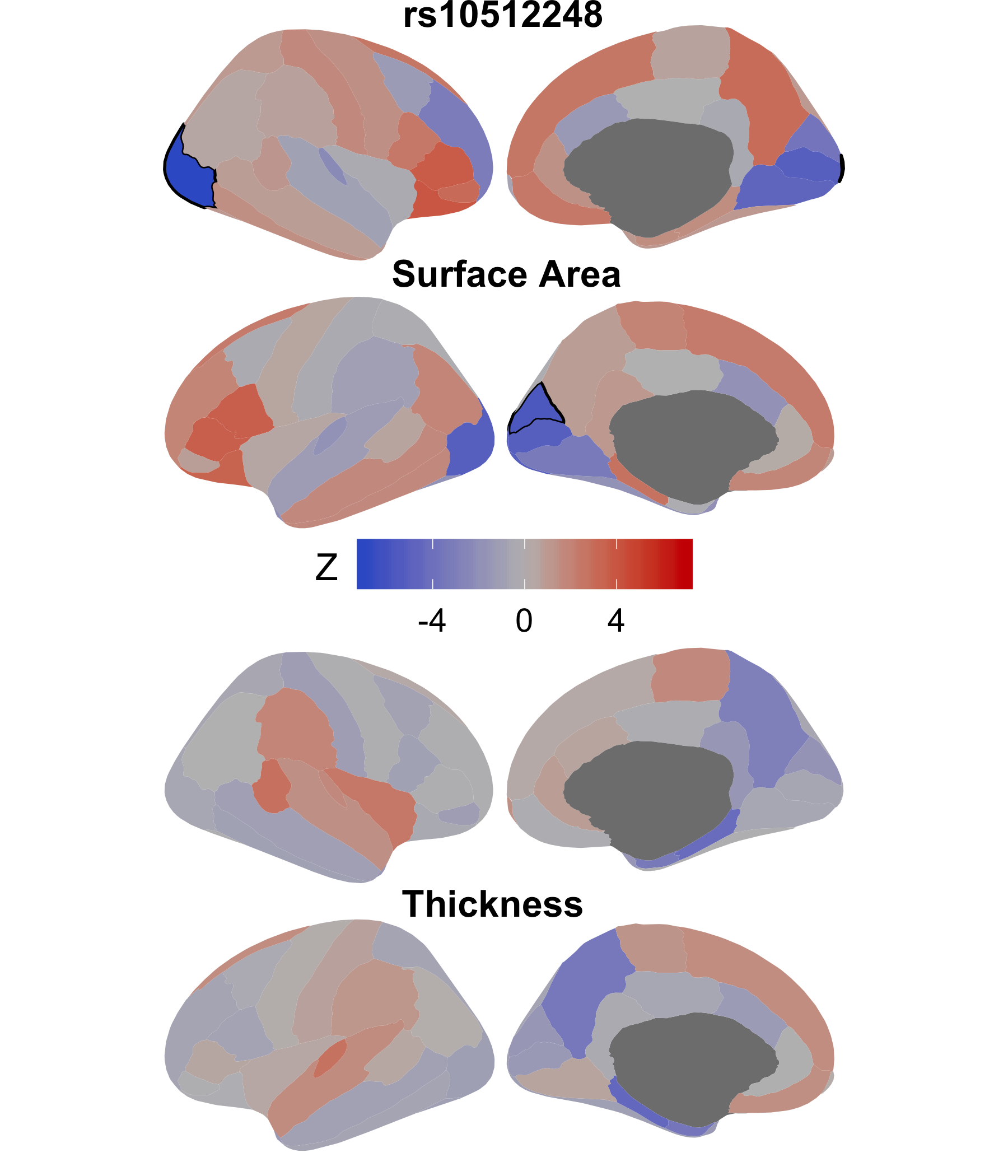

Supplement: Supplementary file 17 — Supplementary Data 14 [file 41467_2020_17368_MOESM17_ESM.gz › BrainMaps/most_aseg_vol/BrainMap152_rs10512248.png]

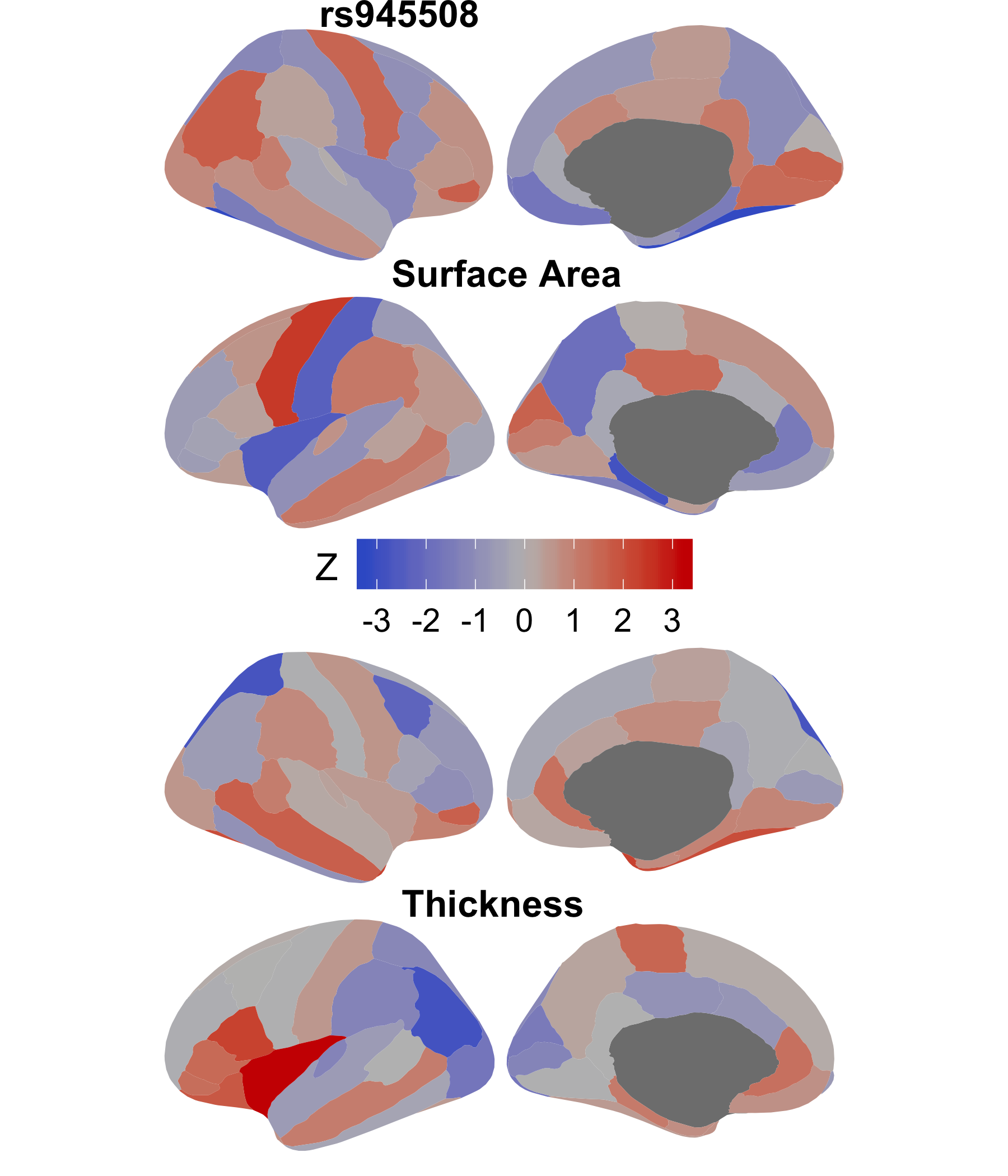

Supplement: Supplementary file 17 — Supplementary Data 14 [file 41467_2020_17368_MOESM17_ESM.gz › BrainMaps/most_aseg_vol/BrainMap039_rs945508.png]

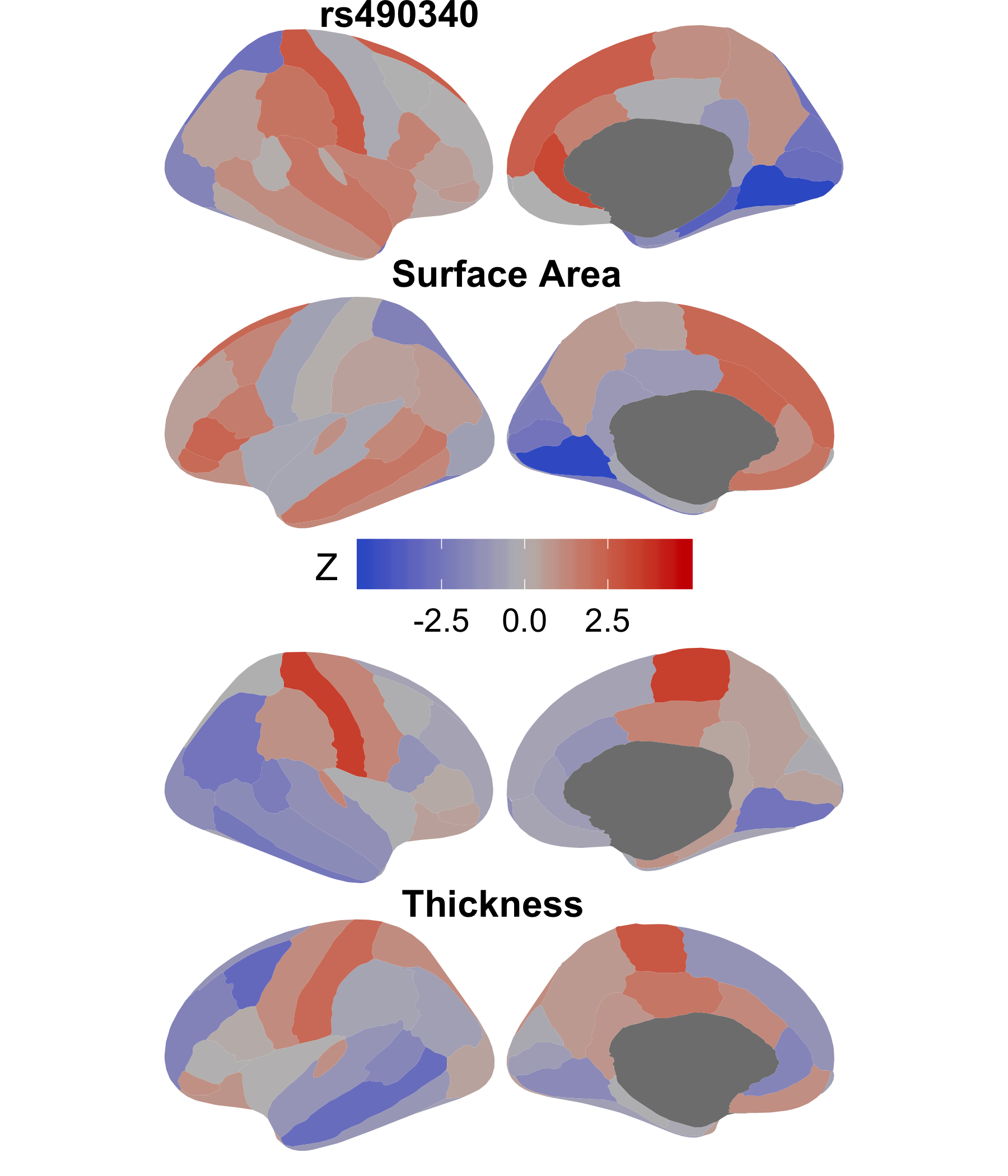

Supplement: Supplementary file 17 — Supplementary Data 14 [file 41467_2020_17368_MOESM17_ESM.gz › BrainMaps/most_aseg_vol/BrainMap038_rs490340.png]

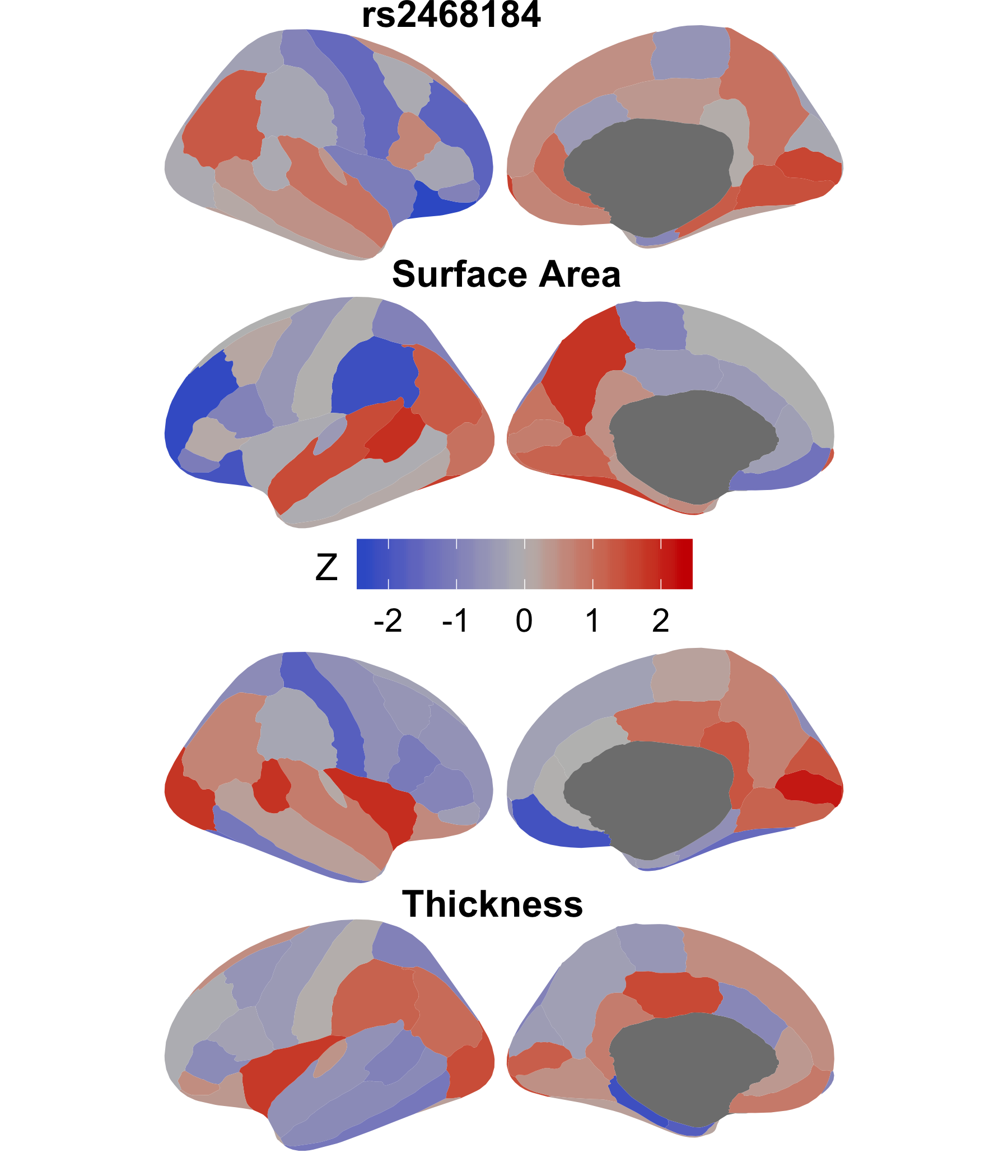

Supplement: Supplementary file 17 — Supplementary Data 14 [file 41467_2020_17368_MOESM17_ESM.gz › BrainMaps/most_aseg_vol/BrainMap129_rs2468184.png]

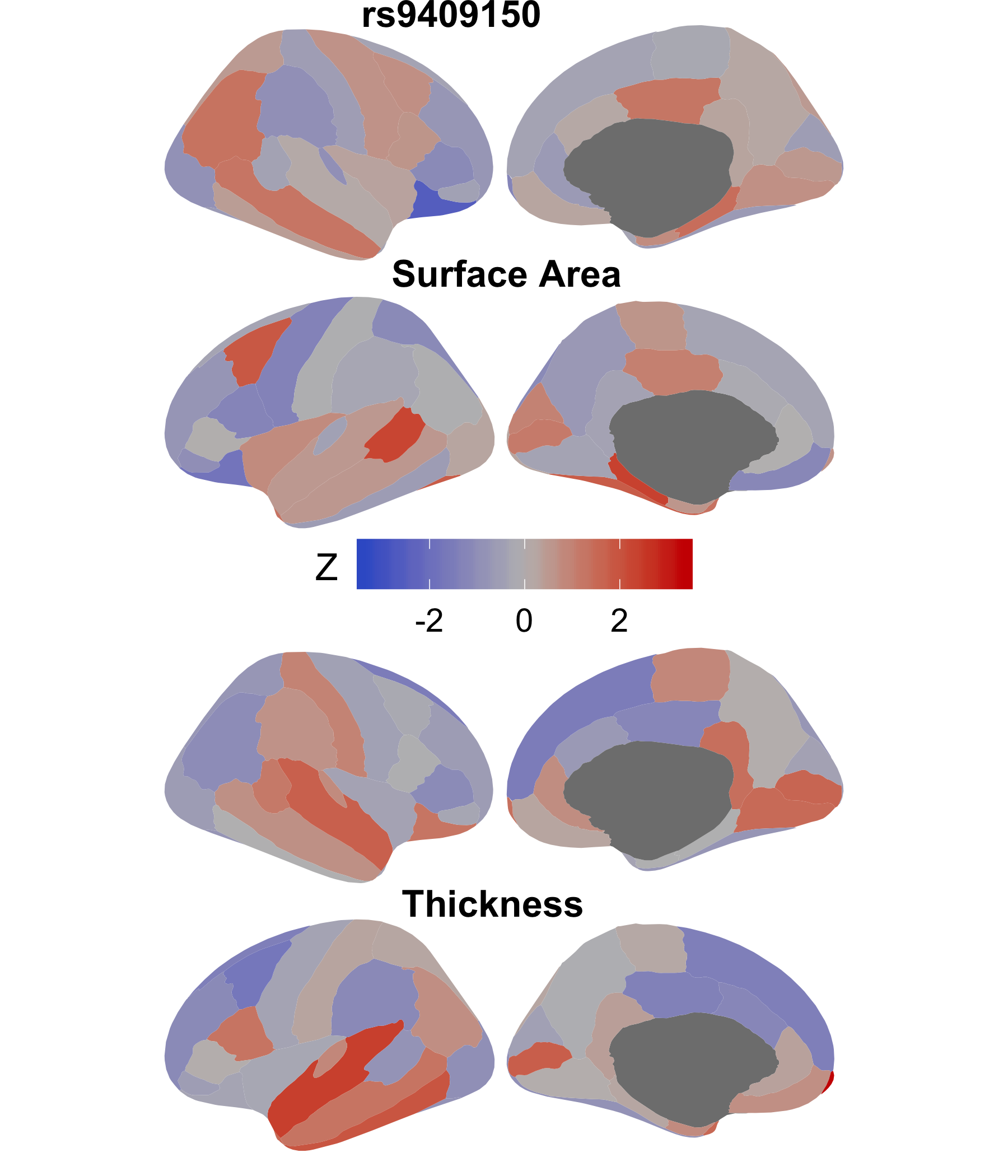

Supplement: Supplementary file 17 — Supplementary Data 14 [file 41467_2020_17368_MOESM17_ESM.gz › BrainMaps/most_aseg_vol/BrainMap089_rs9409150.png]

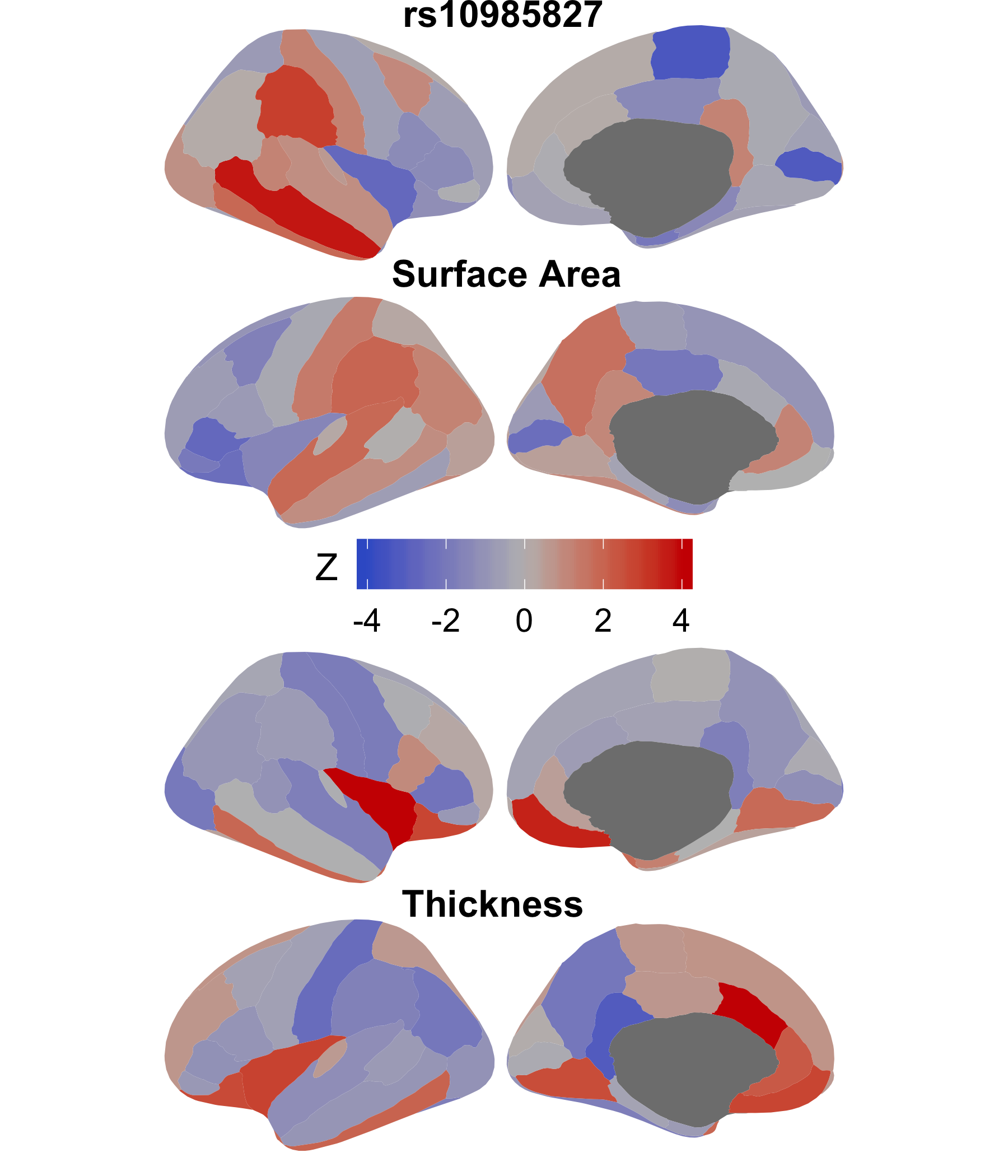

Supplement: Supplementary file 17 — Supplementary Data 14 [file 41467_2020_17368_MOESM17_ESM.gz › BrainMaps/most_aseg_vol/BrainMap103_rs10985827.png]

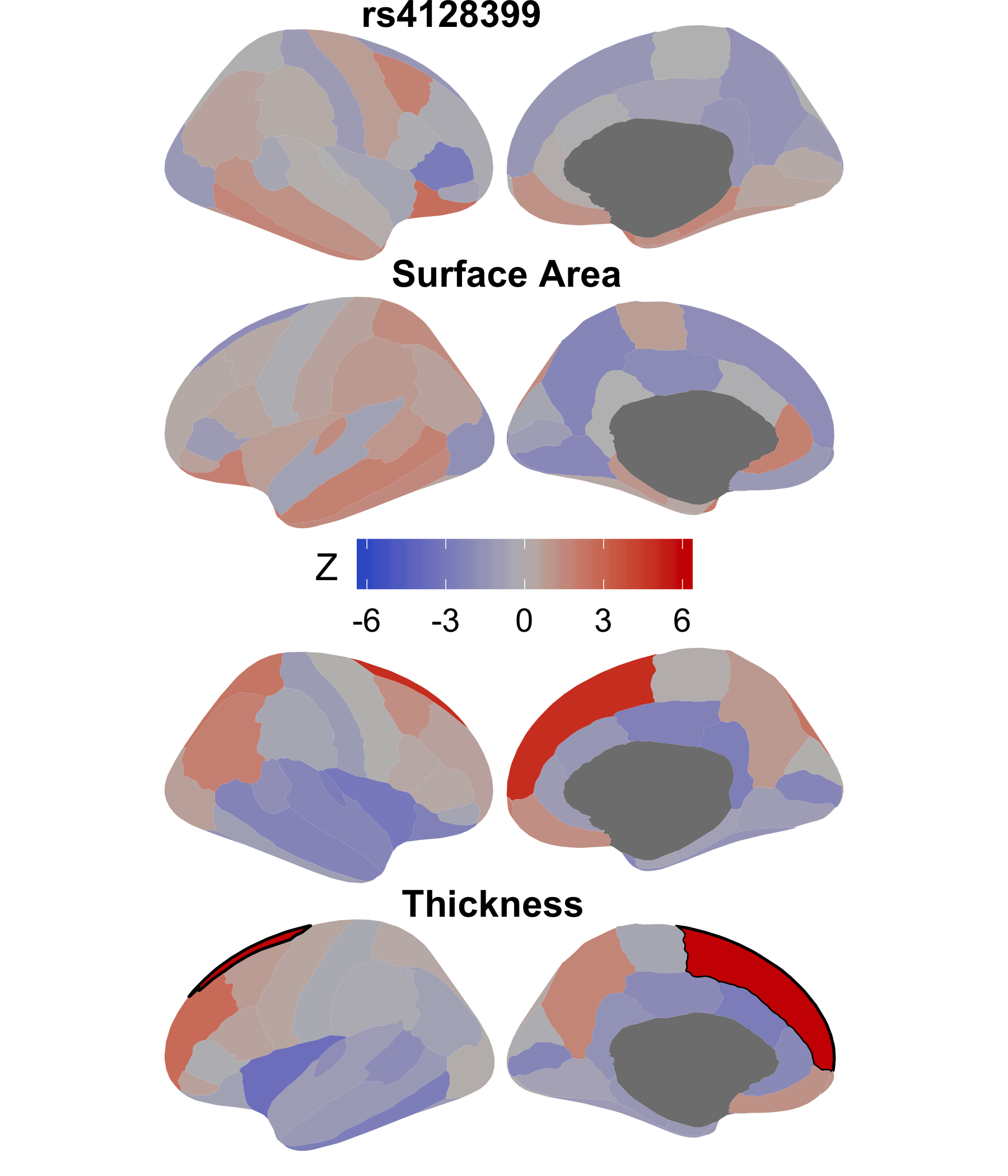

Supplement: Supplementary file 17 — Supplementary Data 14 [file 41467_2020_17368_MOESM17_ESM.gz › BrainMaps/most_aseg_vol/BrainMap052_rs4128399.png]

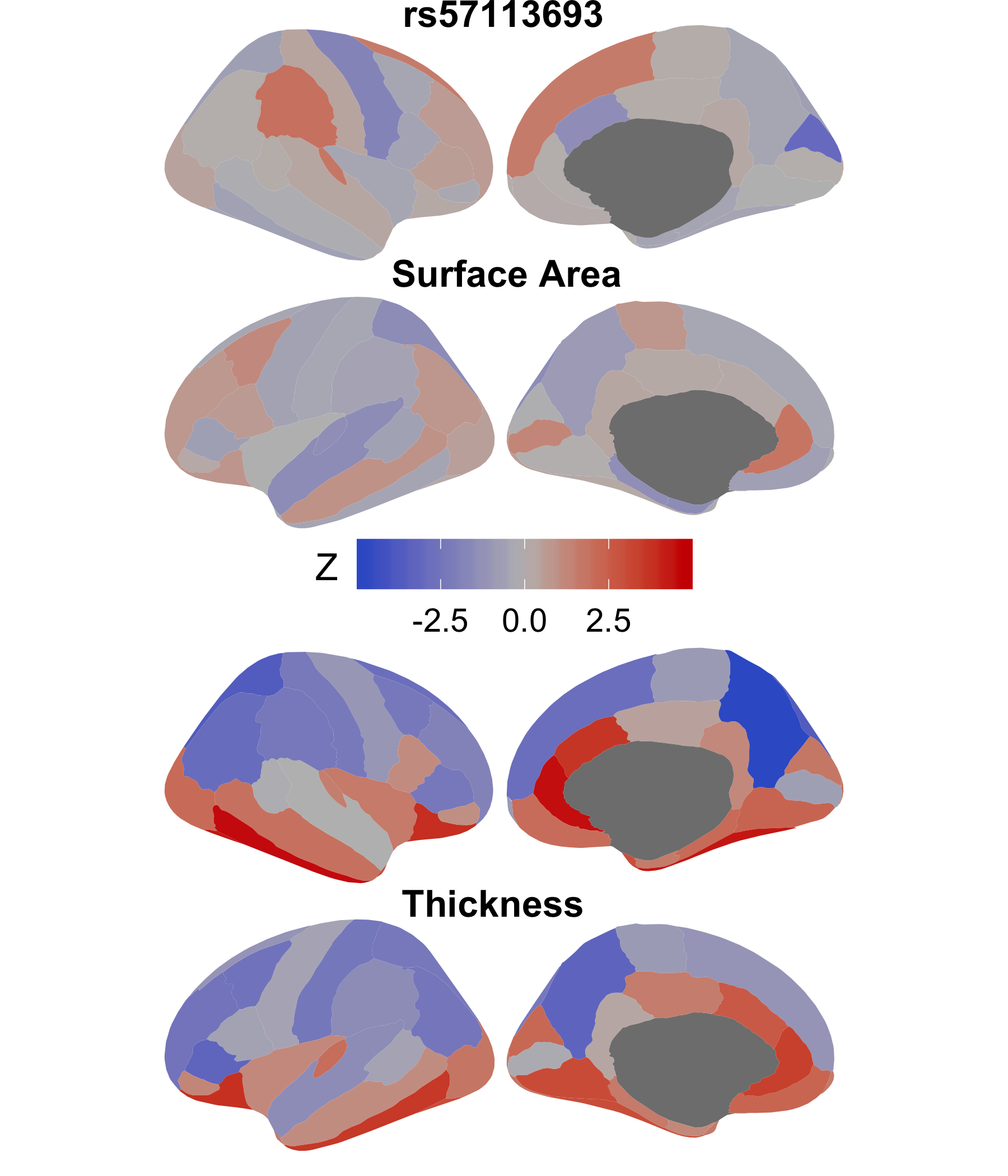

Supplement: Supplementary file 17 — Supplementary Data 14 [file 41467_2020_17368_MOESM17_ESM.gz › BrainMaps/most_aseg_vol/BrainMap046_rs57113693.png]

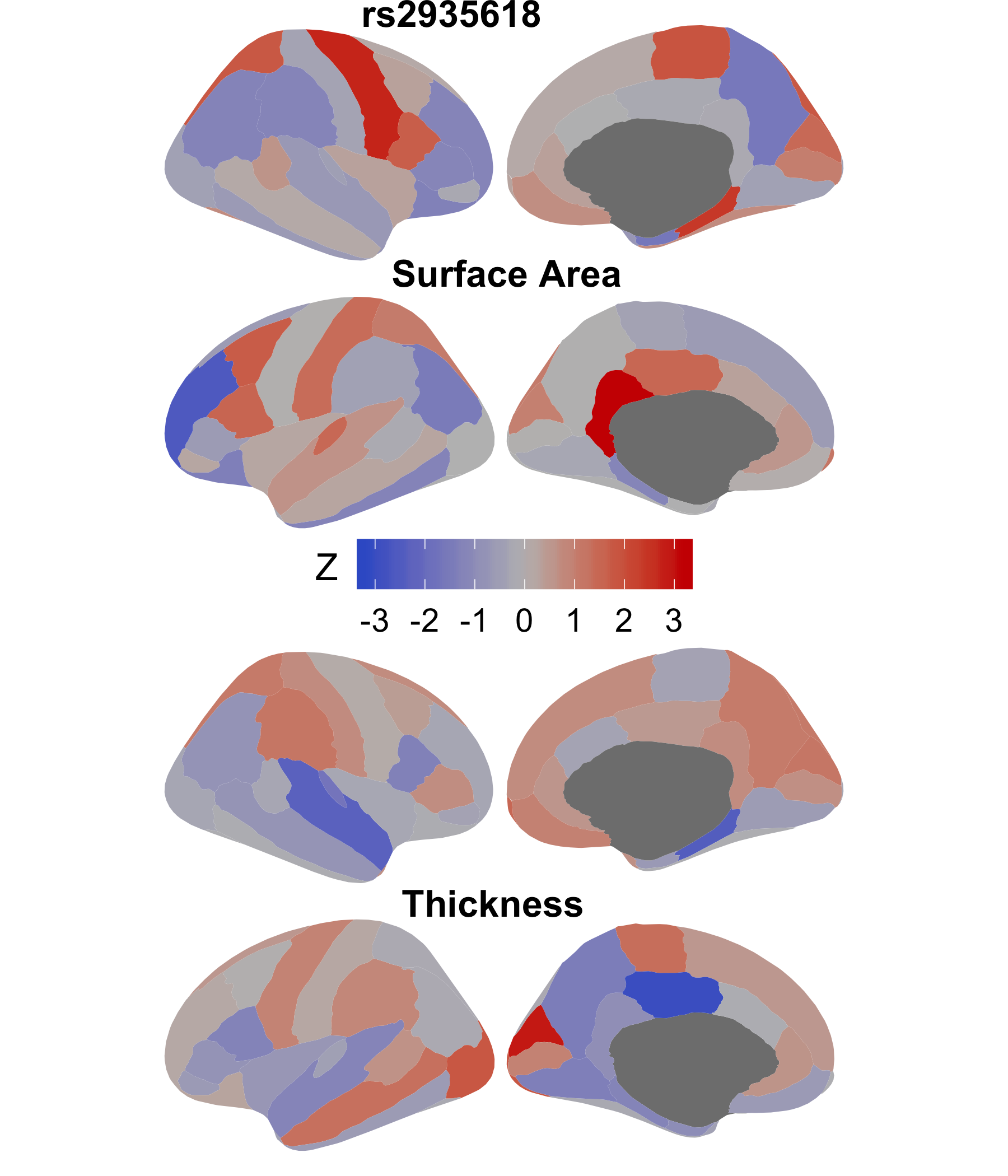

Supplement: Supplementary file 17 — Supplementary Data 14 [file 41467_2020_17368_MOESM17_ESM.gz › BrainMaps/most_aseg_vol/BrainMap167_rs2935618.png]

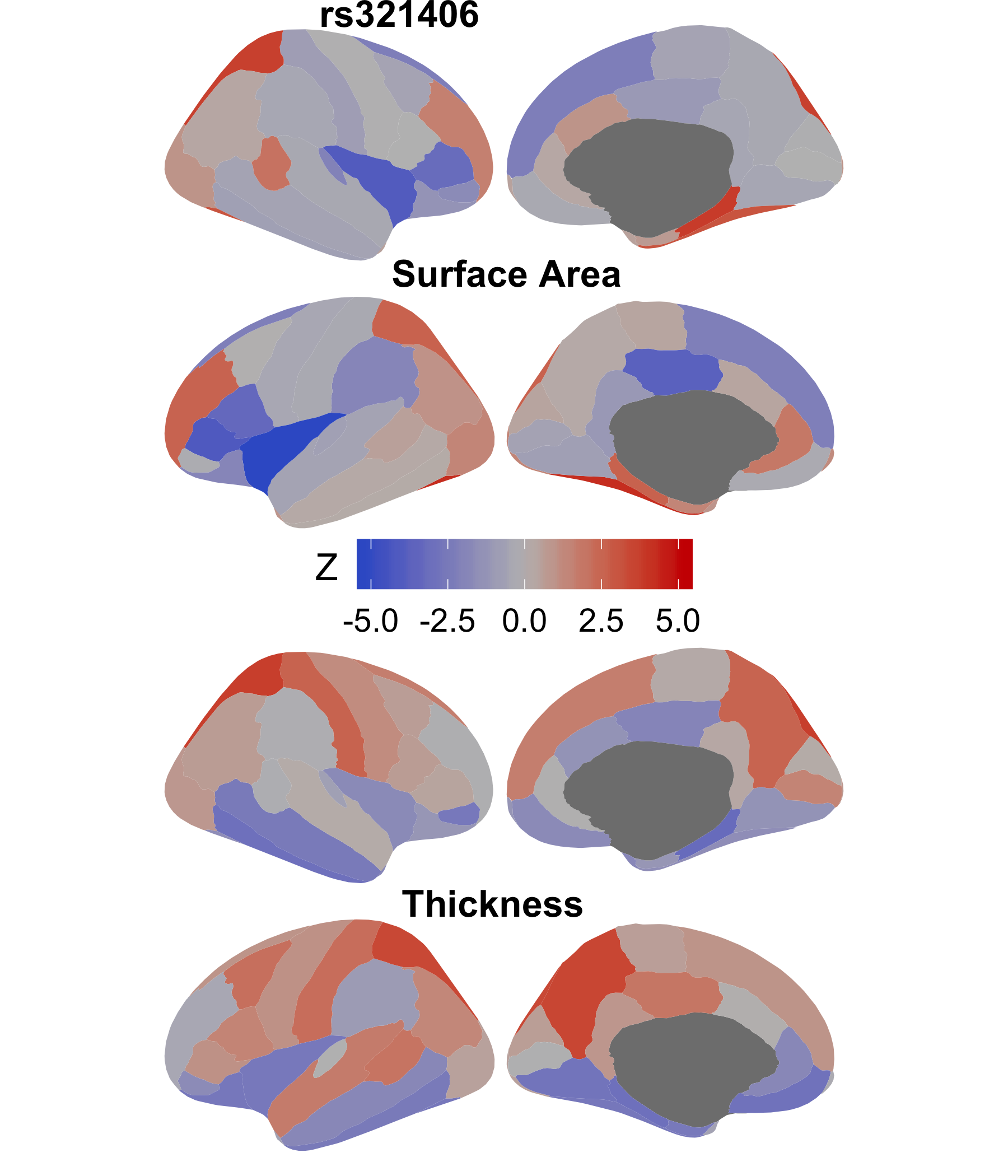

Supplement: Supplementary file 17 — Supplementary Data 14 [file 41467_2020_17368_MOESM17_ESM.gz › BrainMaps/most_aseg_vol/BrainMap057_rs321406.png]

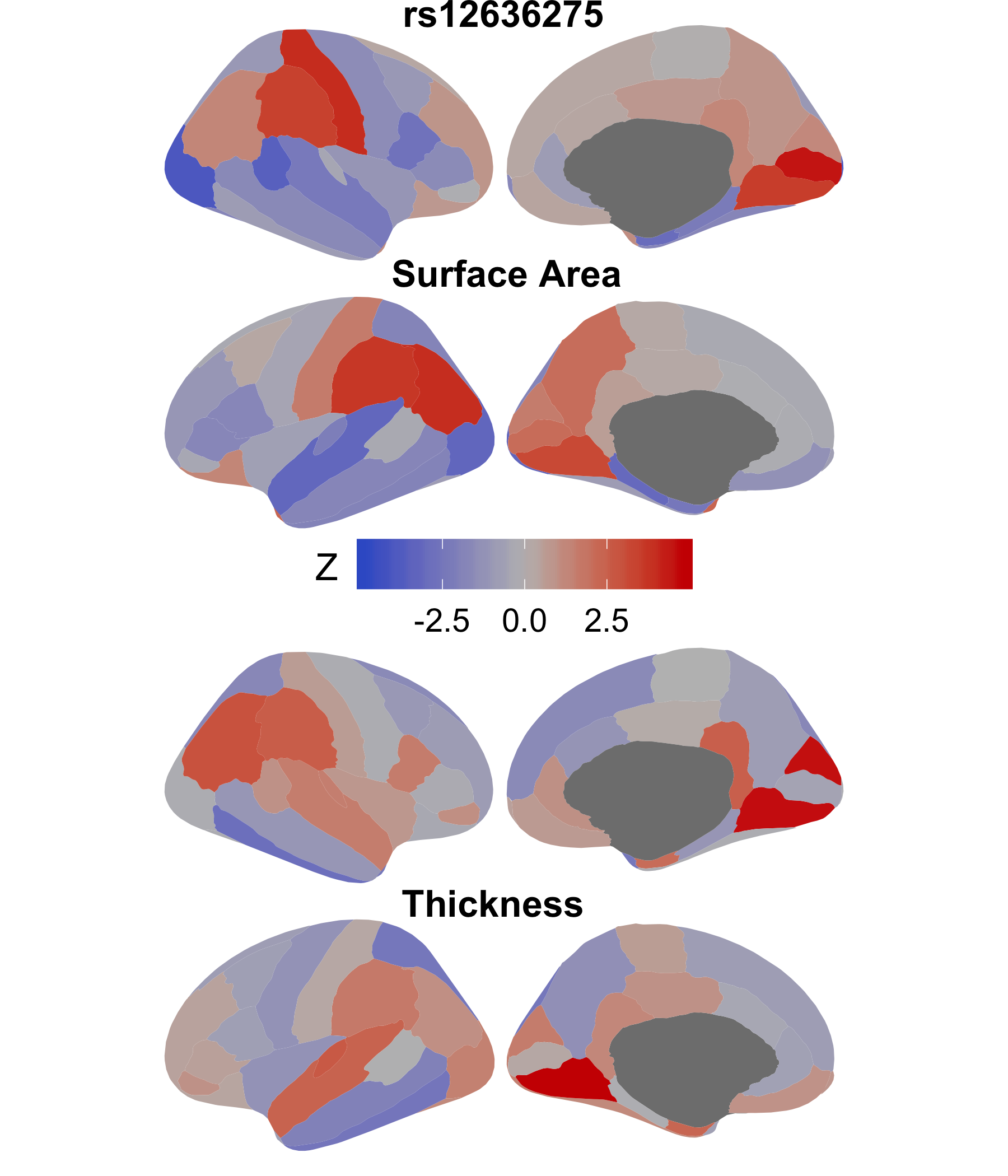

Supplement: Supplementary file 17 — Supplementary Data 14 [file 41467_2020_17368_MOESM17_ESM.gz › BrainMaps/most_aseg_vol/BrainMap026_rs12636275.png]

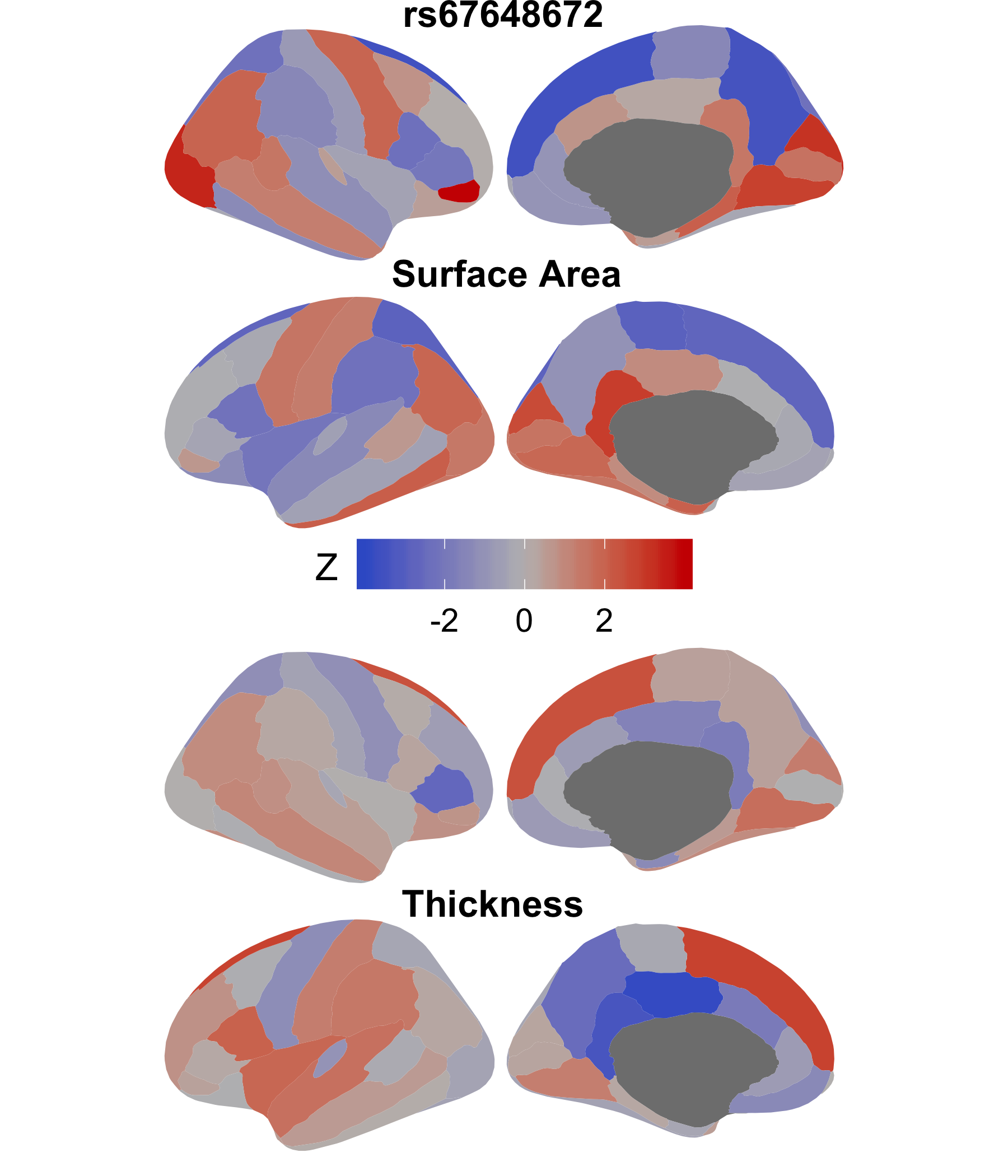

Supplement: Supplementary file 17 — Supplementary Data 14 [file 41467_2020_17368_MOESM17_ESM.gz › BrainMaps/most_aseg_vol/BrainMap059_rs67648672.png]

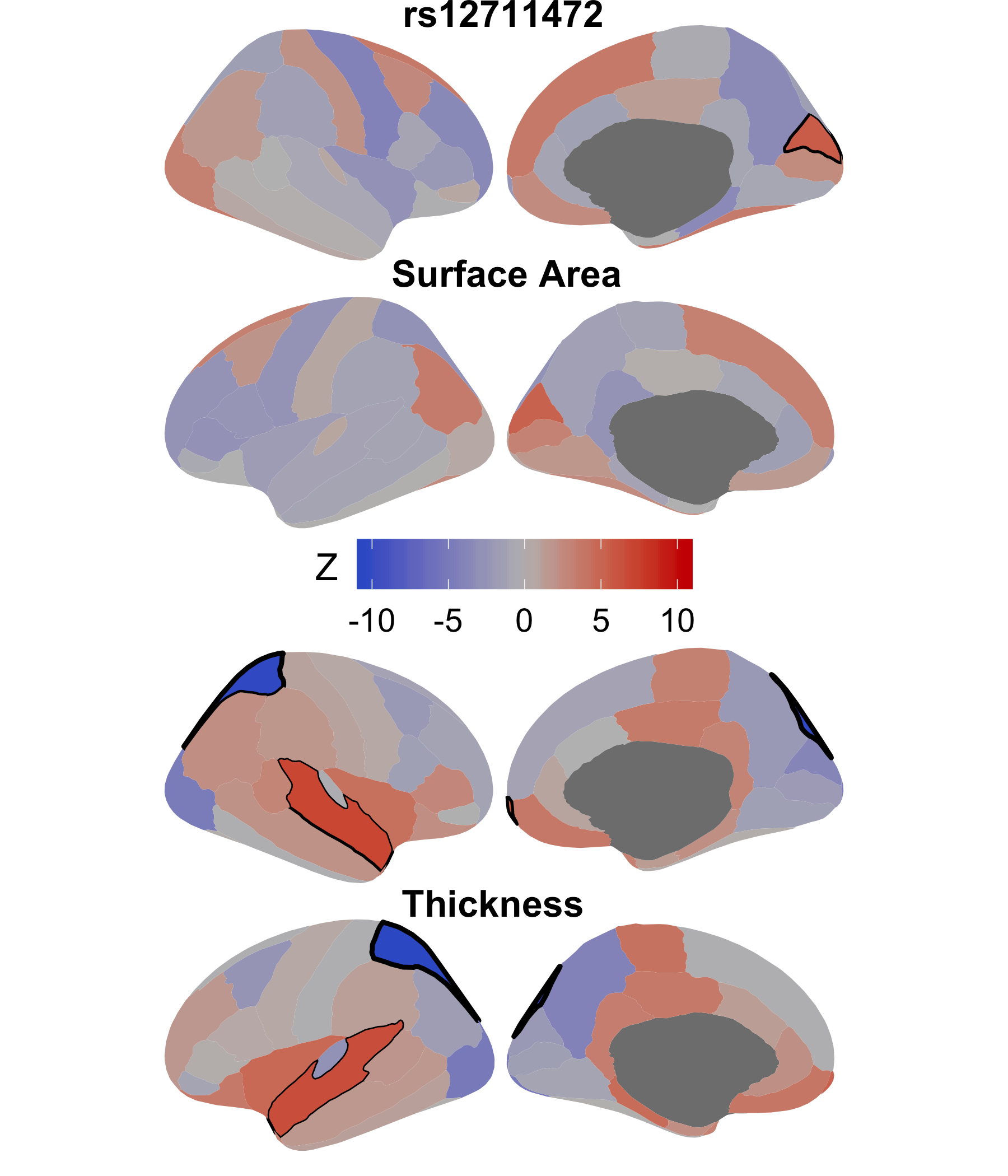

Supplement: Supplementary file 17 — Supplementary Data 14 [file 41467_2020_17368_MOESM17_ESM.gz › BrainMaps/most_aseg_vol/BrainMap016_rs12711472.png]
