# Supplementary figures and images for: Understanding the genetic determinants of the brain with MOSTest (part 1 of 2)
Source: Nat Commun. 2020 Jul 14;11:3512. doi: 10.1038/s41467-020-17368-1 (PMC7360598; doi:10.1038/s41467-020-17368-1)

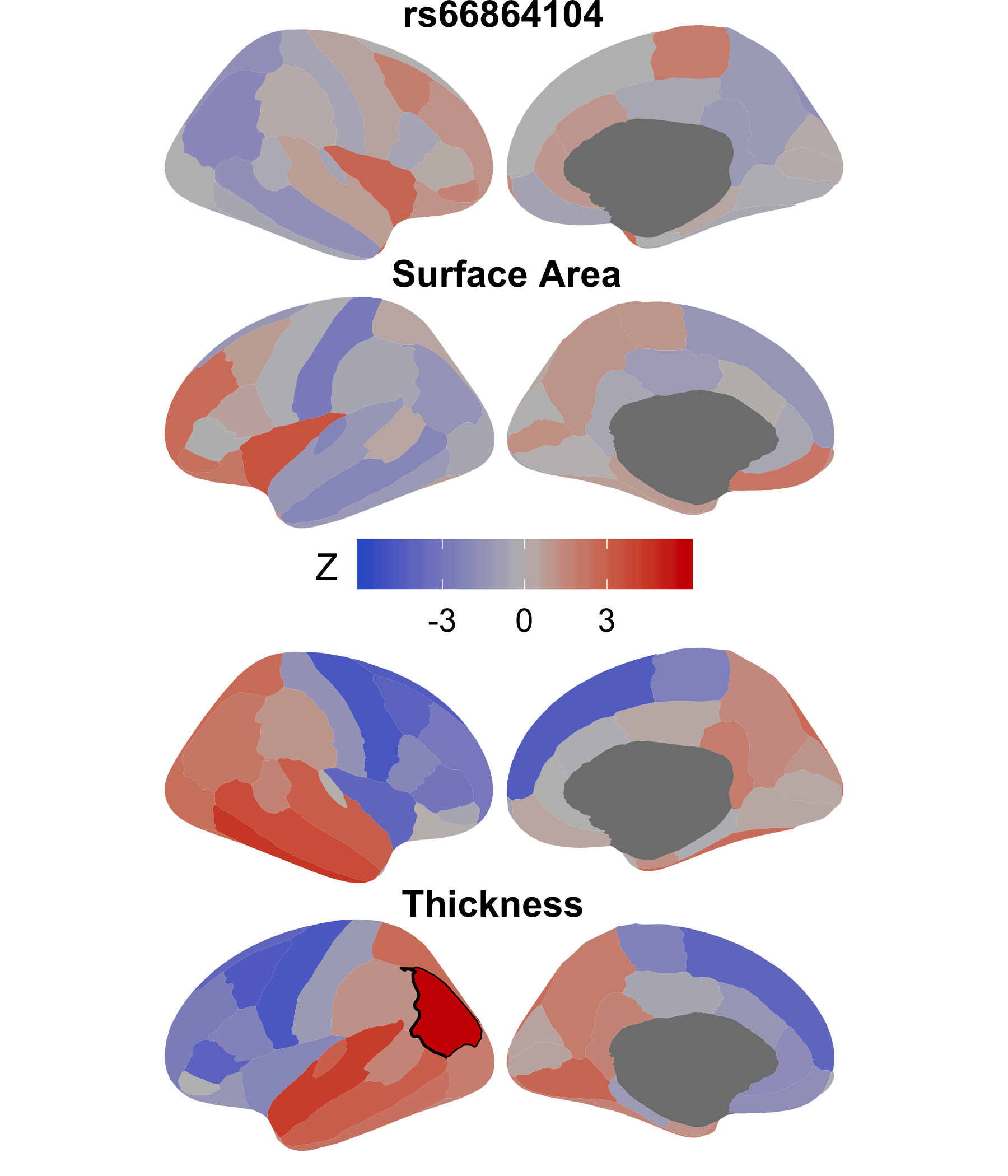

Supplement: Supplementary file 17 — Supplementary Data 14 [file 41467_2020_17368_MOESM17_ESM.gz › BrainMaps/most_dk_thick/BrainMap025_rs66864104.png]

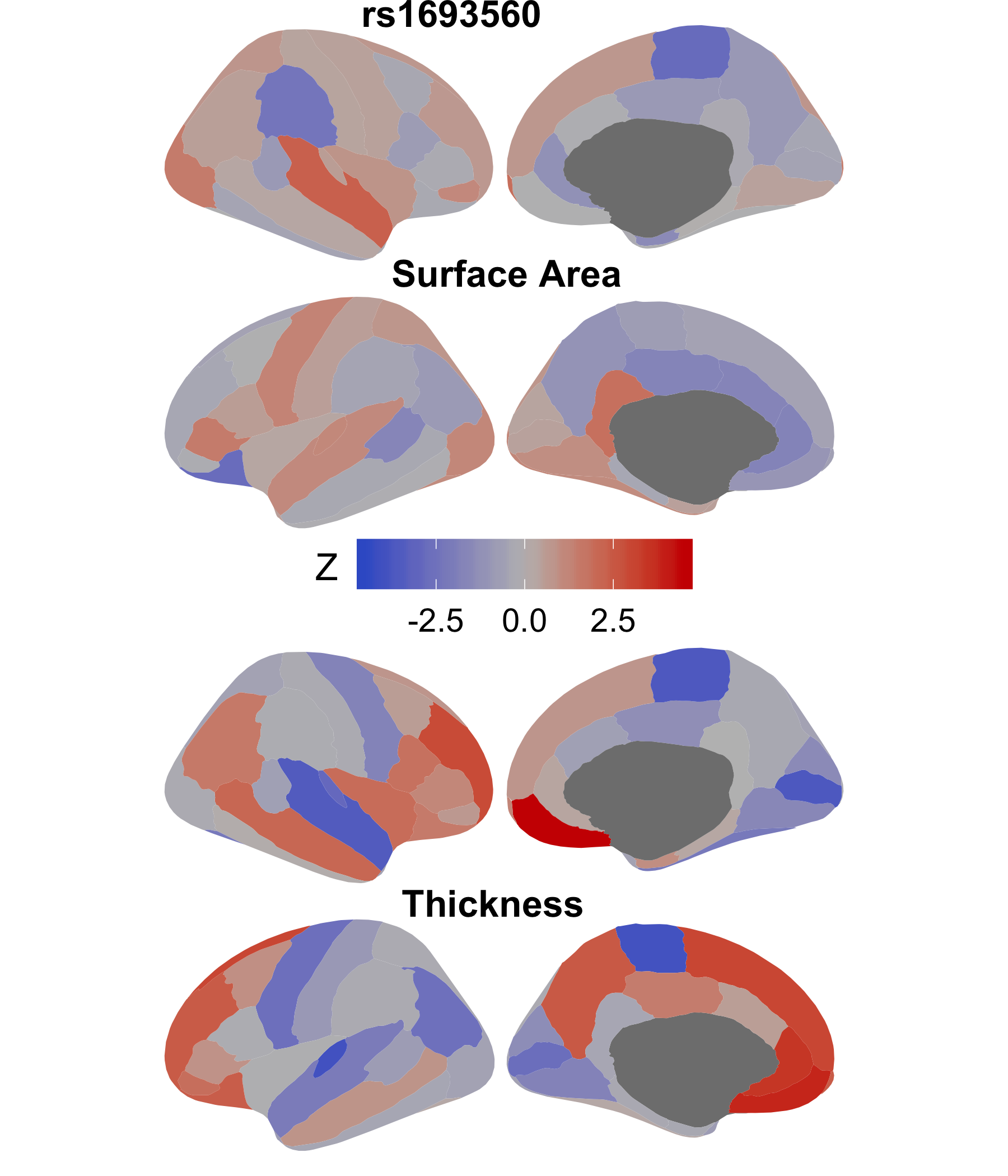

Supplement: Supplementary file 17 — Supplementary Data 14 [file 41467_2020_17368_MOESM17_ESM.gz › BrainMaps/most_dk_thick/BrainMap059_rs1693560.png]

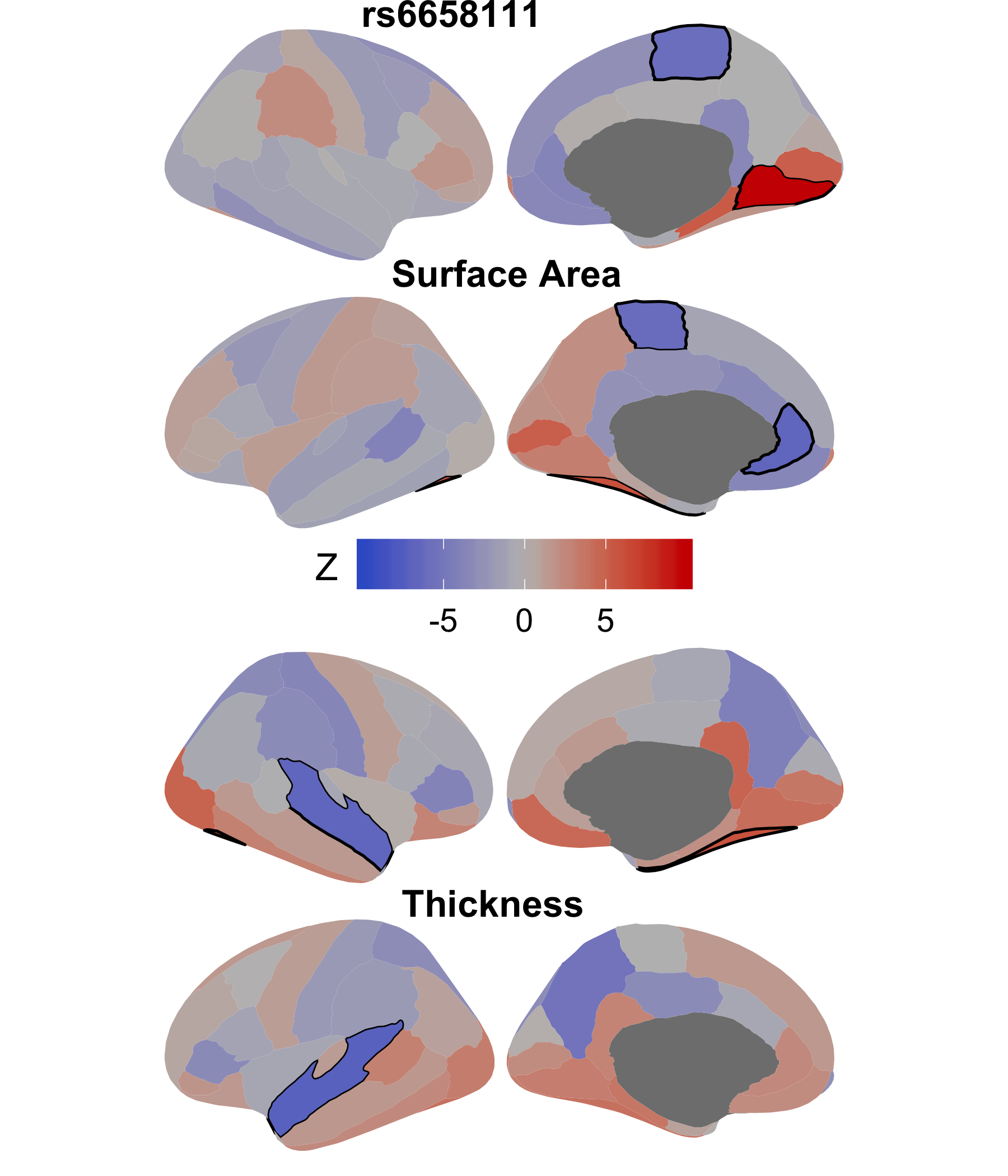

Supplement: Supplementary file 17 — Supplementary Data 14 [file 41467_2020_17368_MOESM17_ESM.gz › BrainMaps/most_dk_thick/BrainMap003_rs6658111.png]

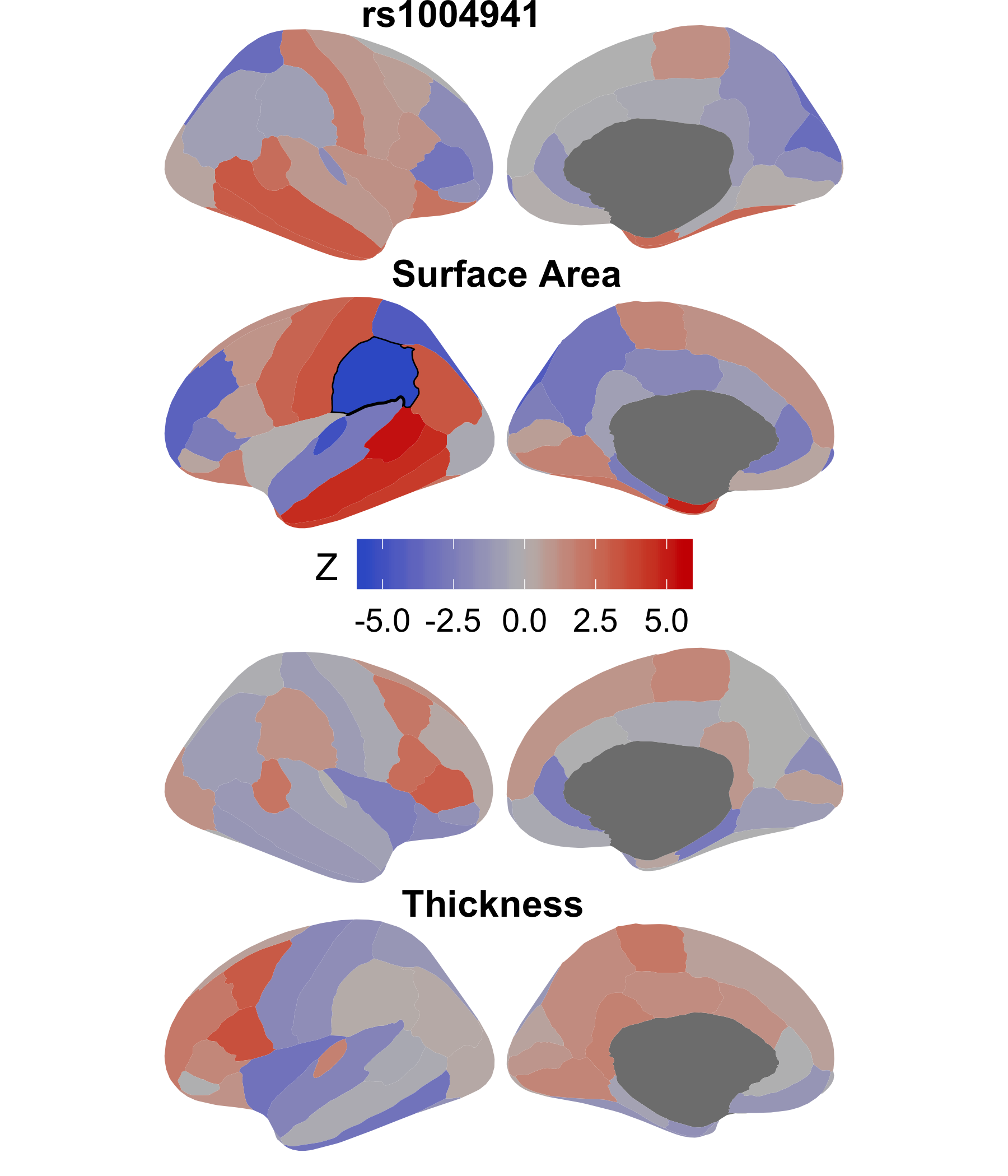

Supplement: Supplementary file 17 — Supplementary Data 14 [file 41467_2020_17368_MOESM17_ESM.gz › BrainMaps/most_dk_thick/BrainMap026_rs1004941.png]

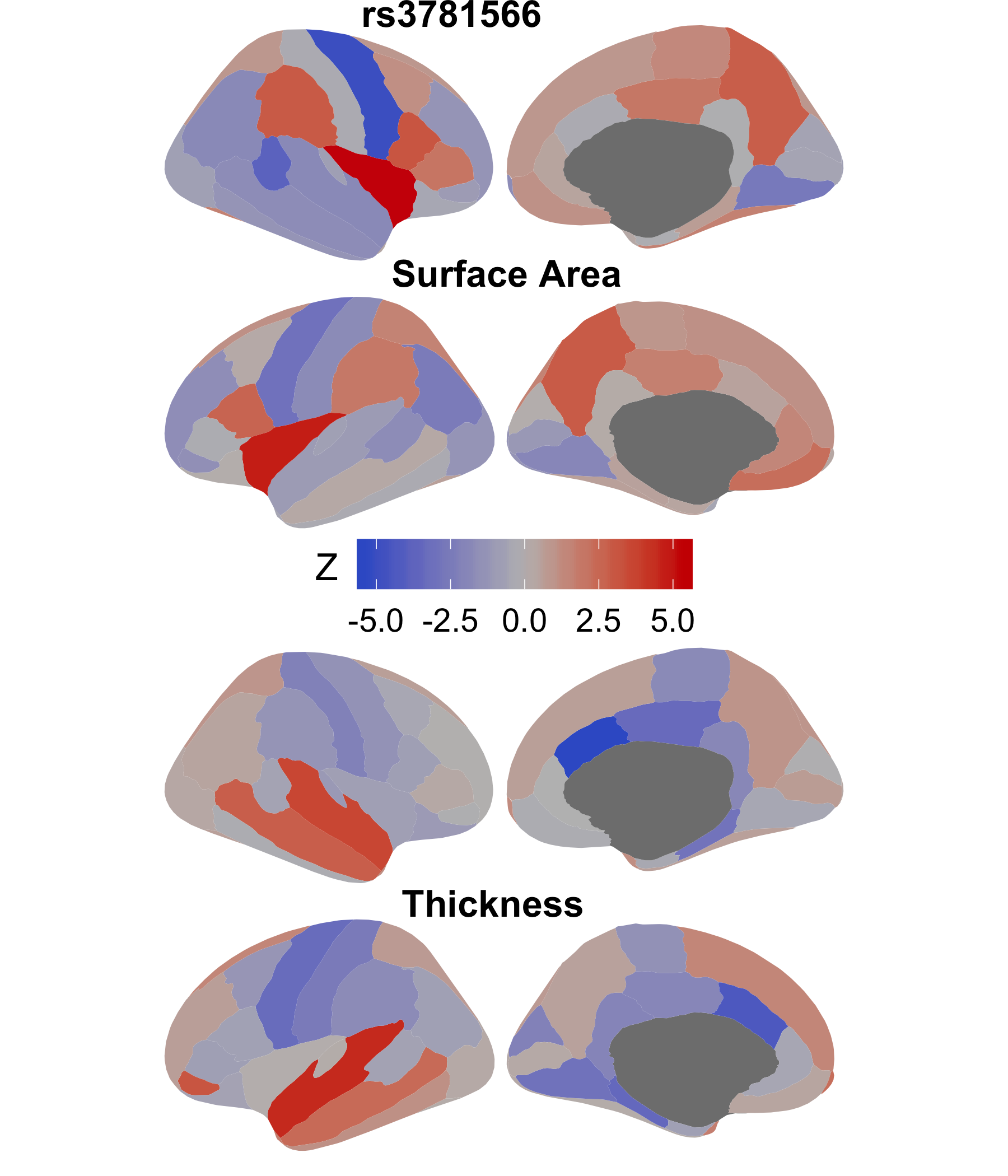

Supplement: Supplementary file 17 — Supplementary Data 14 [file 41467_2020_17368_MOESM17_ESM.gz › BrainMaps/most_dk_thick/BrainMap021_rs3781566.png]

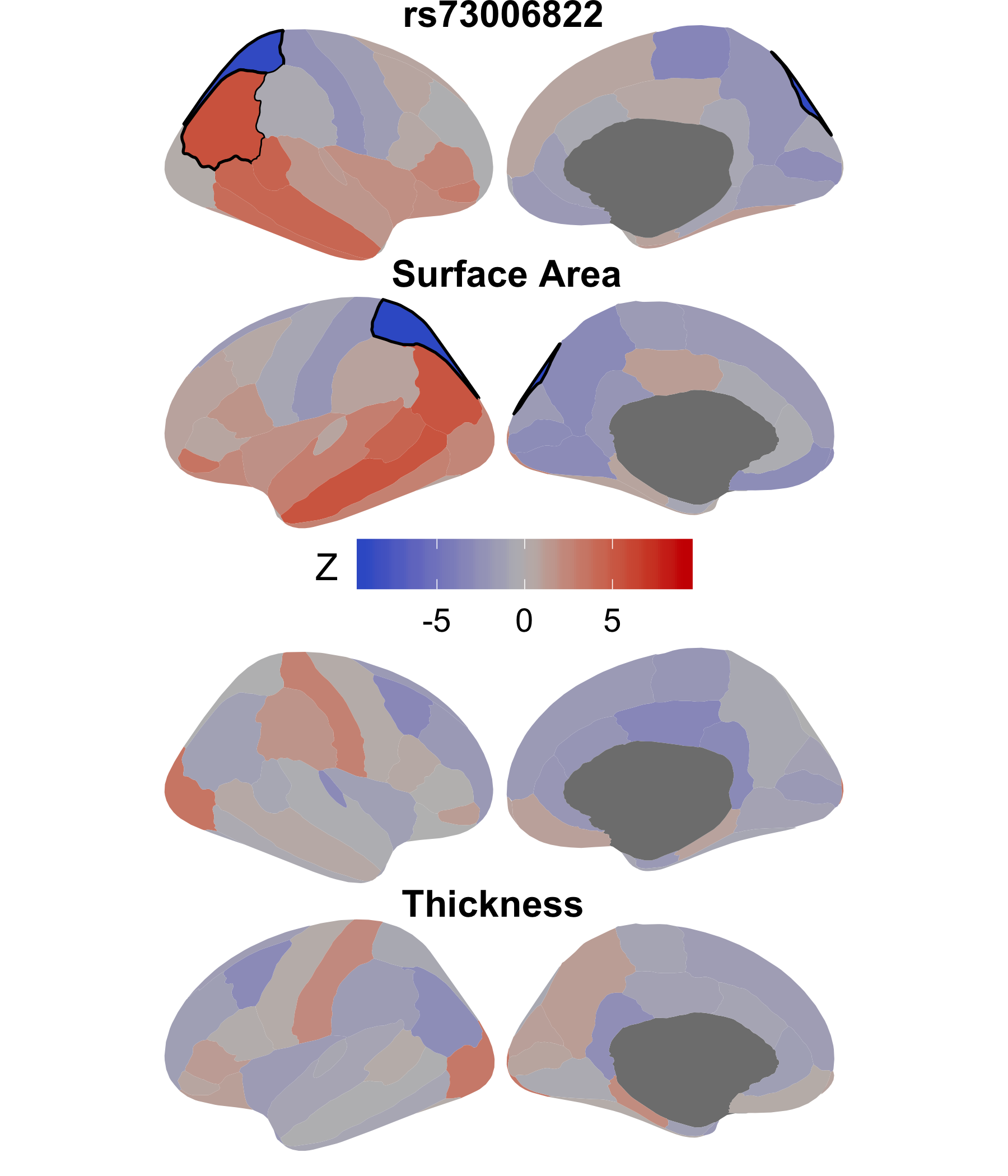

Supplement: Supplementary file 17 — Supplementary Data 14 [file 41467_2020_17368_MOESM17_ESM.gz › BrainMaps/most_dk_thick/BrainMap037_rs73006822.png]

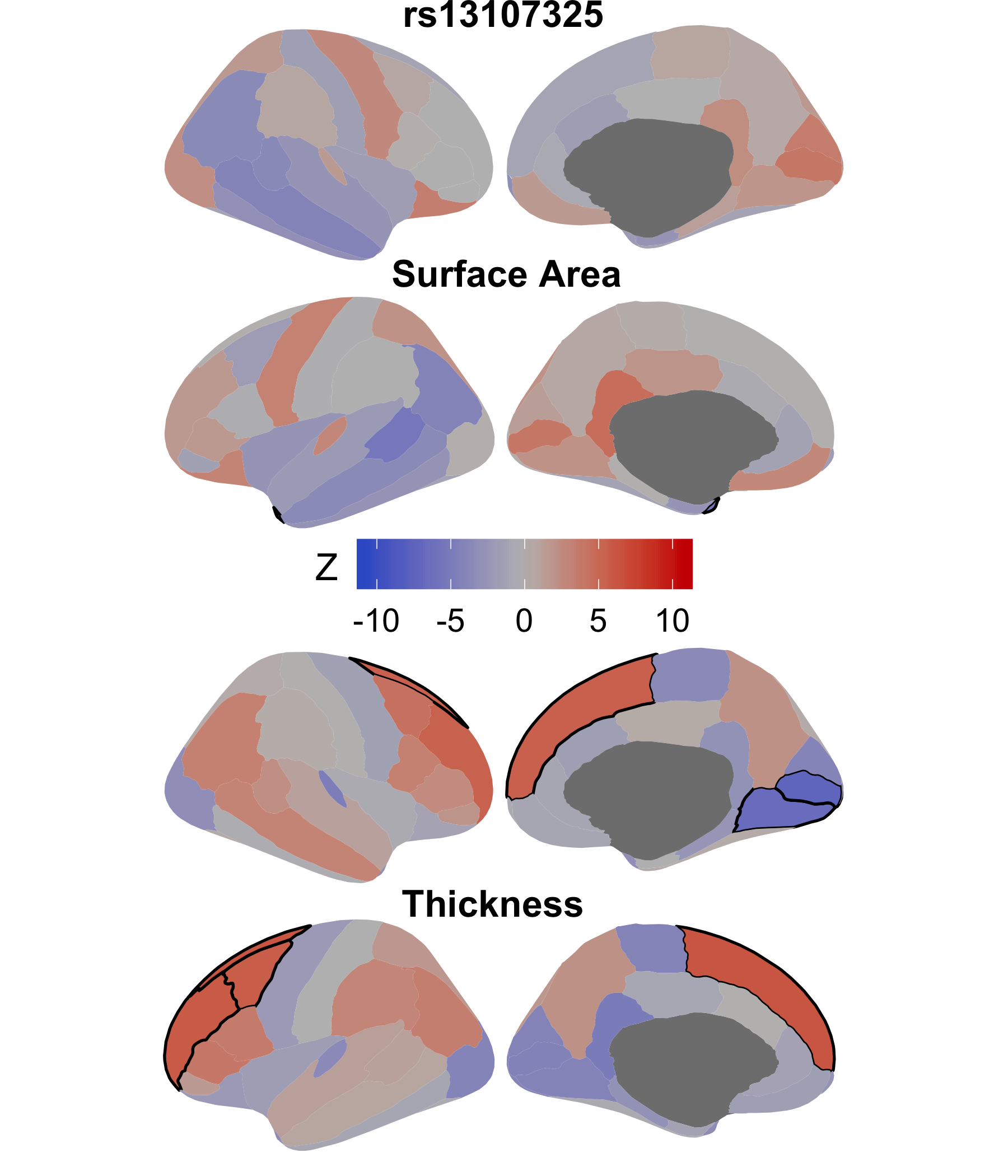

Supplement: Supplementary file 17 — Supplementary Data 14 [file 41467_2020_17368_MOESM17_ESM.gz › BrainMaps/most_dk_thick/BrainMap004_rs13107325.png]

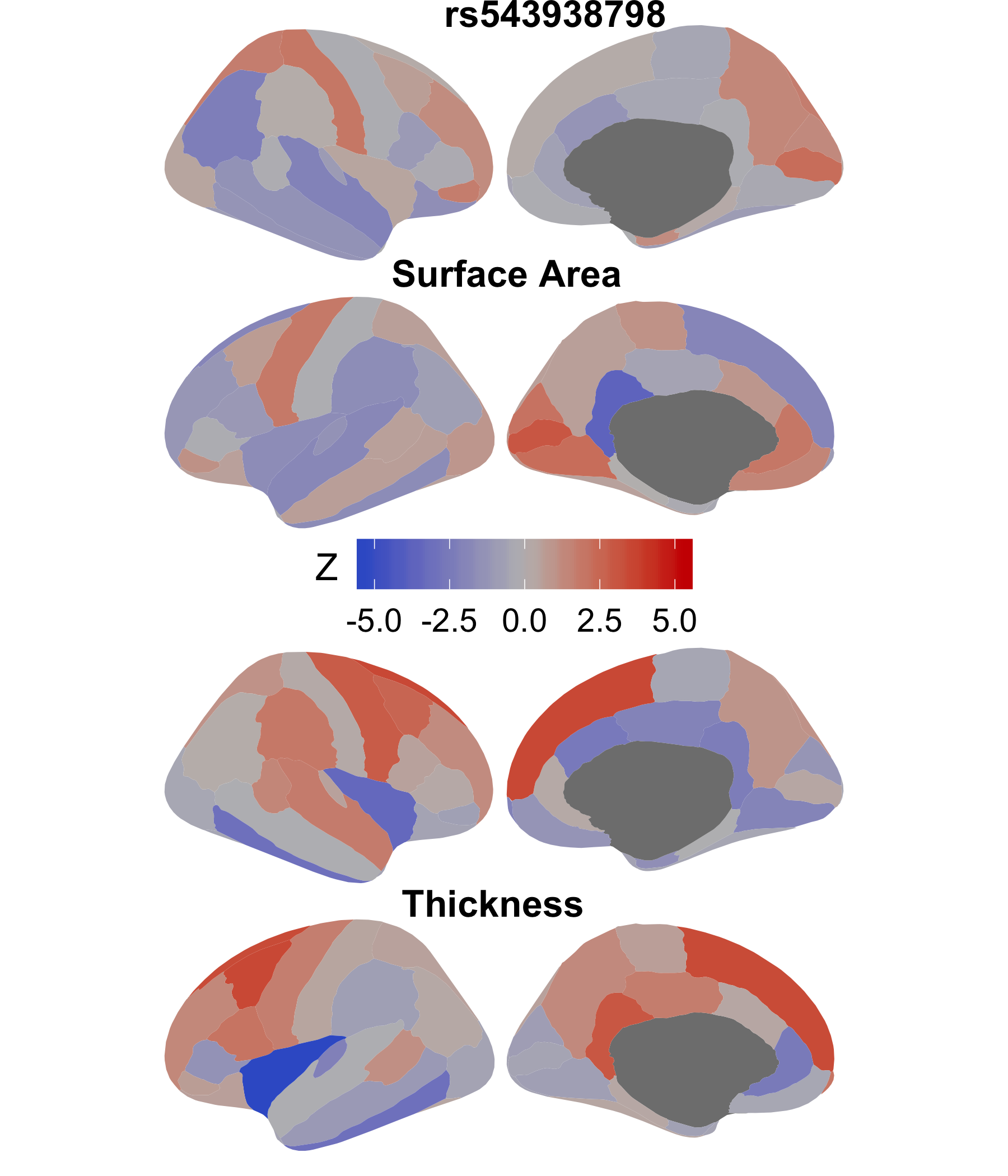

Supplement: Supplementary file 17 — Supplementary Data 14 [file 41467_2020_17368_MOESM17_ESM.gz › BrainMaps/most_dk_thick/BrainMap031_rs543938798.png]

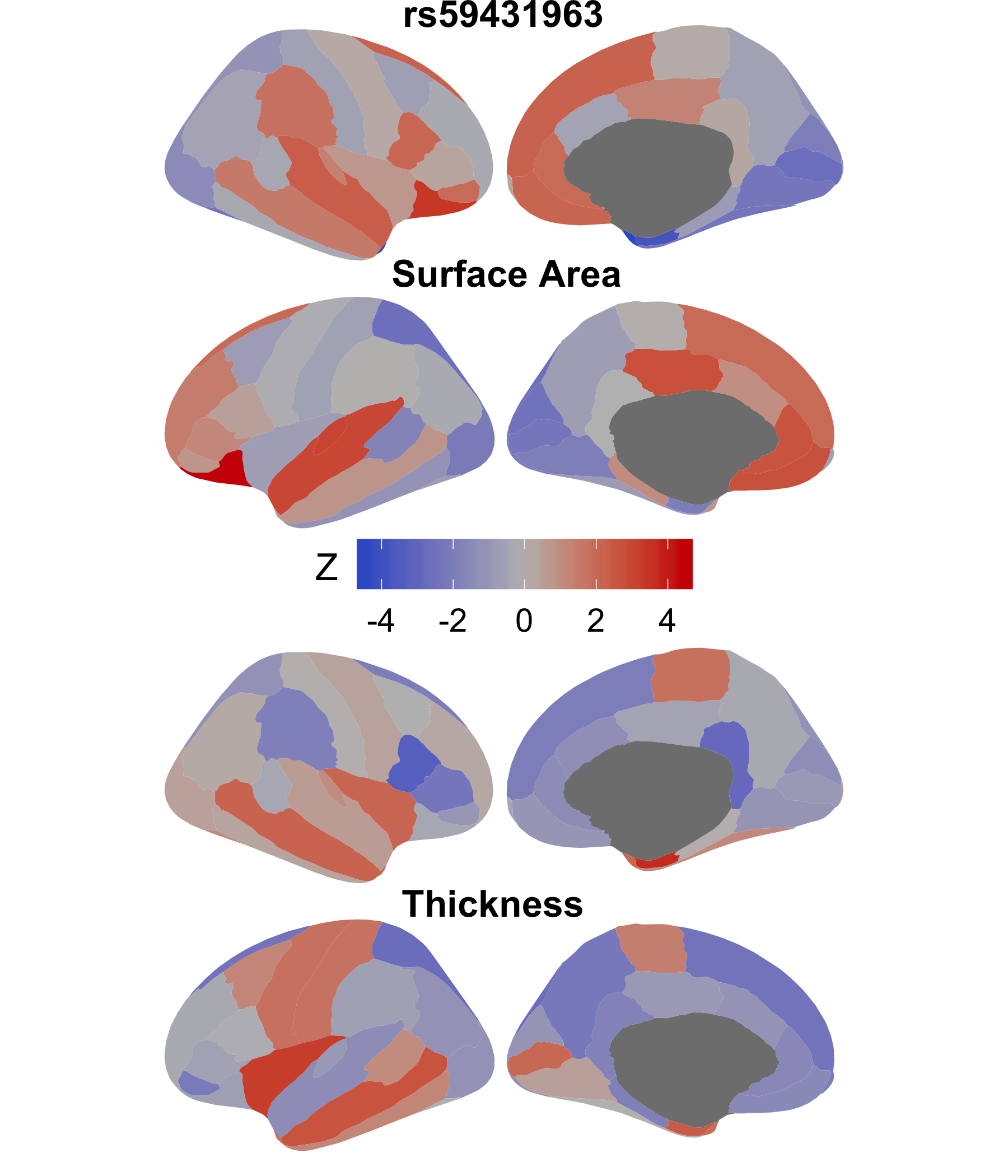

Supplement: Supplementary file 17 — Supplementary Data 14 [file 41467_2020_17368_MOESM17_ESM.gz › BrainMaps/most_dk_thick/BrainMap064_rs59431963.png]

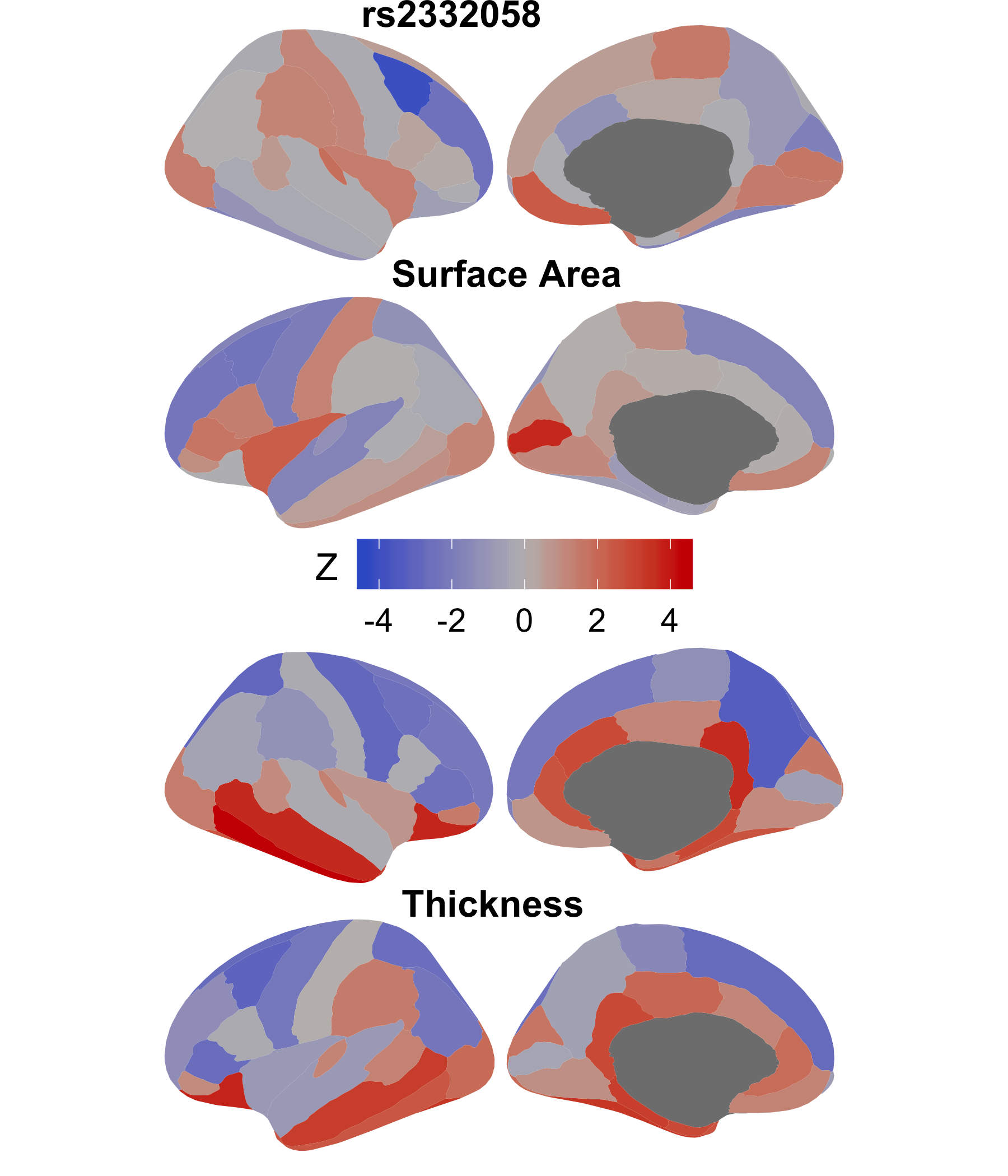

Supplement: Supplementary file 17 — Supplementary Data 14 [file 41467_2020_17368_MOESM17_ESM.gz › BrainMaps/most_dk_thick/BrainMap032_rs2332058.png]

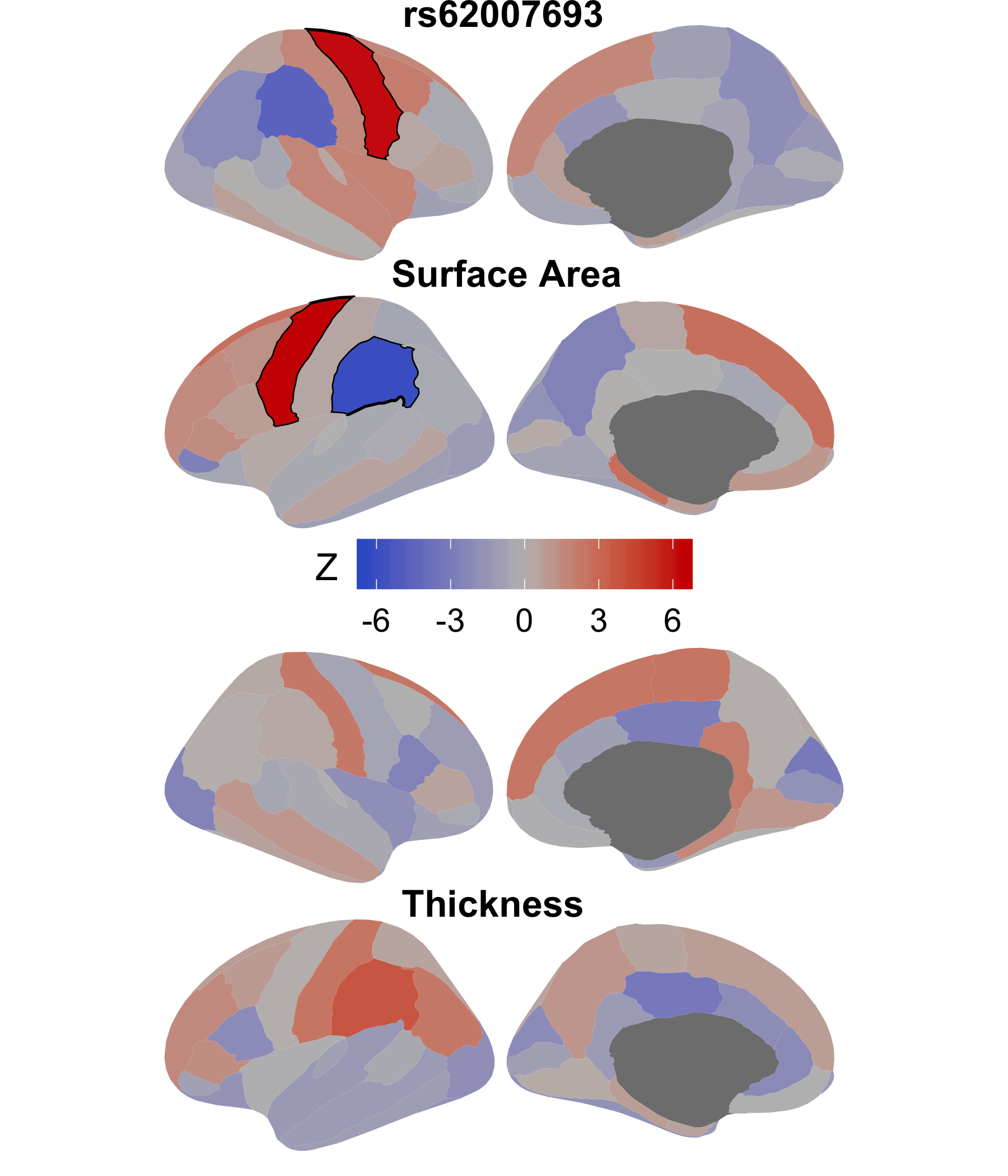

Supplement: Supplementary file 17 — Supplementary Data 14 [file 41467_2020_17368_MOESM17_ESM.gz › BrainMaps/most_dk_thick/BrainMap069_rs62007693.png]

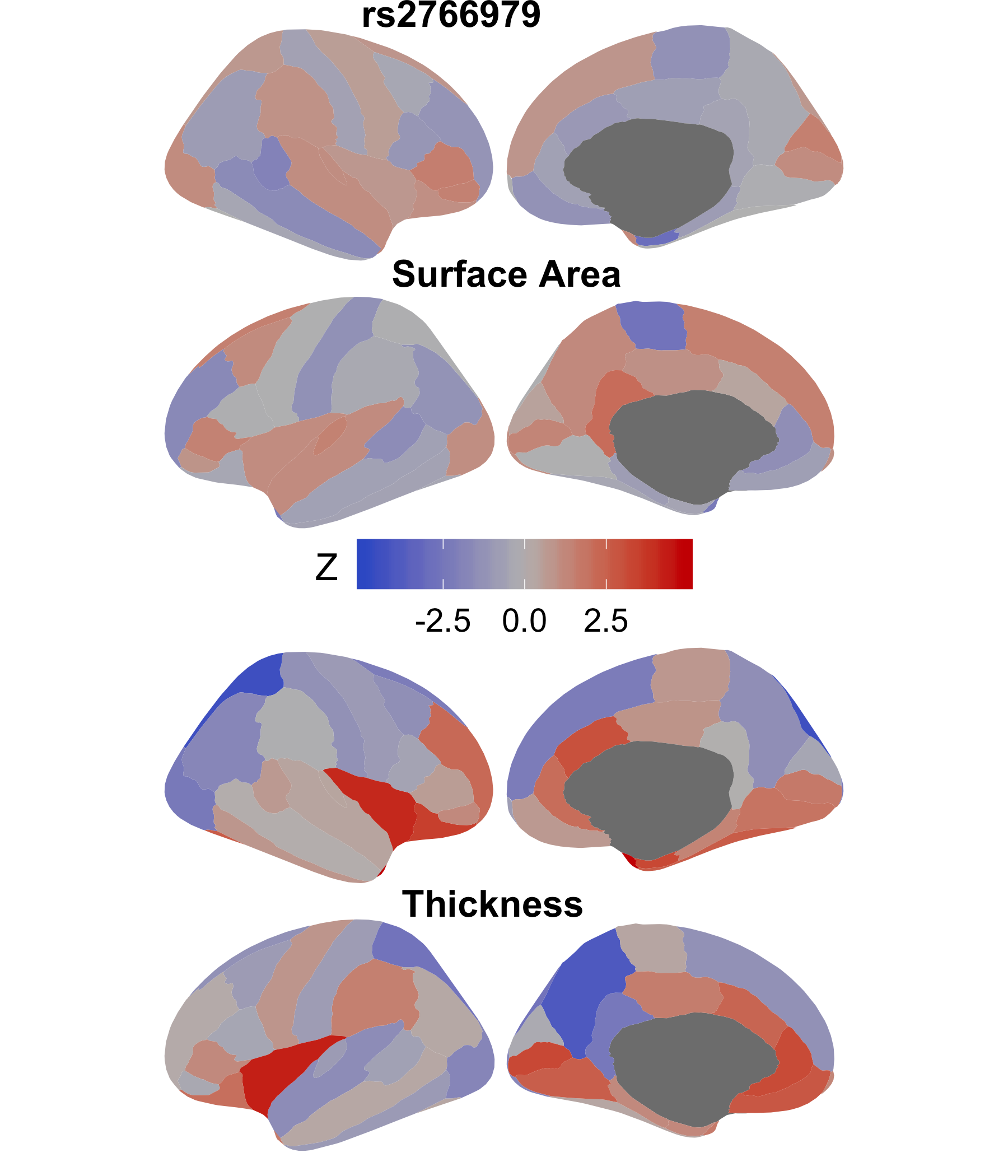

Supplement: Supplementary file 17 — Supplementary Data 14 [file 41467_2020_17368_MOESM17_ESM.gz › BrainMaps/most_dk_thick/BrainMap046_rs2766979.png]

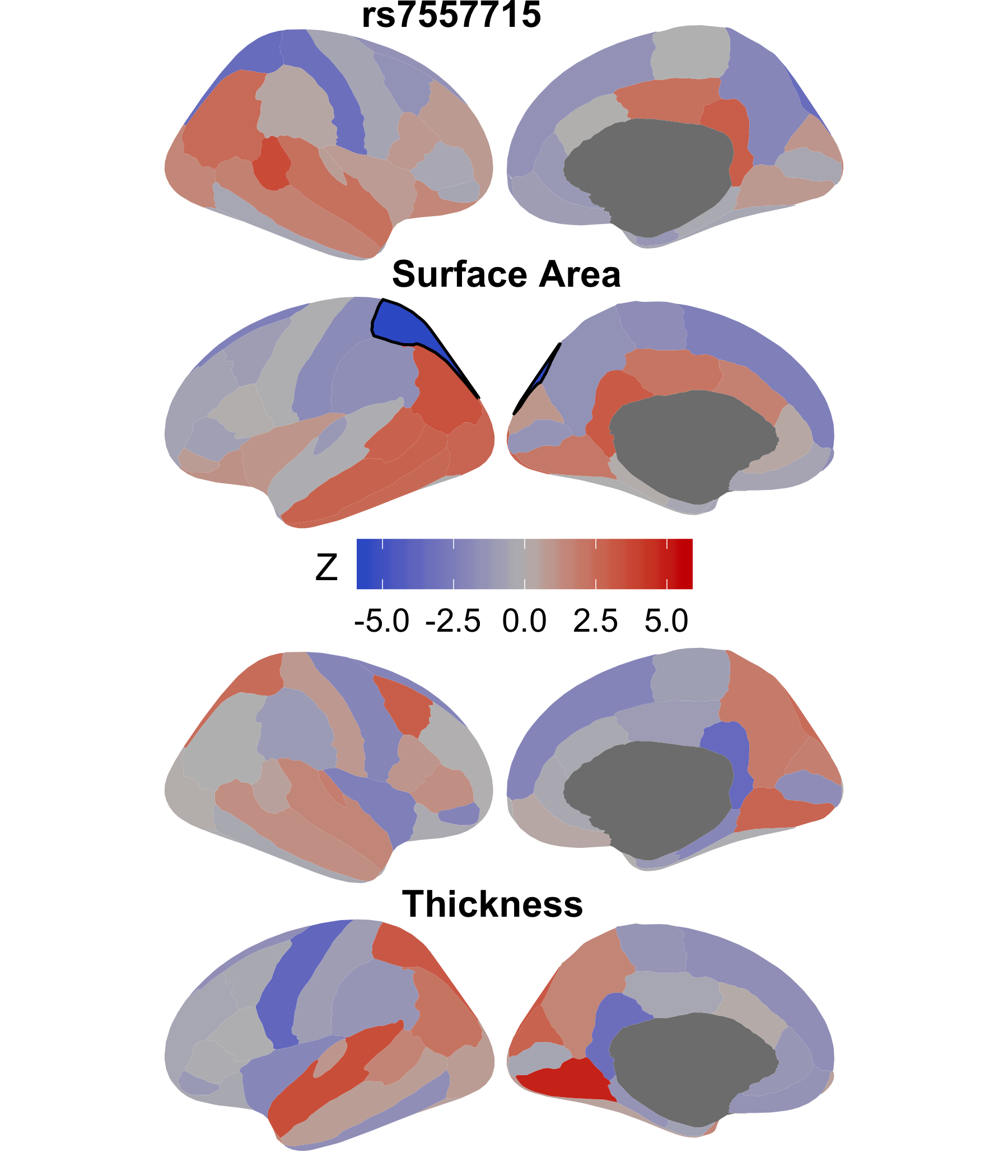

Supplement: Supplementary file 17 — Supplementary Data 14 [file 41467_2020_17368_MOESM17_ESM.gz › BrainMaps/most_dk_thick/BrainMap030_rs7557715.png]

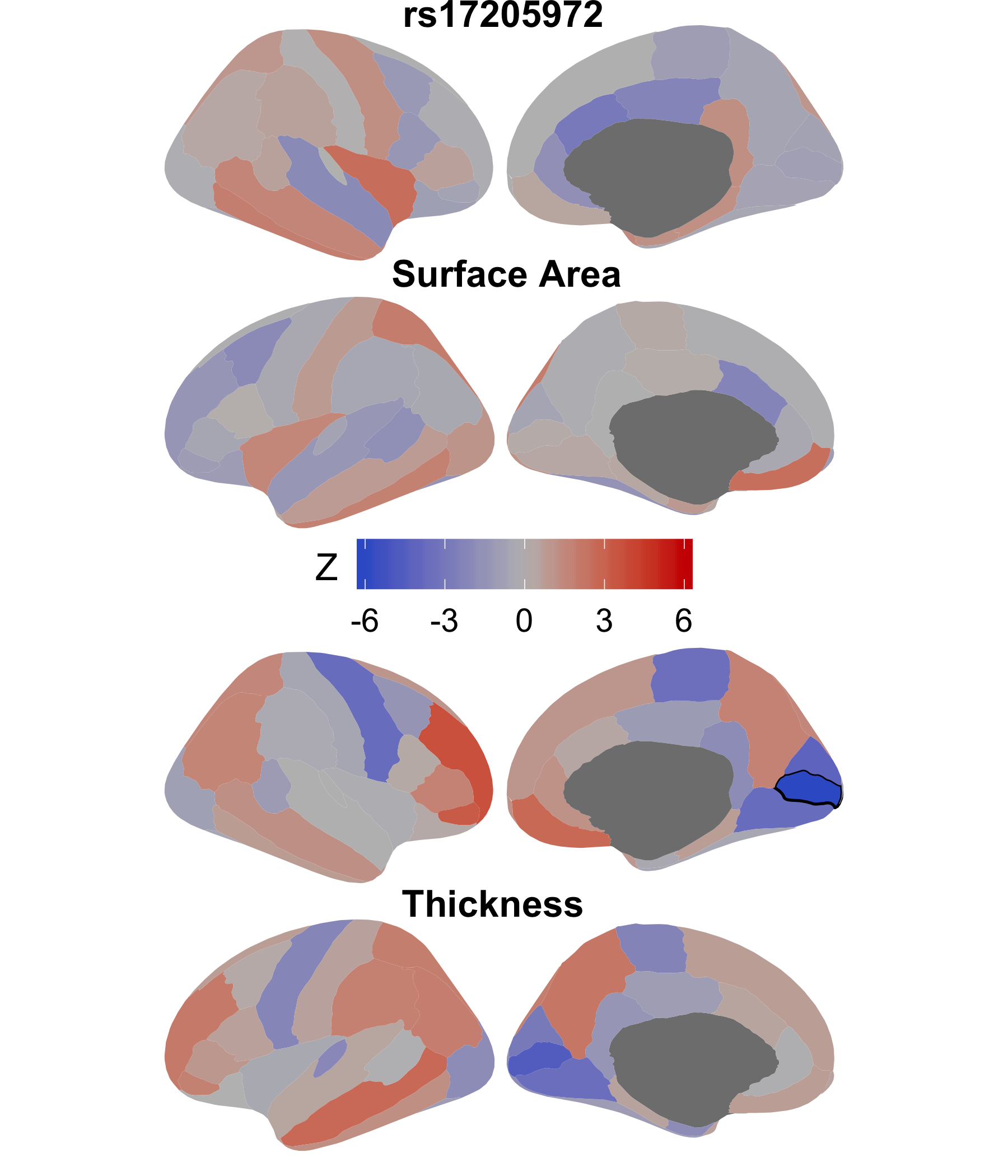

Supplement: Supplementary file 17 — Supplementary Data 14 [file 41467_2020_17368_MOESM17_ESM.gz › BrainMaps/most_dk_thick/BrainMap071_rs17205972.png]

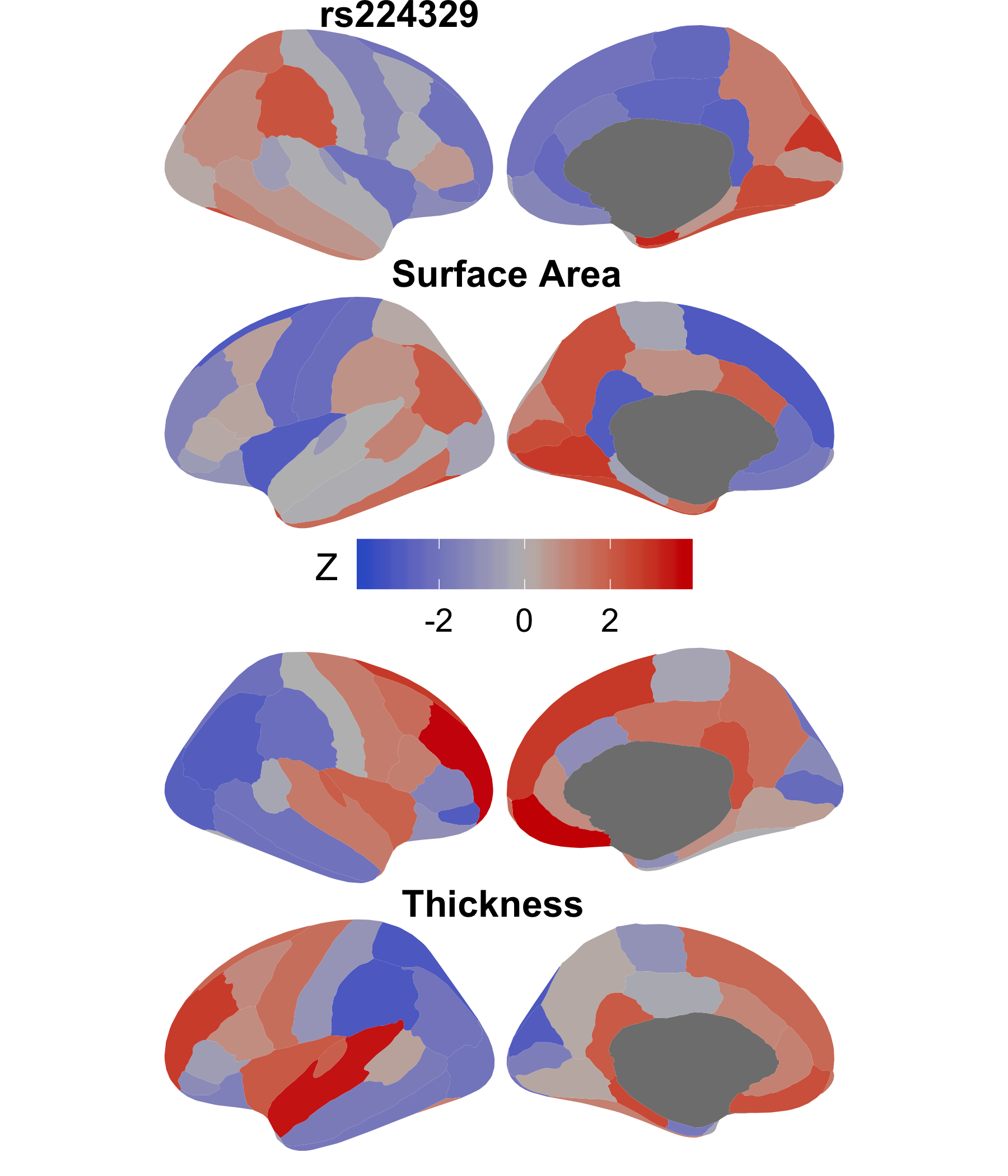

Supplement: Supplementary file 17 — Supplementary Data 14 [file 41467_2020_17368_MOESM17_ESM.gz › BrainMaps/most_dk_thick/BrainMap027_rs224329.png]

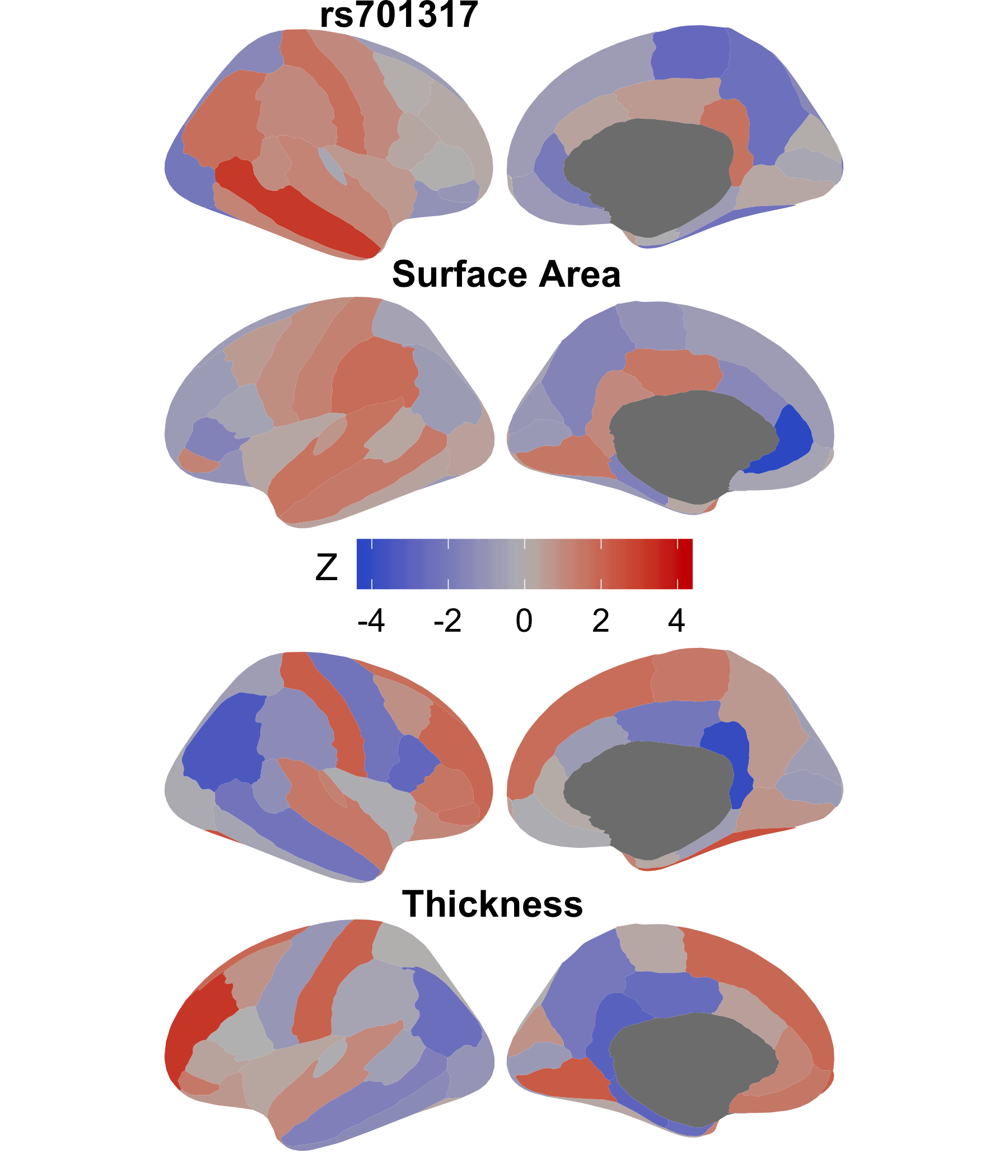

Supplement: Supplementary file 17 — Supplementary Data 14 [file 41467_2020_17368_MOESM17_ESM.gz › BrainMaps/most_dk_thick/BrainMap061_rs701317.png]

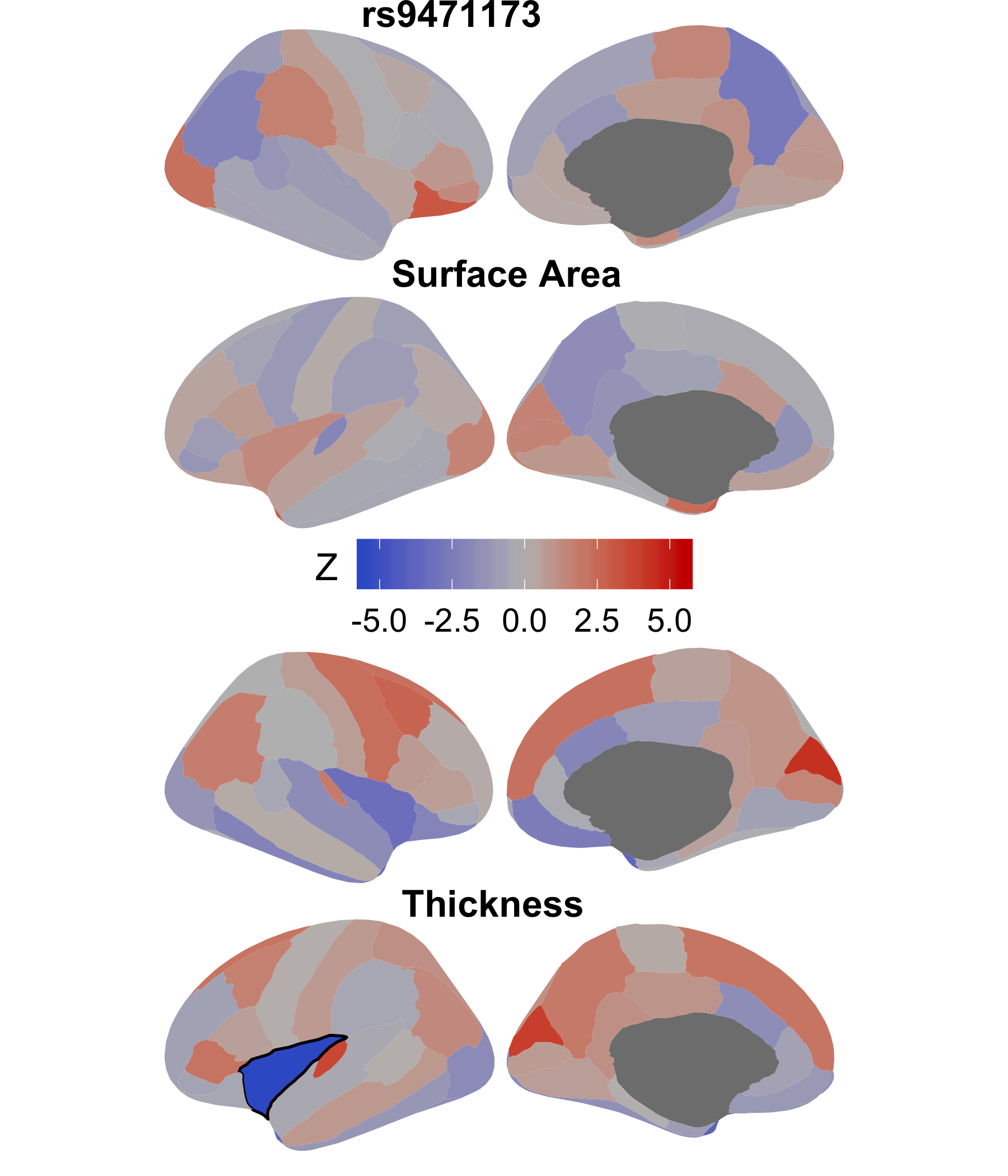

Supplement: Supplementary file 17 — Supplementary Data 14 [file 41467_2020_17368_MOESM17_ESM.gz › BrainMaps/most_dk_thick/BrainMap051_rs9471173.png]

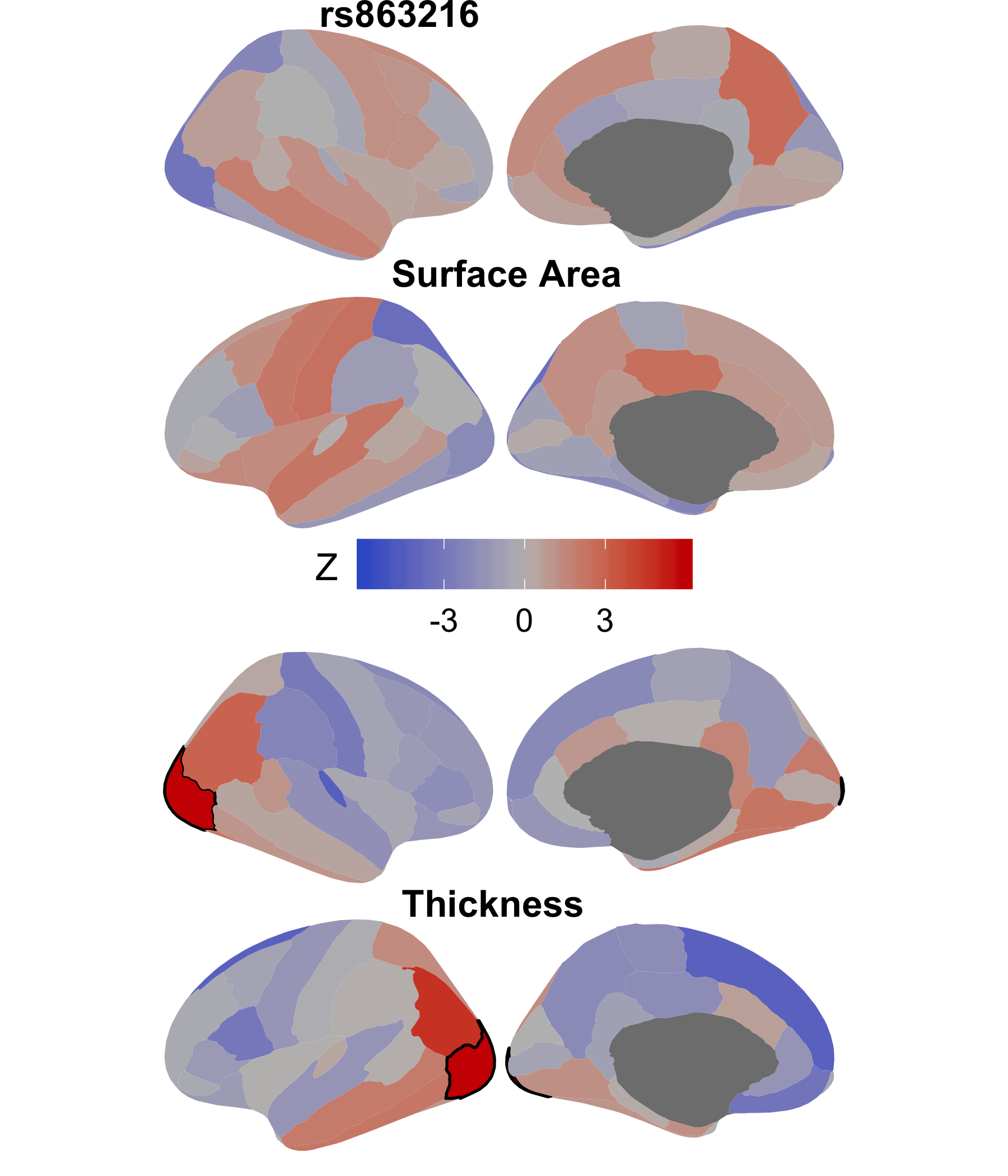

Supplement: Supplementary file 17 — Supplementary Data 14 [file 41467_2020_17368_MOESM17_ESM.gz › BrainMaps/most_dk_thick/BrainMap033_rs863216.png]

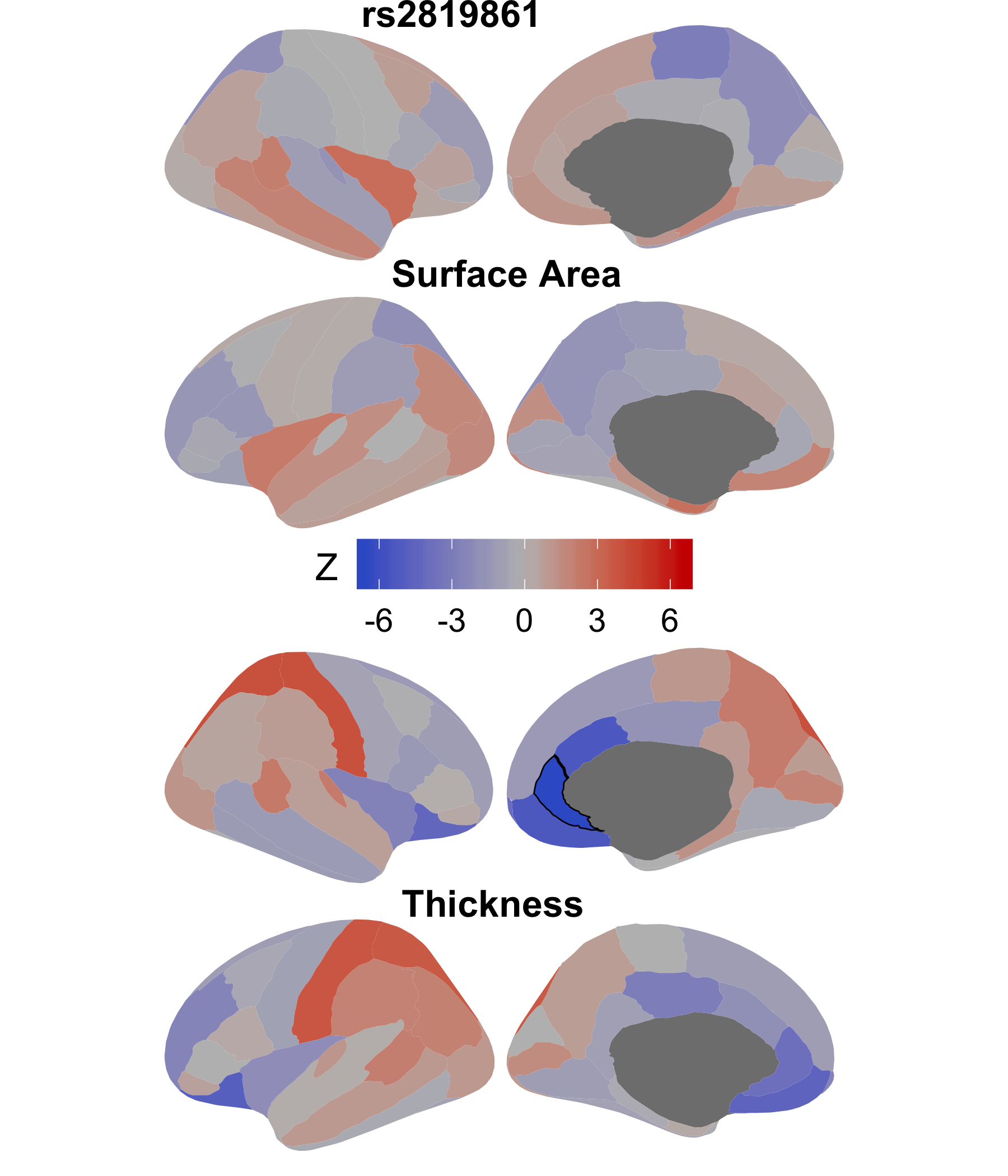

Supplement: Supplementary file 17 — Supplementary Data 14 [file 41467_2020_17368_MOESM17_ESM.gz › BrainMaps/most_dk_thick/BrainMap045_rs2819861.png]

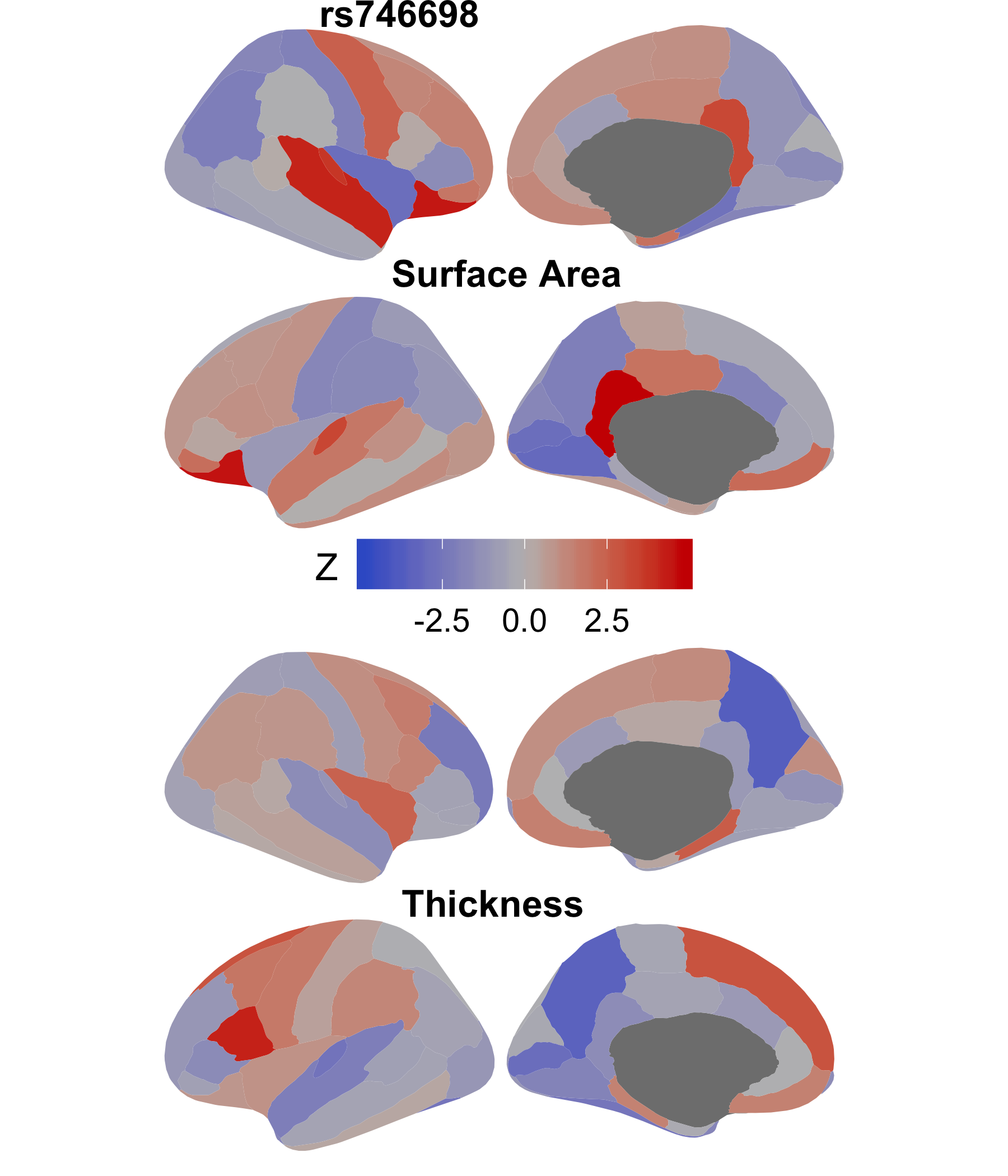

Supplement: Supplementary file 17 — Supplementary Data 14 [file 41467_2020_17368_MOESM17_ESM.gz › BrainMaps/most_dk_thick/BrainMap057_rs746698.png]

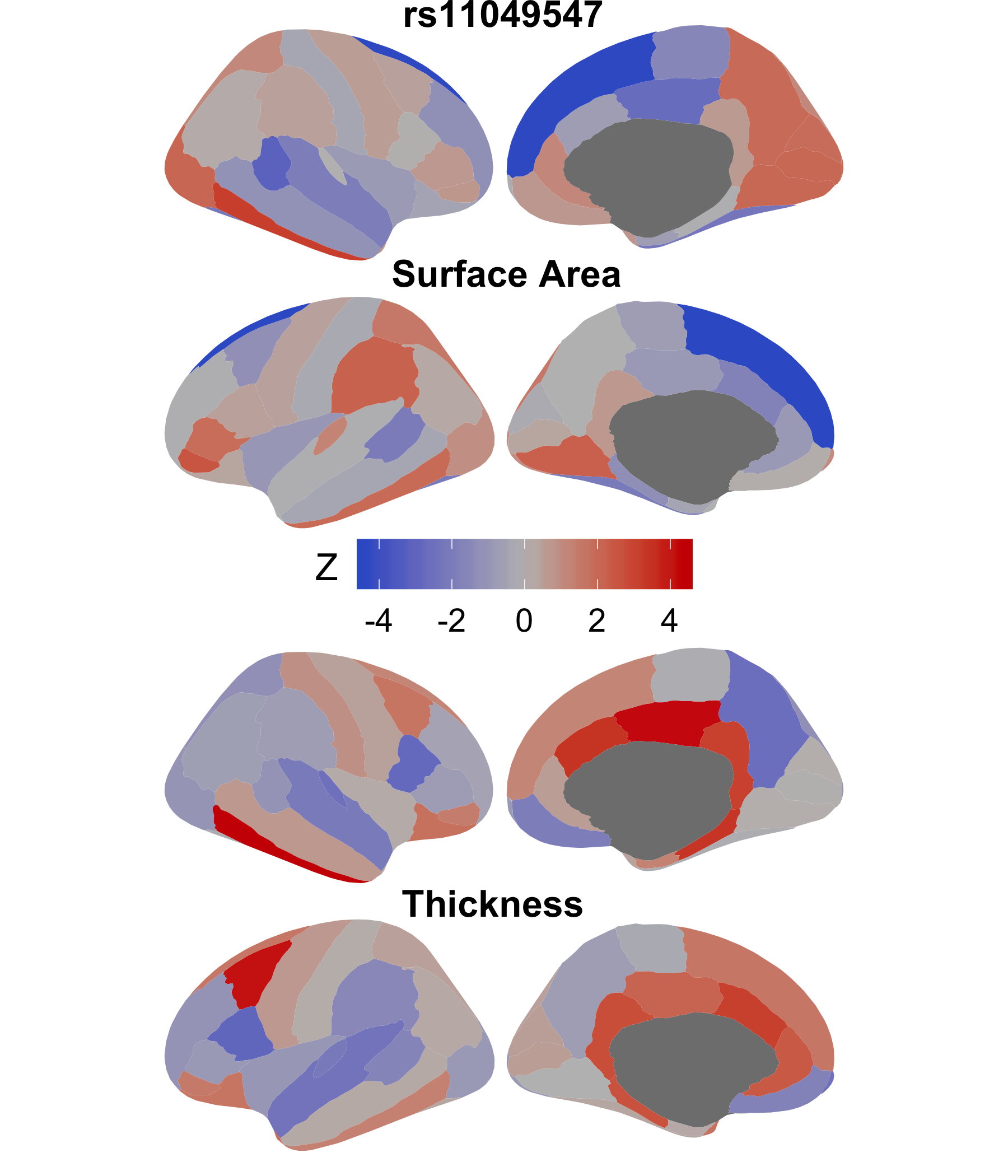

Supplement: Supplementary file 17 — Supplementary Data 14 [file 41467_2020_17368_MOESM17_ESM.gz › BrainMaps/most_dk_thick/BrainMap015_rs11049547.png]

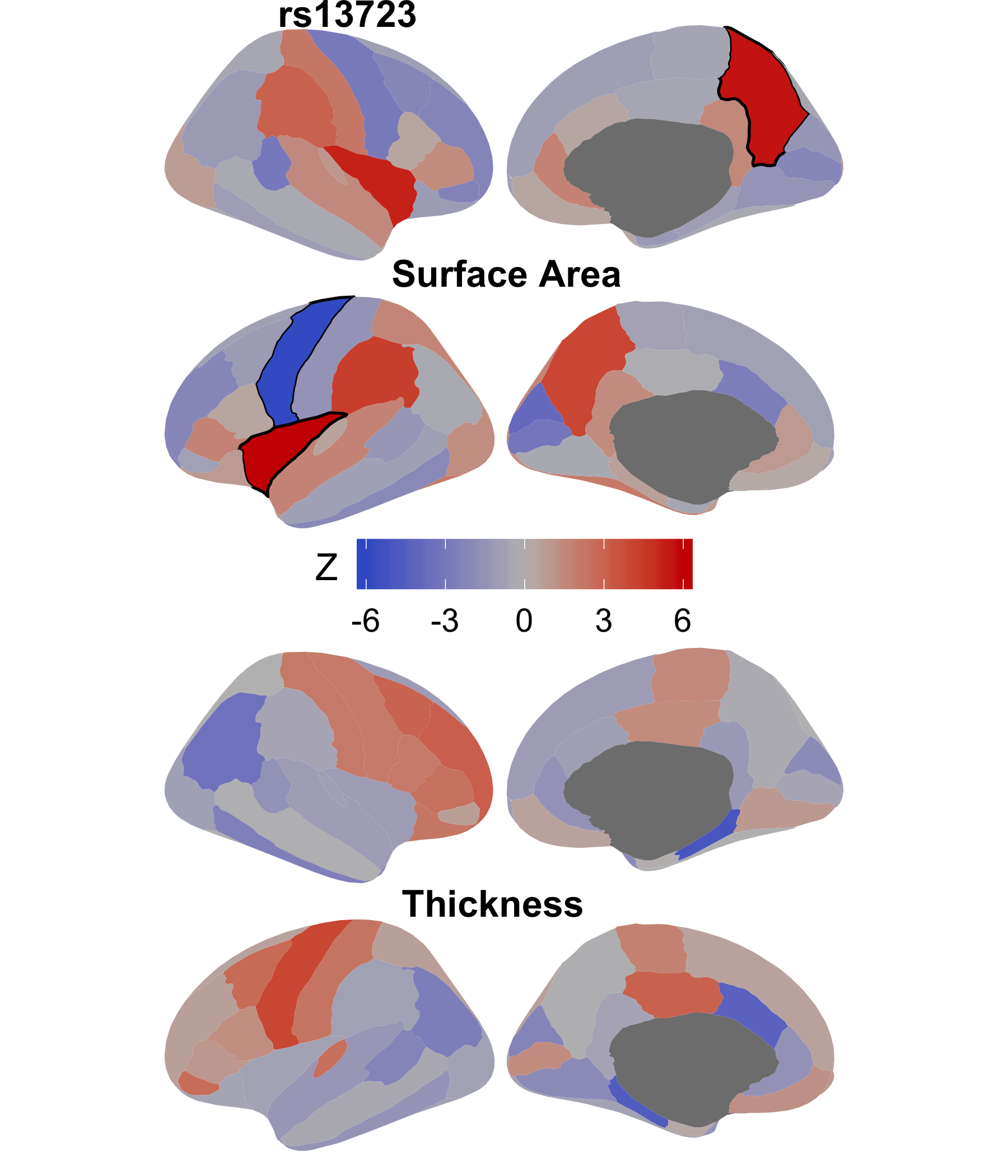

Supplement: Supplementary file 17 — Supplementary Data 14 [file 41467_2020_17368_MOESM17_ESM.gz › BrainMaps/most_dk_thick/BrainMap012_rs13723.png]

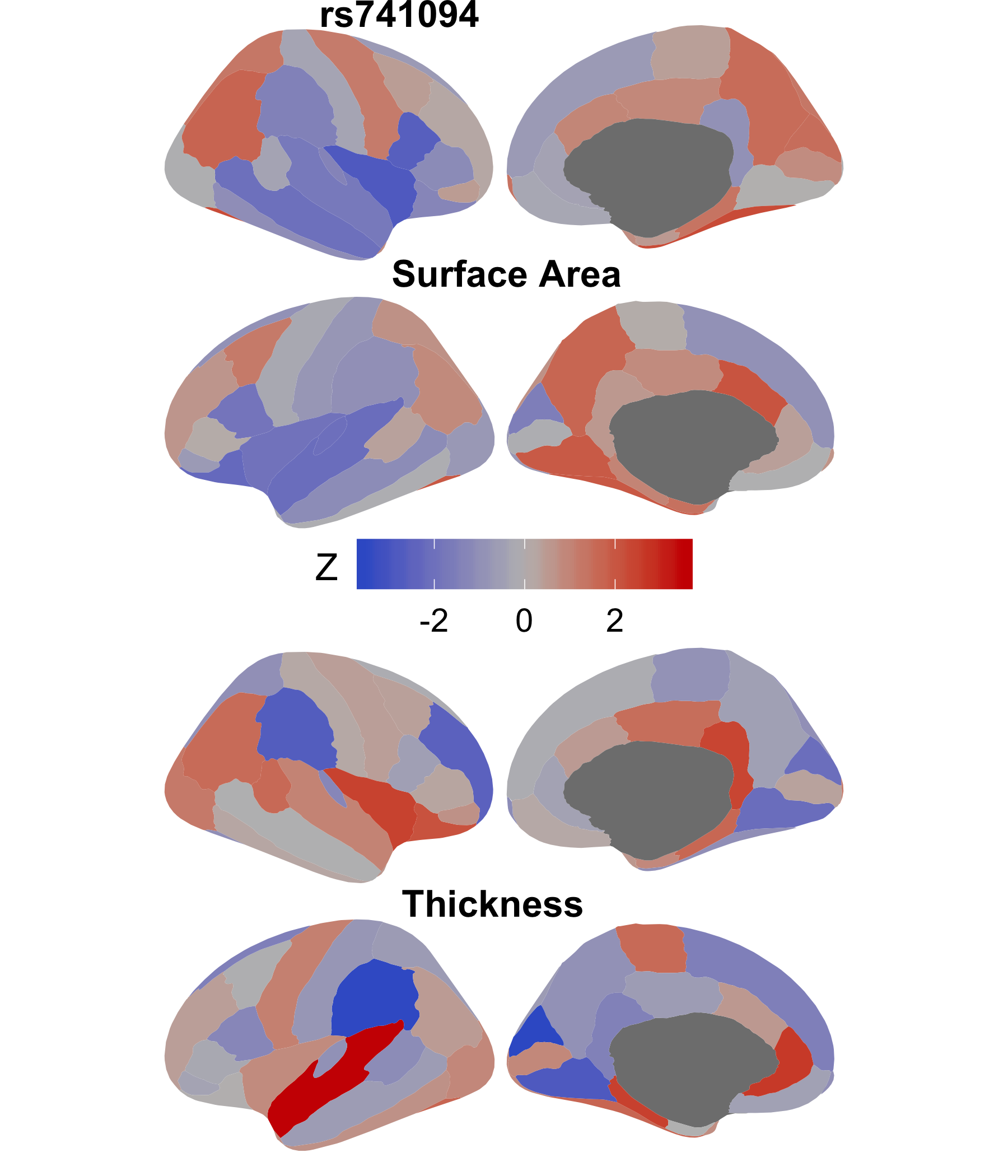

Supplement: Supplementary file 17 — Supplementary Data 14 [file 41467_2020_17368_MOESM17_ESM.gz › BrainMaps/most_dk_thick/BrainMap034_rs741094.png]

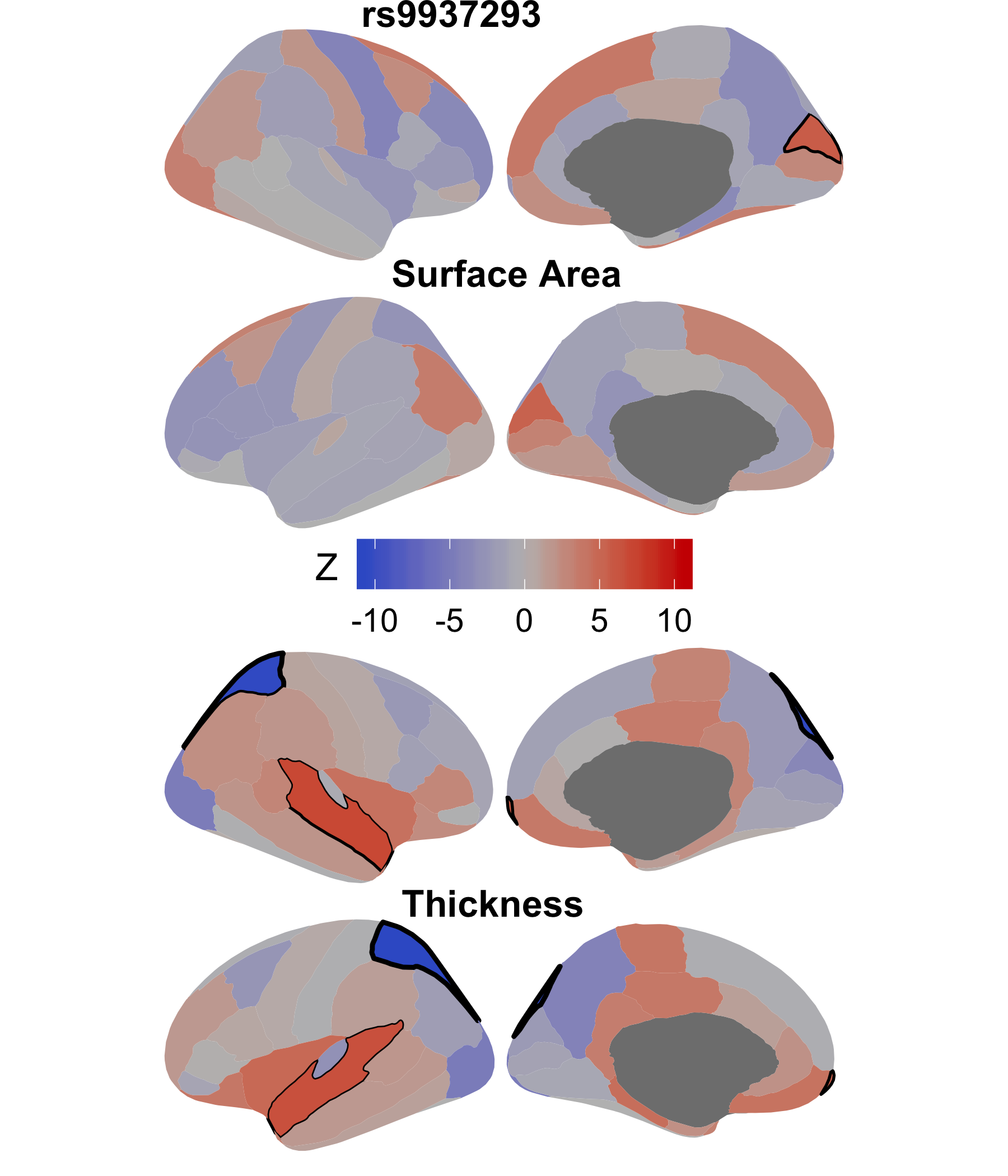

Supplement: Supplementary file 17 — Supplementary Data 14 [file 41467_2020_17368_MOESM17_ESM.gz › BrainMaps/most_dk_thick/BrainMap002_rs9937293.png]

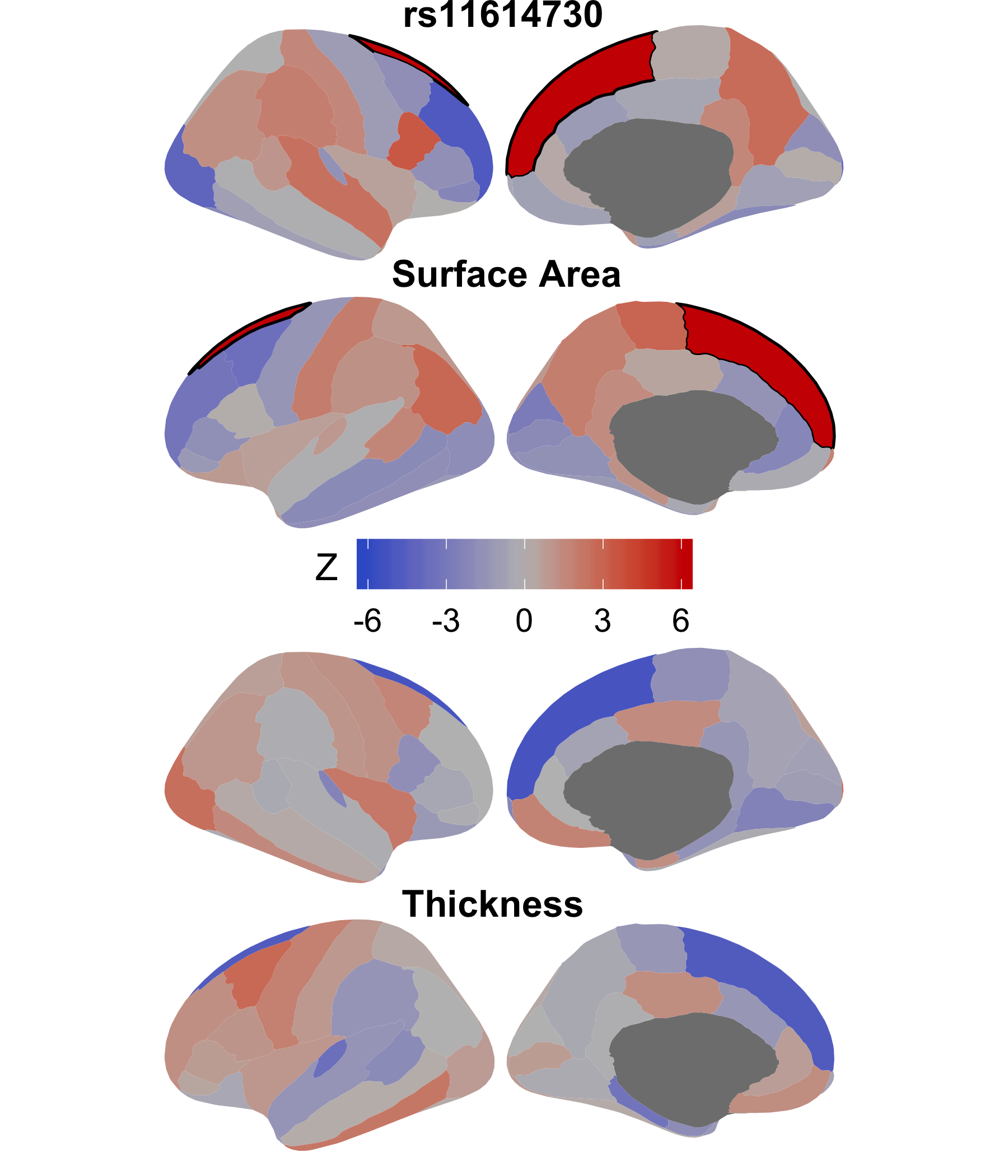

Supplement: Supplementary file 17 — Supplementary Data 14 [file 41467_2020_17368_MOESM17_ESM.gz › BrainMaps/most_dk_thick/BrainMap018_rs11614730.png]

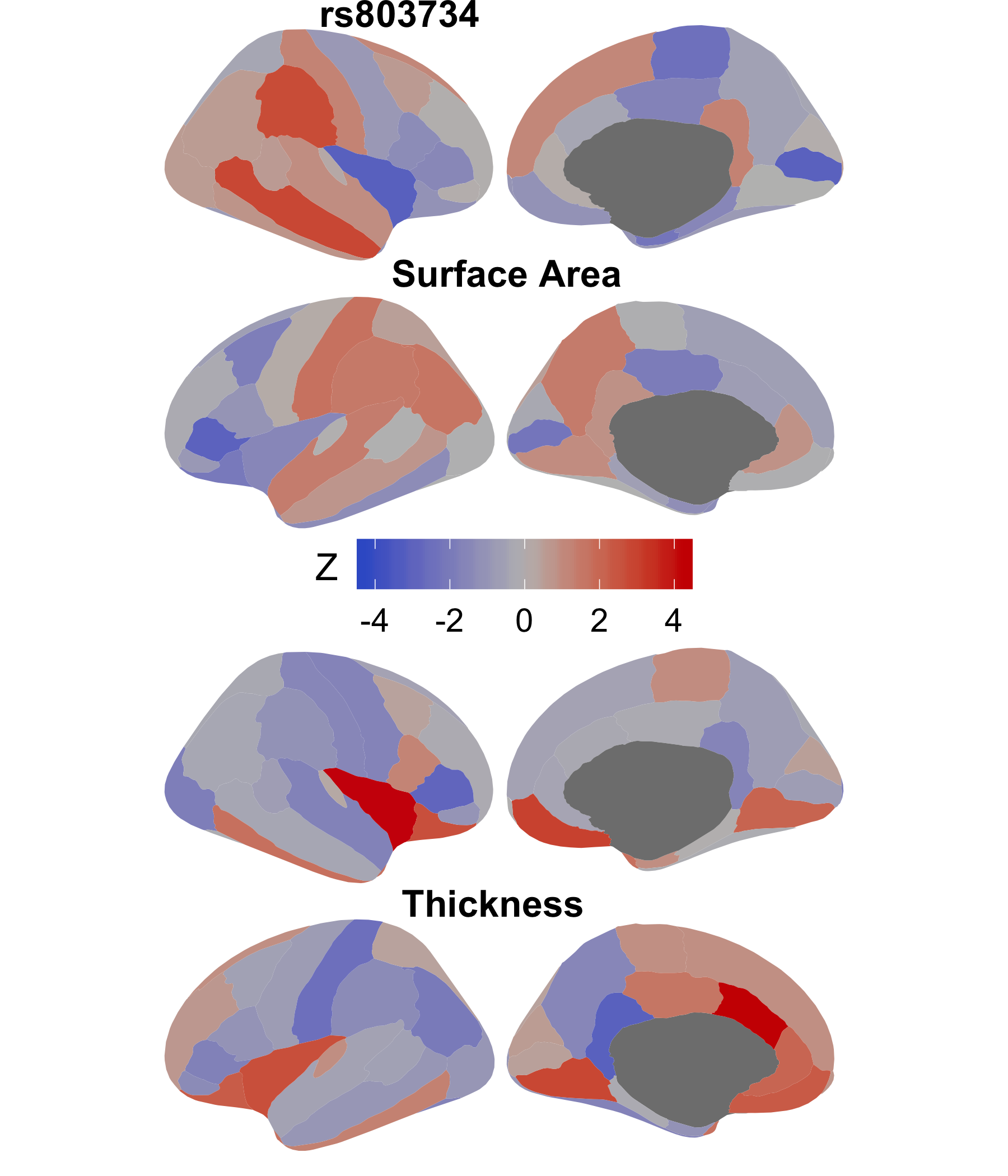

Supplement: Supplementary file 17 — Supplementary Data 14 [file 41467_2020_17368_MOESM17_ESM.gz › BrainMaps/most_dk_thick/BrainMap056_rs803734.png]

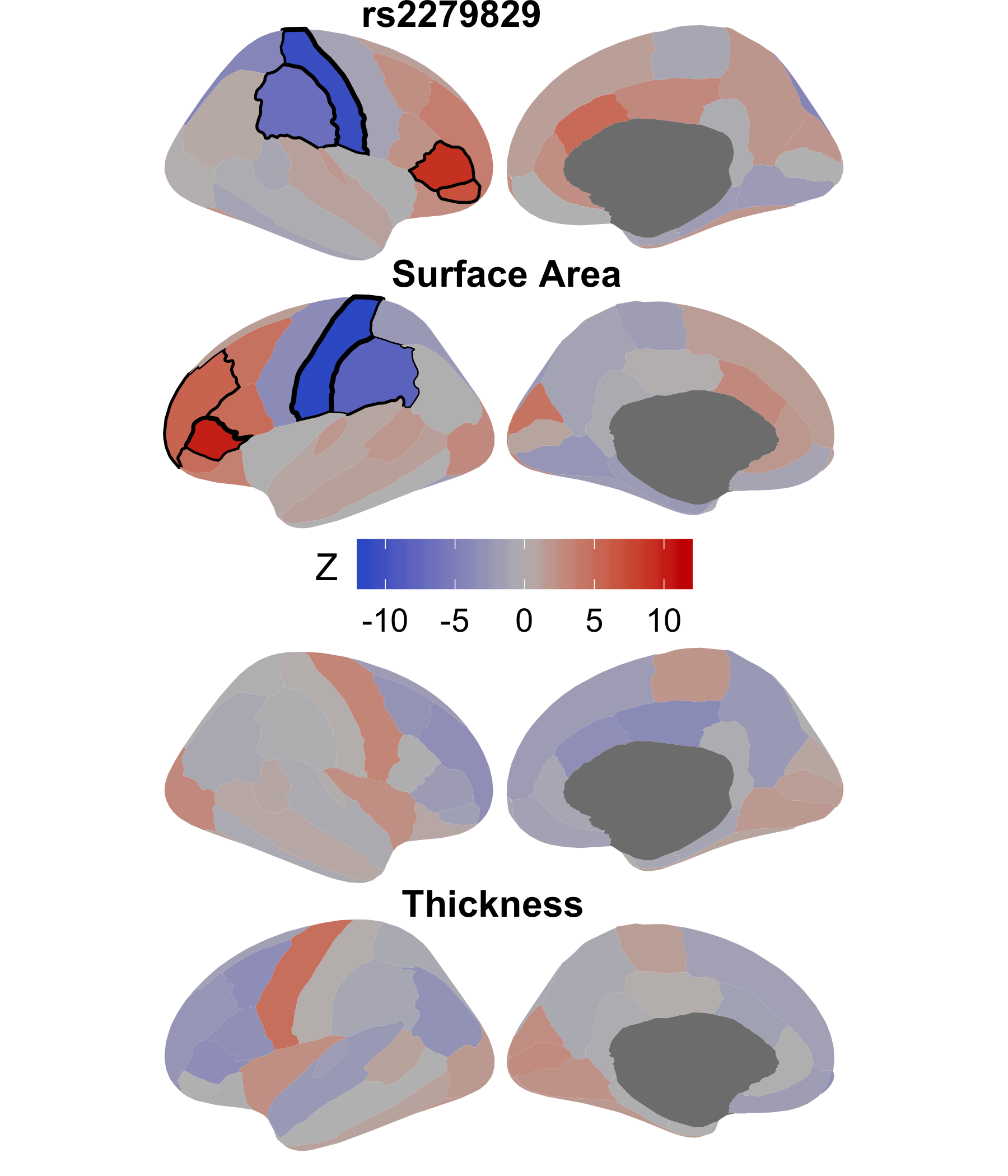

Supplement: Supplementary file 17 — Supplementary Data 14 [file 41467_2020_17368_MOESM17_ESM.gz › BrainMaps/most_dk_thick/BrainMap019_rs2279829.png]

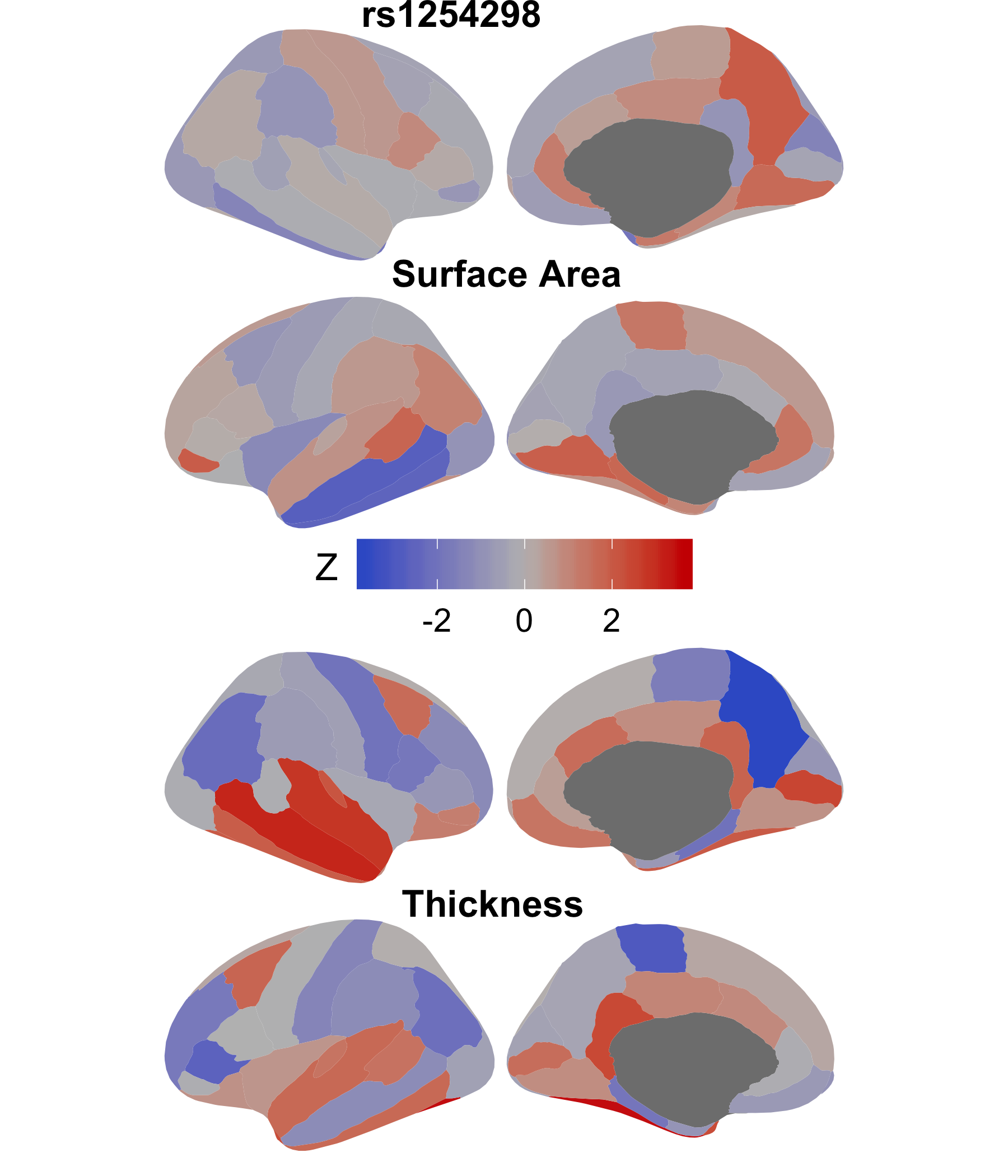

Supplement: Supplementary file 17 — Supplementary Data 14 [file 41467_2020_17368_MOESM17_ESM.gz › BrainMaps/most_dk_thick/BrainMap062_rs1254298.png]

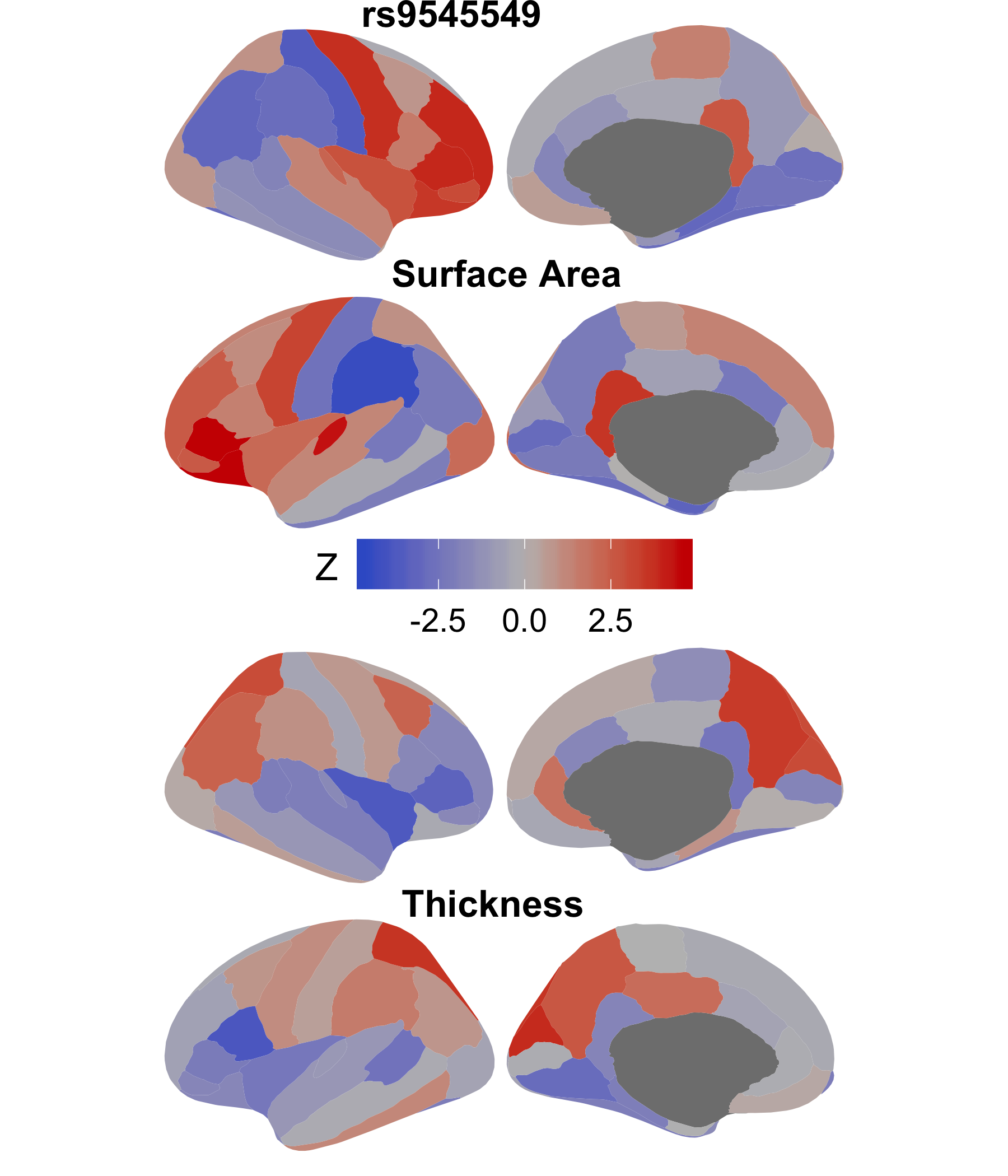

Supplement: Supplementary file 17 — Supplementary Data 14 [file 41467_2020_17368_MOESM17_ESM.gz › BrainMaps/most_dk_thick/BrainMap036_rs9545549.png]

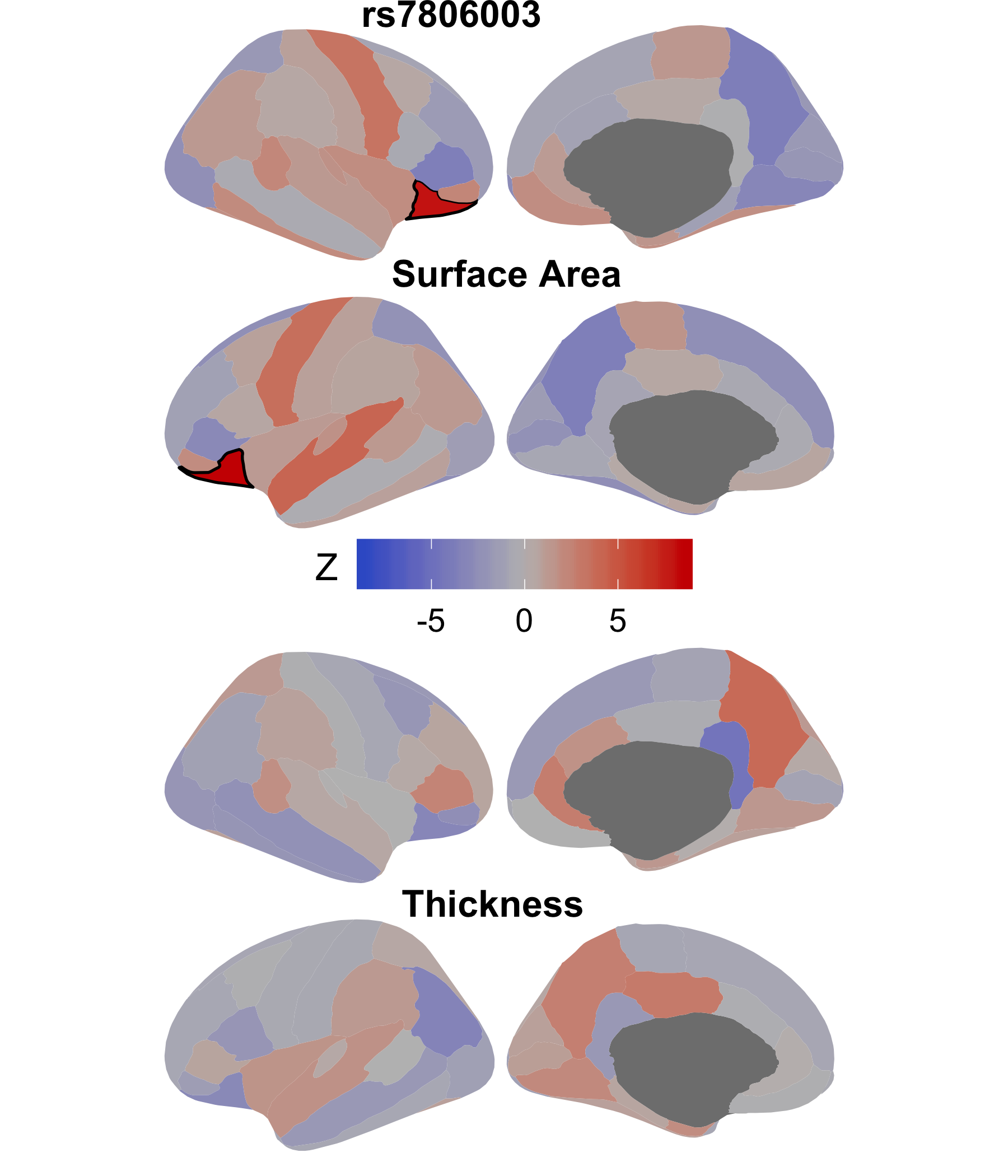

Supplement: Supplementary file 17 — Supplementary Data 14 [file 41467_2020_17368_MOESM17_ESM.gz › BrainMaps/most_dk_thick/BrainMap020_rs7806003.png]

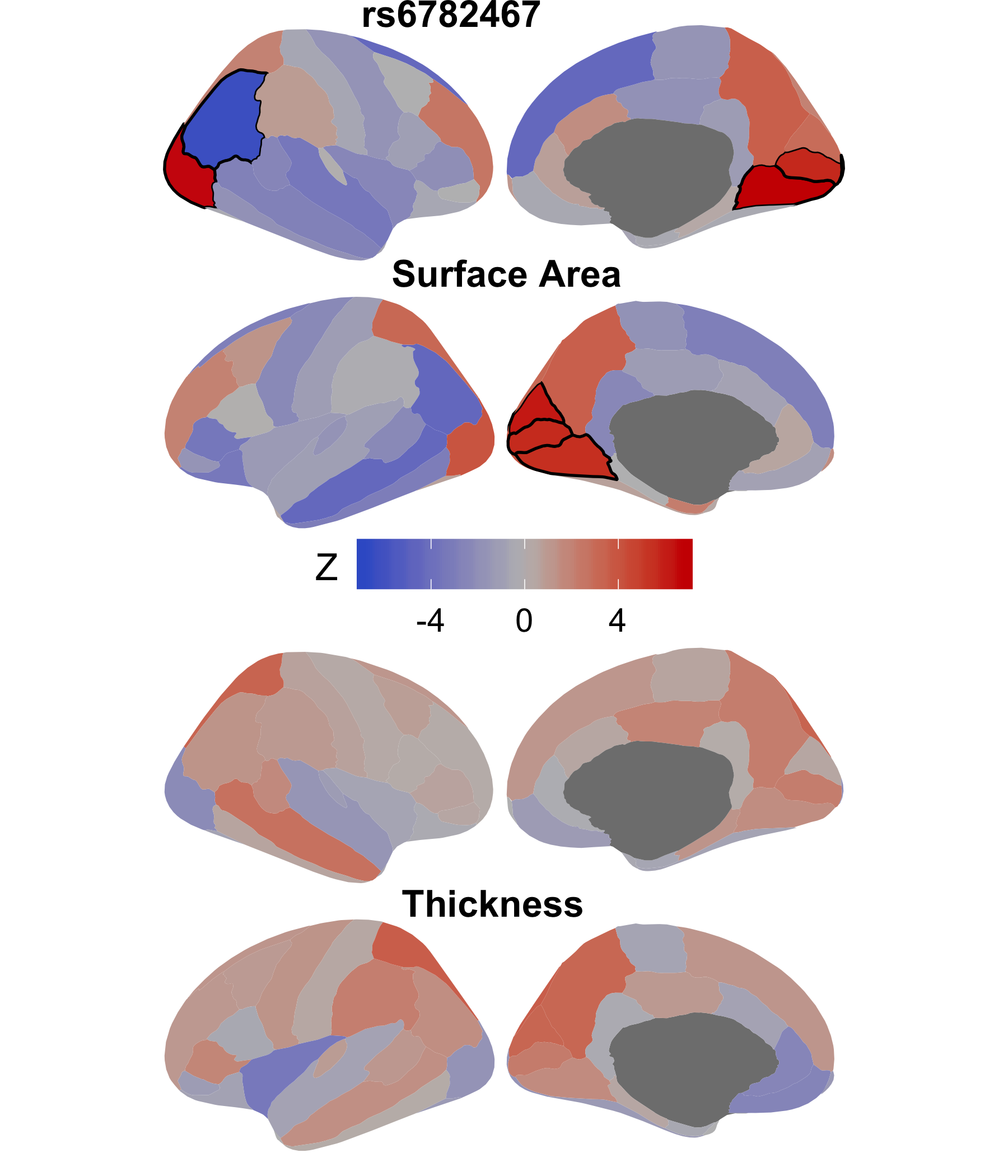

Supplement: Supplementary file 17 — Supplementary Data 14 [file 41467_2020_17368_MOESM17_ESM.gz › BrainMaps/most_dk_thick/BrainMap016_rs6782467.png]

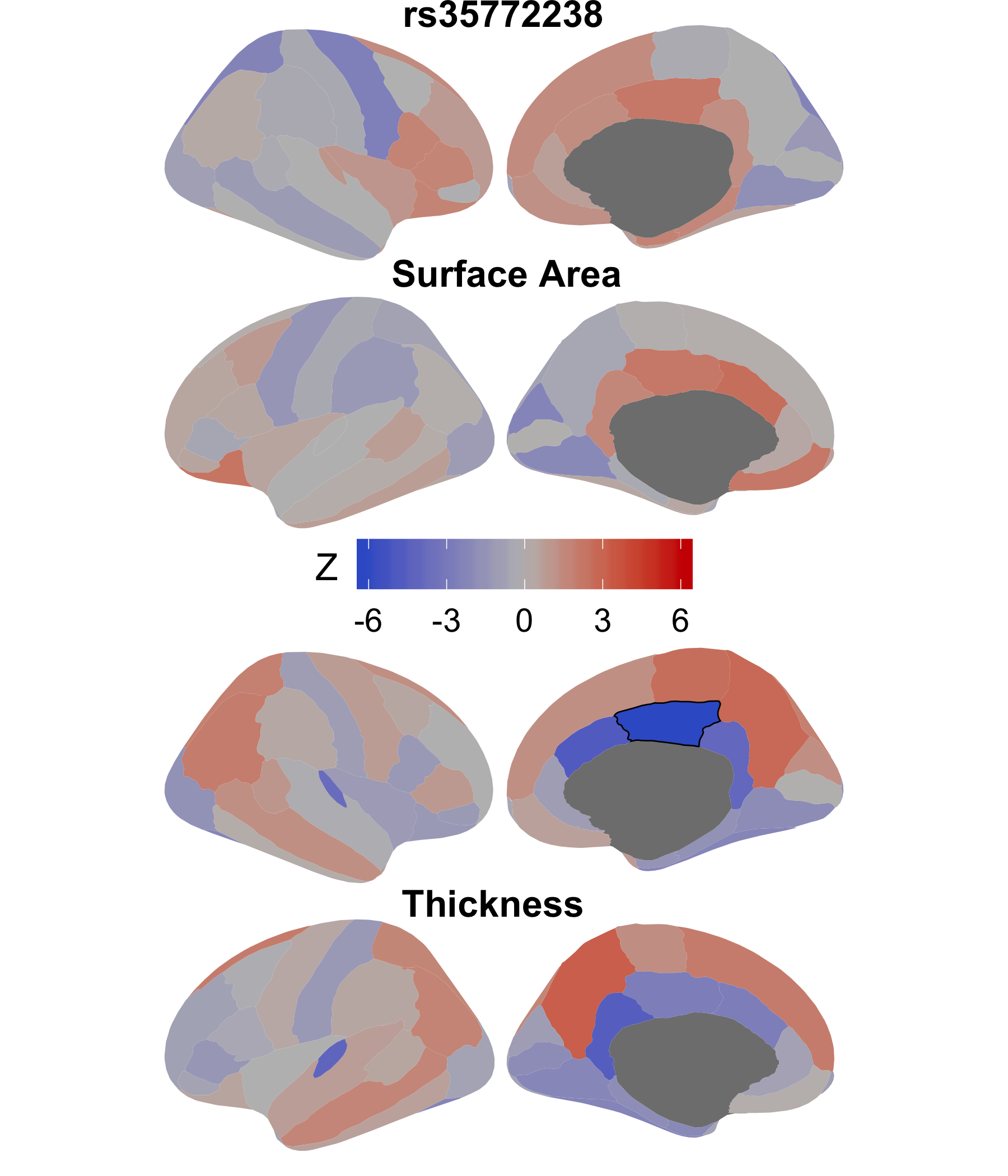

Supplement: Supplementary file 17 — Supplementary Data 14 [file 41467_2020_17368_MOESM17_ESM.gz › BrainMaps/most_dk_thick/BrainMap028_rs35772238.png]

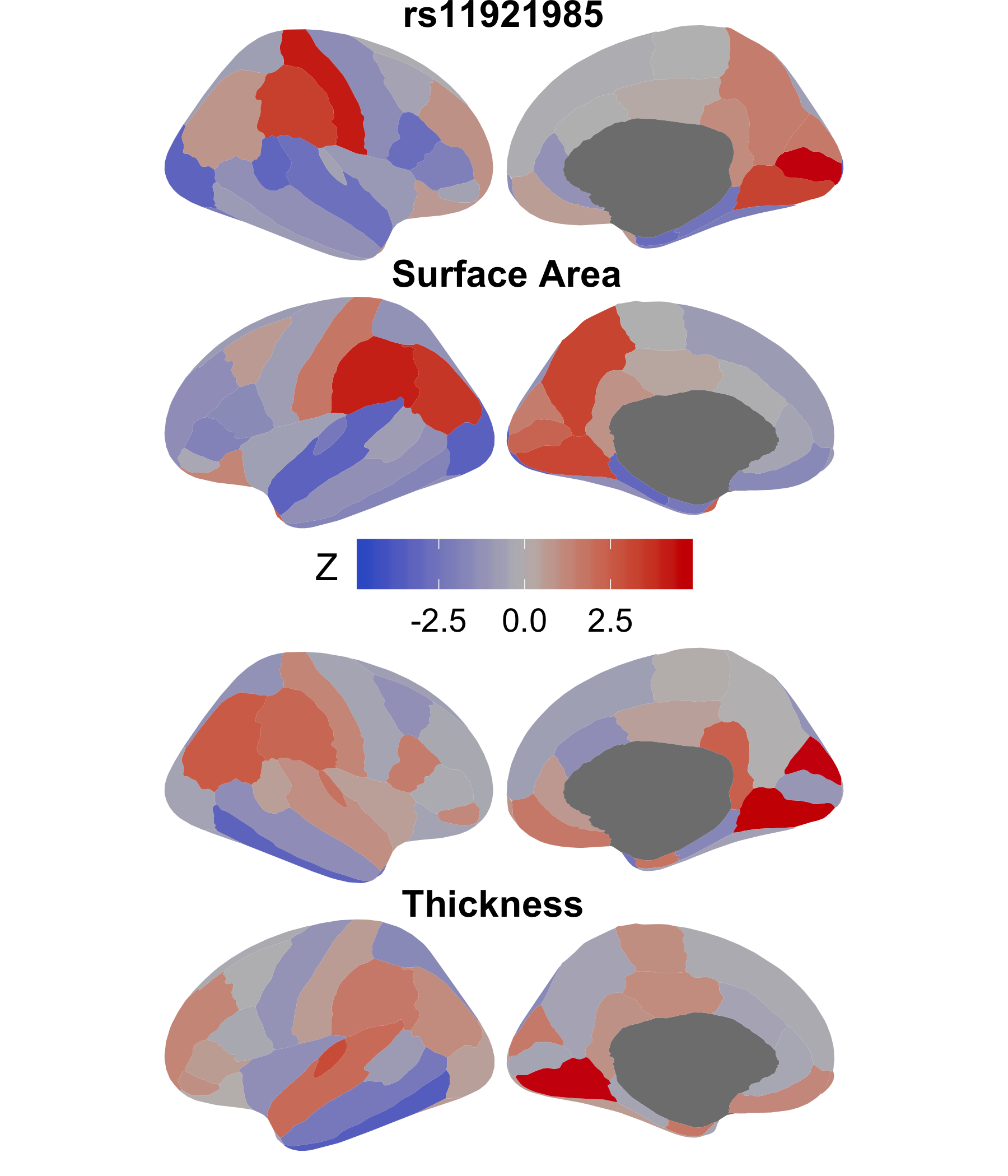

Supplement: Supplementary file 17 — Supplementary Data 14 [file 41467_2020_17368_MOESM17_ESM.gz › BrainMaps/most_dk_thick/BrainMap022_rs11921985.png]

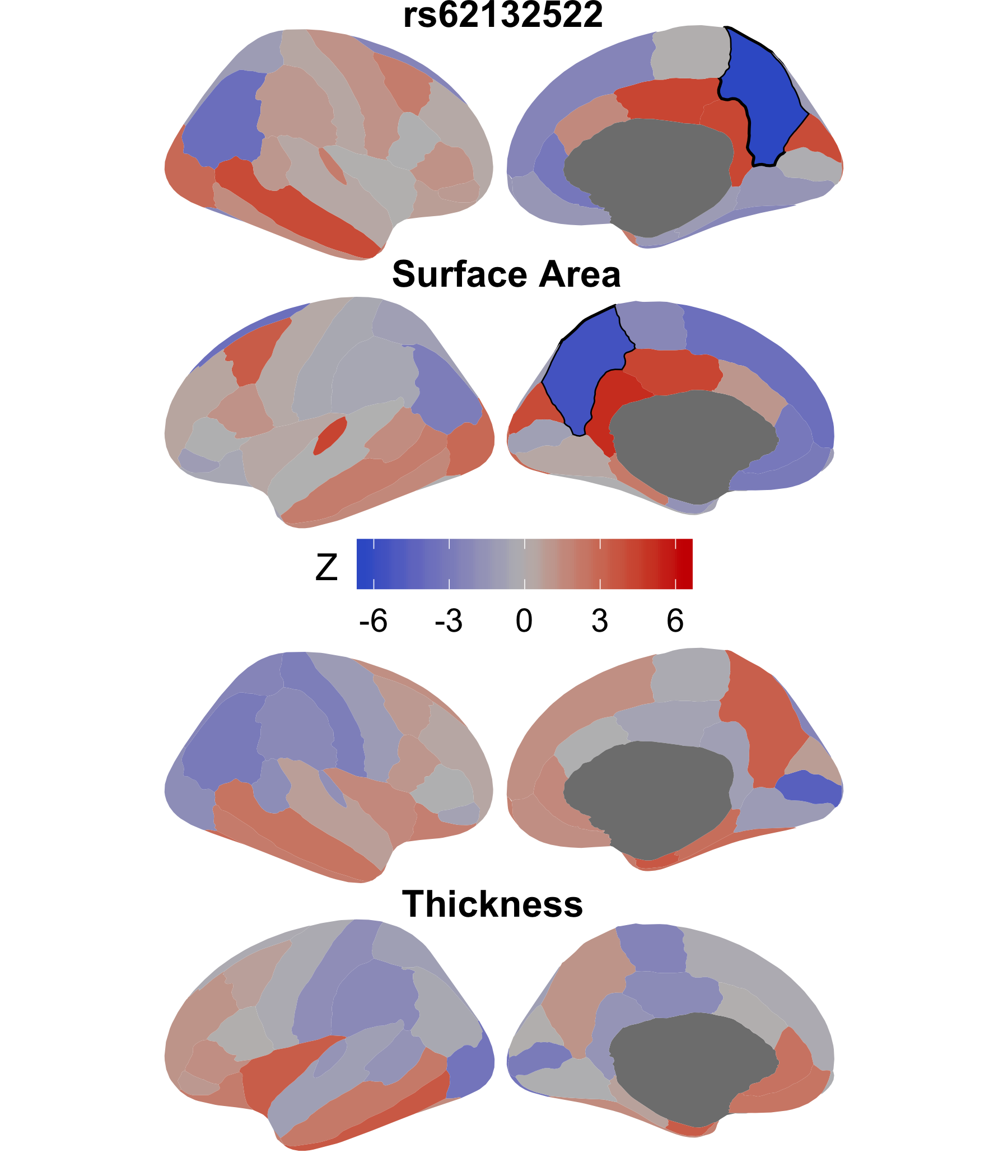

Supplement: Supplementary file 17 — Supplementary Data 14 [file 41467_2020_17368_MOESM17_ESM.gz › BrainMaps/most_dk_thick/BrainMap049_rs62132522.png]

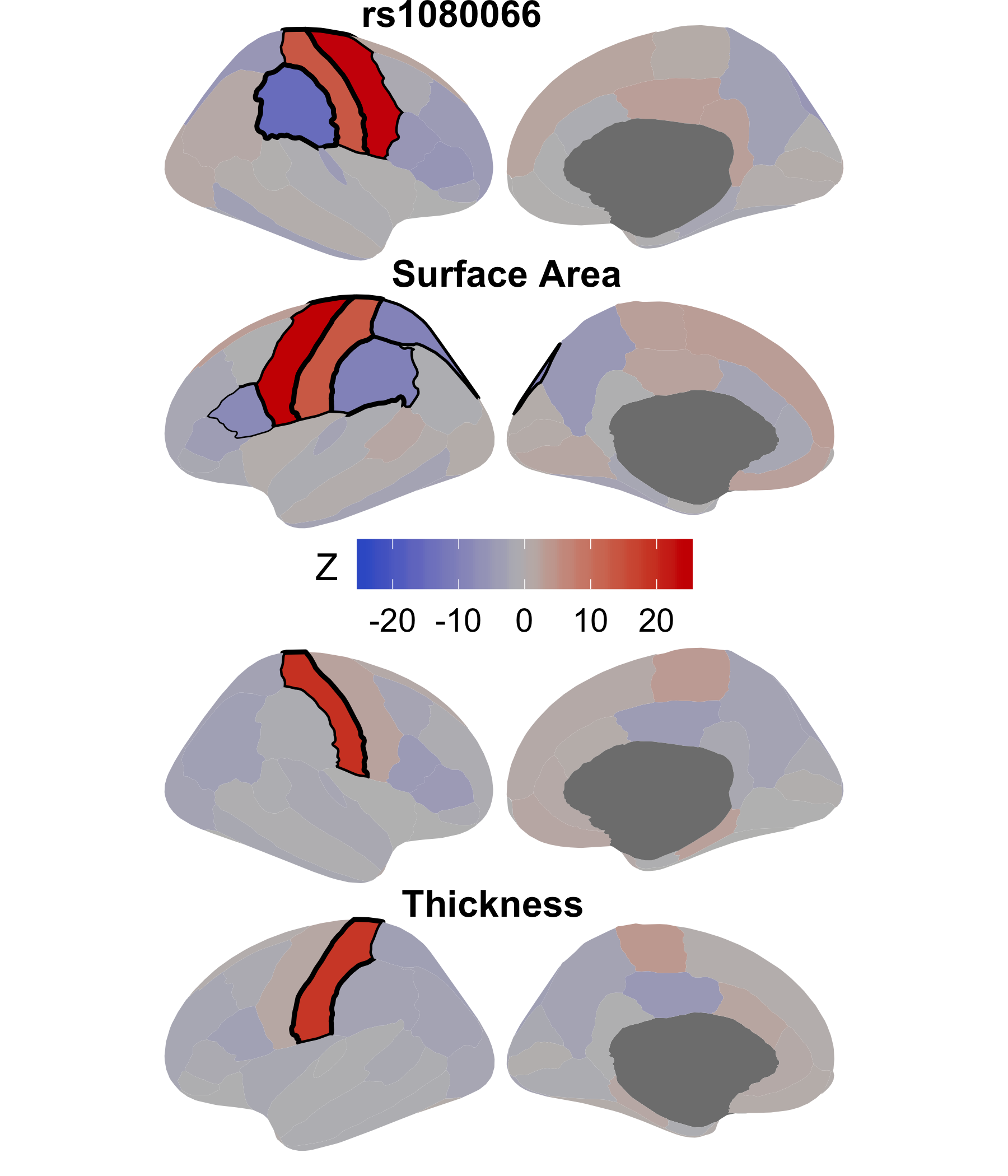

Supplement: Supplementary file 17 — Supplementary Data 14 [file 41467_2020_17368_MOESM17_ESM.gz › BrainMaps/most_dk_thick/BrainMap001_rs1080066.png]

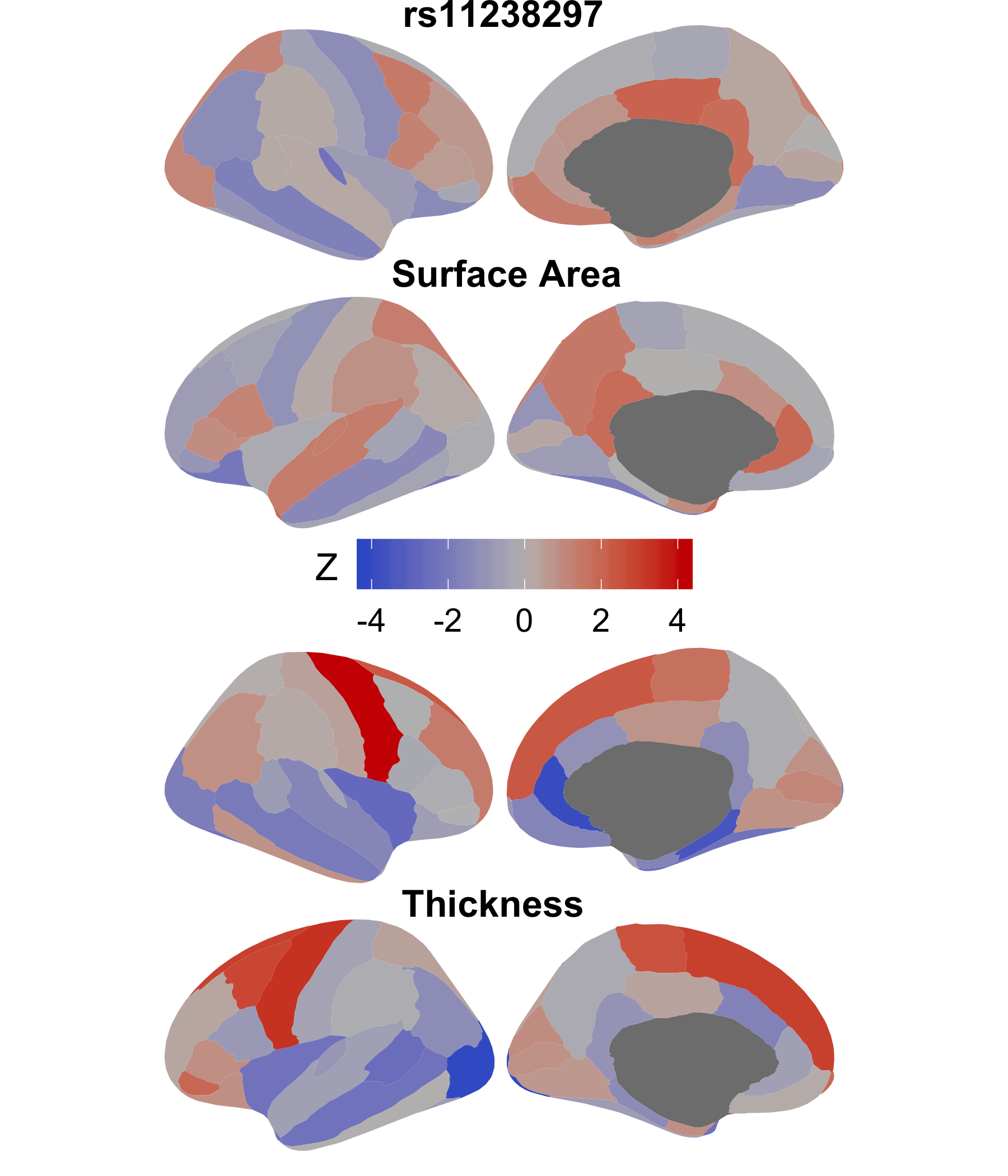

Supplement: Supplementary file 17 — Supplementary Data 14 [file 41467_2020_17368_MOESM17_ESM.gz › BrainMaps/most_dk_thick/BrainMap065_rs11238297.png]

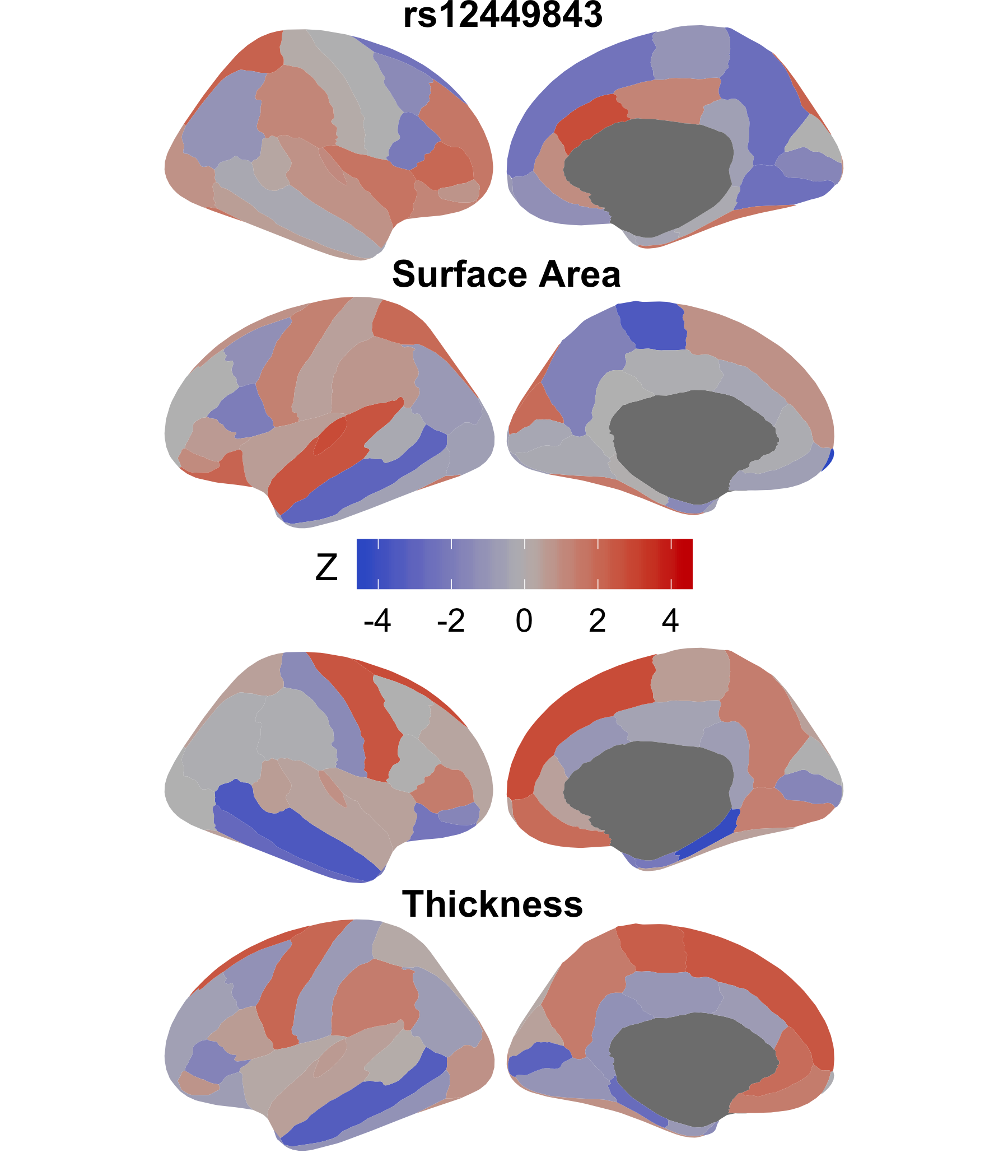

Supplement: Supplementary file 17 — Supplementary Data 14 [file 41467_2020_17368_MOESM17_ESM.gz › BrainMaps/most_dk_thick/BrainMap060_rs12449843.png]

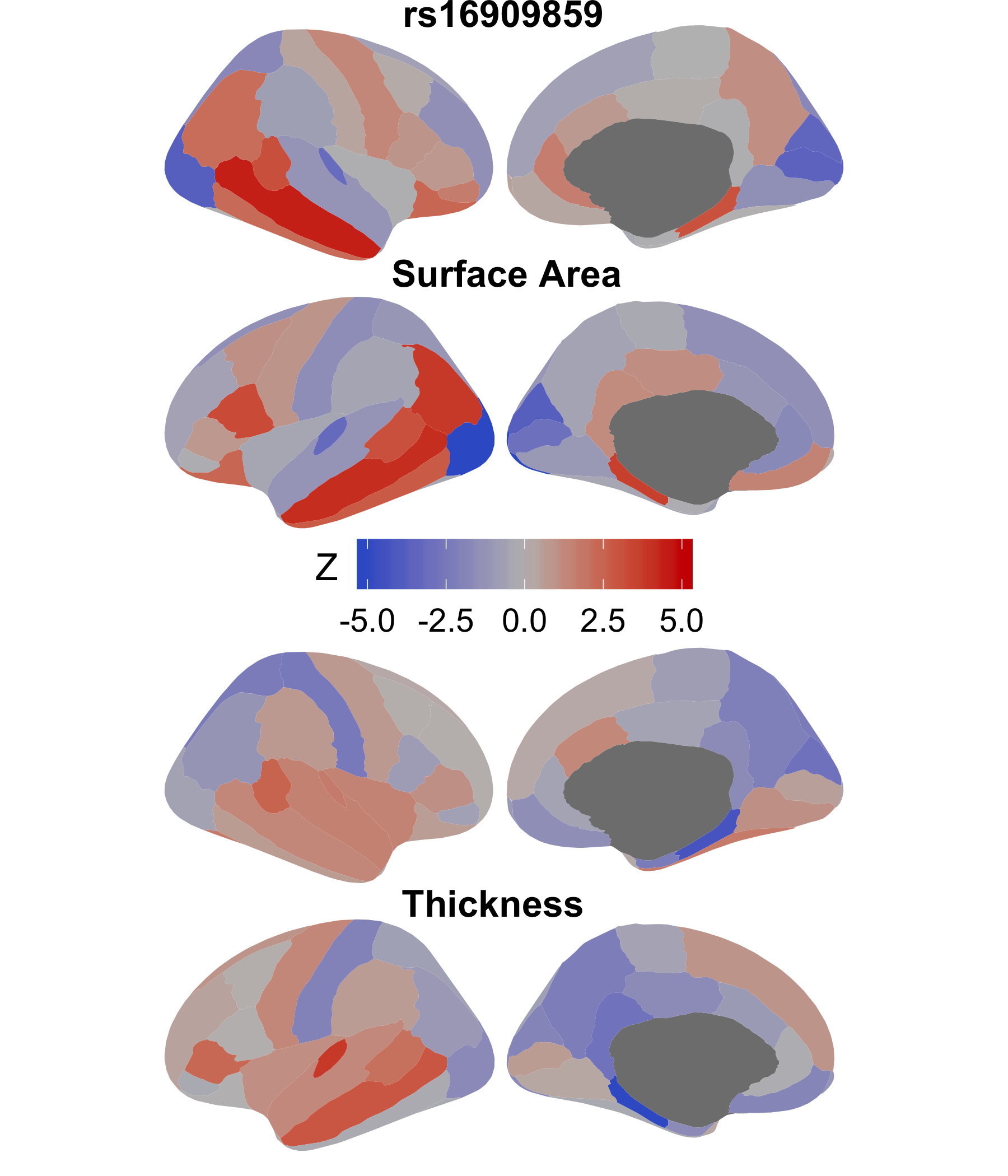

Supplement: Supplementary file 17 — Supplementary Data 14 [file 41467_2020_17368_MOESM17_ESM.gz › BrainMaps/most_dk_thick/BrainMap054_rs16909859.png]

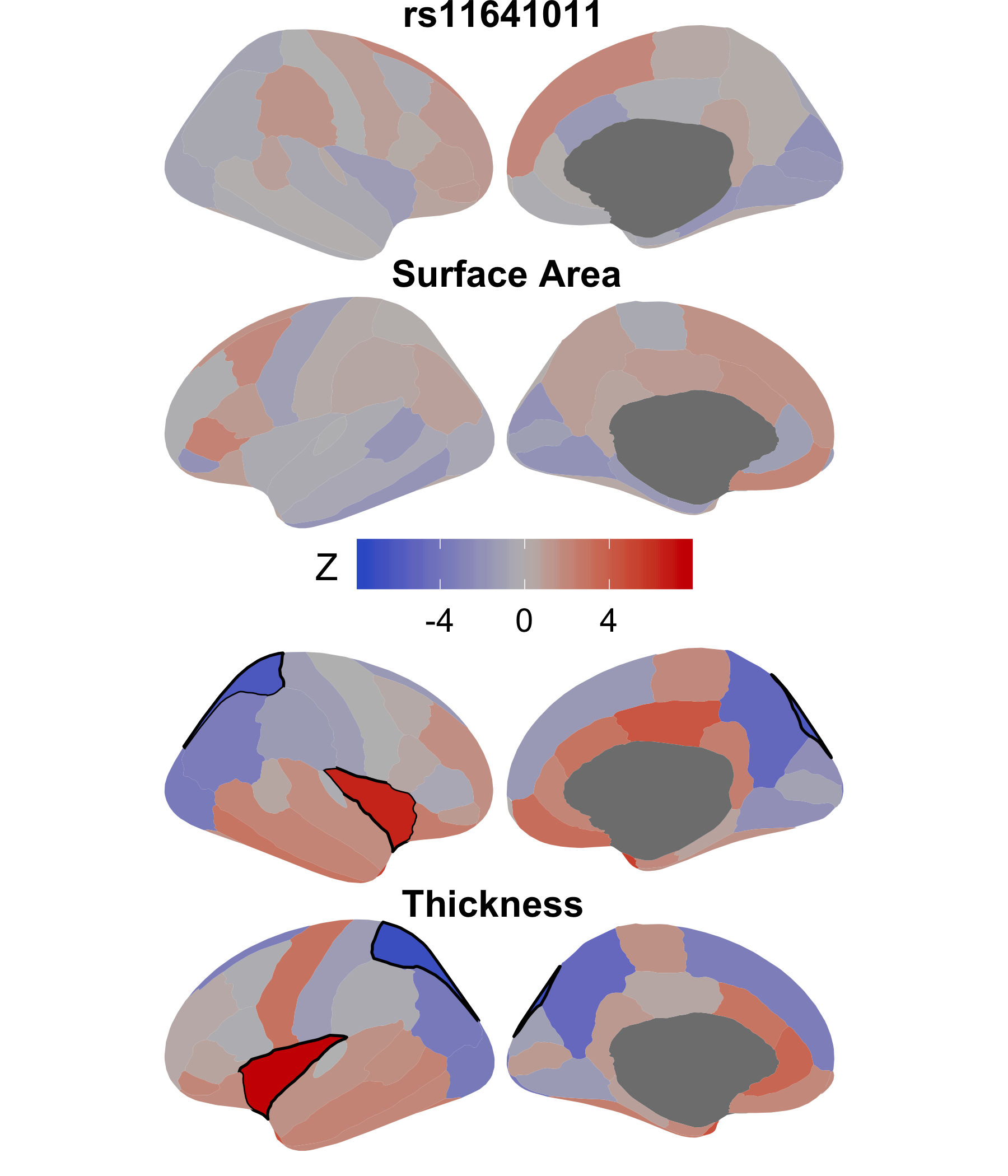

Supplement: Supplementary file 17 — Supplementary Data 14 [file 41467_2020_17368_MOESM17_ESM.gz › BrainMaps/most_dk_thick/BrainMap014_rs11641011.png]

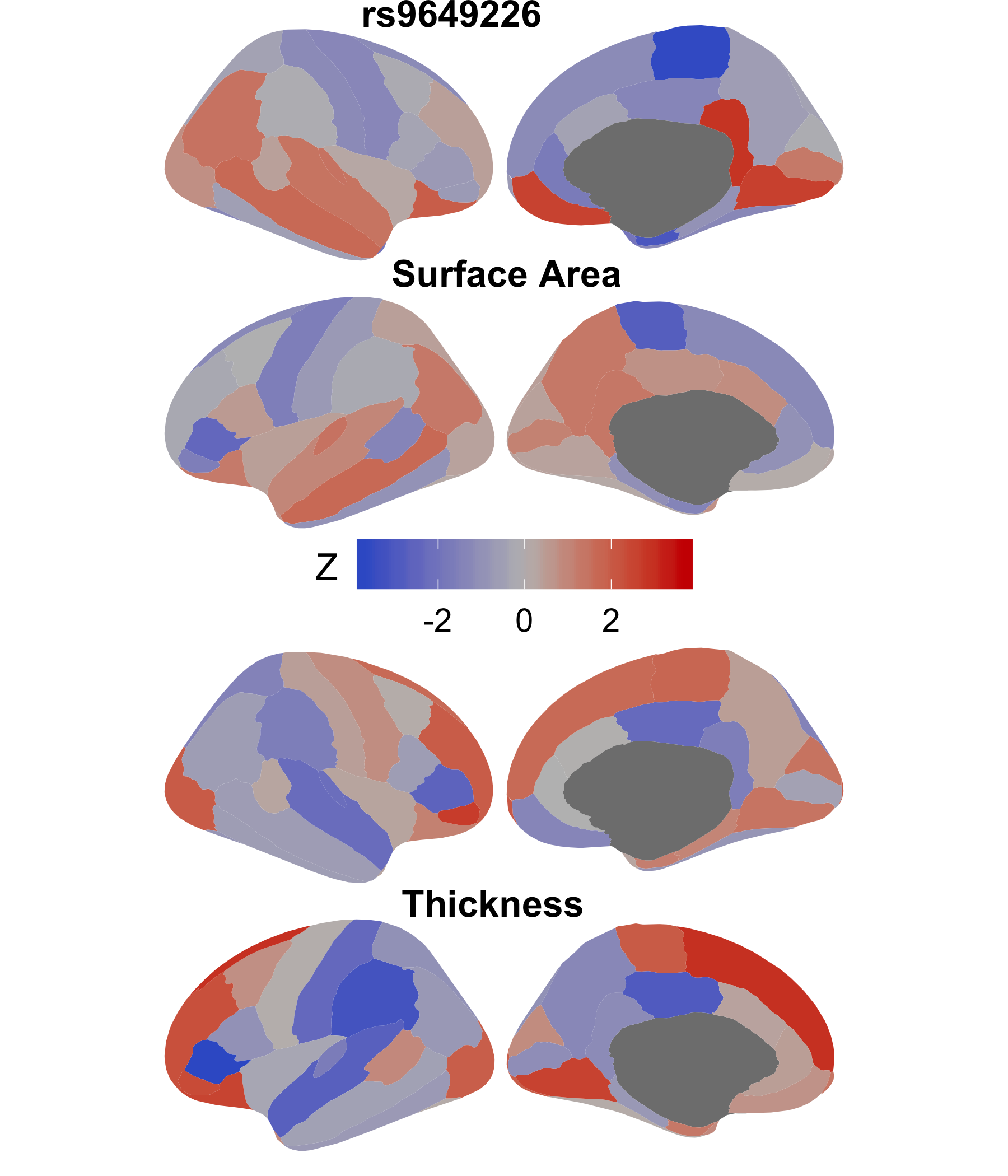

Supplement: Supplementary file 17 — Supplementary Data 14 [file 41467_2020_17368_MOESM17_ESM.gz › BrainMaps/most_dk_thick/BrainMap067_rs9649226.png]

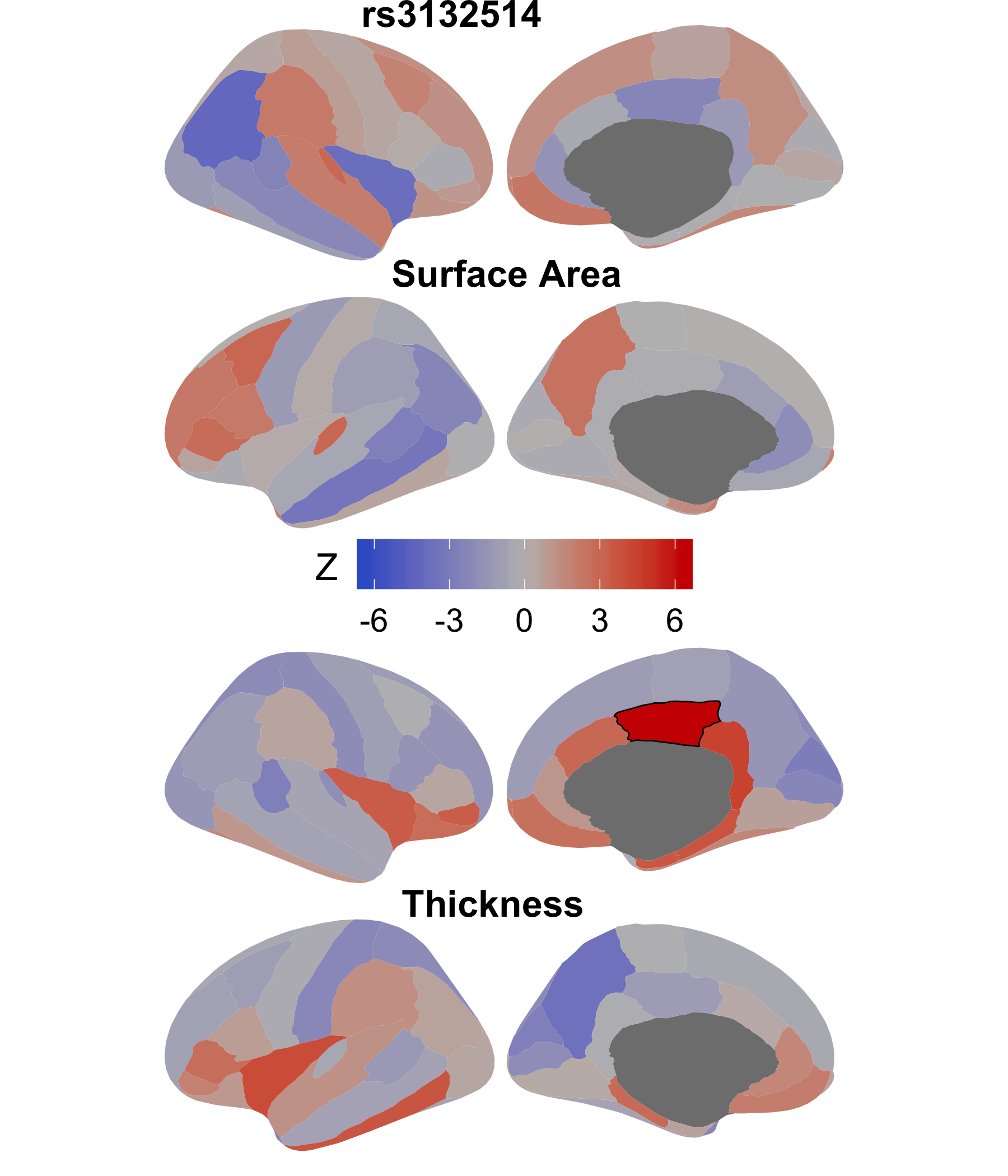

Supplement: Supplementary file 17 — Supplementary Data 14 [file 41467_2020_17368_MOESM17_ESM.gz › BrainMaps/most_dk_thick/BrainMap011_rs3132514.png]

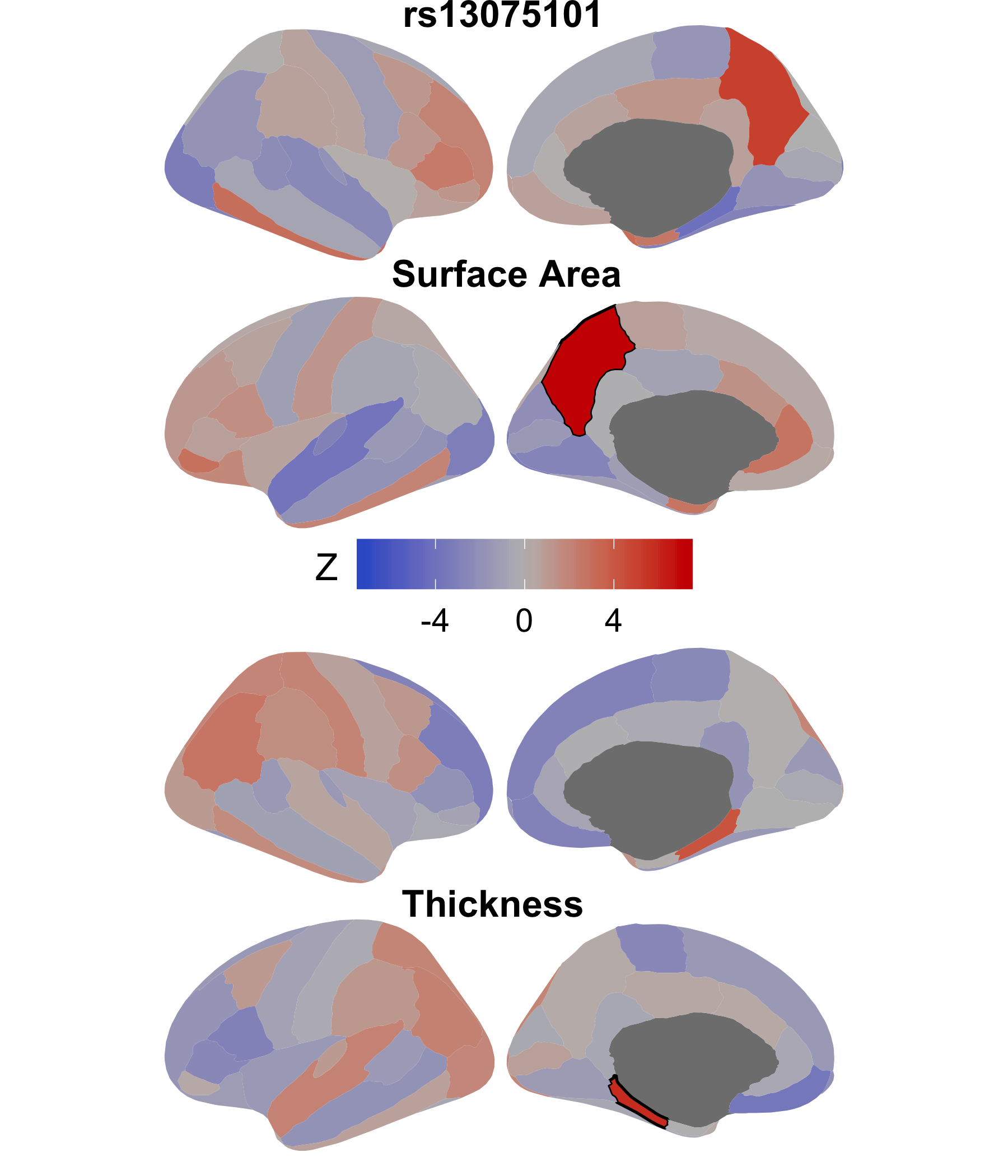

Supplement: Supplementary file 17 — Supplementary Data 14 [file 41467_2020_17368_MOESM17_ESM.gz › BrainMaps/most_dk_thick/BrainMap040_rs13075101.png]

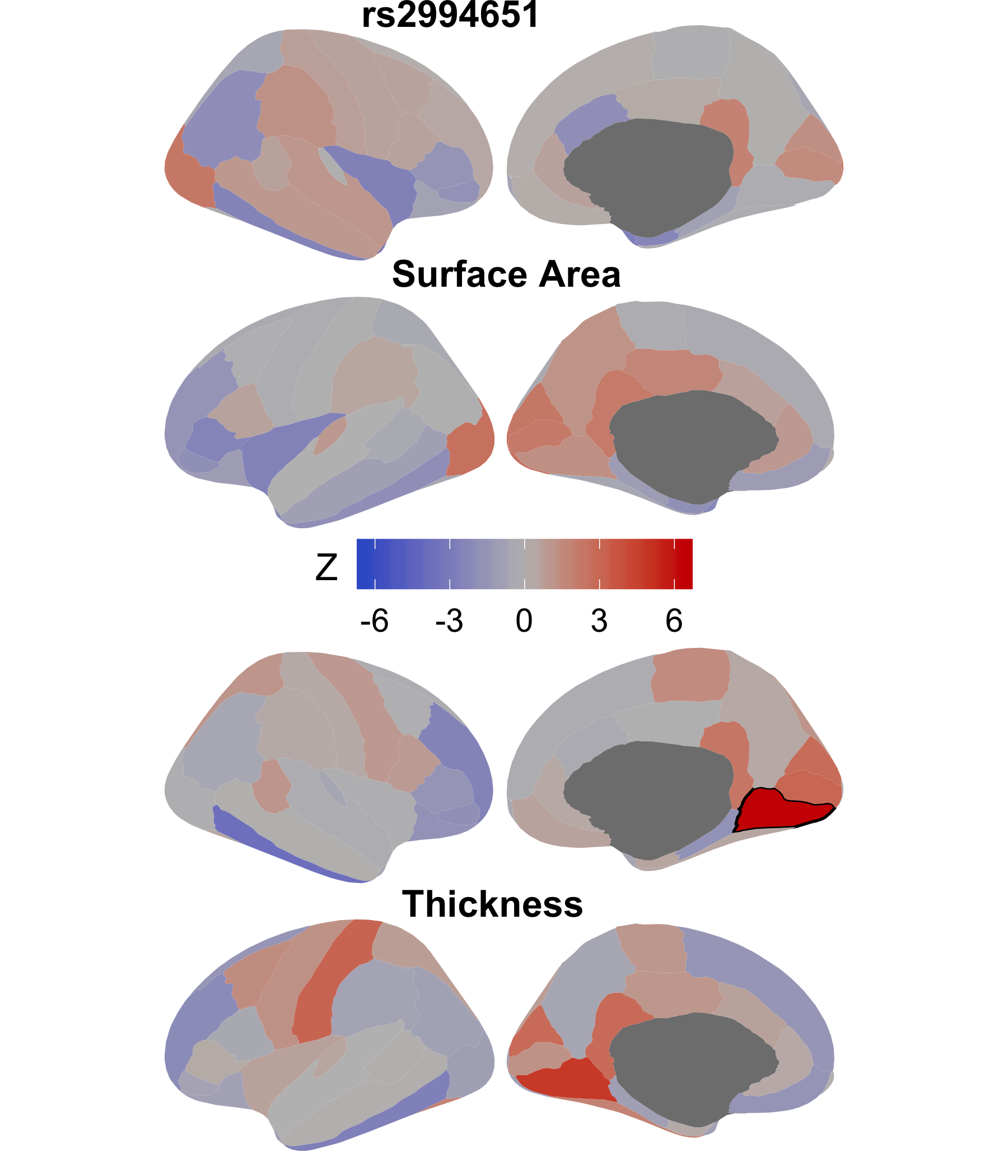

Supplement: Supplementary file 17 — Supplementary Data 14 [file 41467_2020_17368_MOESM17_ESM.gz › BrainMaps/most_dk_thick/BrainMap058_rs2994651.png]

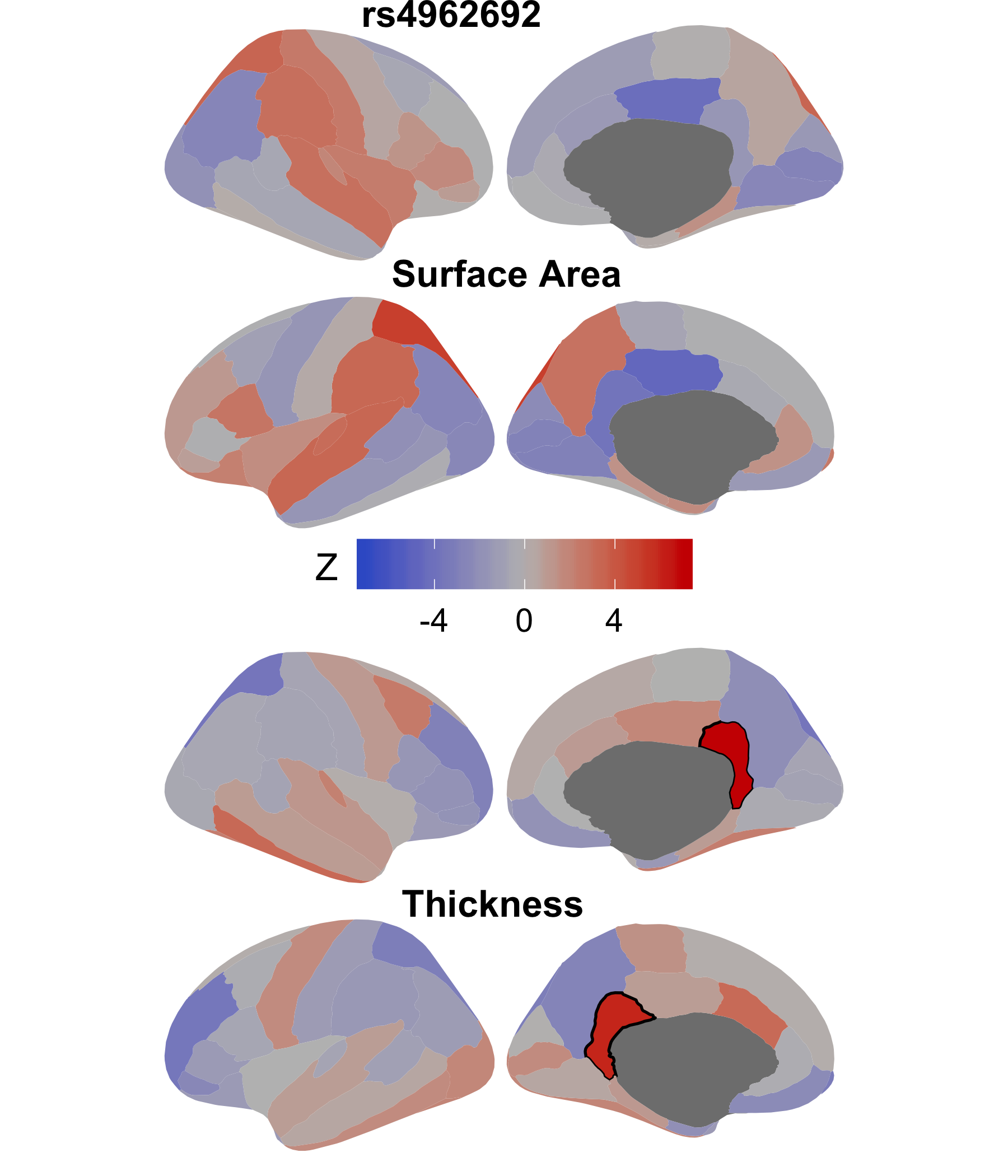

Supplement: Supplementary file 17 — Supplementary Data 14 [file 41467_2020_17368_MOESM17_ESM.gz › BrainMaps/most_dk_thick/BrainMap013_rs4962692.png]

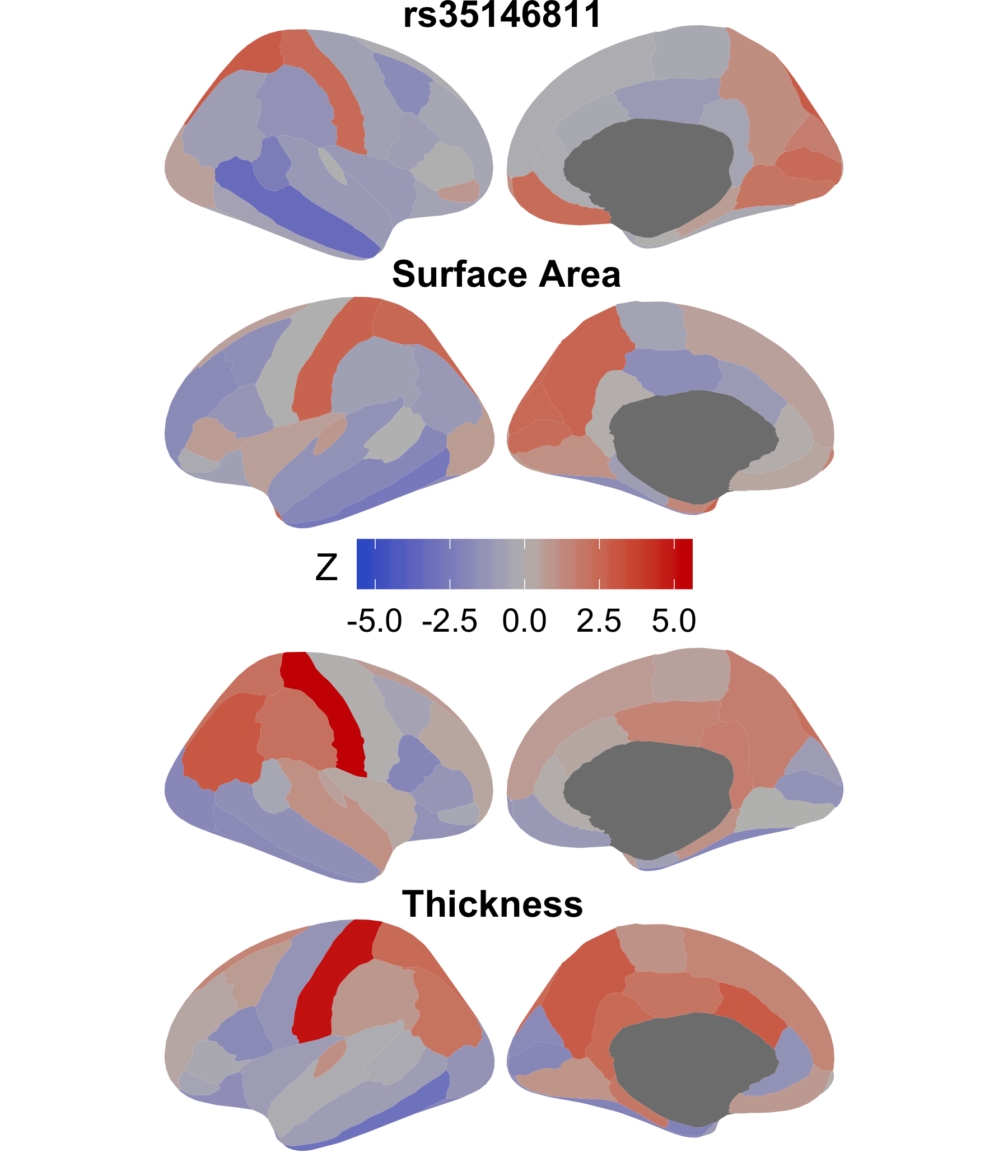

Supplement: Supplementary file 17 — Supplementary Data 14 [file 41467_2020_17368_MOESM17_ESM.gz › BrainMaps/most_dk_thick/BrainMap038_rs35146811.png]

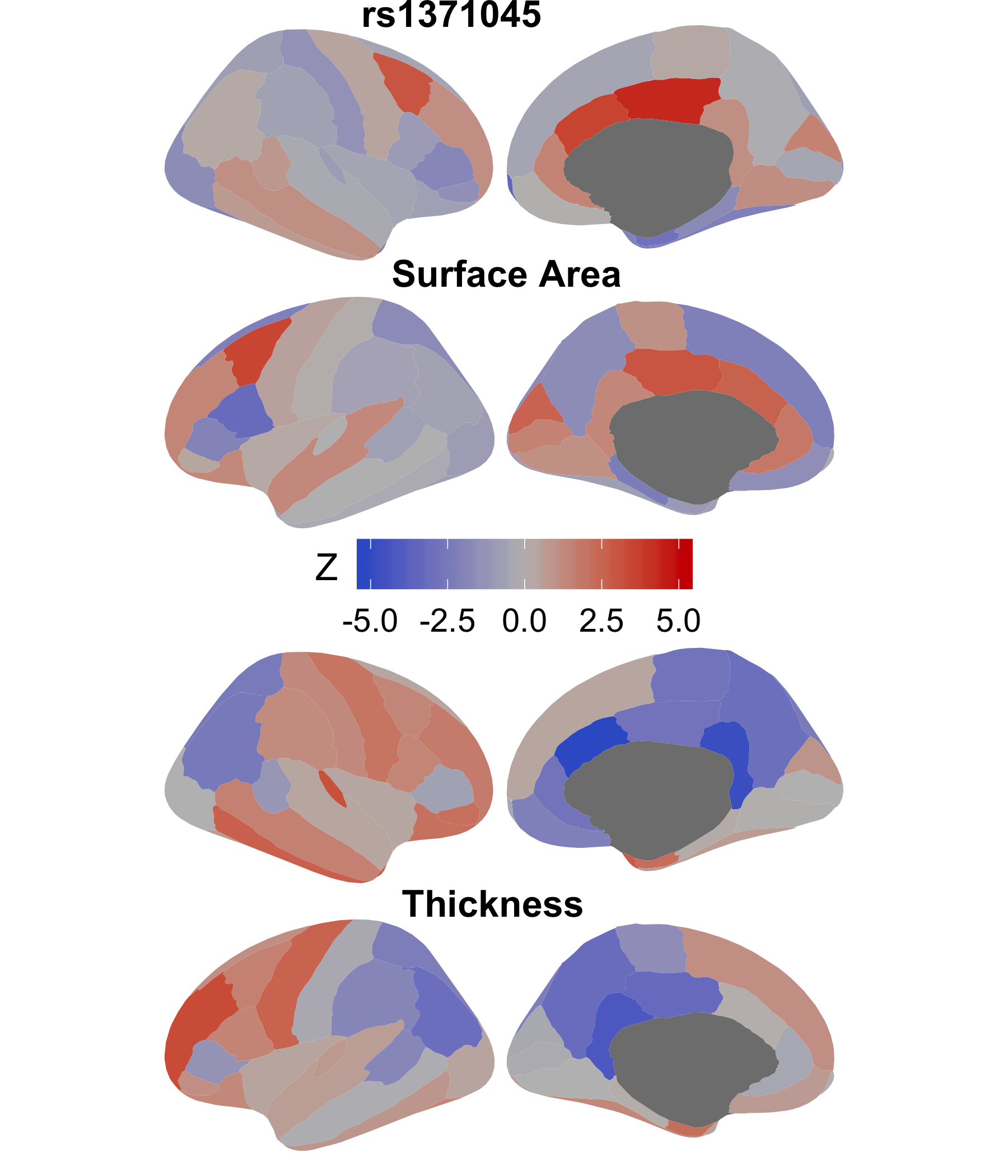

Supplement: Supplementary file 17 — Supplementary Data 14 [file 41467_2020_17368_MOESM17_ESM.gz › BrainMaps/most_dk_thick/BrainMap023_rs1371045.png]

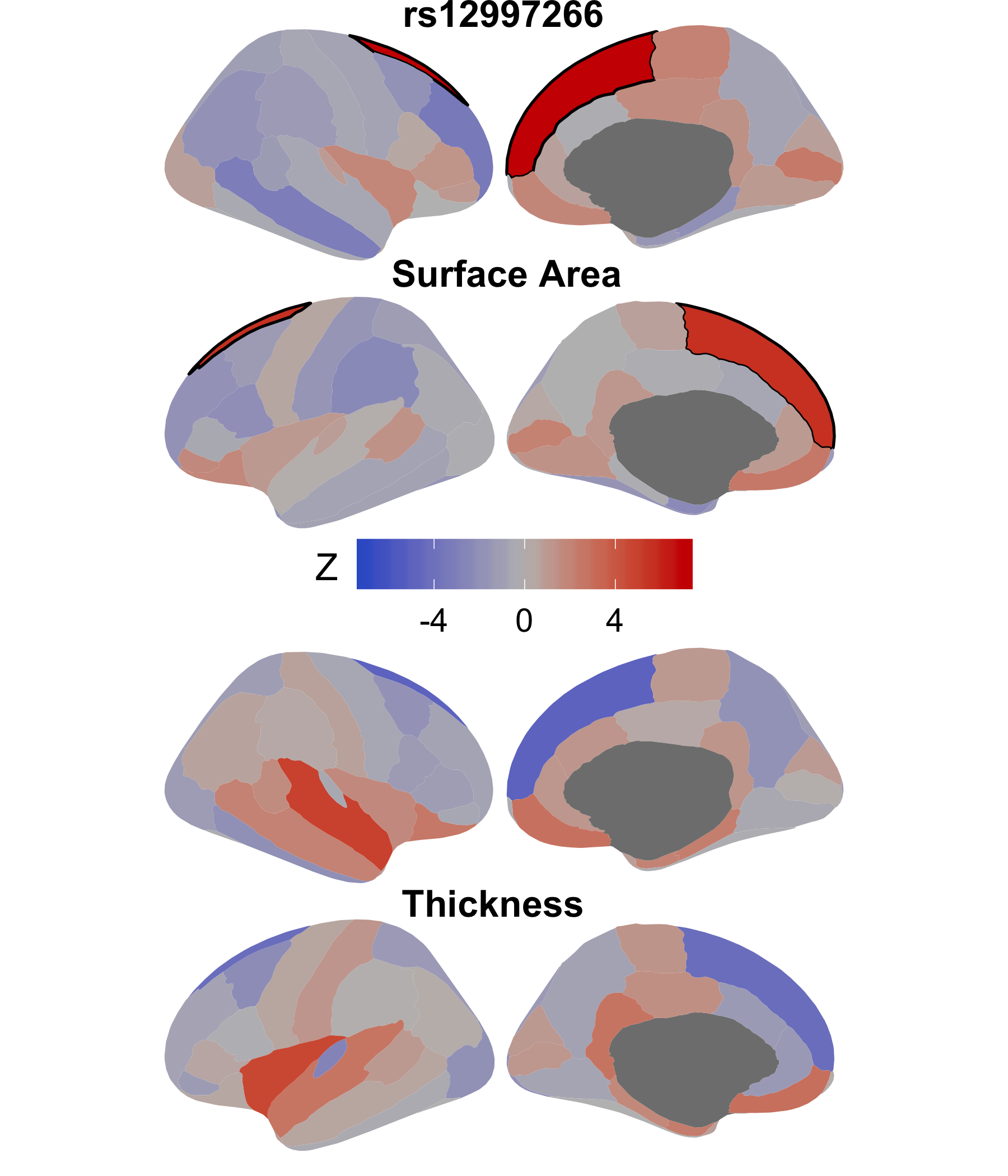

Supplement: Supplementary file 17 — Supplementary Data 14 [file 41467_2020_17368_MOESM17_ESM.gz › BrainMaps/most_dk_thick/BrainMap029_rs12997266.png]

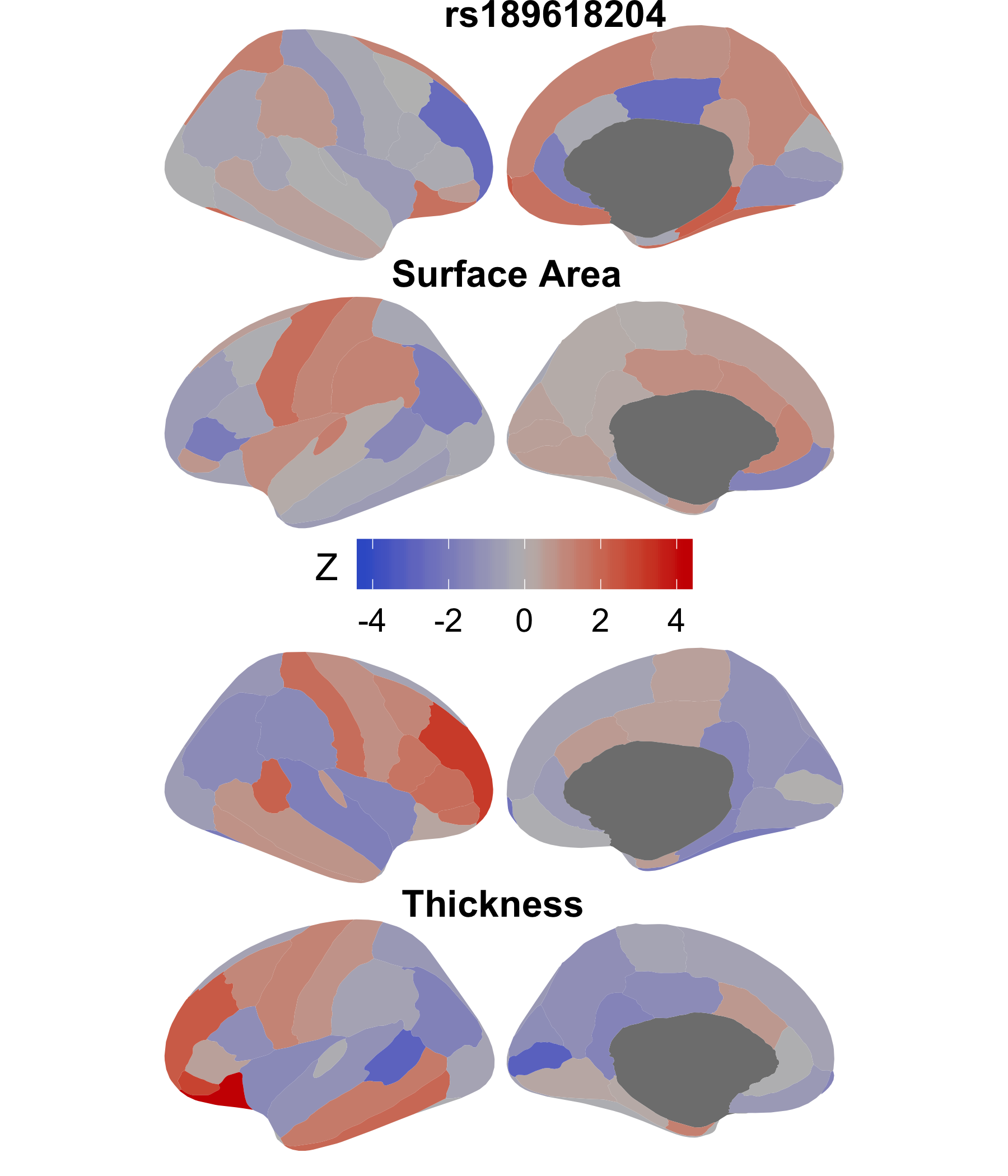

Supplement: Supplementary file 17 — Supplementary Data 14 [file 41467_2020_17368_MOESM17_ESM.gz › BrainMaps/most_dk_thick/BrainMap066_rs189618204.png]

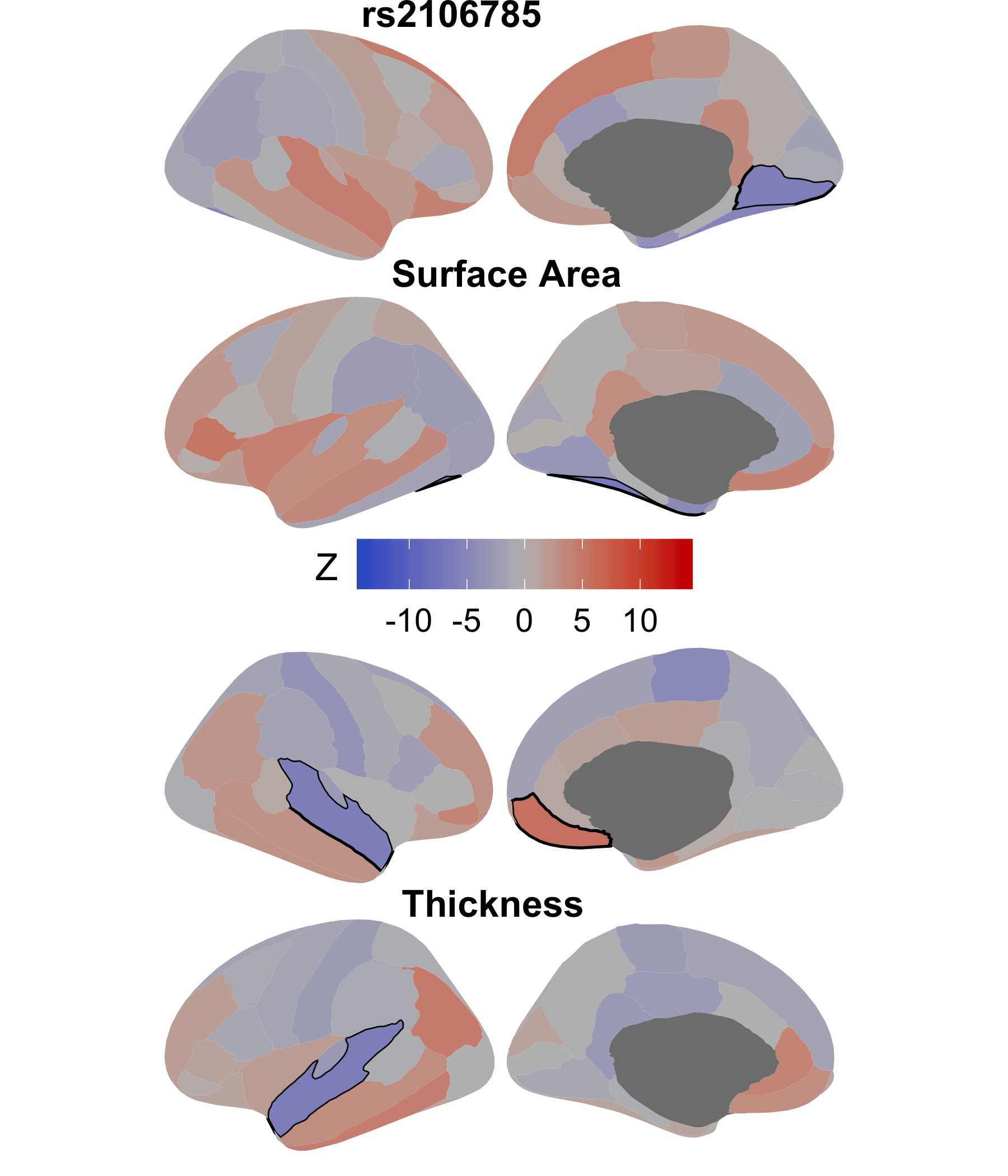

Supplement: Supplementary file 17 — Supplementary Data 14 [file 41467_2020_17368_MOESM17_ESM.gz › BrainMaps/most_dk_thick/BrainMap007_rs2106785.png]

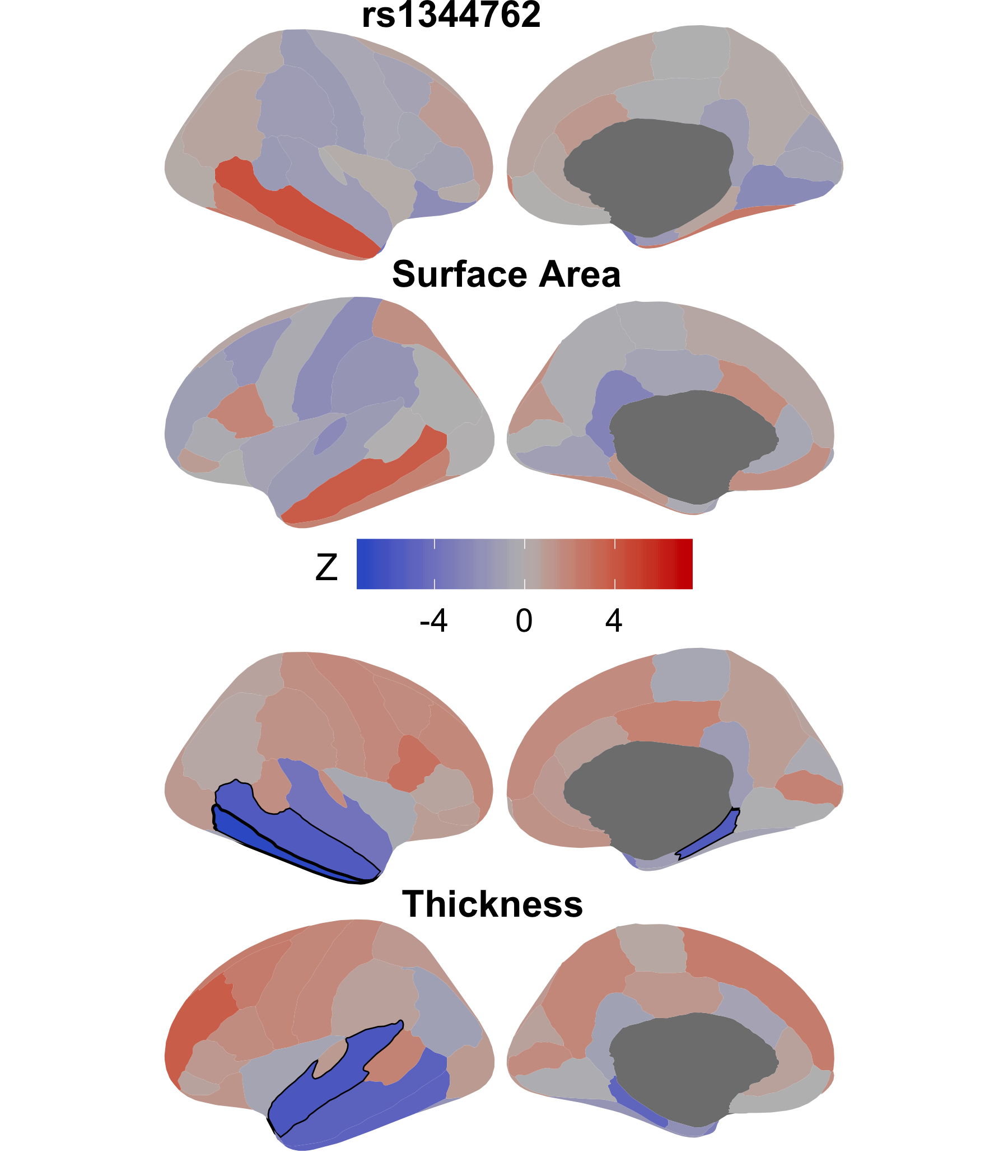

Supplement: Supplementary file 17 — Supplementary Data 14 [file 41467_2020_17368_MOESM17_ESM.gz › BrainMaps/most_dk_thick/BrainMap009_rs1344762.png]

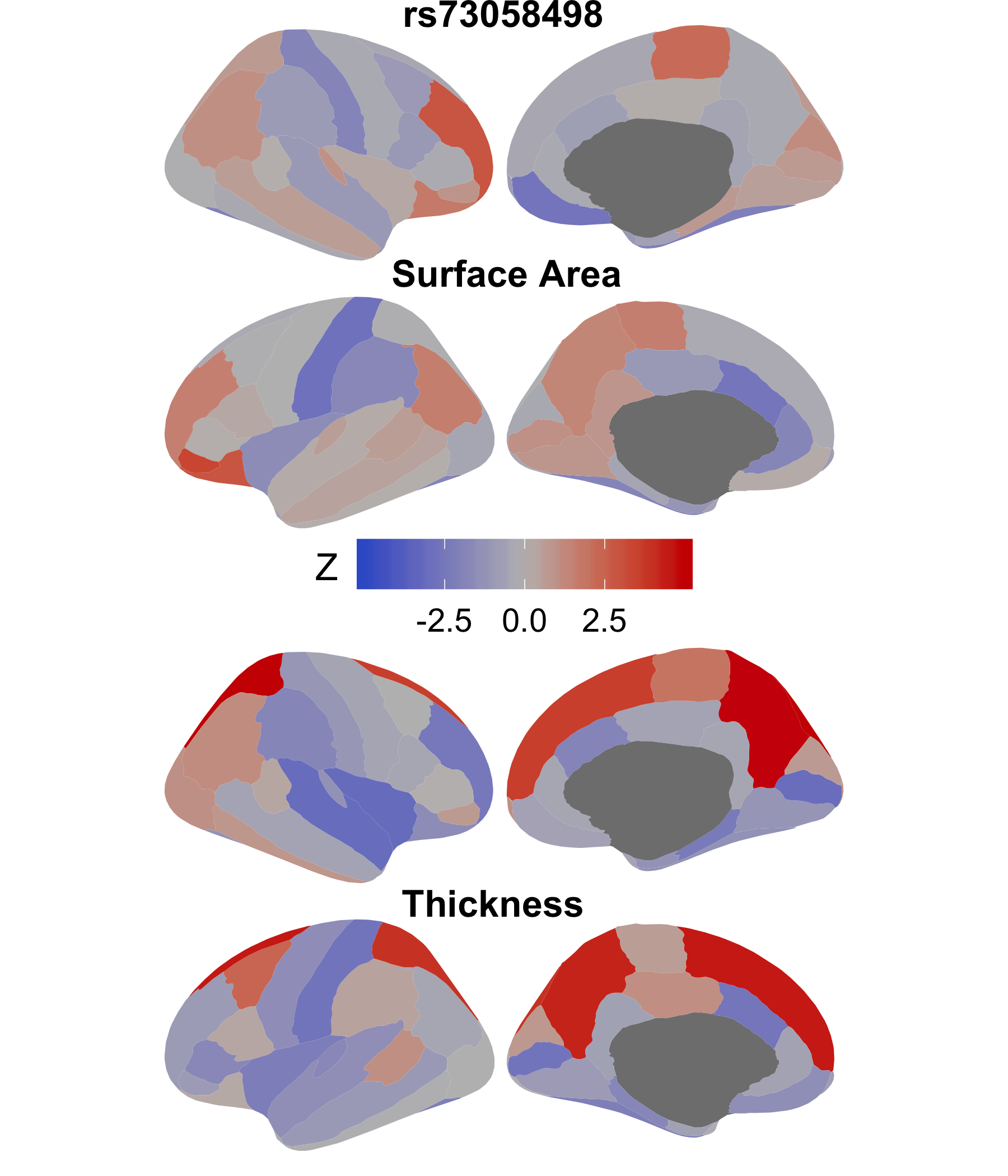

Supplement: Supplementary file 17 — Supplementary Data 14 [file 41467_2020_17368_MOESM17_ESM.gz › BrainMaps/most_dk_thick/BrainMap048_rs73058498.png]

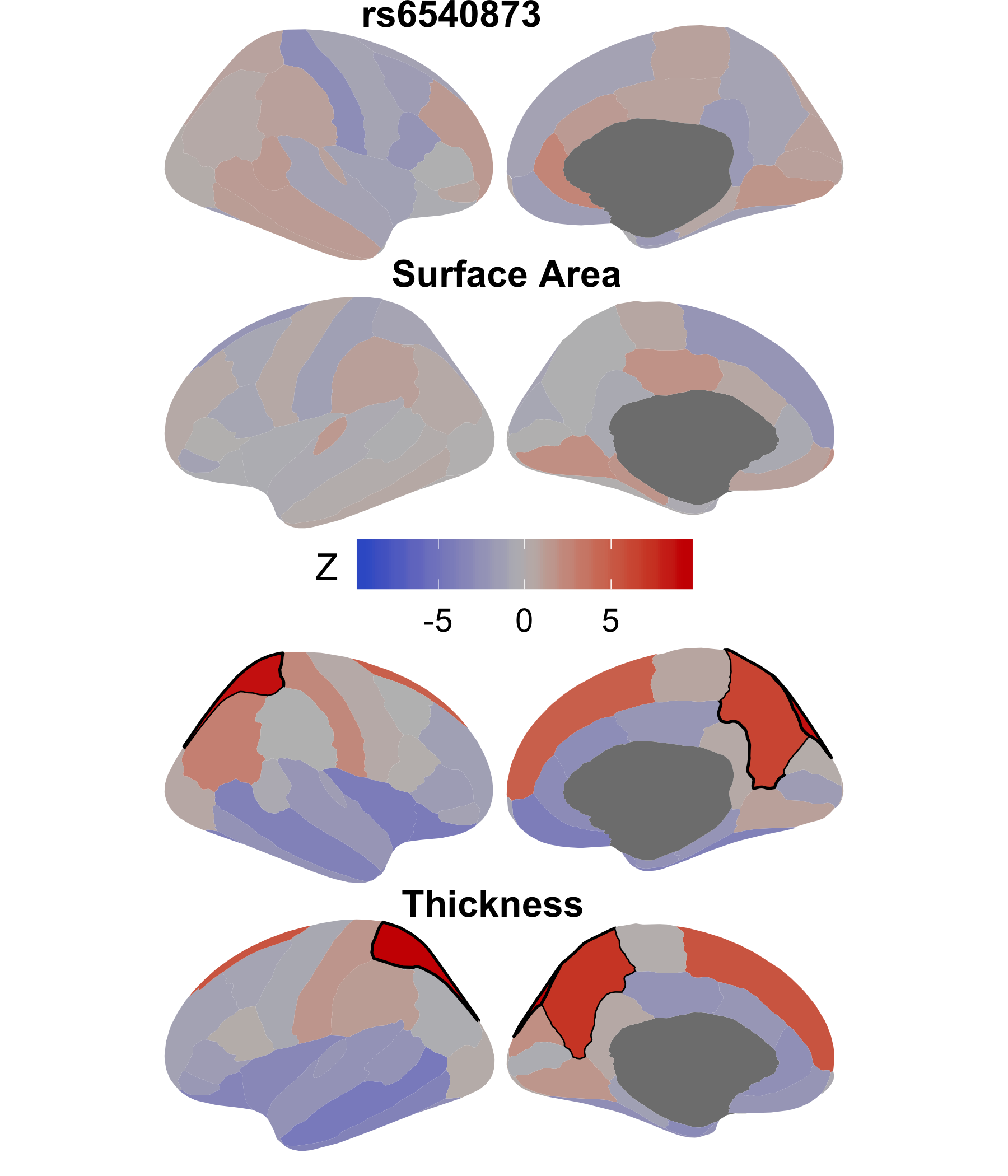

Supplement: Supplementary file 17 — Supplementary Data 14 [file 41467_2020_17368_MOESM17_ESM.gz › BrainMaps/most_dk_thick/BrainMap008_rs6540873.png]

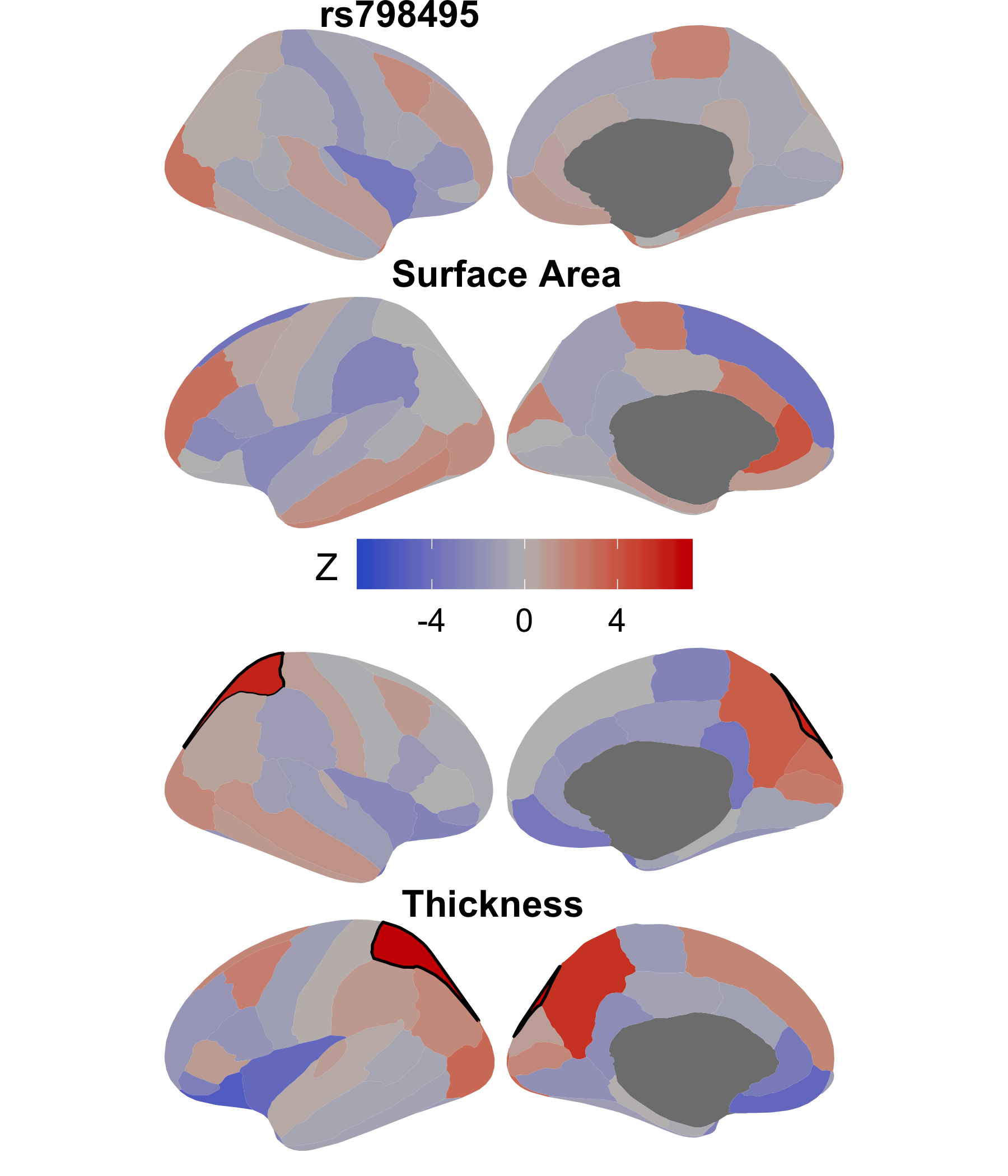

Supplement: Supplementary file 17 — Supplementary Data 14 [file 41467_2020_17368_MOESM17_ESM.gz › BrainMaps/most_dk_thick/BrainMap024_rs798495.png]

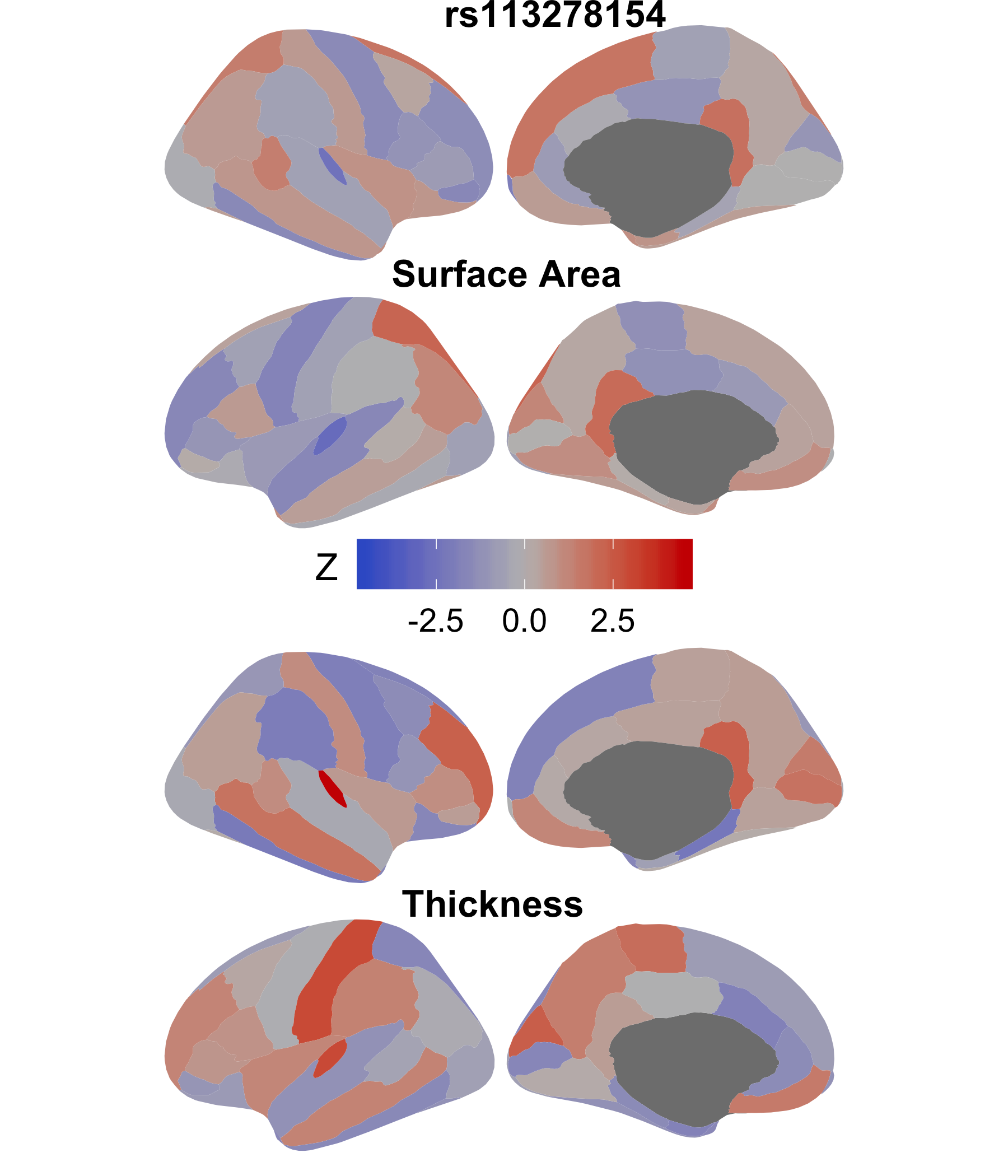

Supplement: Supplementary file 17 — Supplementary Data 14 [file 41467_2020_17368_MOESM17_ESM.gz › BrainMaps/most_dk_thick/BrainMap052_rs113278154.png]

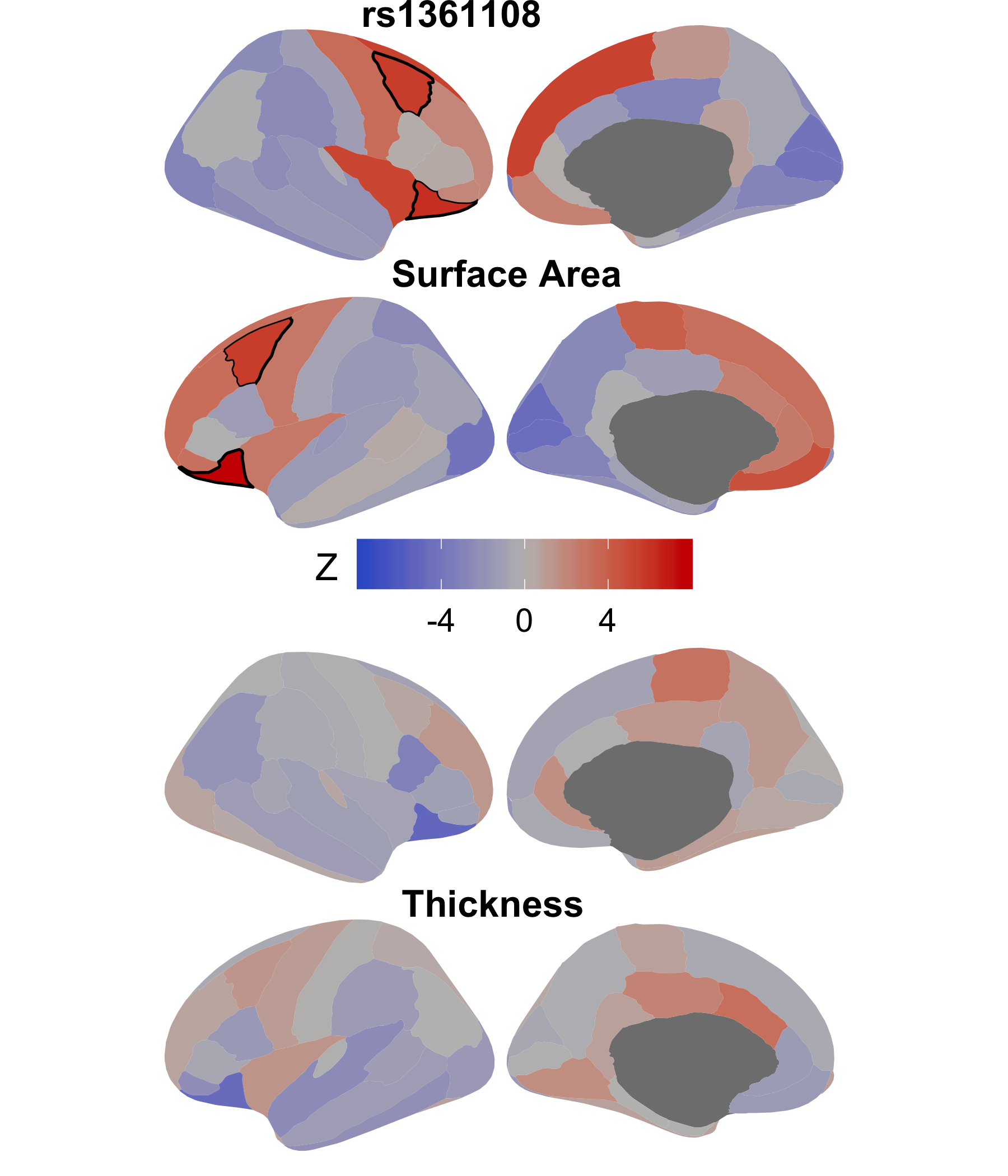

Supplement: Supplementary file 17 — Supplementary Data 14 [file 41467_2020_17368_MOESM17_ESM.gz › BrainMaps/most_dk_thick/BrainMap050_rs1361108.png]

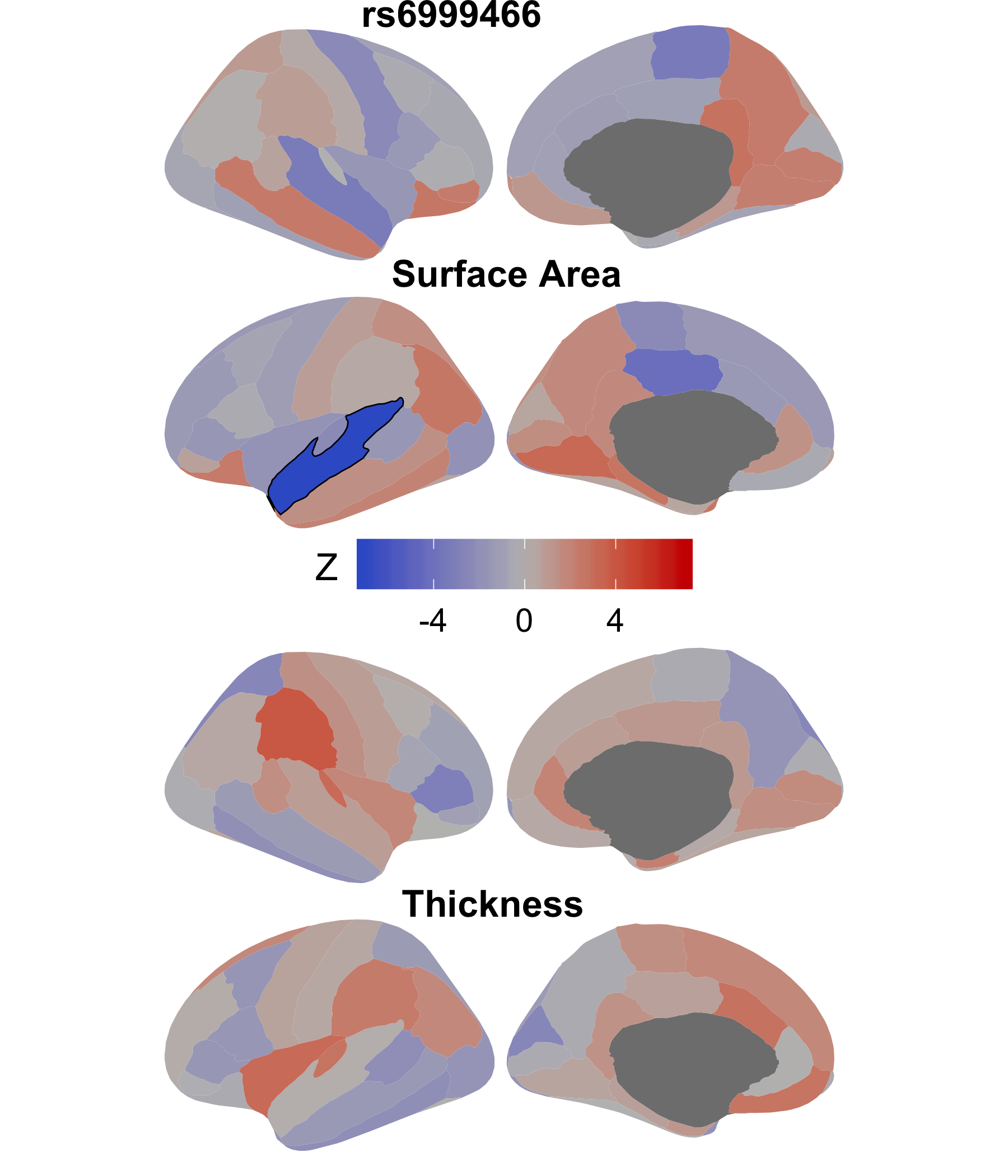

Supplement: Supplementary file 17 — Supplementary Data 14 [file 41467_2020_17368_MOESM17_ESM.gz › BrainMaps/most_dk_thick/BrainMap063_rs6999466.png]

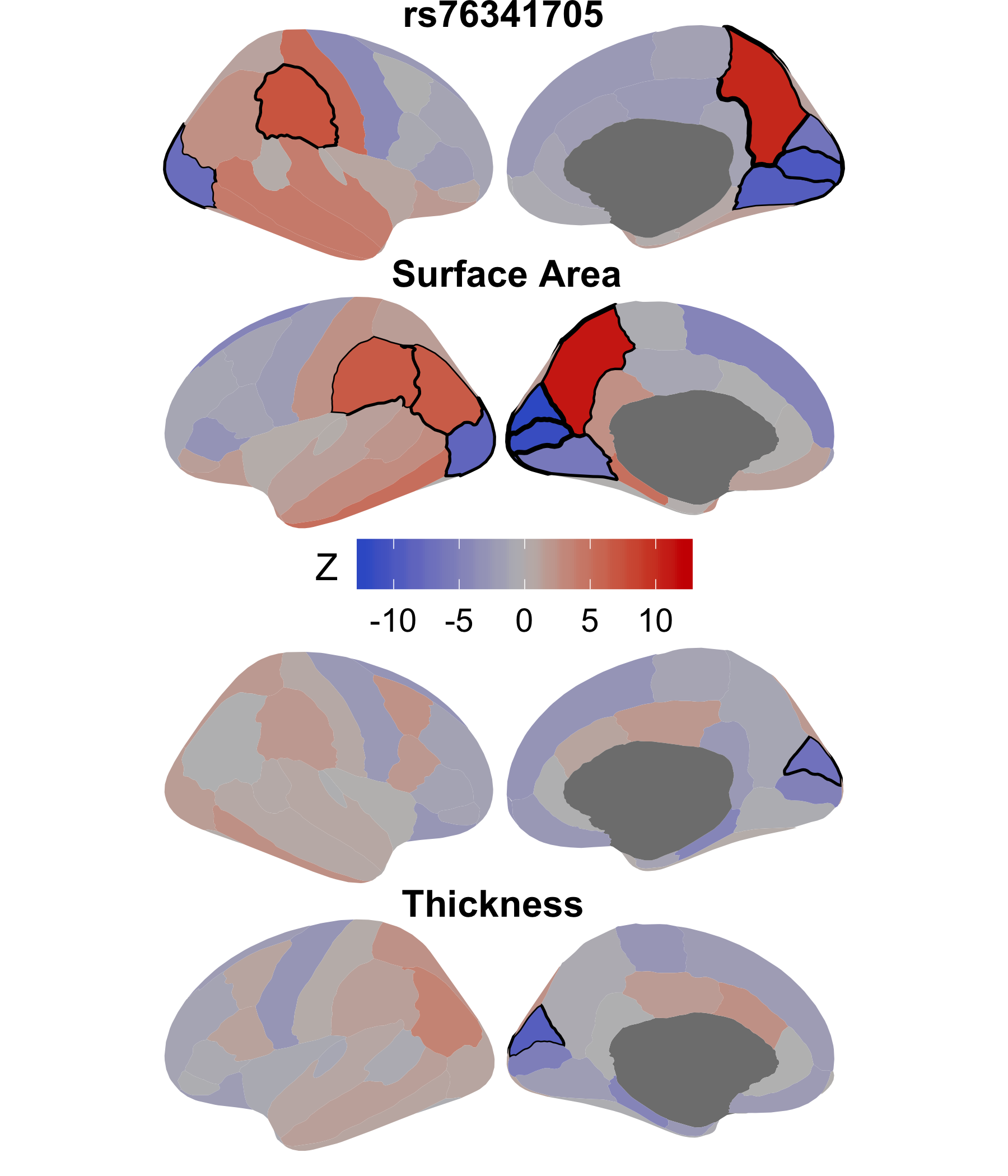

Supplement: Supplementary file 17 — Supplementary Data 14 [file 41467_2020_17368_MOESM17_ESM.gz › BrainMaps/most_dk_thick/BrainMap005_rs76341705.png]

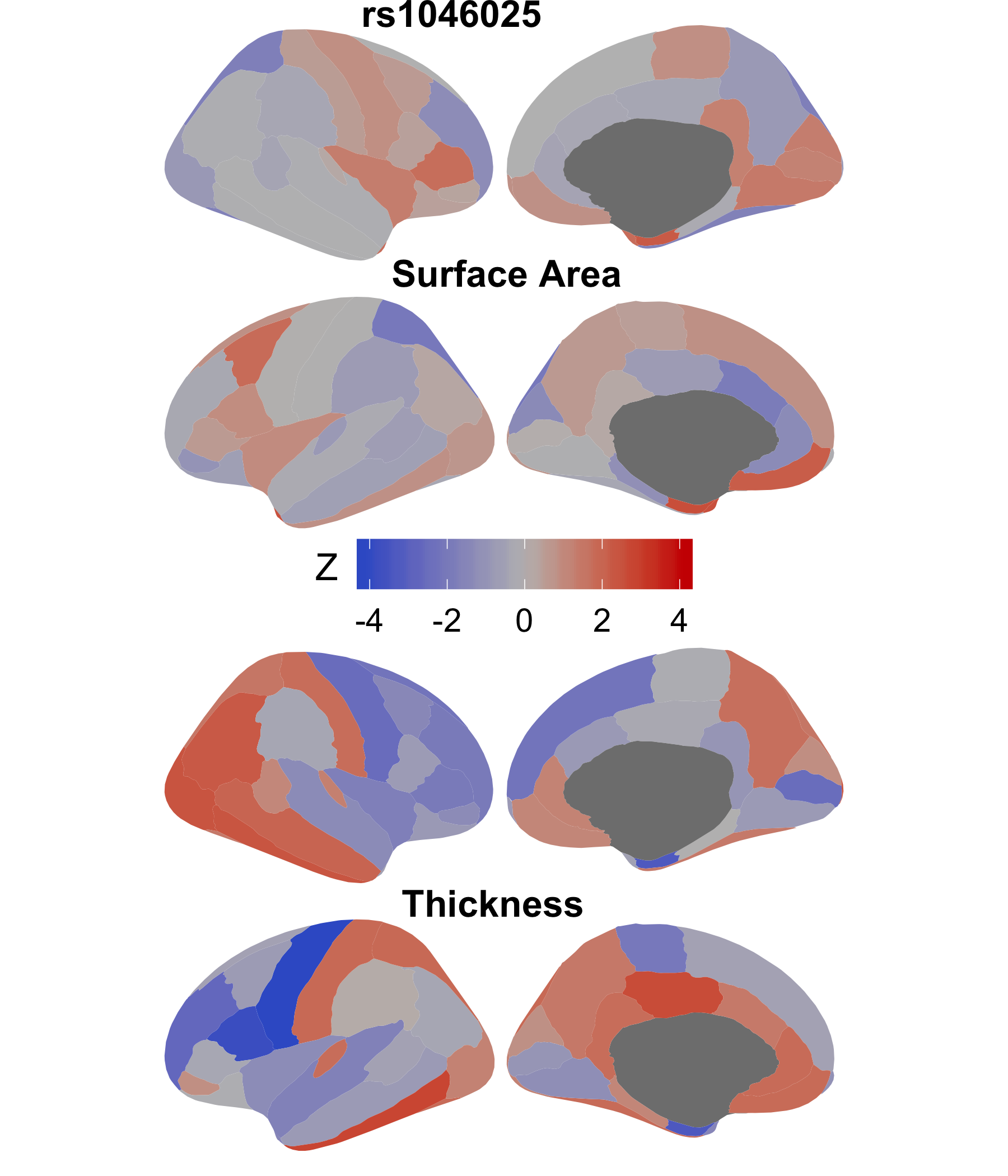

Supplement: Supplementary file 17 — Supplementary Data 14 [file 41467_2020_17368_MOESM17_ESM.gz › BrainMaps/most_dk_thick/BrainMap047_rs1046025.png]

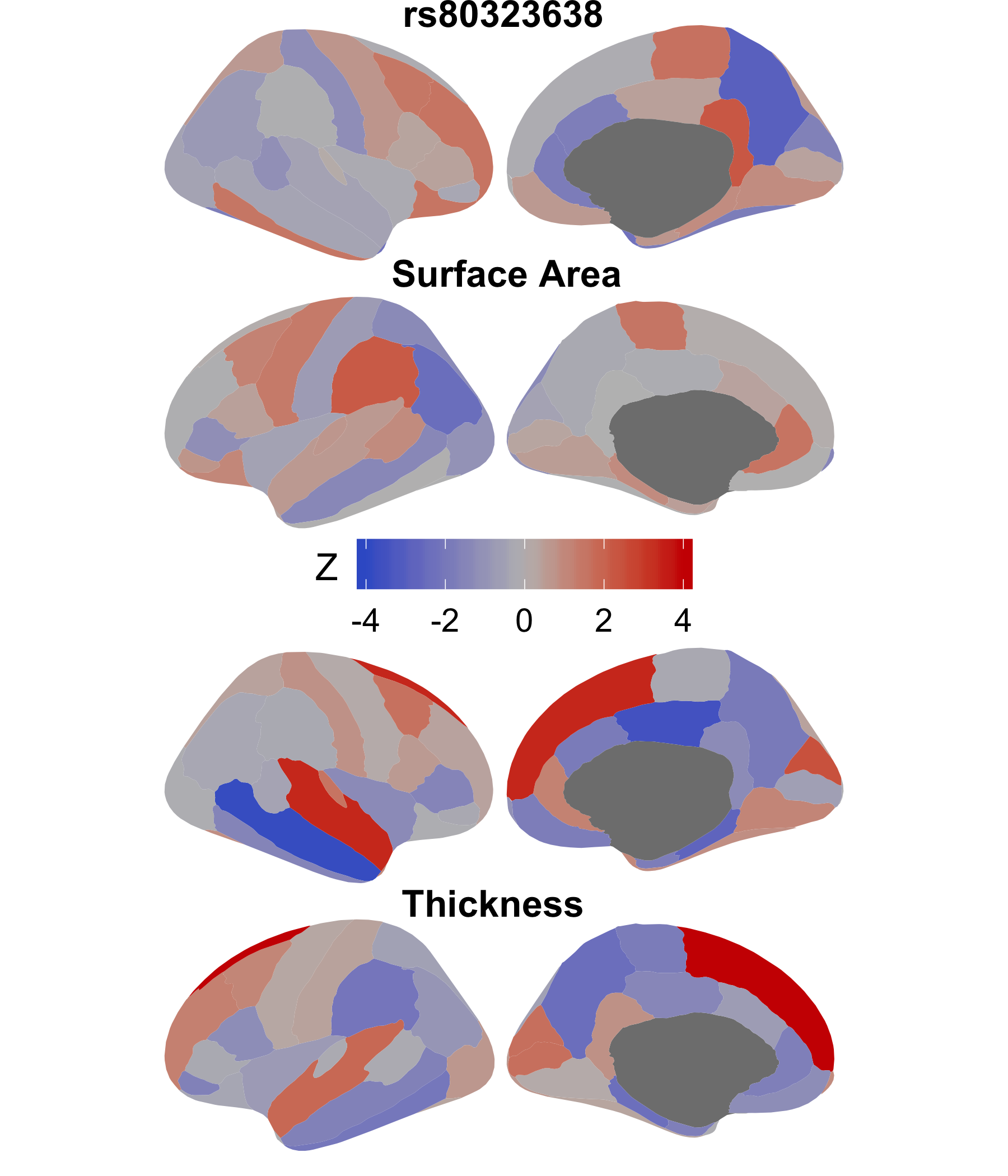

Supplement: Supplementary file 17 — Supplementary Data 14 [file 41467_2020_17368_MOESM17_ESM.gz › BrainMaps/most_dk_thick/BrainMap043_rs80323638.png]

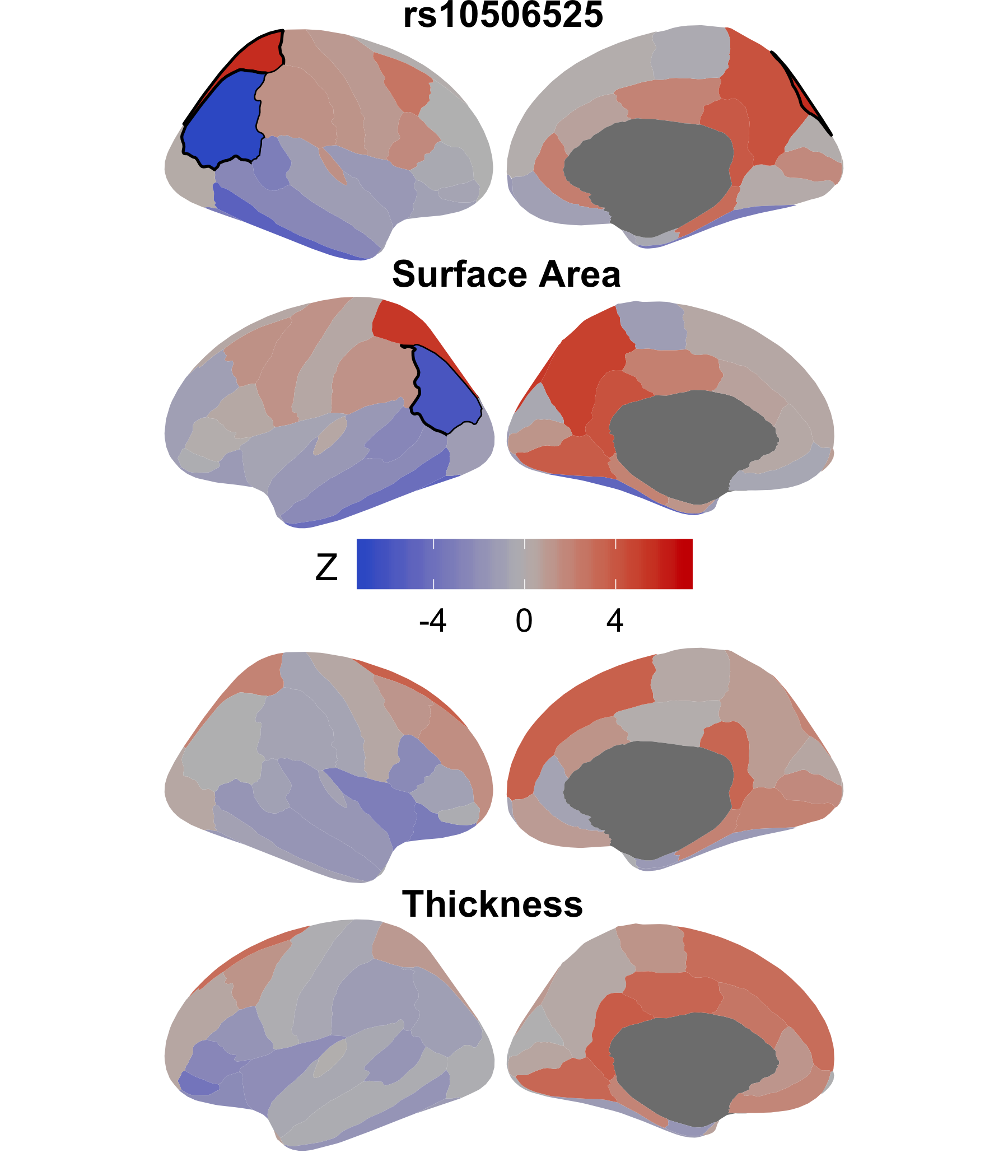

Supplement: Supplementary file 17 — Supplementary Data 14 [file 41467_2020_17368_MOESM17_ESM.gz › BrainMaps/most_dk_thick/BrainMap070_rs10506525.png]

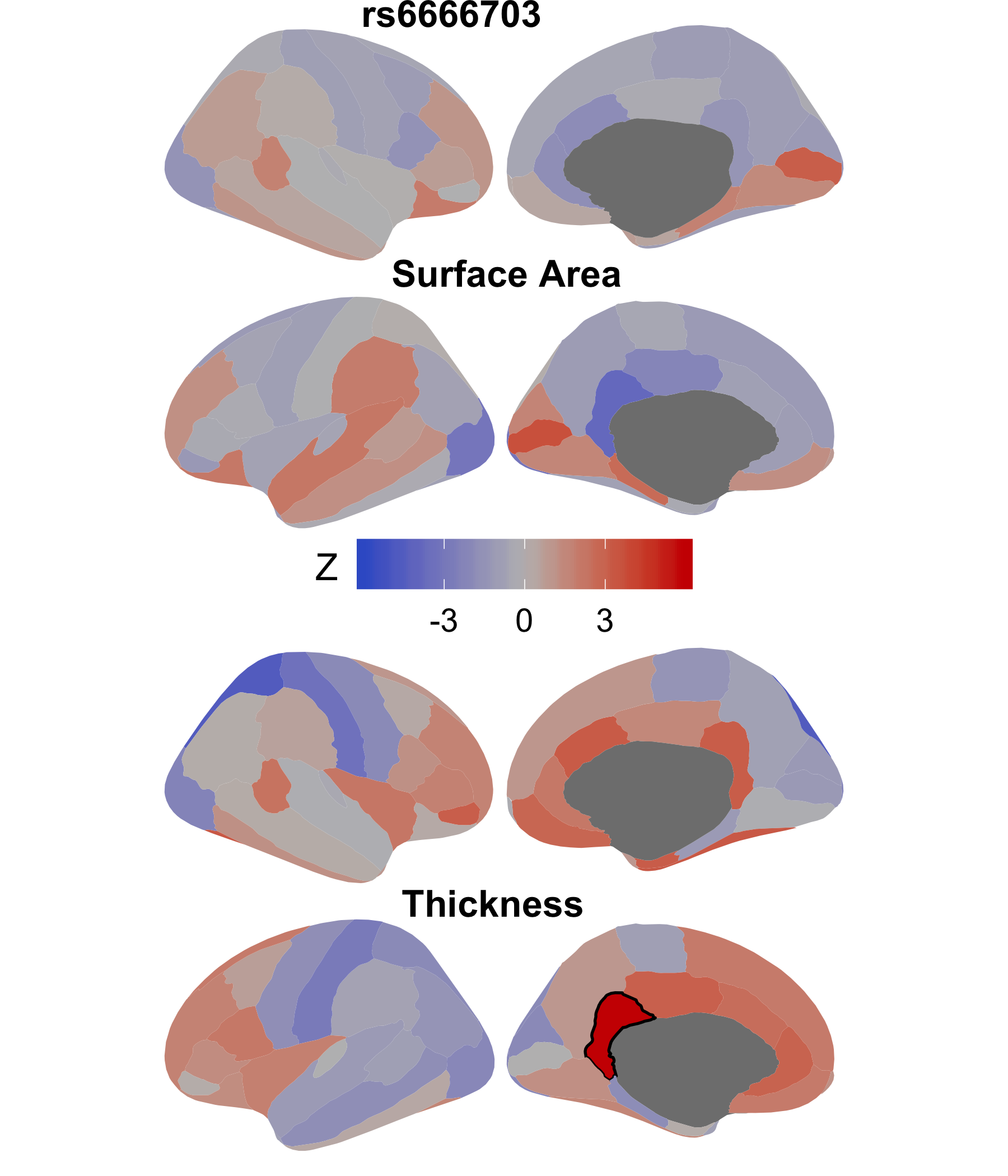

Supplement: Supplementary file 17 — Supplementary Data 14 [file 41467_2020_17368_MOESM17_ESM.gz › BrainMaps/most_dk_thick/BrainMap068_rs6666703.png]

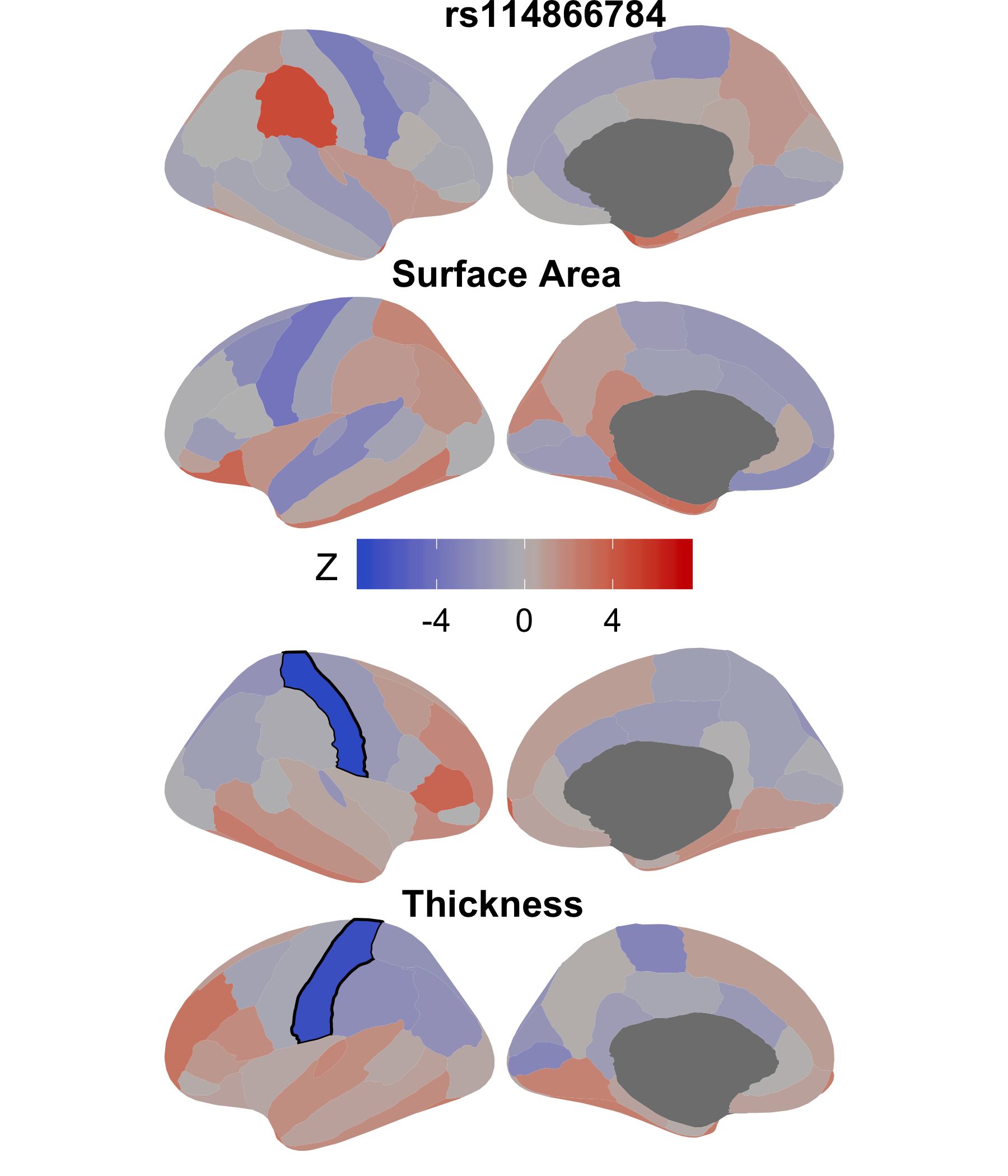

Supplement: Supplementary file 17 — Supplementary Data 14 [file 41467_2020_17368_MOESM17_ESM.gz › BrainMaps/most_dk_thick/BrainMap044_rs114866784.png]

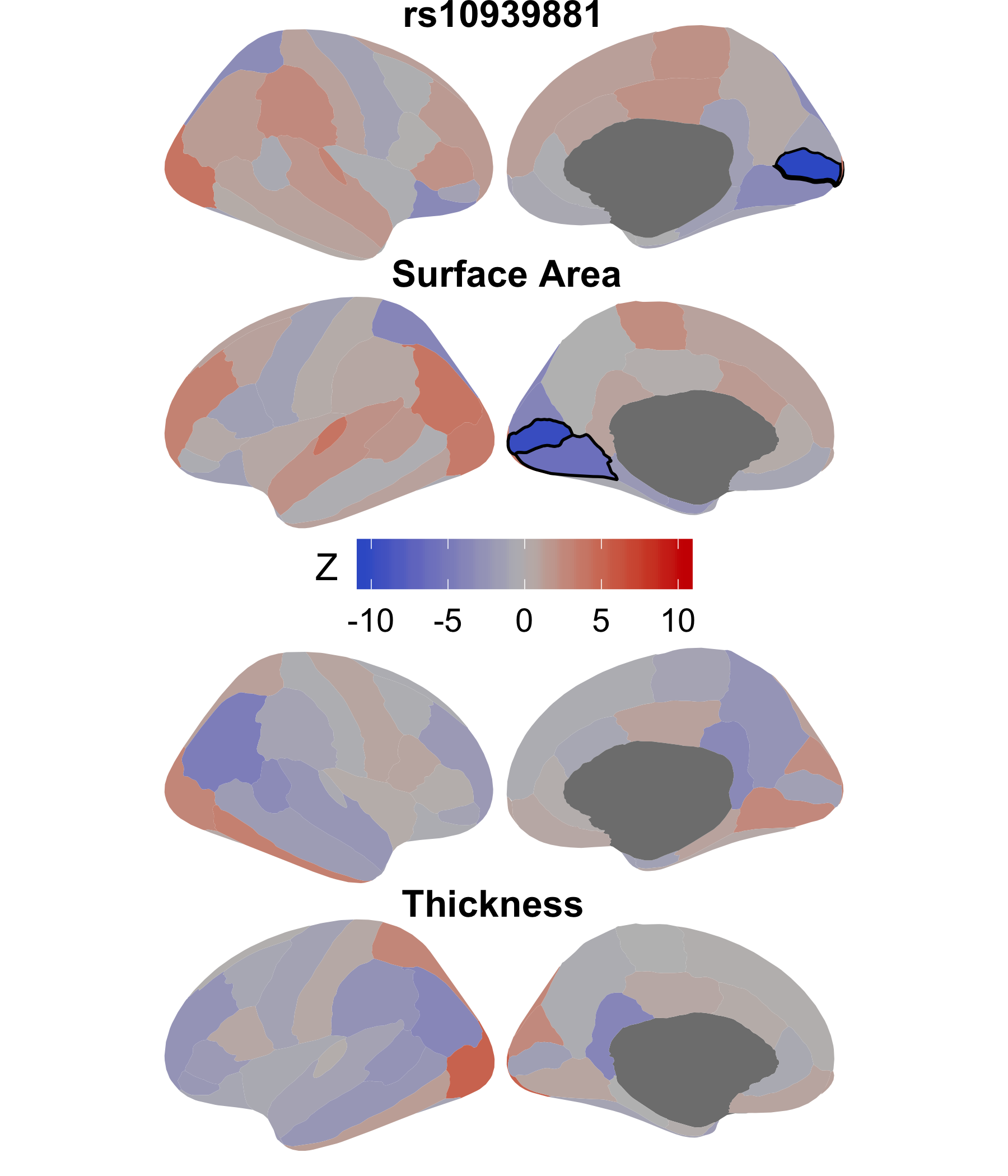

Supplement: Supplementary file 17 — Supplementary Data 14 [file 41467_2020_17368_MOESM17_ESM.gz › BrainMaps/most_dk_thick/BrainMap010_rs10939881.png]

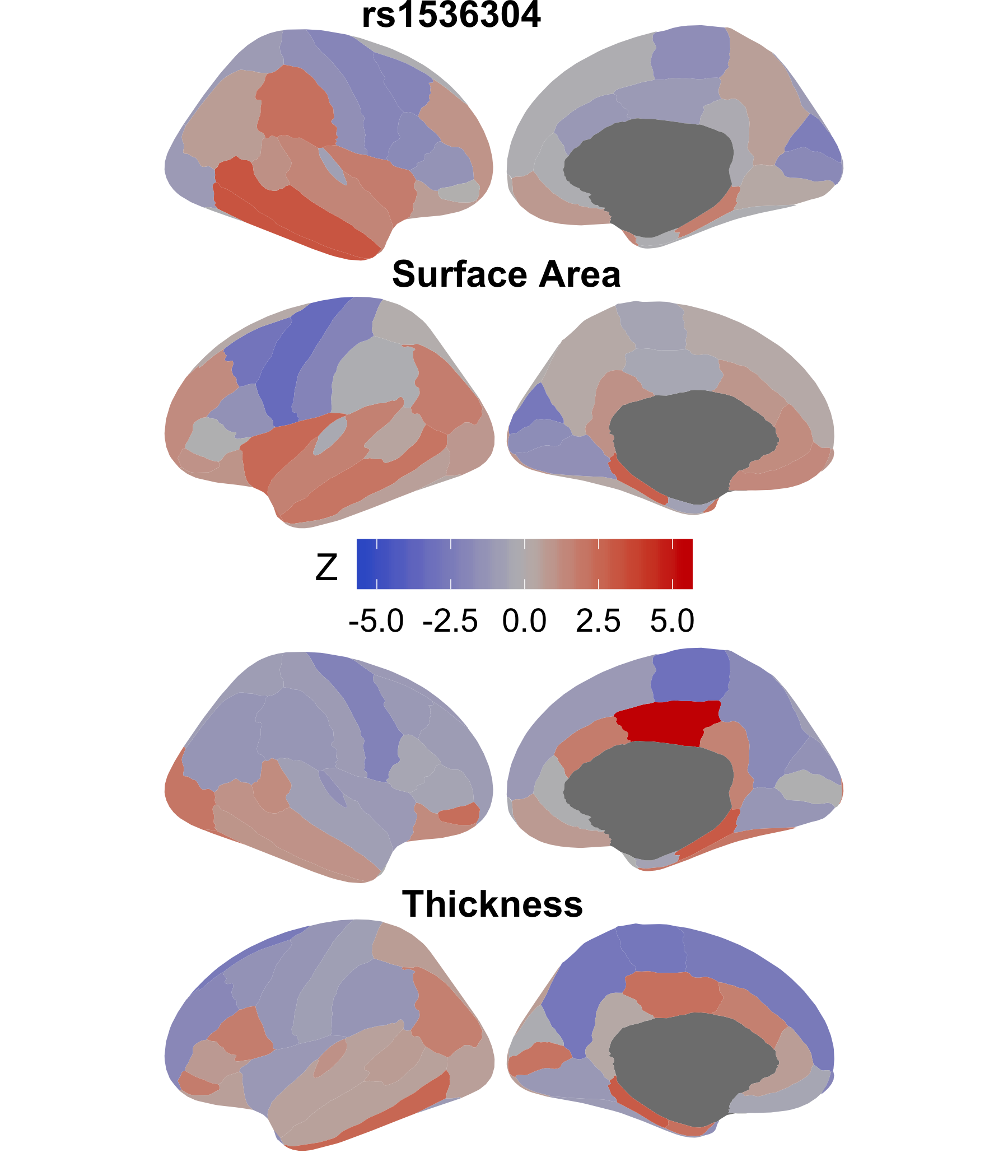

Supplement: Supplementary file 17 — Supplementary Data 14 [file 41467_2020_17368_MOESM17_ESM.gz › BrainMaps/most_dk_thick/BrainMap041_rs1536304.png]

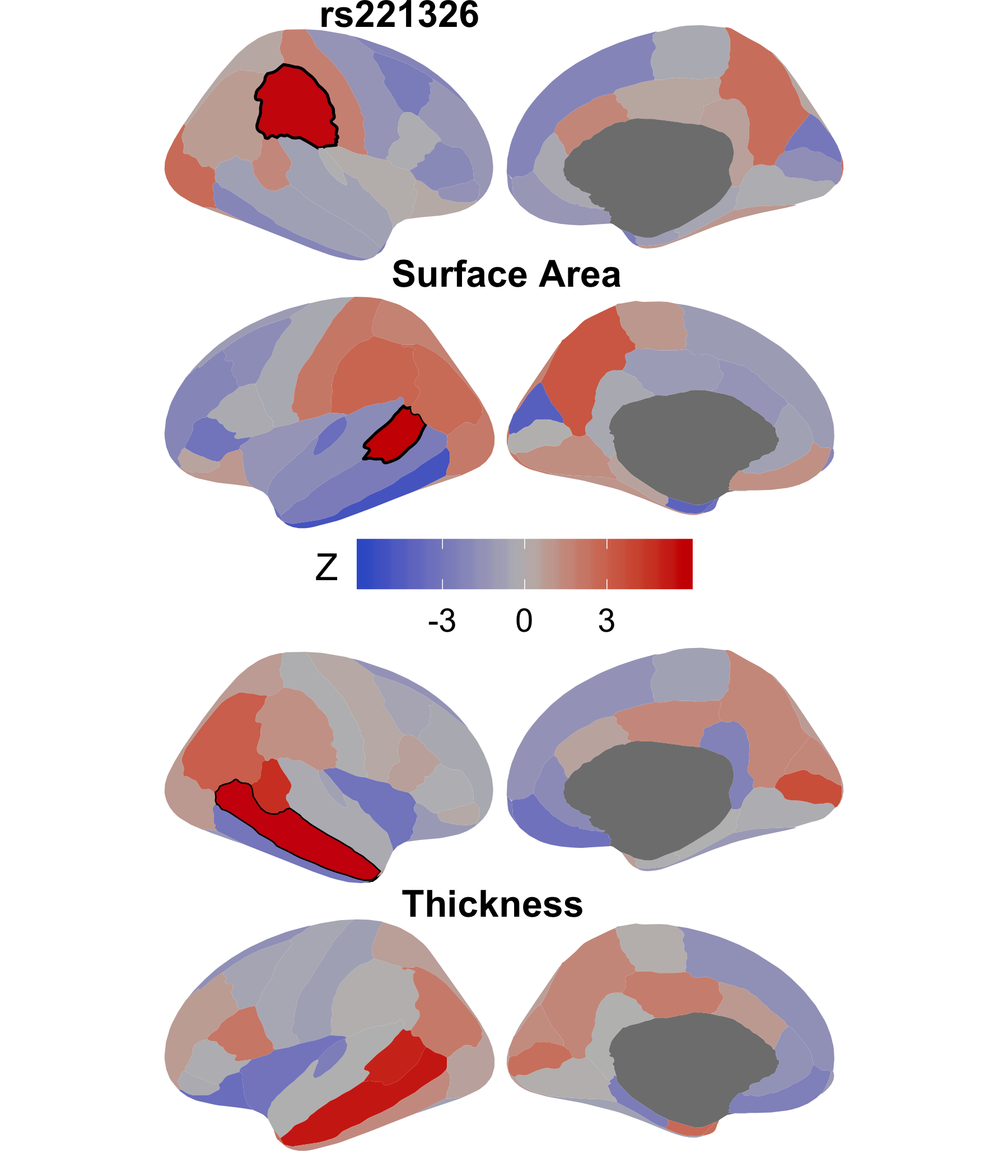

Supplement: Supplementary file 17 — Supplementary Data 14 [file 41467_2020_17368_MOESM17_ESM.gz › BrainMaps/most_dk_thick/BrainMap006_rs221326.png]

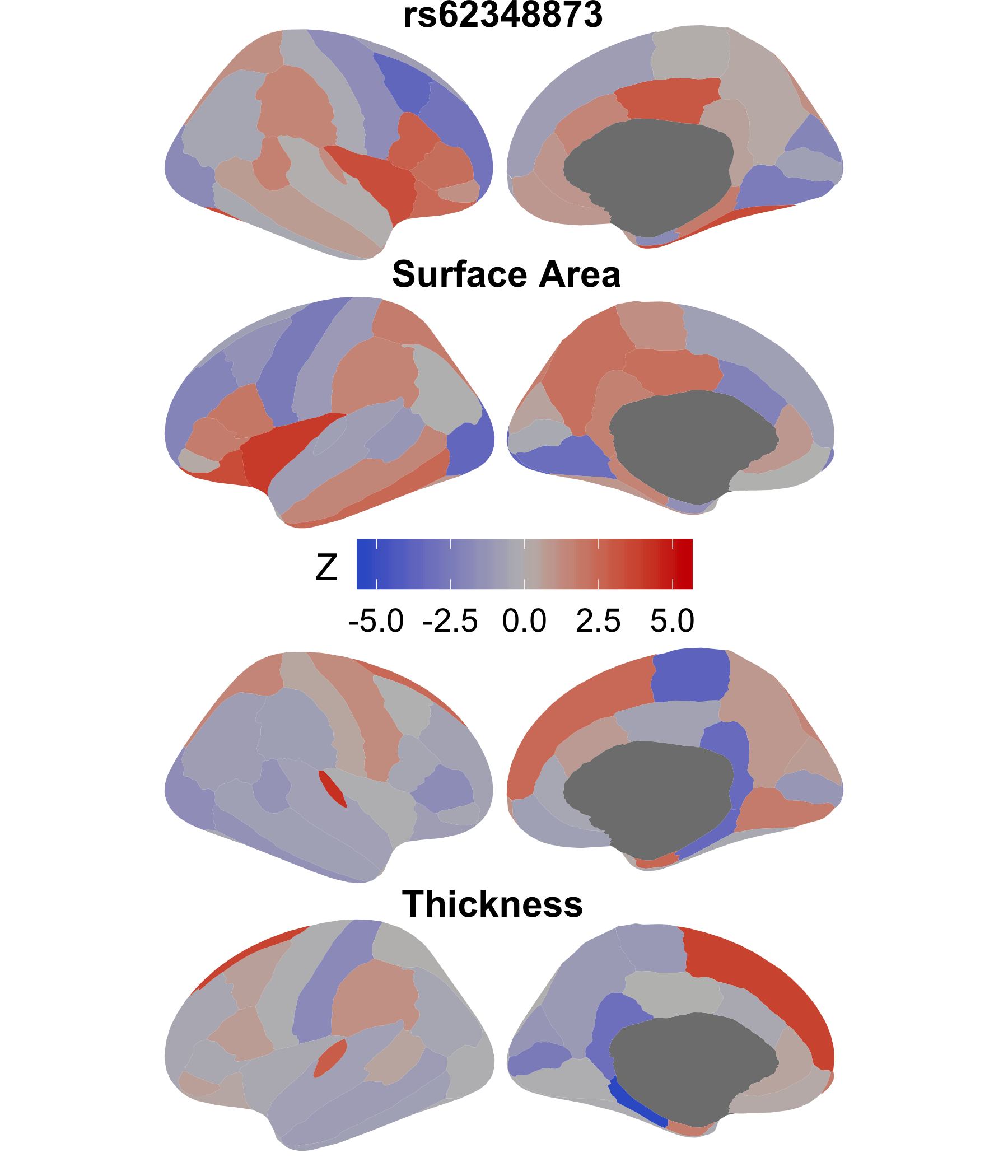

Supplement: Supplementary file 17 — Supplementary Data 14 [file 41467_2020_17368_MOESM17_ESM.gz › BrainMaps/most_dk_thick/BrainMap017_rs62348873.png]

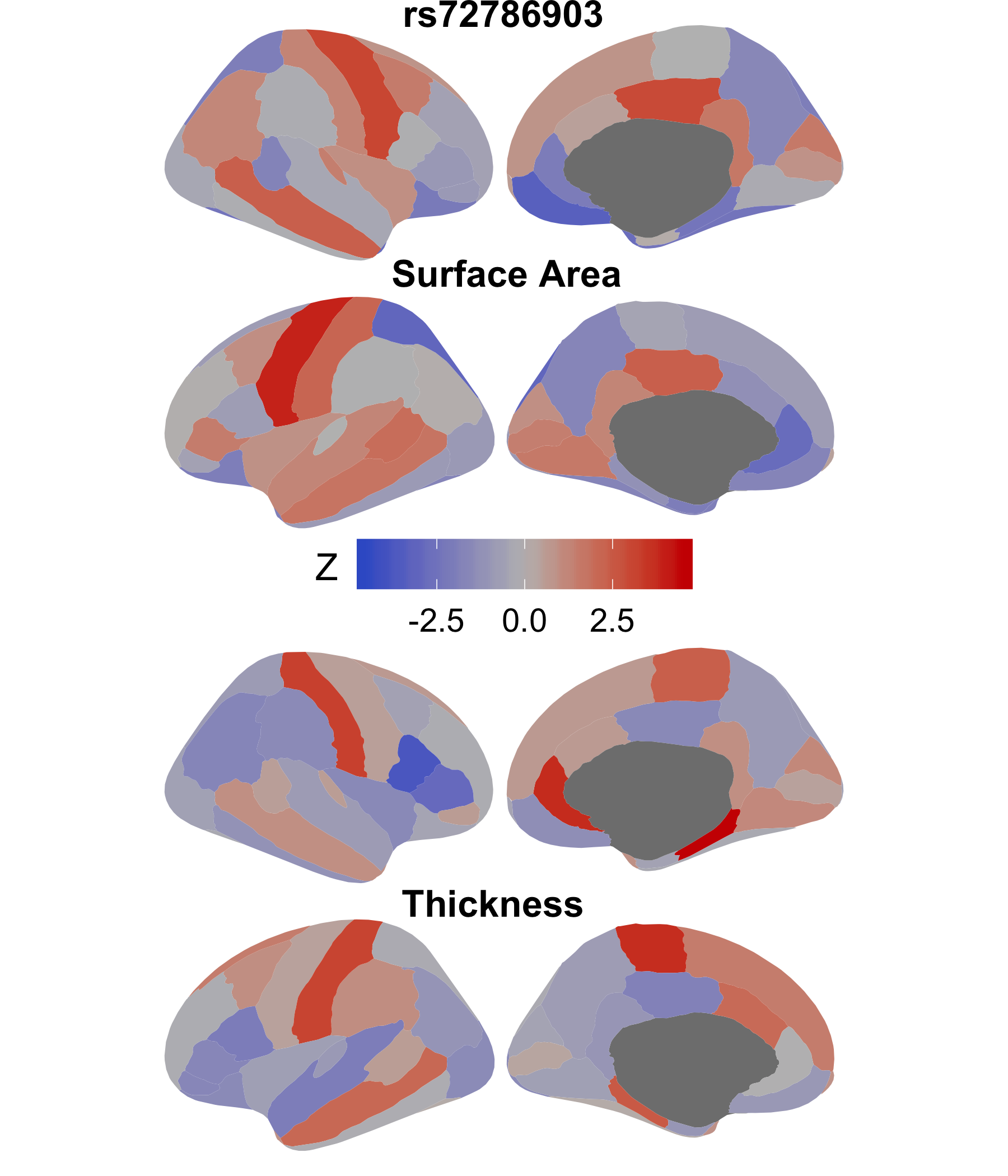

Supplement: Supplementary file 17 — Supplementary Data 14 [file 41467_2020_17368_MOESM17_ESM.gz › BrainMaps/most_dk_thick/BrainMap042_rs72786903.png]

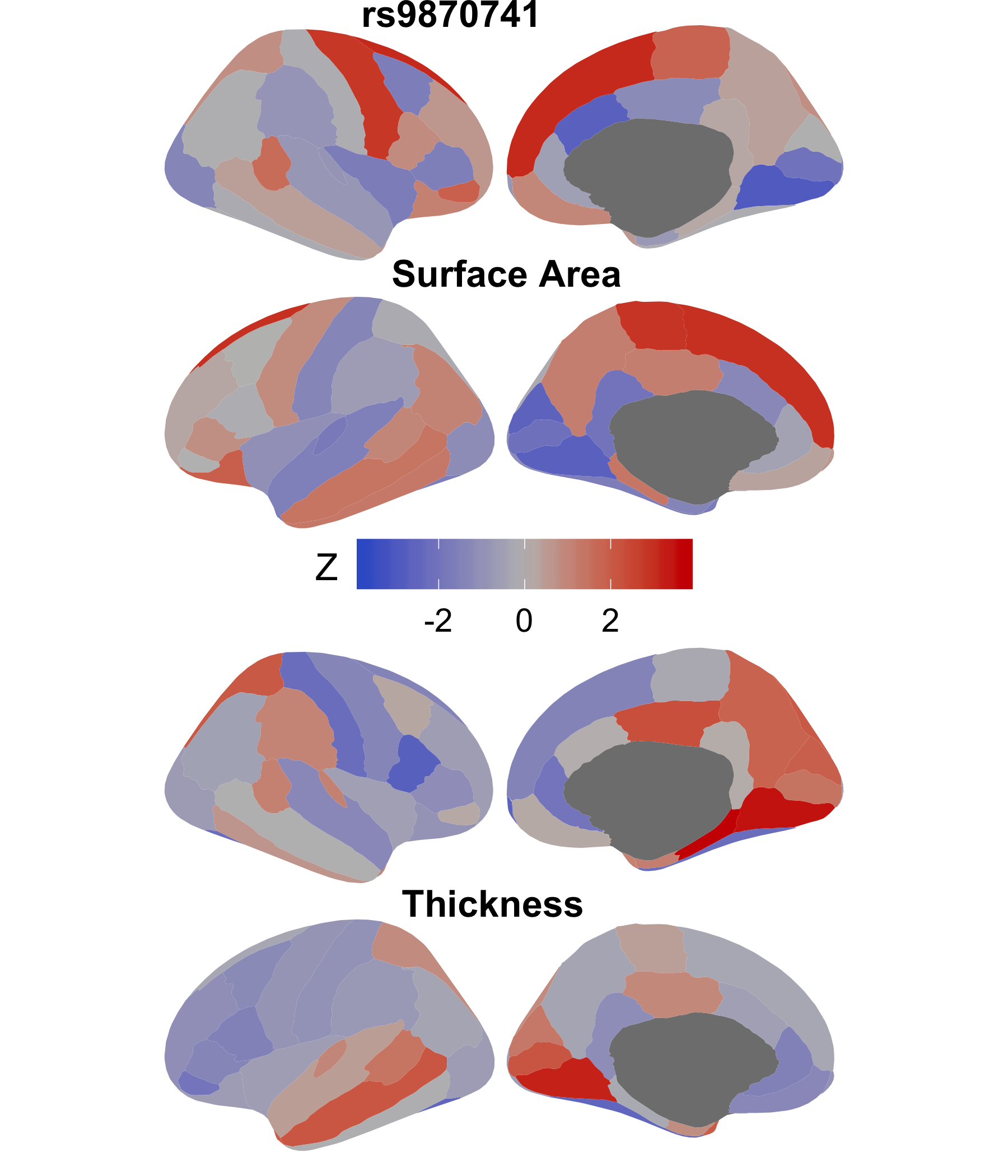

Supplement: Supplementary file 17 — Supplementary Data 14 [file 41467_2020_17368_MOESM17_ESM.gz › BrainMaps/most_dk_thick/BrainMap039_rs9870741.png]

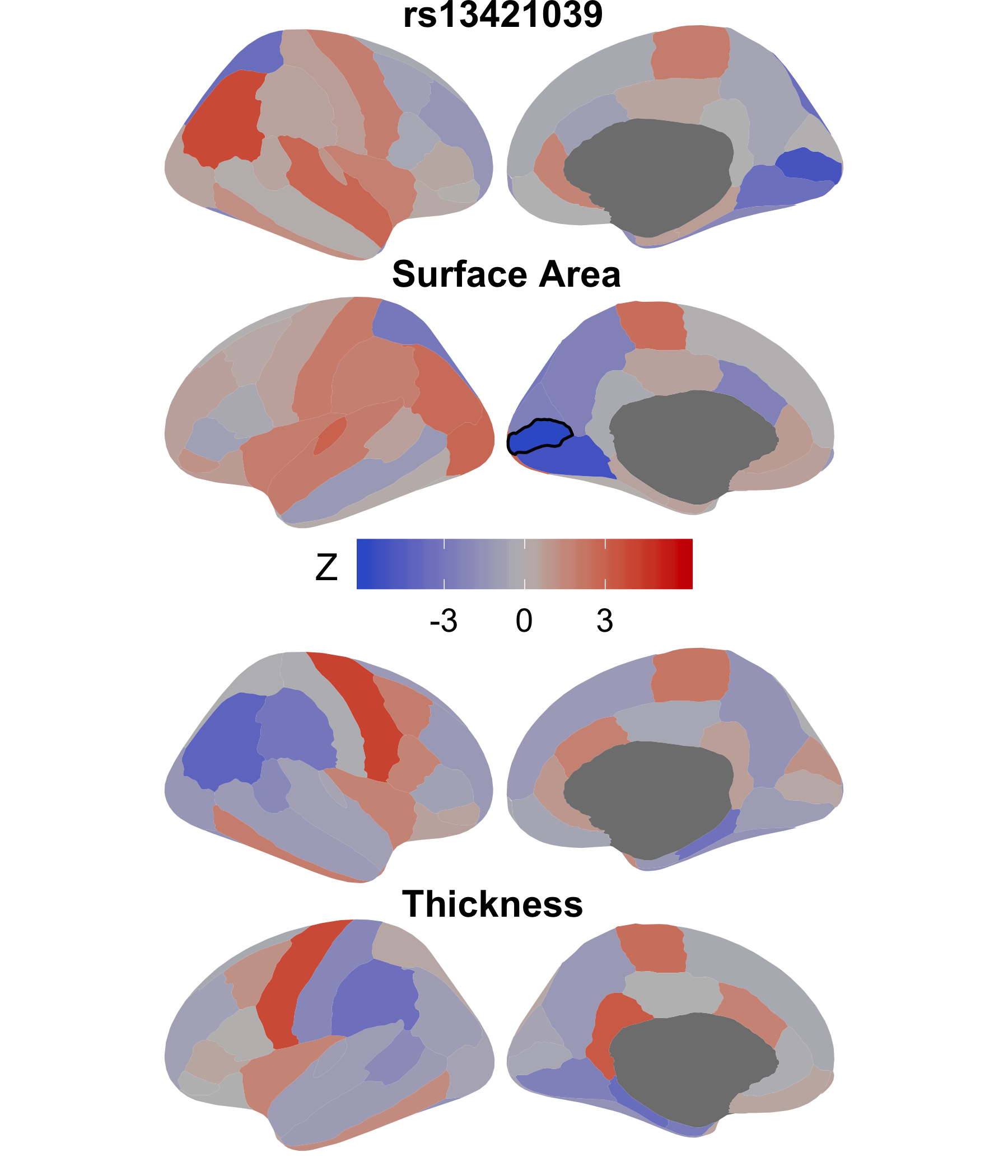

Supplement: Supplementary file 17 — Supplementary Data 14 [file 41467_2020_17368_MOESM17_ESM.gz › BrainMaps/most_dk_thick/BrainMap035_rs13421039.png]

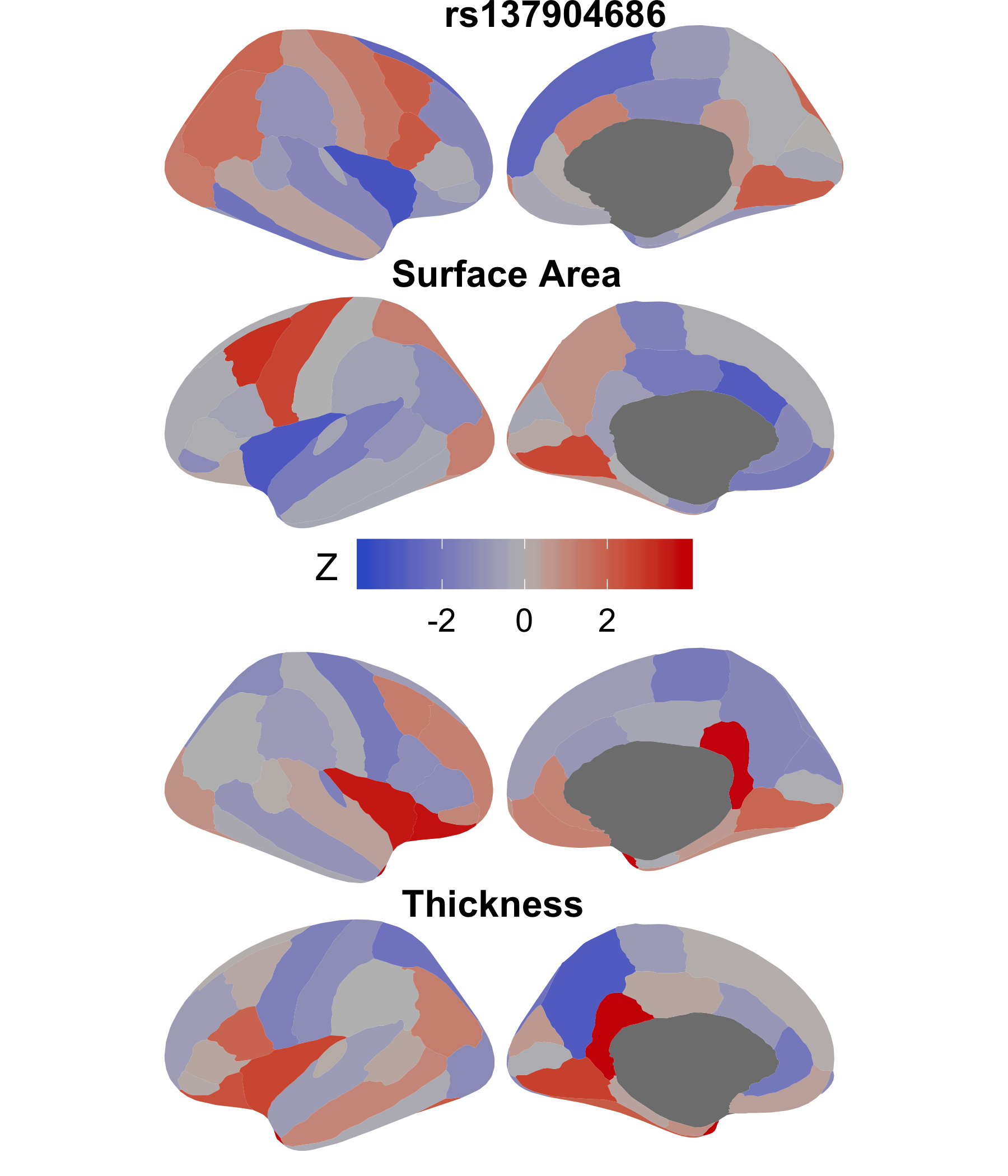

Supplement: Supplementary file 17 — Supplementary Data 14 [file 41467_2020_17368_MOESM17_ESM.gz › BrainMaps/most_dk_thick/BrainMap053_rs137904686.png]

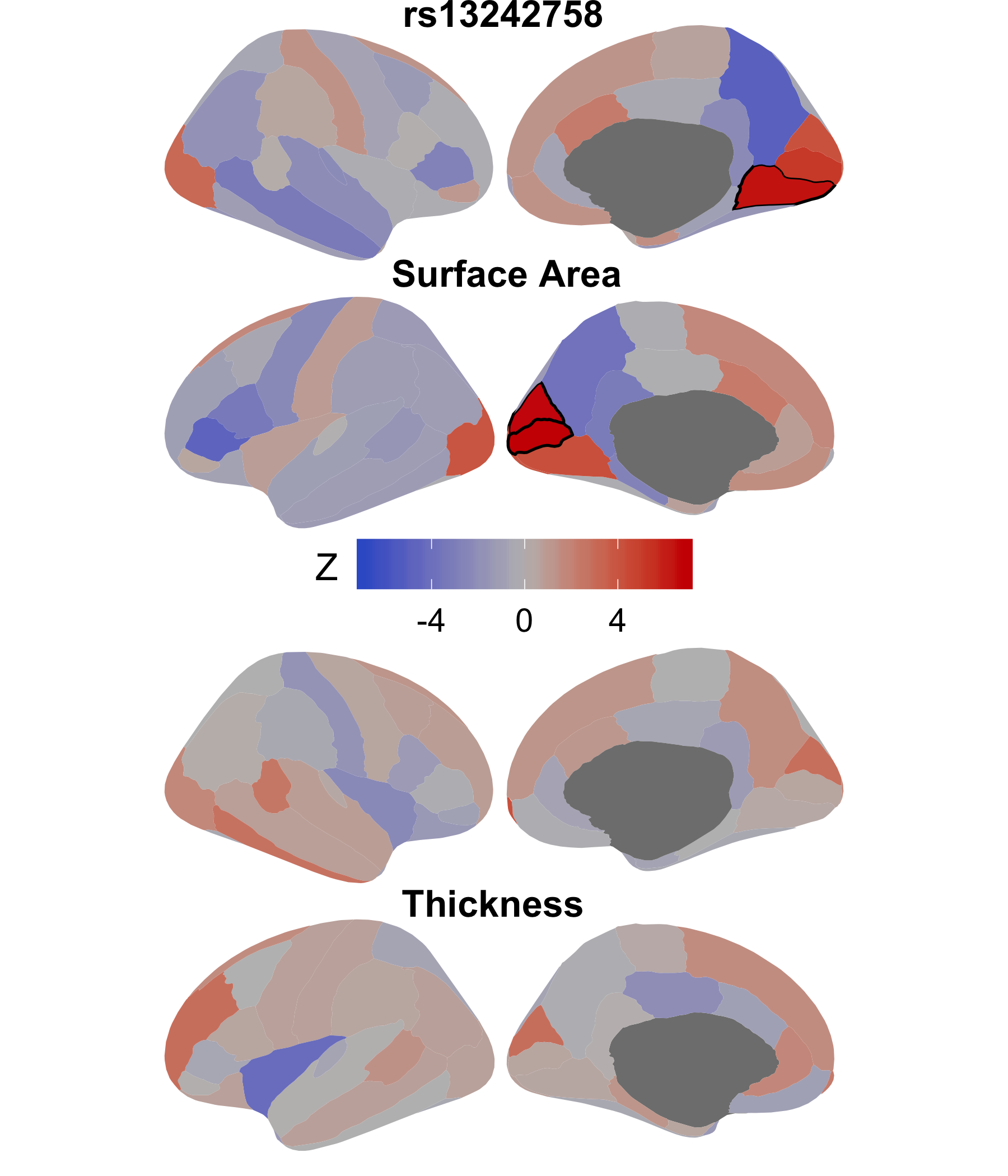

Supplement: Supplementary file 17 — Supplementary Data 14 [file 41467_2020_17368_MOESM17_ESM.gz › BrainMaps/most_dk_thick/BrainMap055_rs13242758.png]

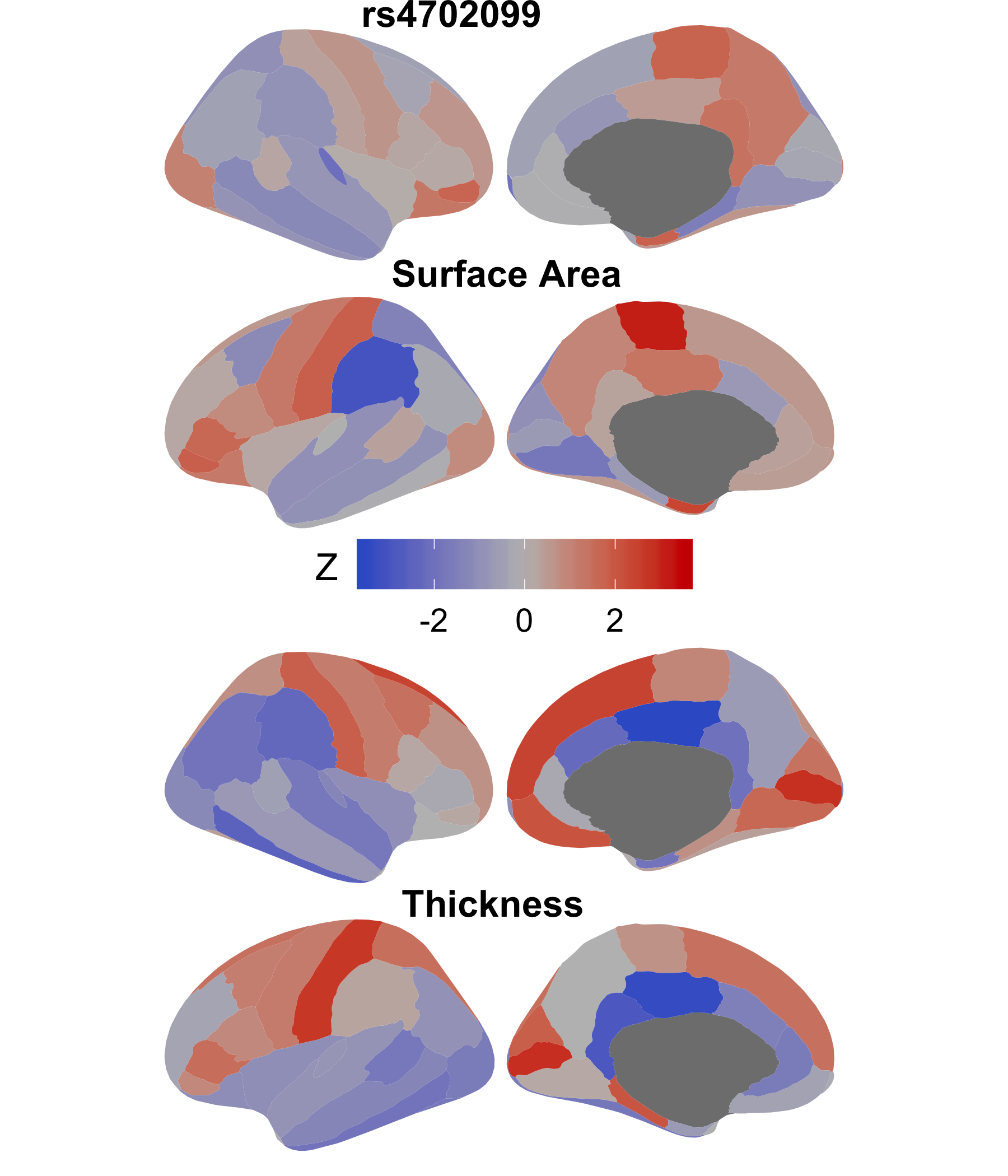

Supplement: Supplementary file 17 — Supplementary Data 14 [file 41467_2020_17368_MOESM17_ESM.gz › BrainMaps/most_aseg_vol/BrainMap084_rs4702099.png]

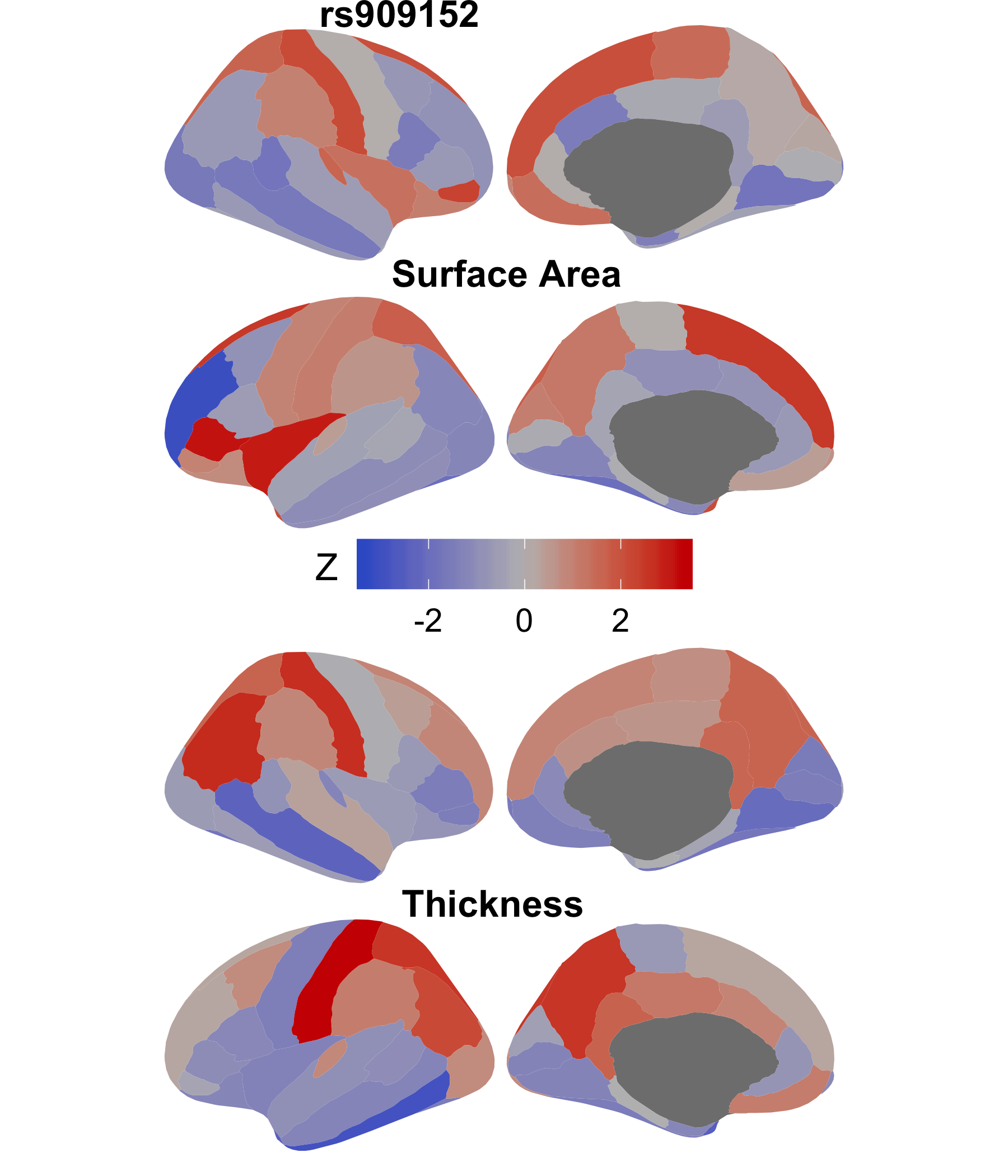

Supplement: Supplementary file 17 — Supplementary Data 14 [file 41467_2020_17368_MOESM17_ESM.gz › BrainMaps/most_aseg_vol/BrainMap078_rs909152.png]

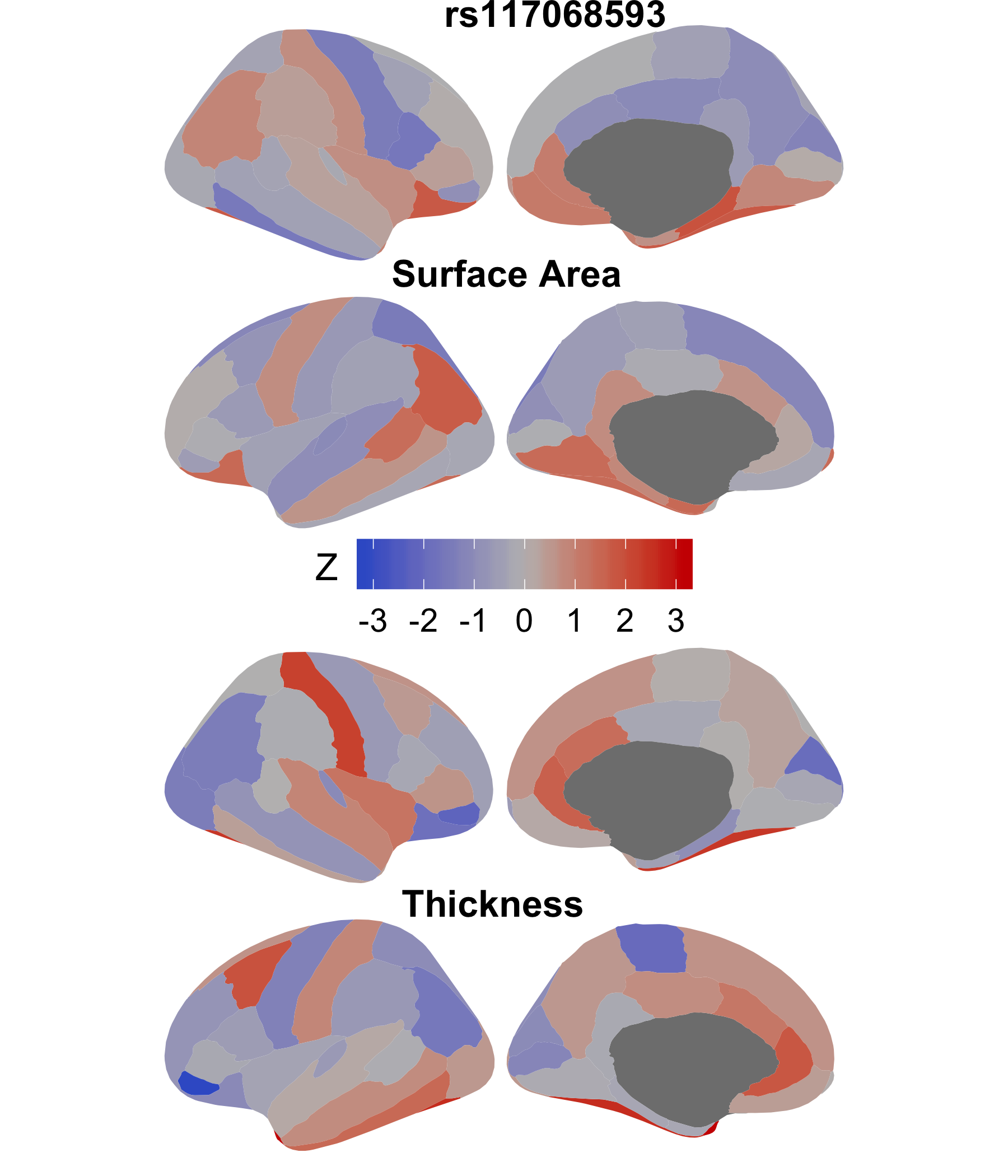

Supplement: Supplementary file 17 — Supplementary Data 14 [file 41467_2020_17368_MOESM17_ESM.gz › BrainMaps/most_aseg_vol/BrainMap119_rs117068593.png]

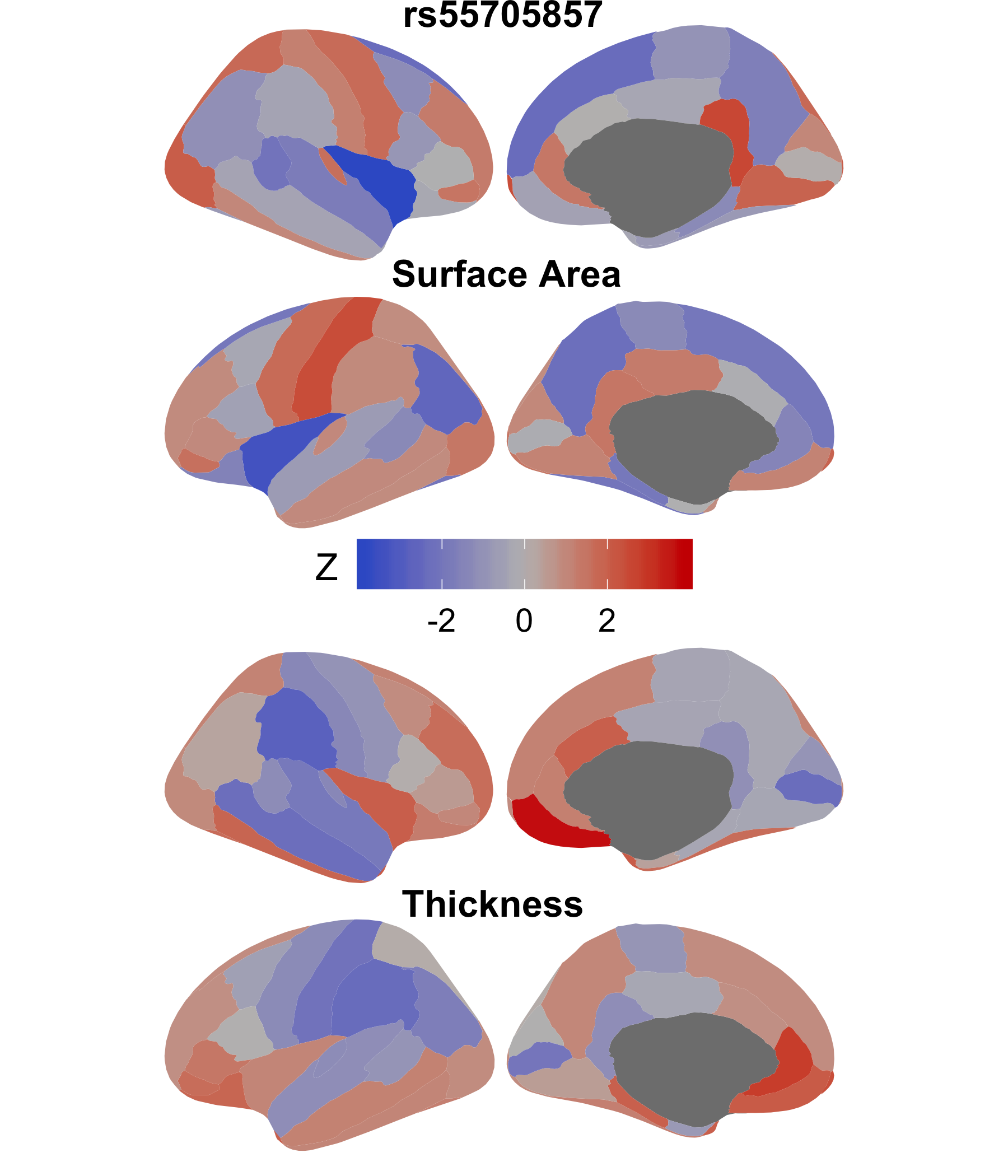

Supplement: Supplementary file 17 — Supplementary Data 14 [file 41467_2020_17368_MOESM17_ESM.gz › BrainMaps/most_aseg_vol/BrainMap068_rs55705857.png]

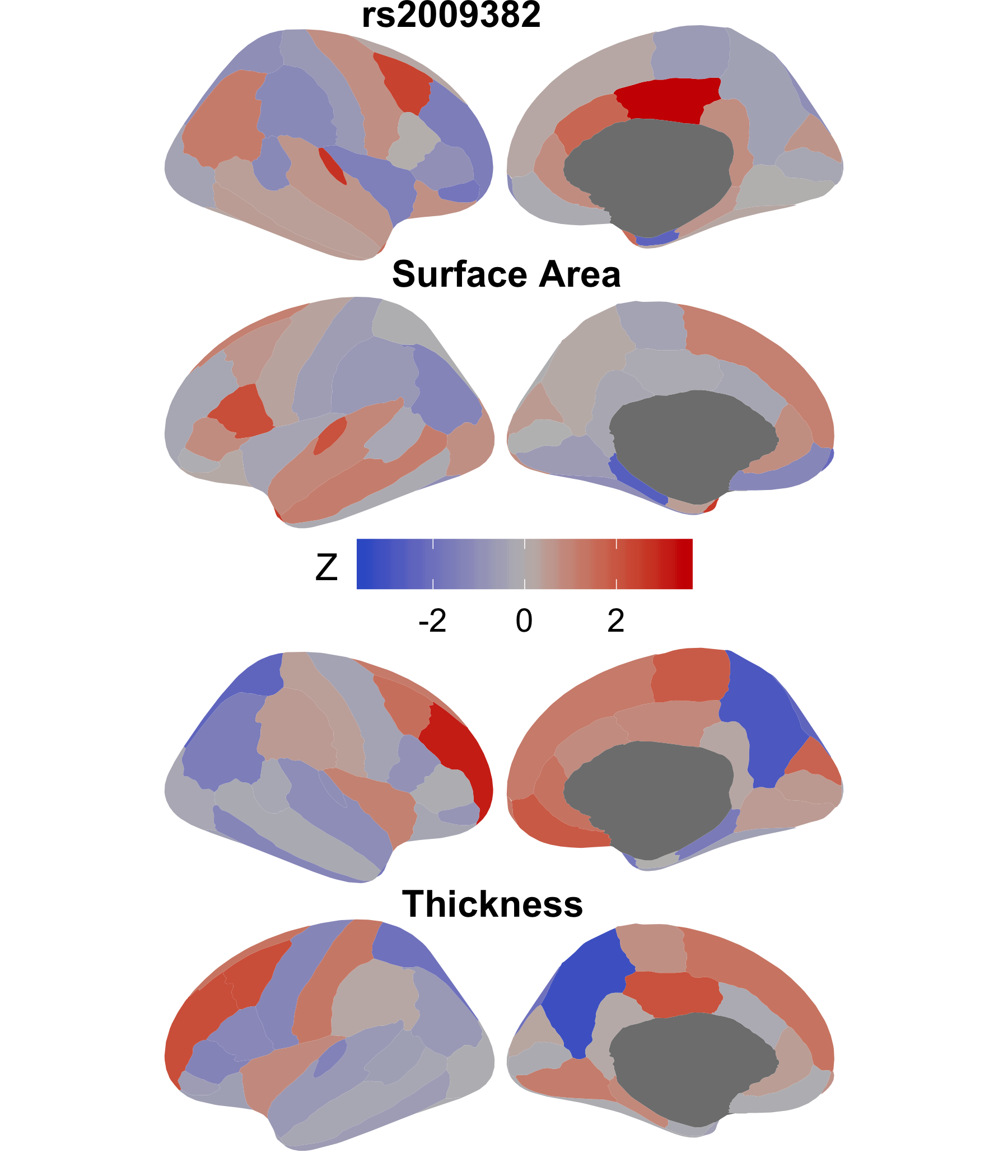

Supplement: Supplementary file 17 — Supplementary Data 14 [file 41467_2020_17368_MOESM17_ESM.gz › BrainMaps/most_aseg_vol/BrainMap127_rs2009382.png]

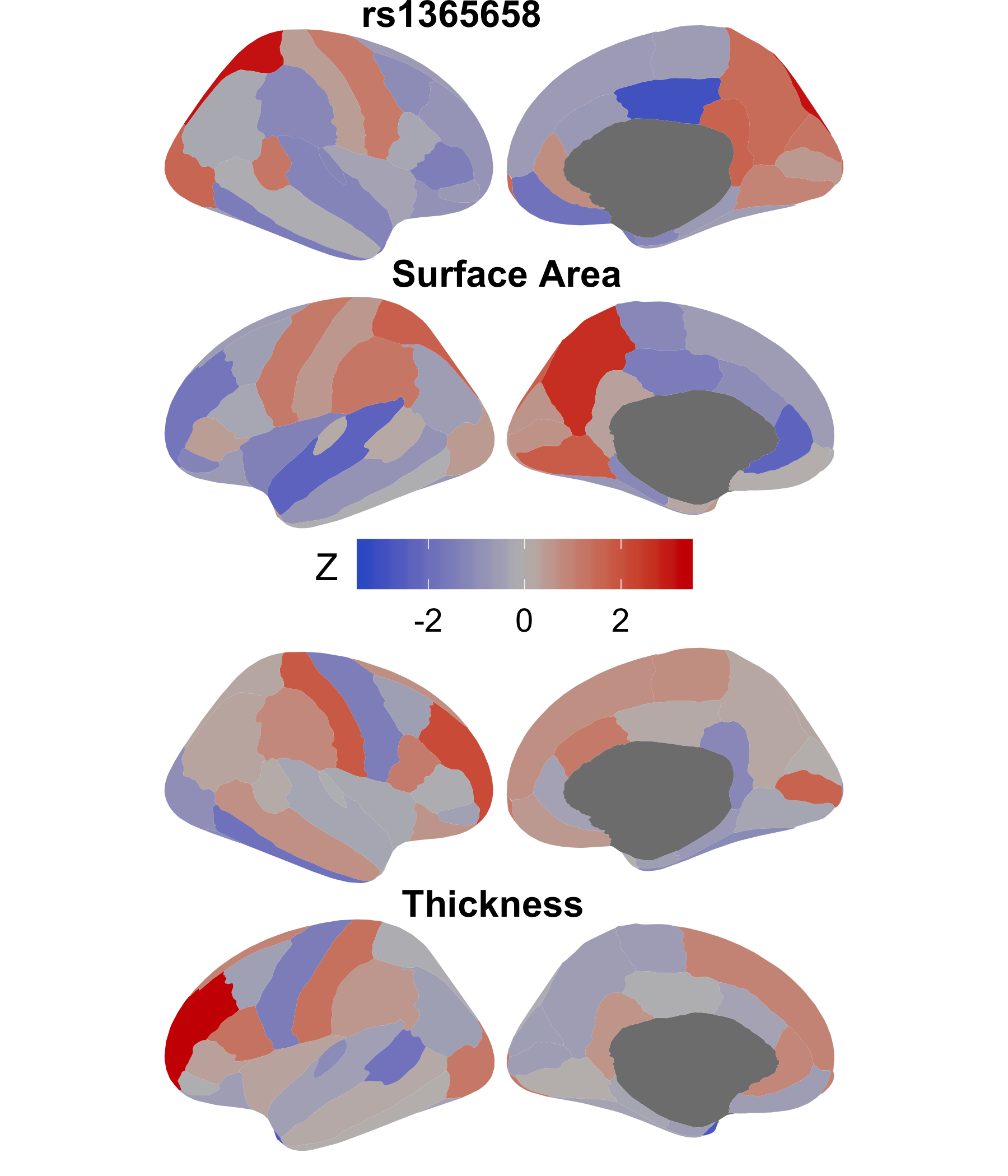

Supplement: Supplementary file 17 — Supplementary Data 14 [file 41467_2020_17368_MOESM17_ESM.gz › BrainMaps/most_aseg_vol/BrainMap043_rs1365658.png]

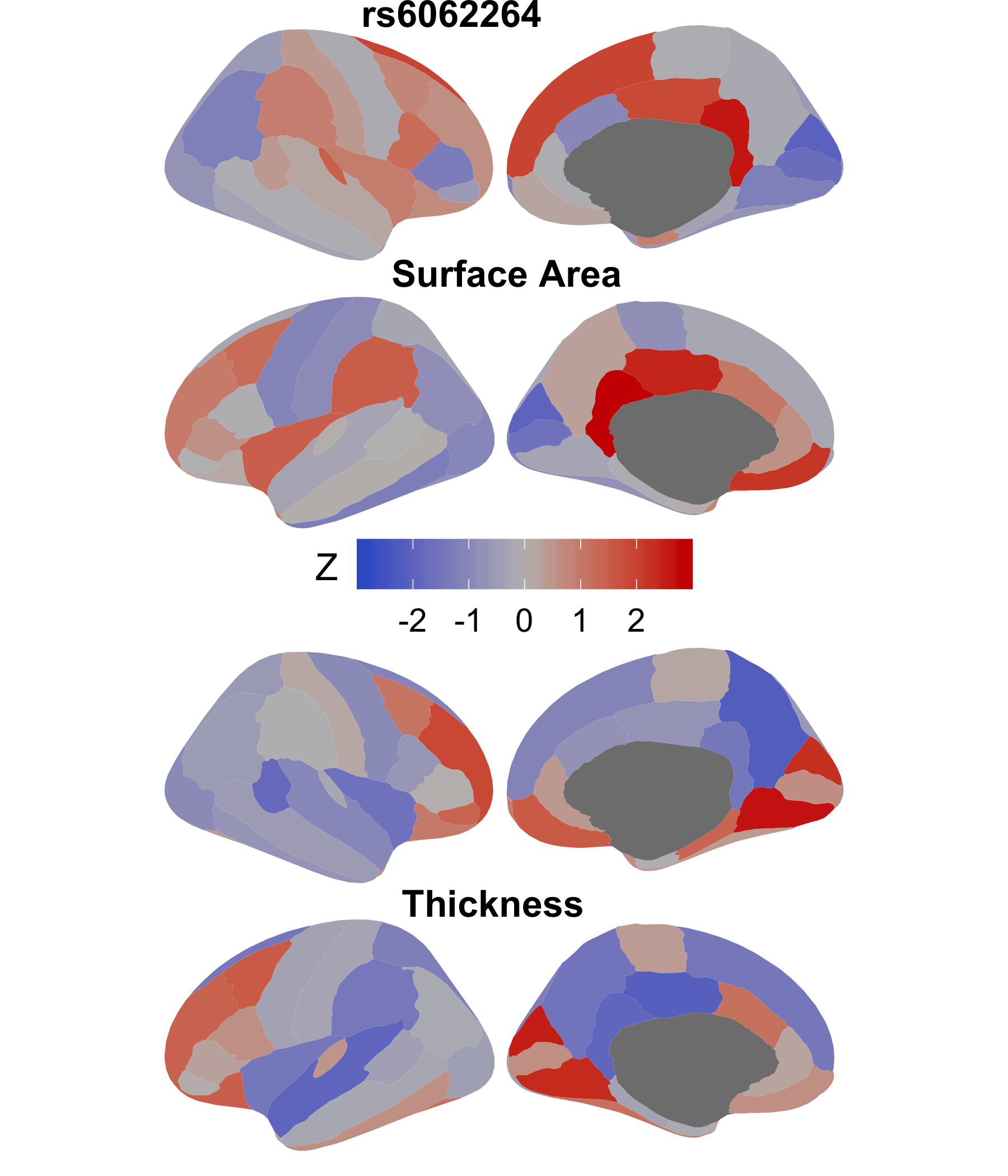

Supplement: Supplementary file 17 — Supplementary Data 14 [file 41467_2020_17368_MOESM17_ESM.gz › BrainMaps/most_aseg_vol/BrainMap044_rs6062264.png]

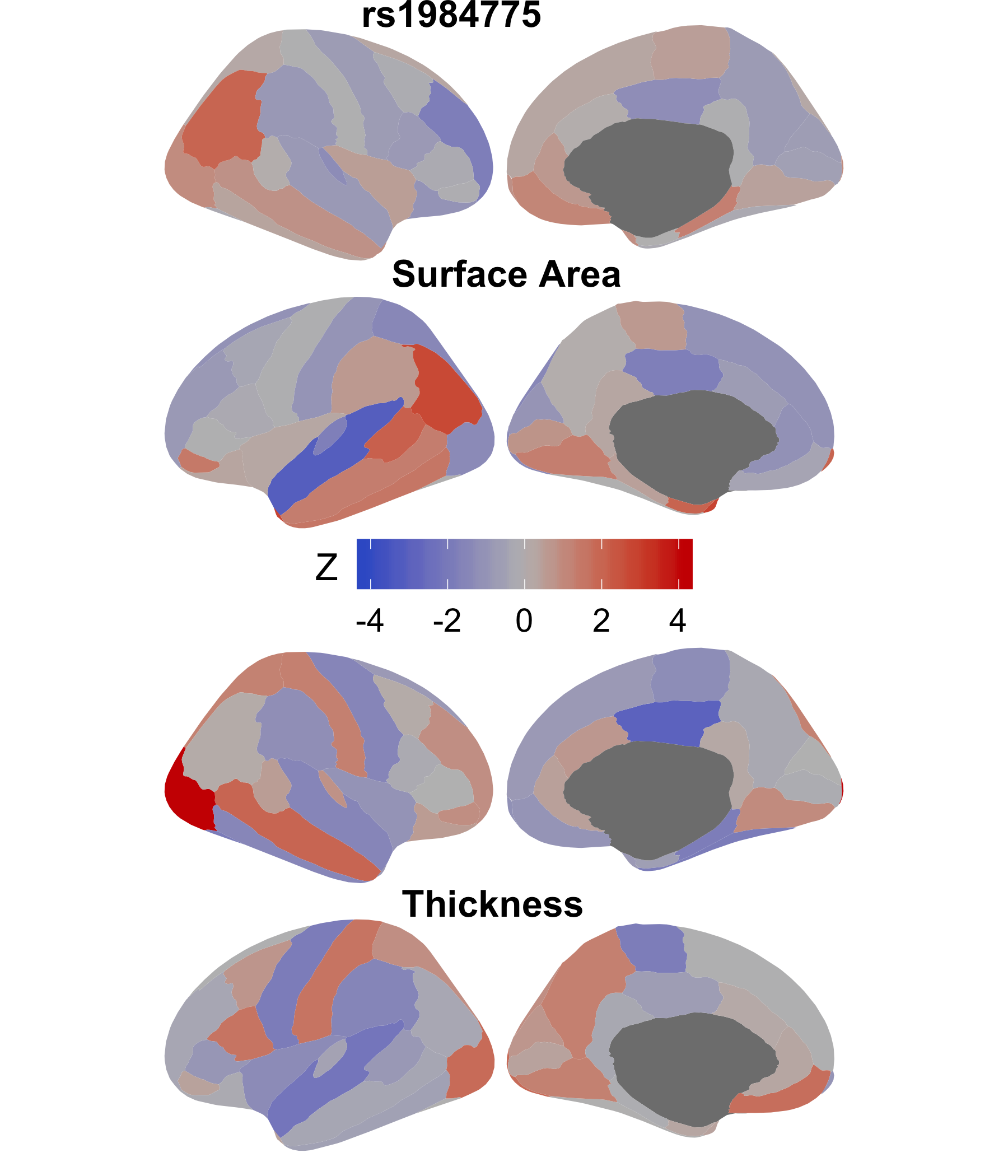

Supplement: Supplementary file 17 — Supplementary Data 14 [file 41467_2020_17368_MOESM17_ESM.gz › BrainMaps/most_aseg_vol/BrainMap120_rs1984775.png]

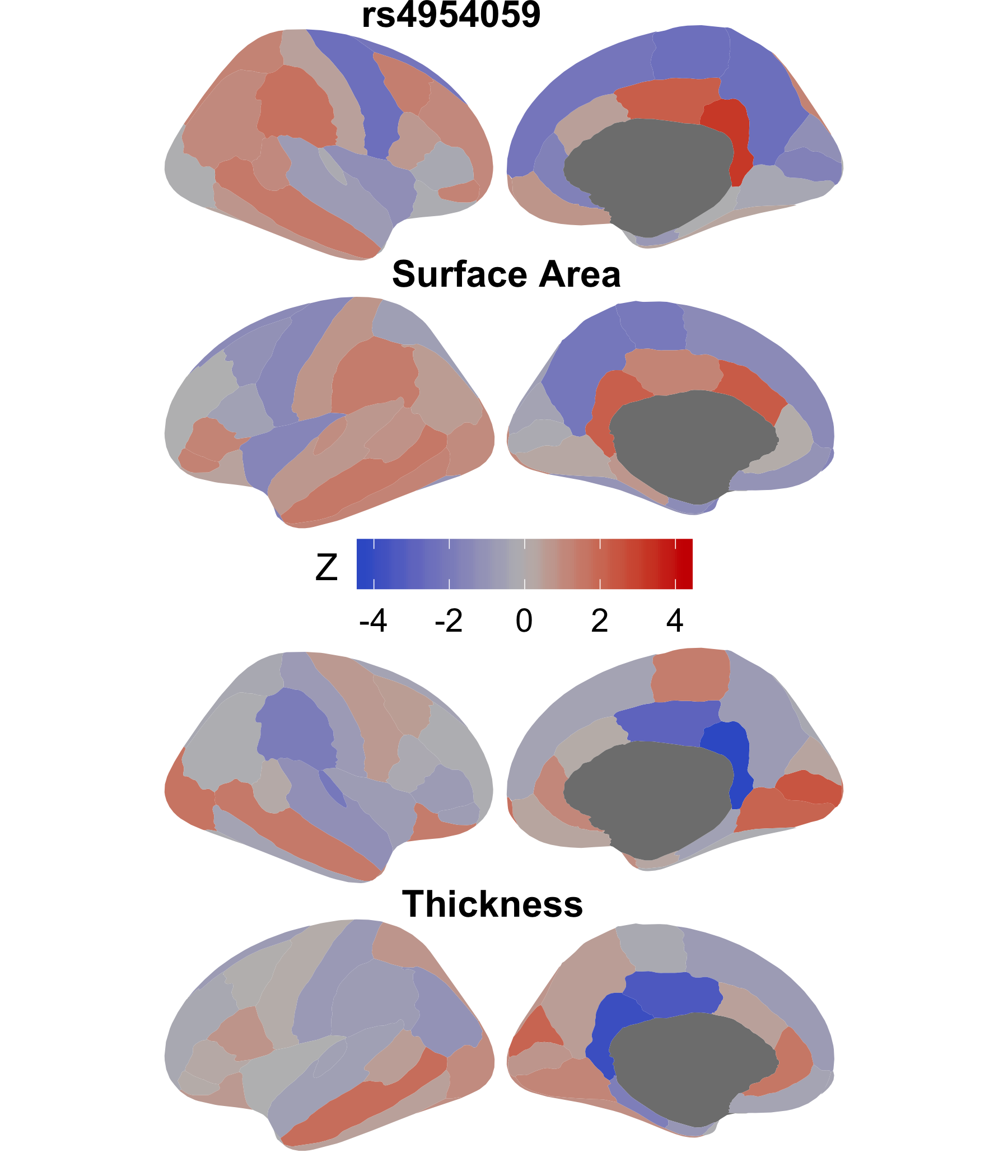

Supplement: Supplementary file 17 — Supplementary Data 14 [file 41467_2020_17368_MOESM17_ESM.gz › BrainMaps/most_aseg_vol/BrainMap100_rs4954059.png]

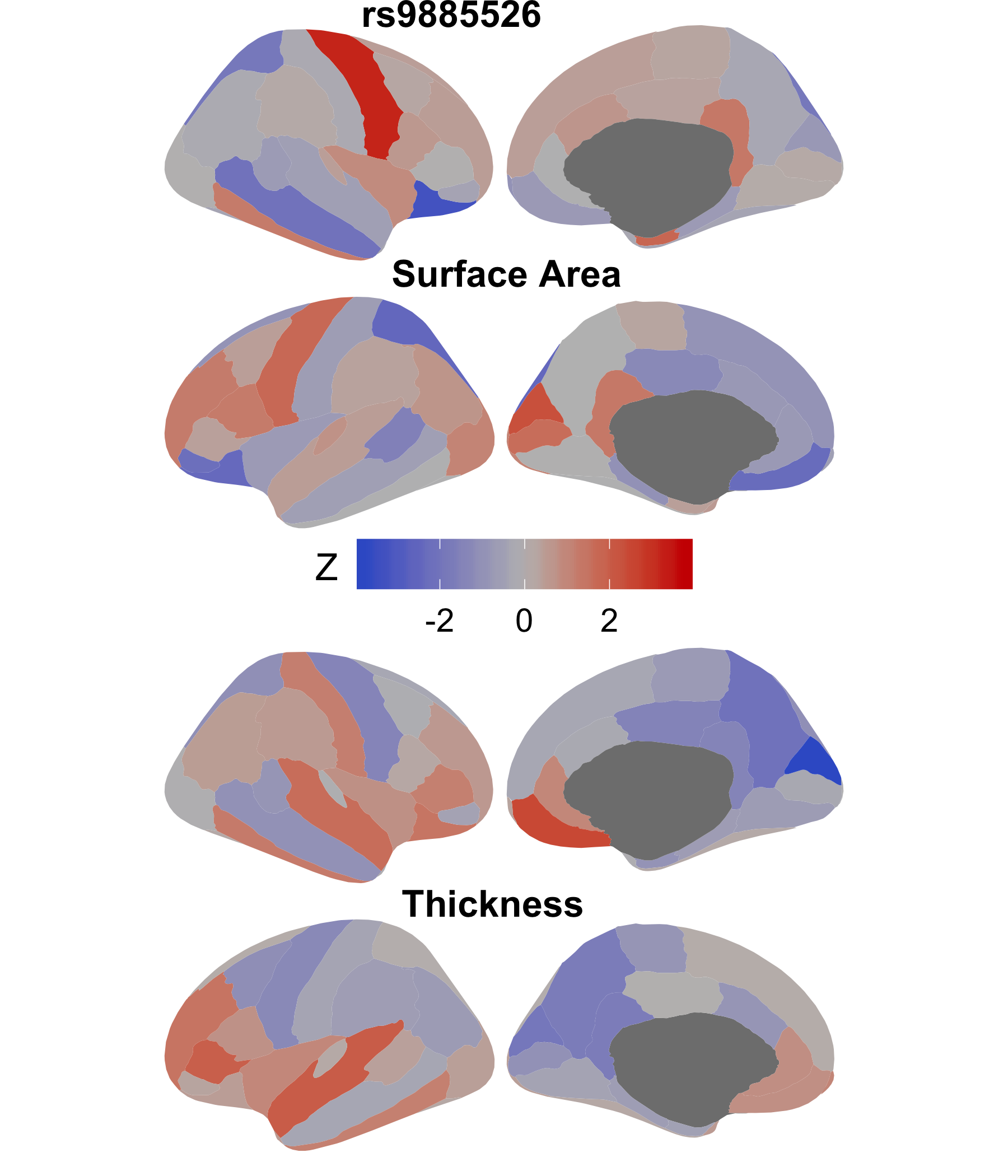

Supplement: Supplementary file 17 — Supplementary Data 14 [file 41467_2020_17368_MOESM17_ESM.gz › BrainMaps/most_aseg_vol/BrainMap141_rs9885526.png]

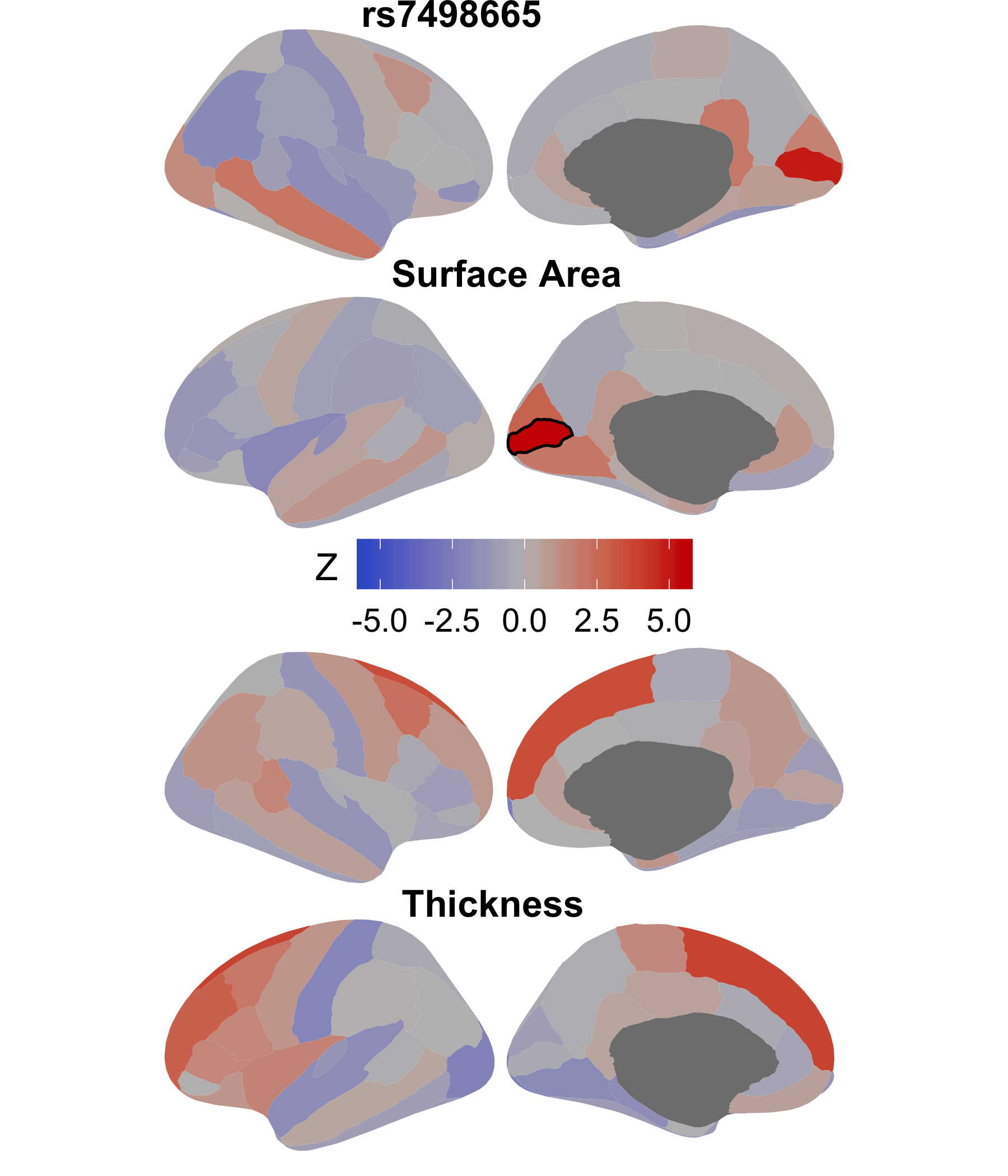

Supplement: Supplementary file 17 — Supplementary Data 14 [file 41467_2020_17368_MOESM17_ESM.gz › BrainMaps/most_aseg_vol/BrainMap049_rs7498665.png]

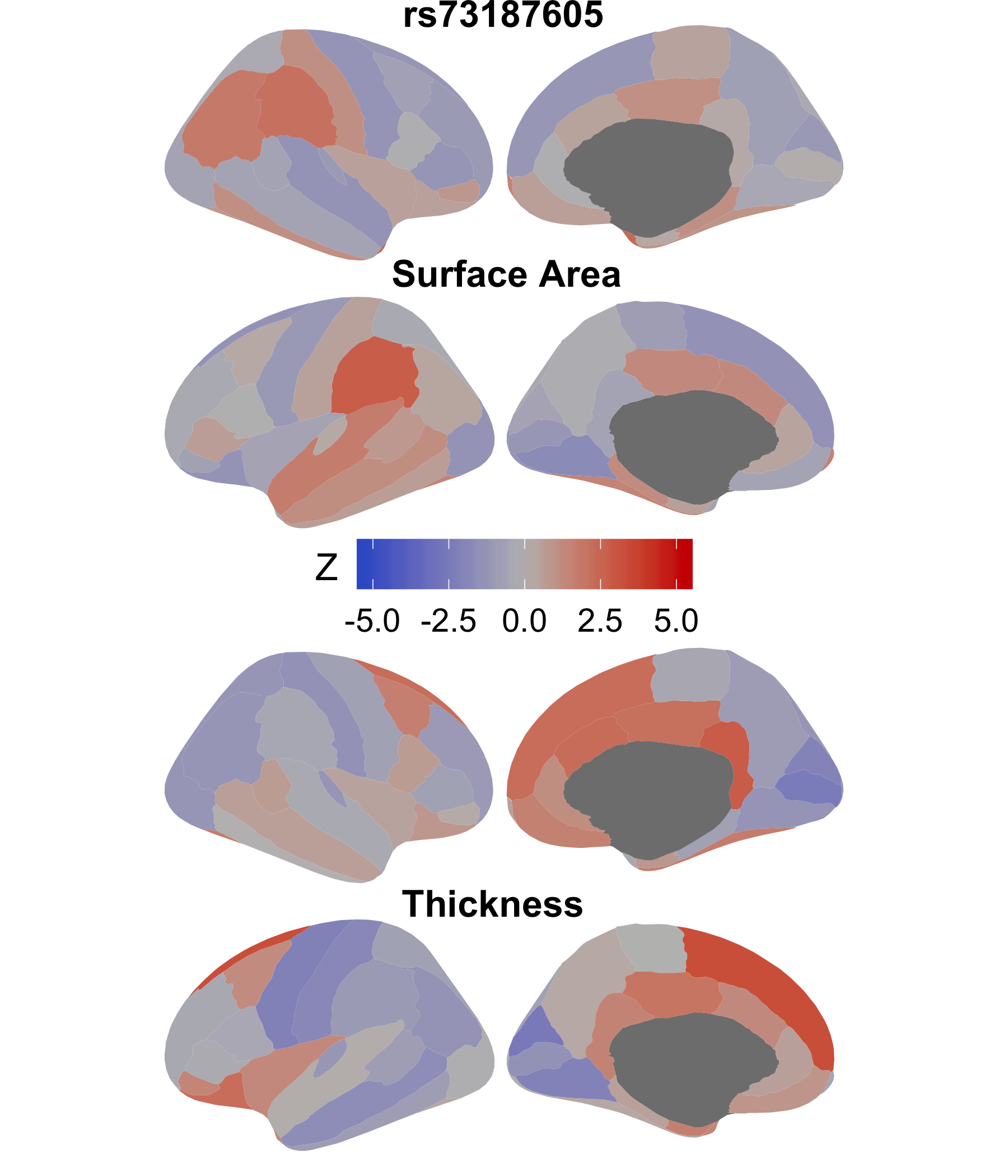

Supplement: Supplementary file 17 — Supplementary Data 14 [file 41467_2020_17368_MOESM17_ESM.gz › BrainMaps/most_aseg_vol/BrainMap172_rs73187605.png]

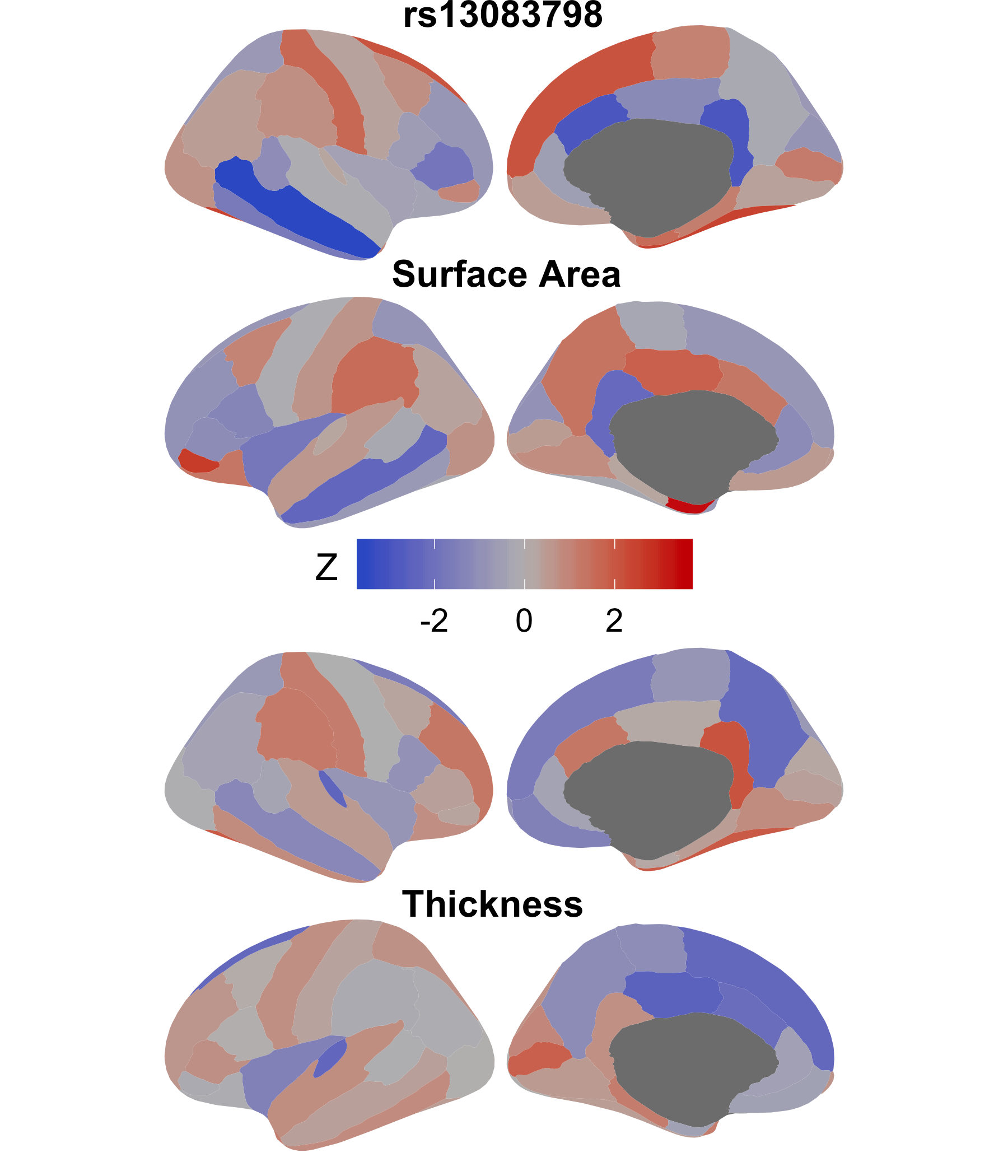

Supplement: Supplementary file 17 — Supplementary Data 14 [file 41467_2020_17368_MOESM17_ESM.gz › BrainMaps/most_aseg_vol/BrainMap157_rs13083798.png]

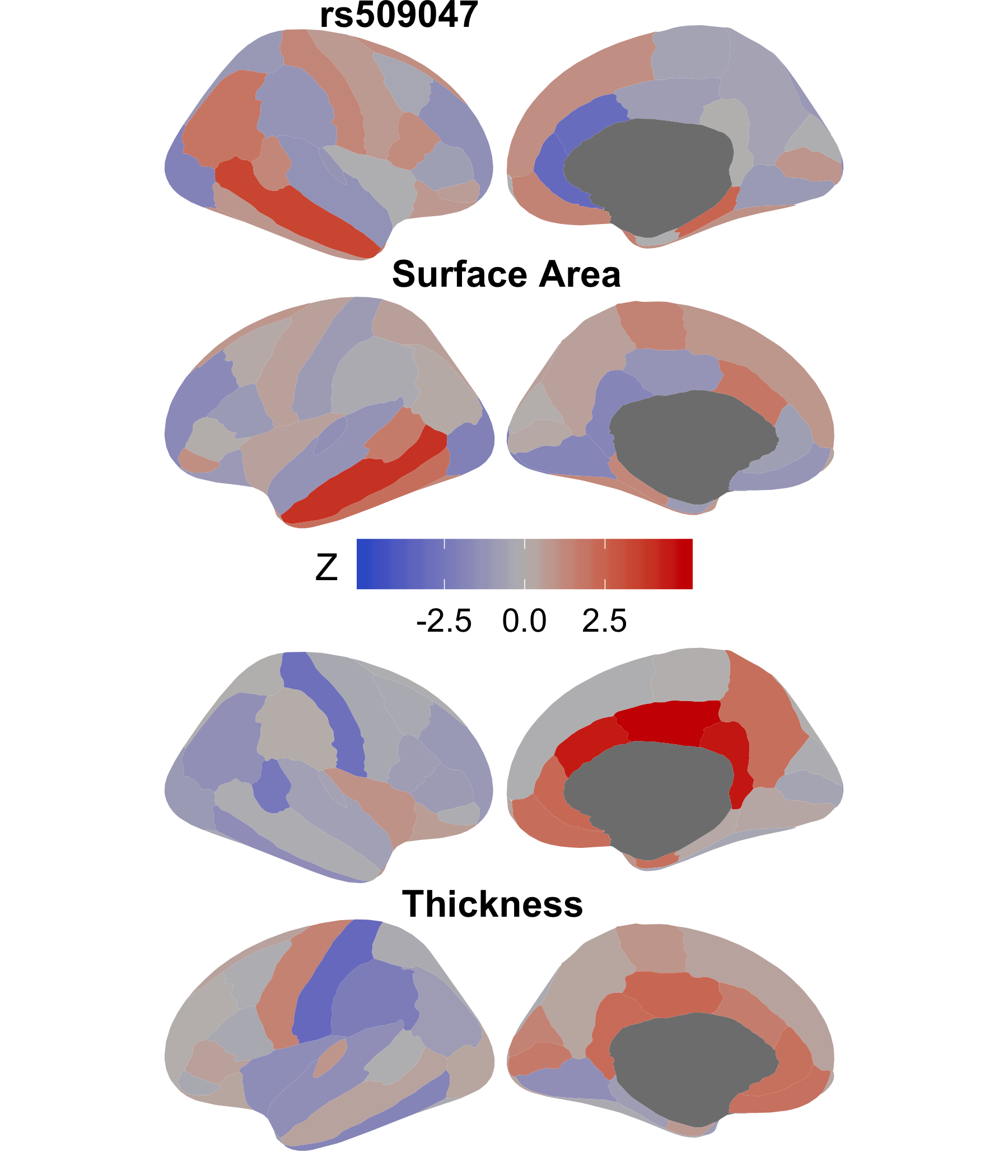

Supplement: Supplementary file 17 — Supplementary Data 14 [file 41467_2020_17368_MOESM17_ESM.gz › BrainMaps/most_aseg_vol/BrainMap138_rs509047.png]

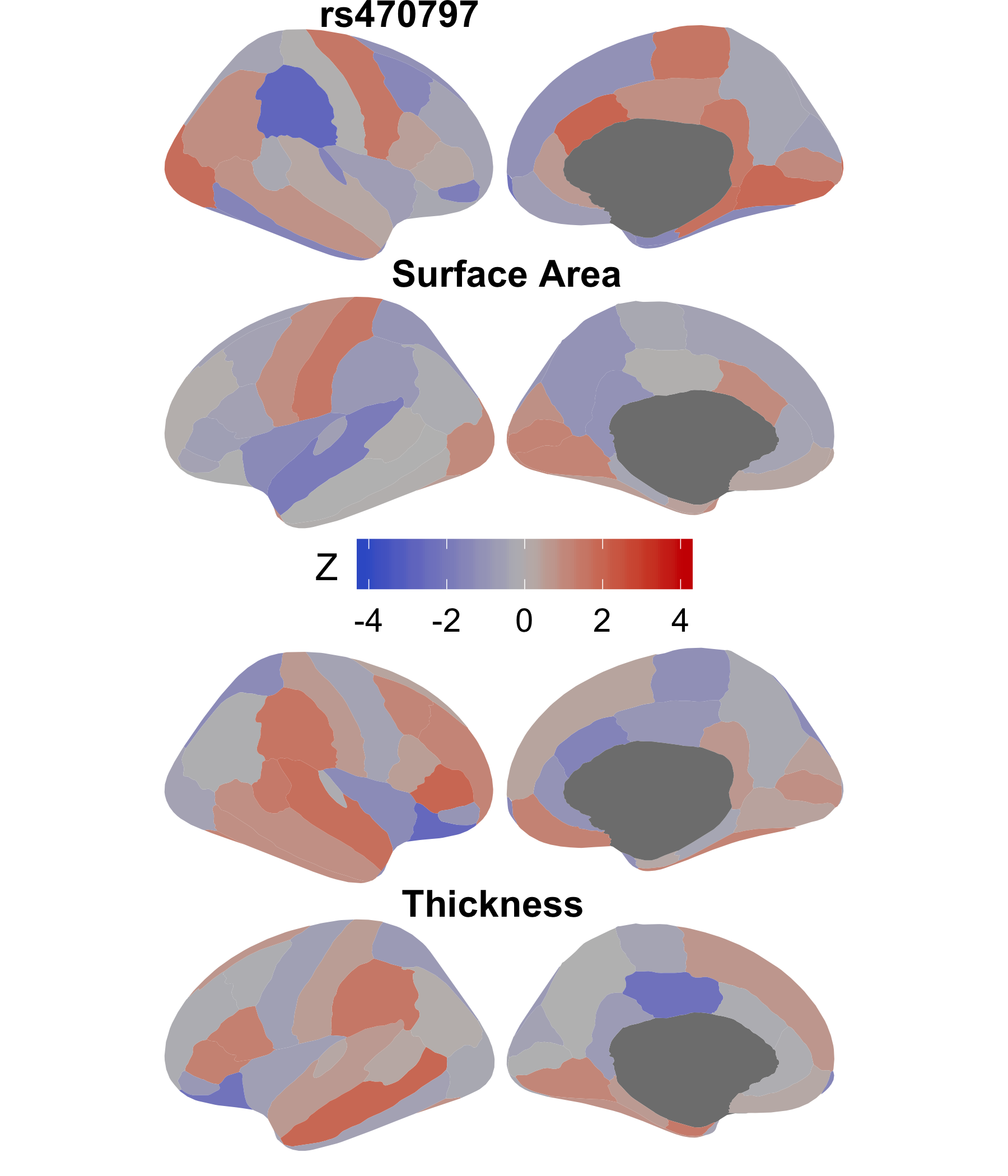

Supplement: Supplementary file 17 — Supplementary Data 14 [file 41467_2020_17368_MOESM17_ESM.gz › BrainMaps/most_aseg_vol/BrainMap108_rs470797.png]

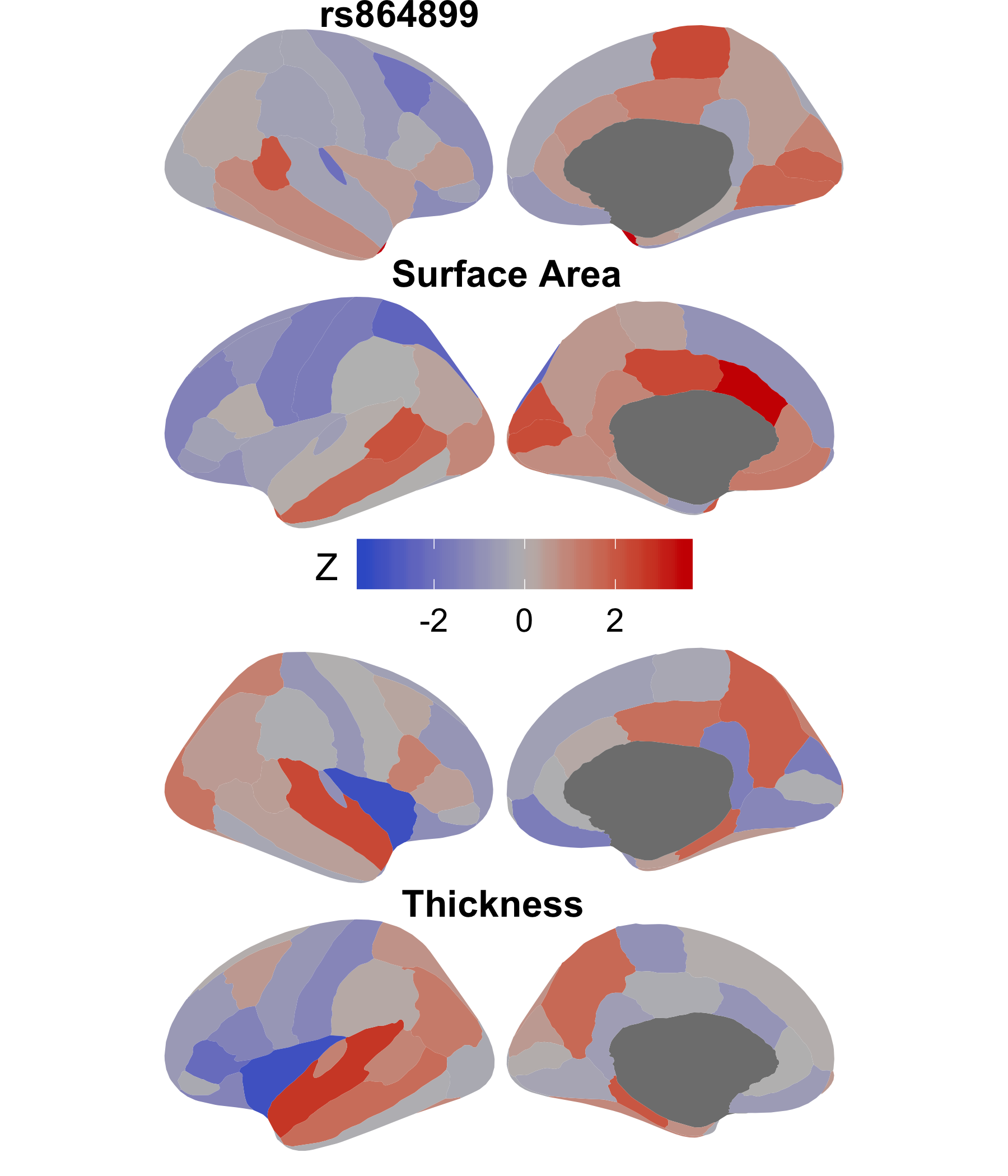

Supplement: Supplementary file 17 — Supplementary Data 14 [file 41467_2020_17368_MOESM17_ESM.gz › BrainMaps/most_aseg_vol/BrainMap110_rs864899.png]

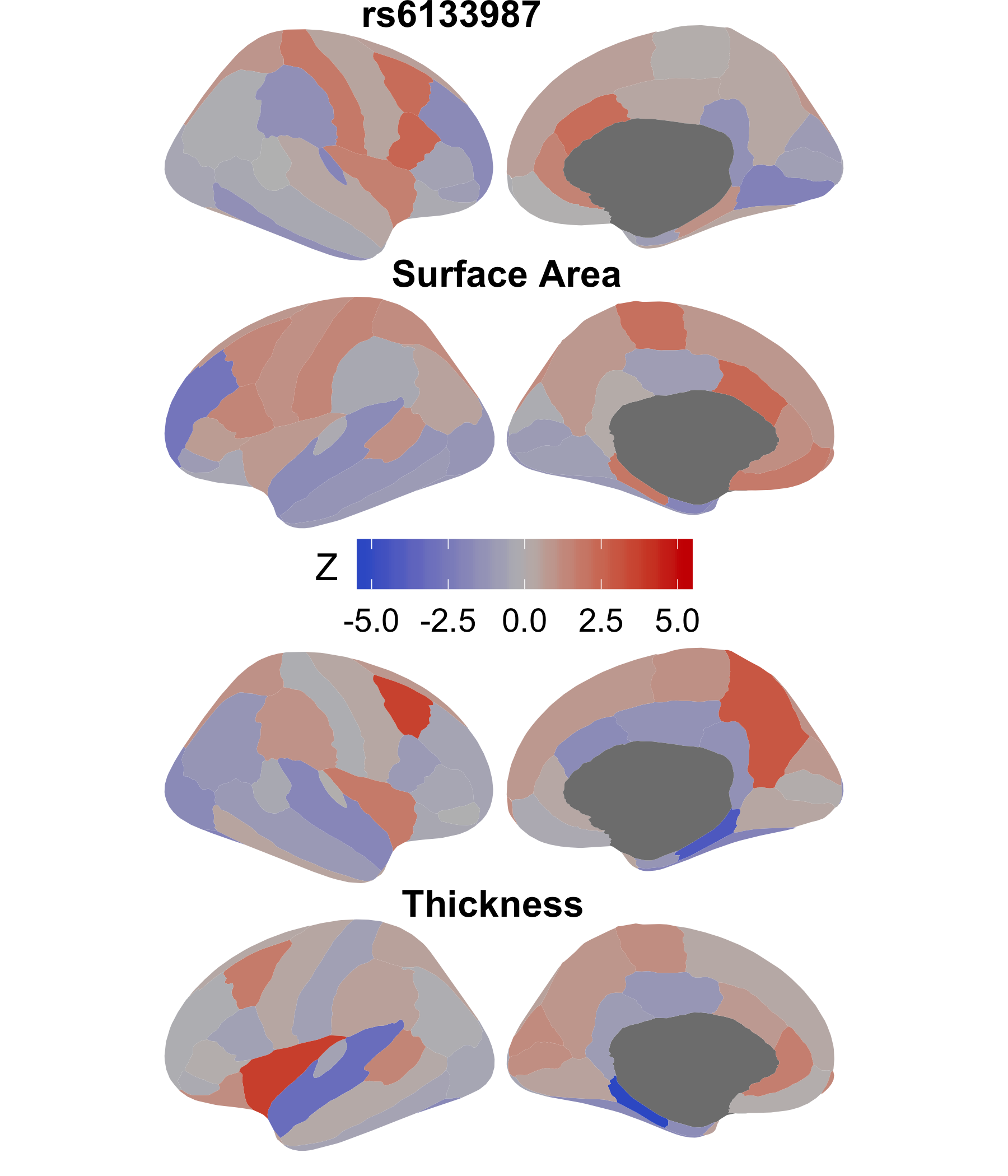

Supplement: Supplementary file 17 — Supplementary Data 14 [file 41467_2020_17368_MOESM17_ESM.gz › BrainMaps/most_aseg_vol/BrainMap024_rs6133987.png]

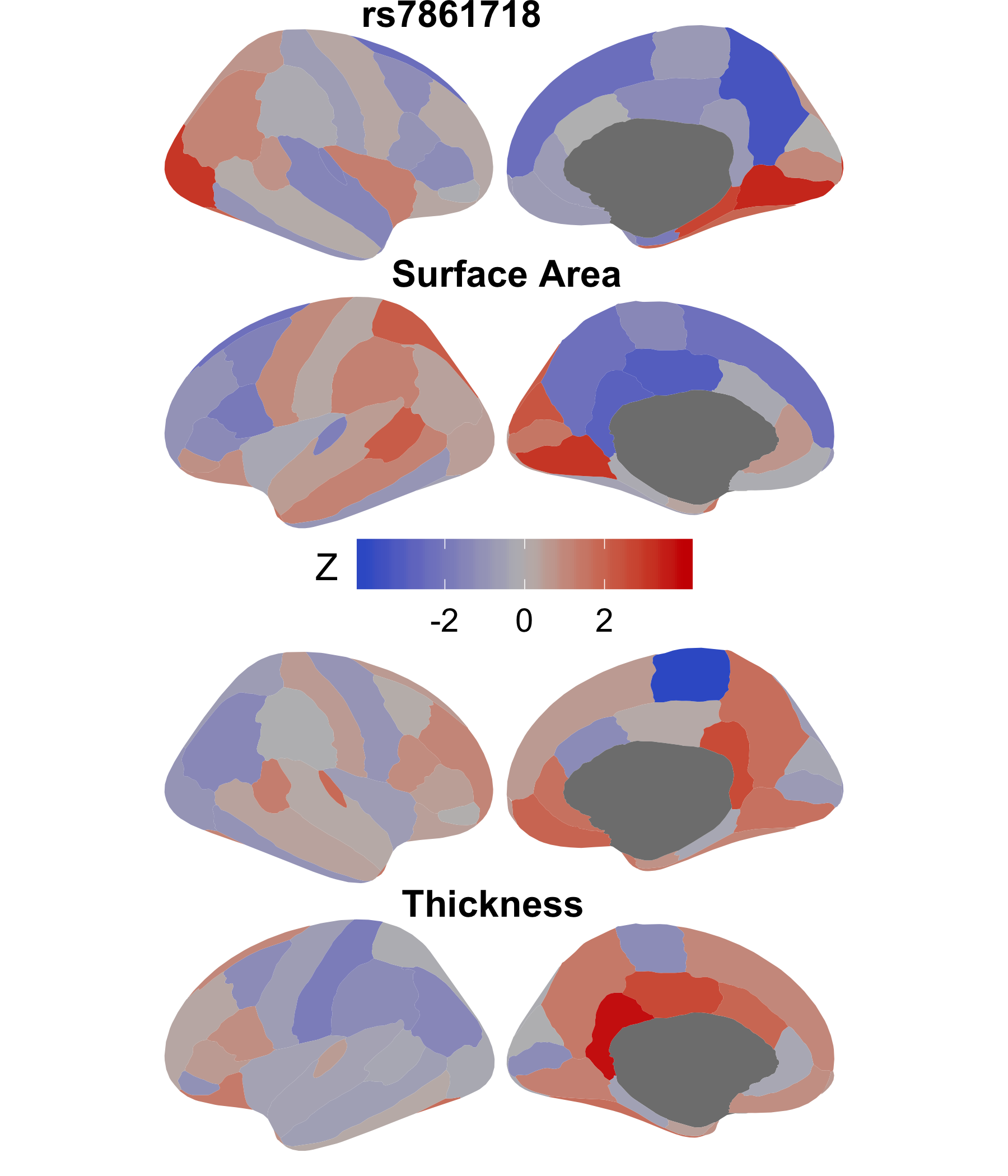

Supplement: Supplementary file 17 — Supplementary Data 14 [file 41467_2020_17368_MOESM17_ESM.gz › BrainMaps/most_aseg_vol/BrainMap081_rs7861718.png]

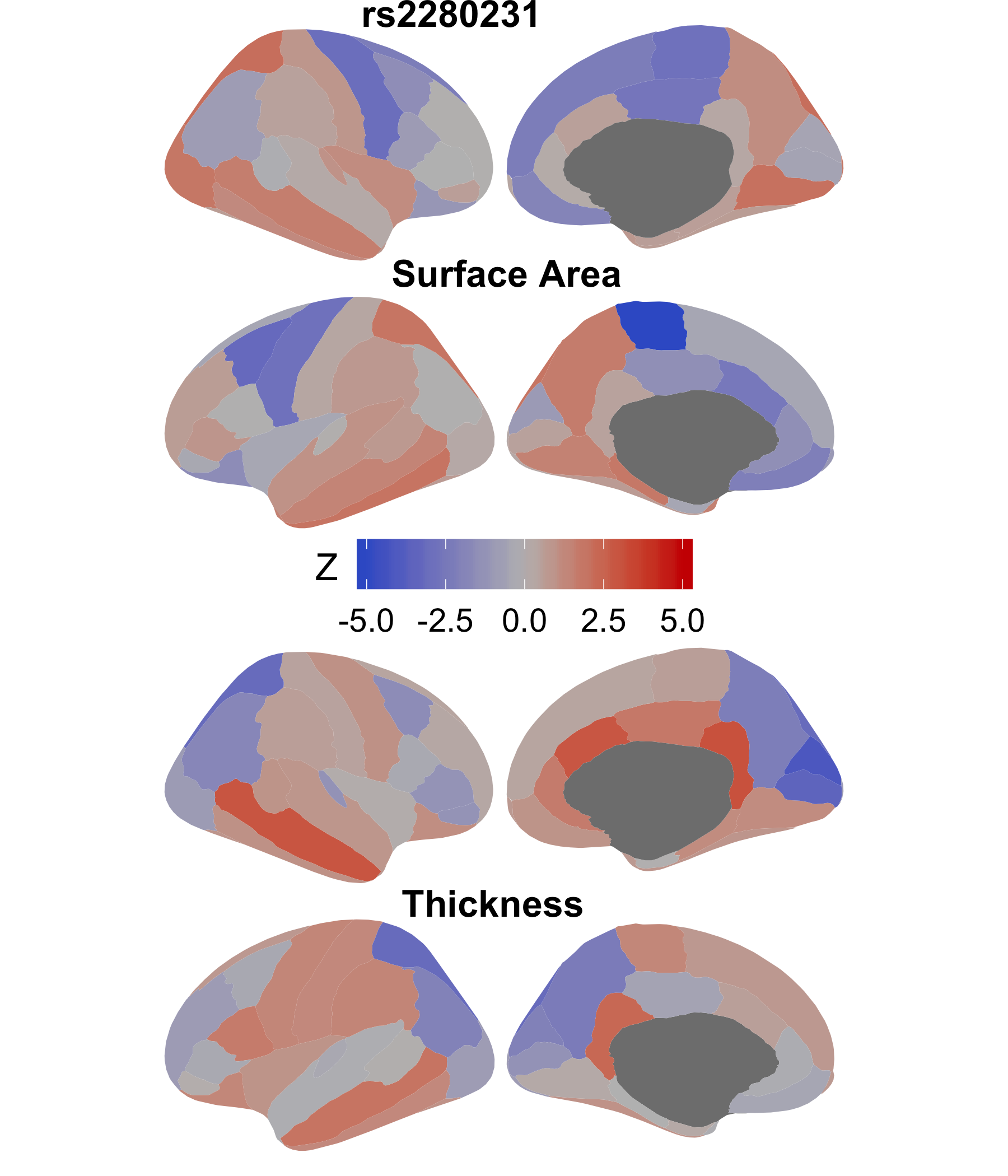

Supplement: Supplementary file 17 — Supplementary Data 14 [file 41467_2020_17368_MOESM17_ESM.gz › BrainMaps/most_aseg_vol/BrainMap069_rs2280231.png]

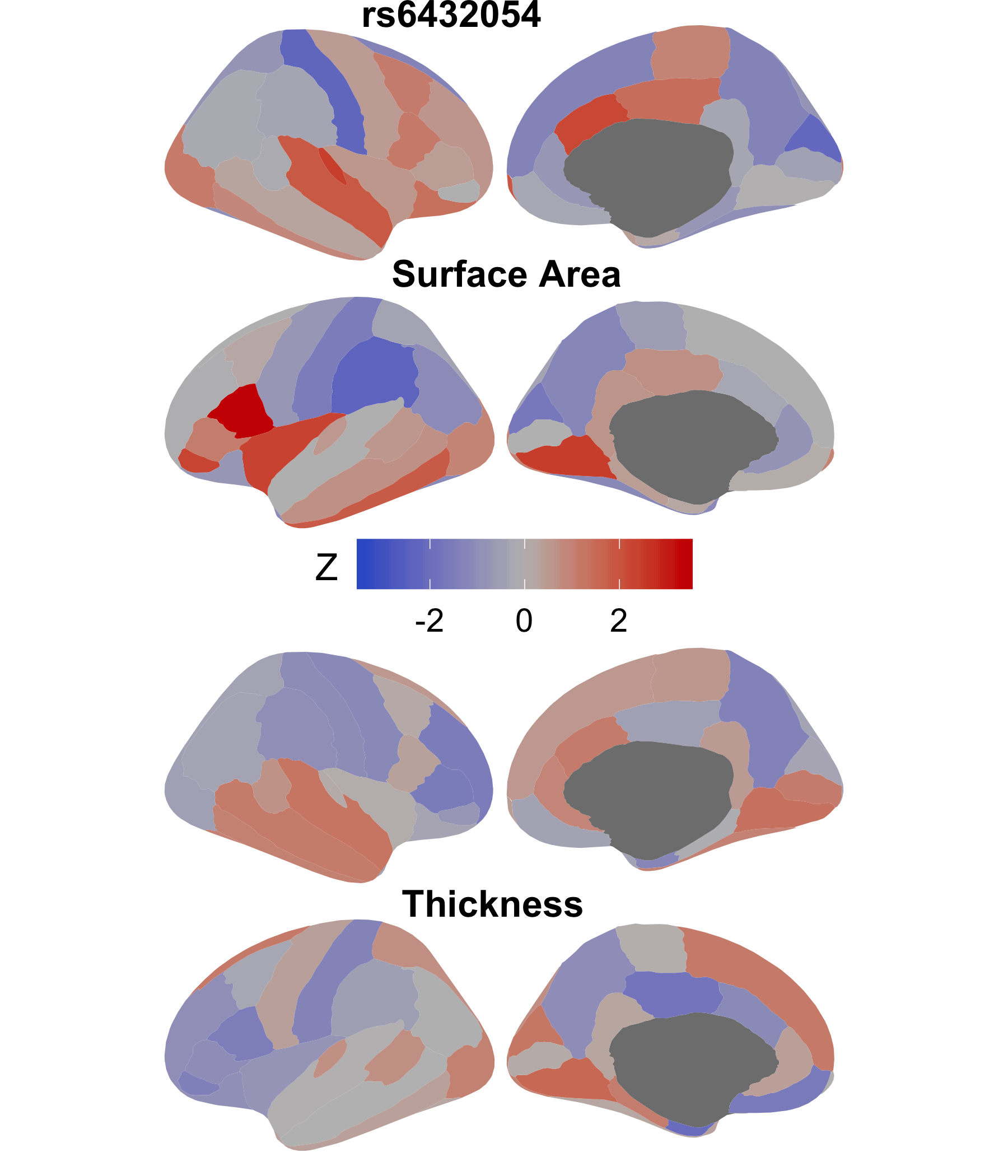

Supplement: Supplementary file 17 — Supplementary Data 14 [file 41467_2020_17368_MOESM17_ESM.gz › BrainMaps/most_aseg_vol/BrainMap166_rs6432054.png]

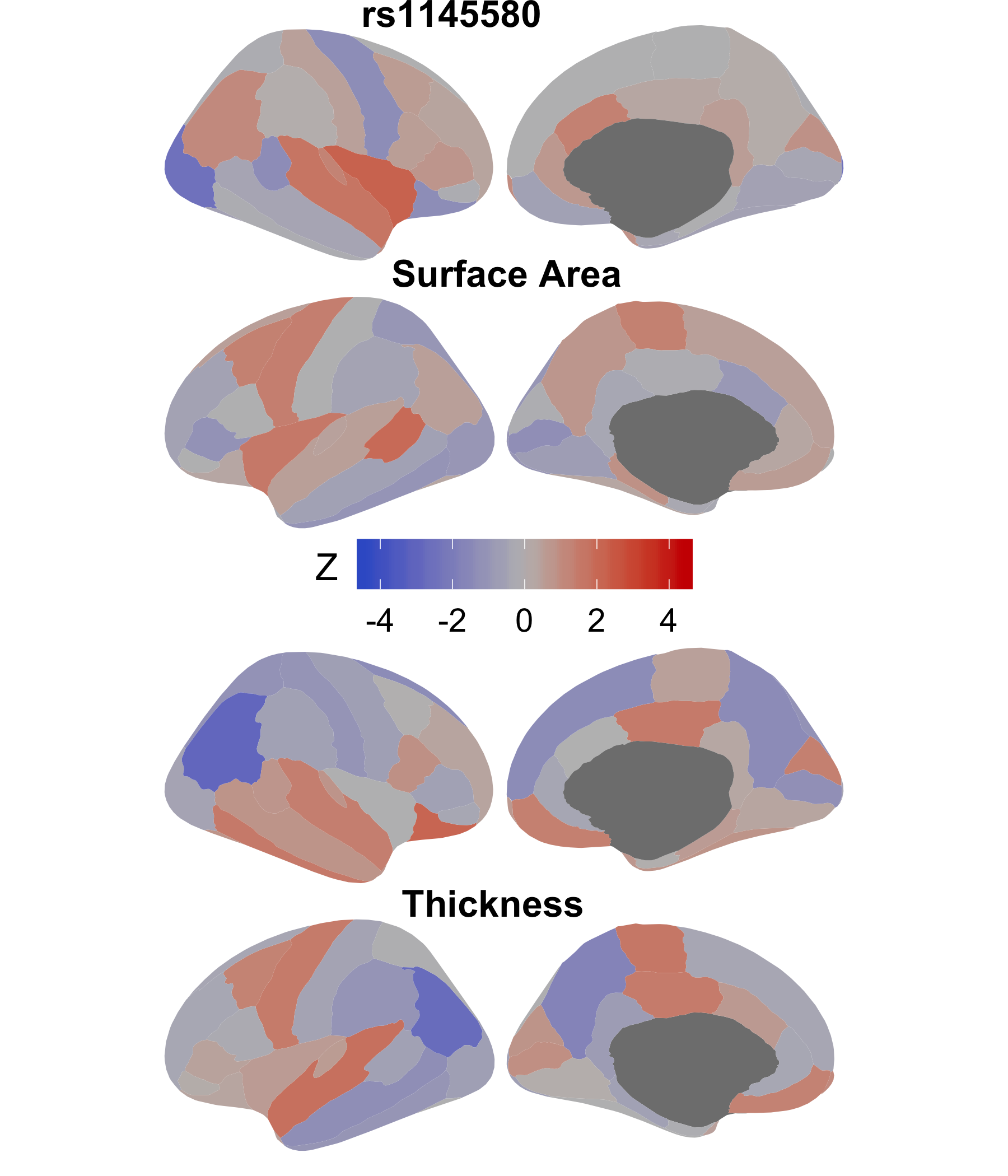

Supplement: Supplementary file 17 — Supplementary Data 14 [file 41467_2020_17368_MOESM17_ESM.gz › BrainMaps/most_aseg_vol/BrainMap150_rs1145580.png]

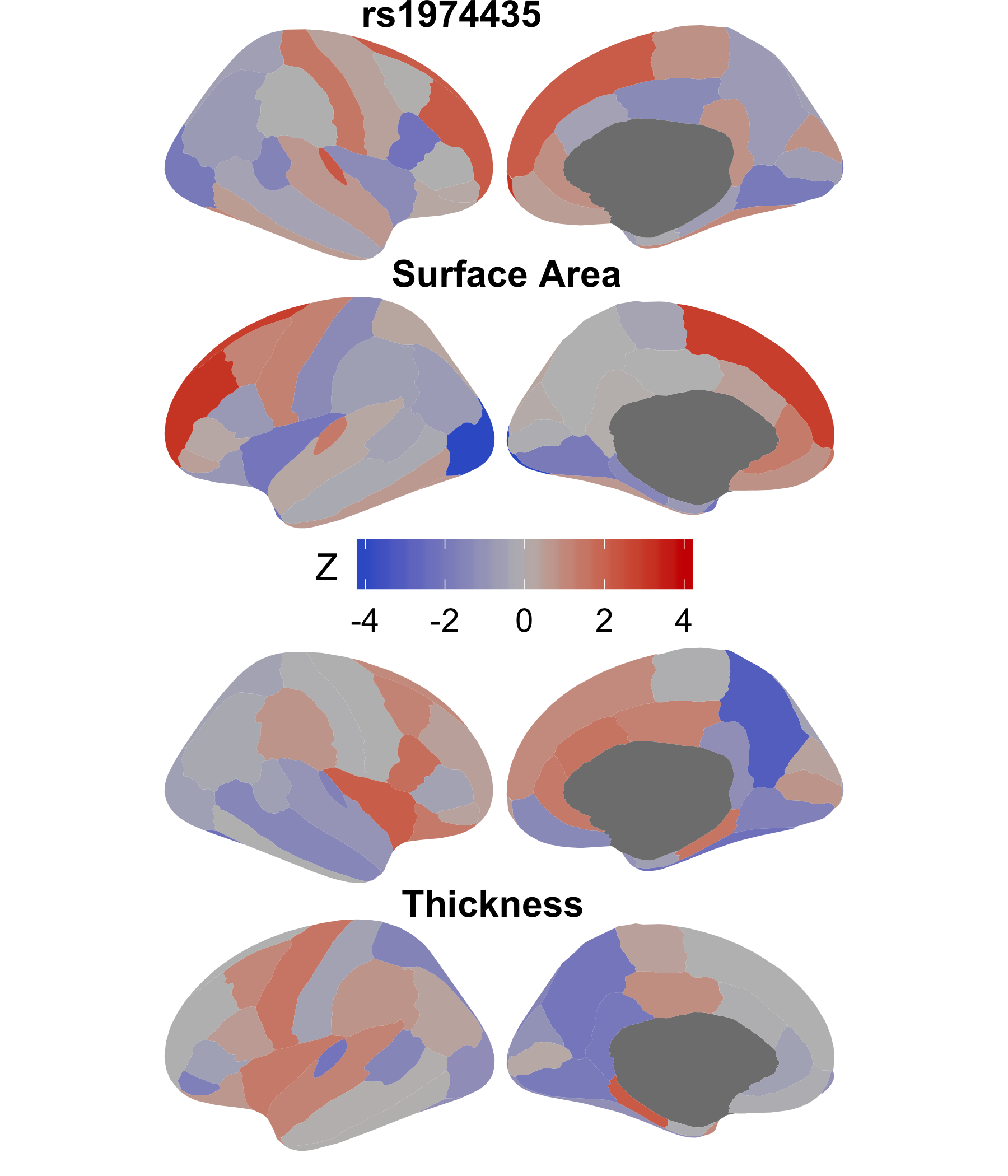

Supplement: Supplementary file 17 — Supplementary Data 14 [file 41467_2020_17368_MOESM17_ESM.gz › BrainMaps/most_aseg_vol/BrainMap149_rs1974435.png]

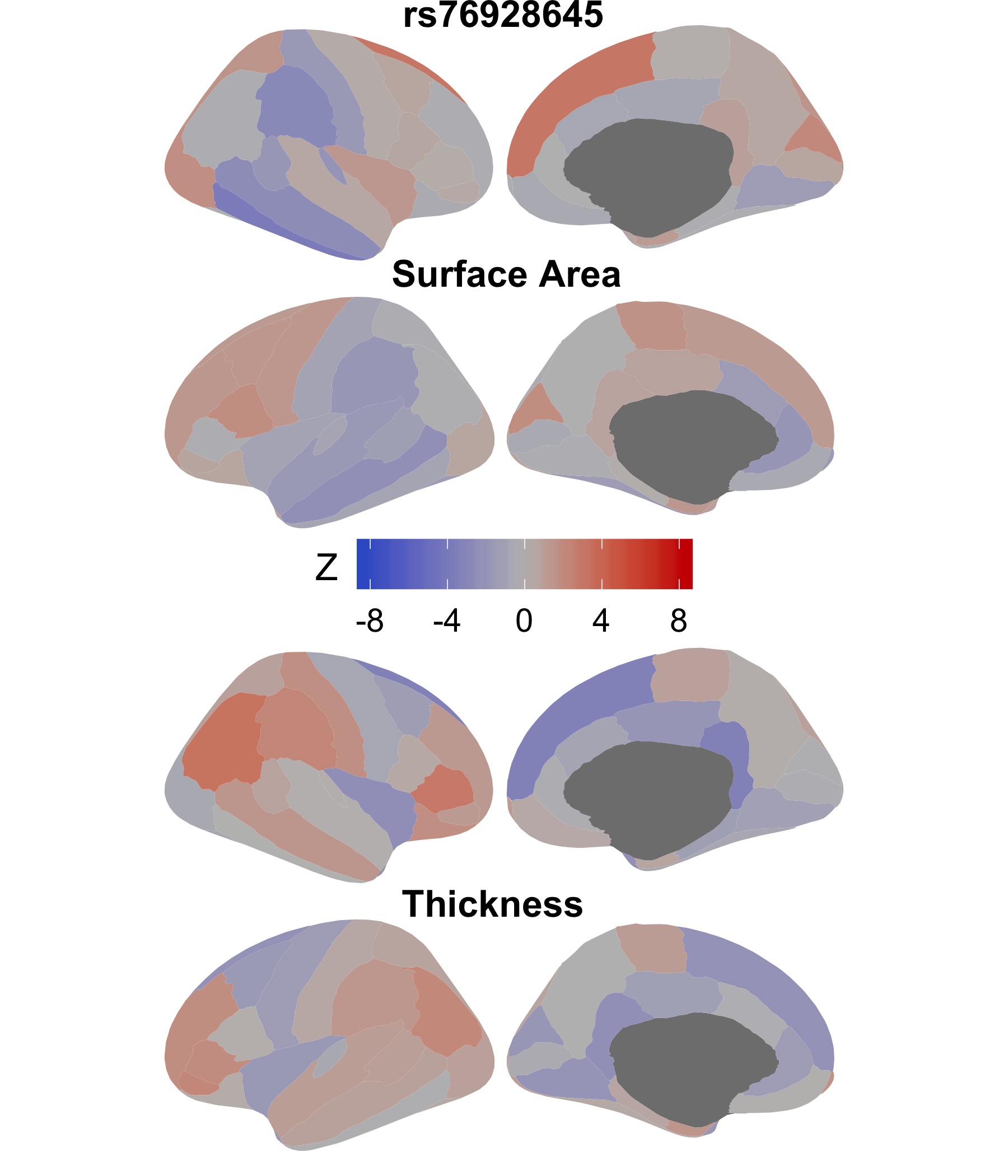

Supplement: Supplementary file 17 — Supplementary Data 14 [file 41467_2020_17368_MOESM17_ESM.gz › BrainMaps/most_aseg_vol/BrainMap014_rs76928645.png]

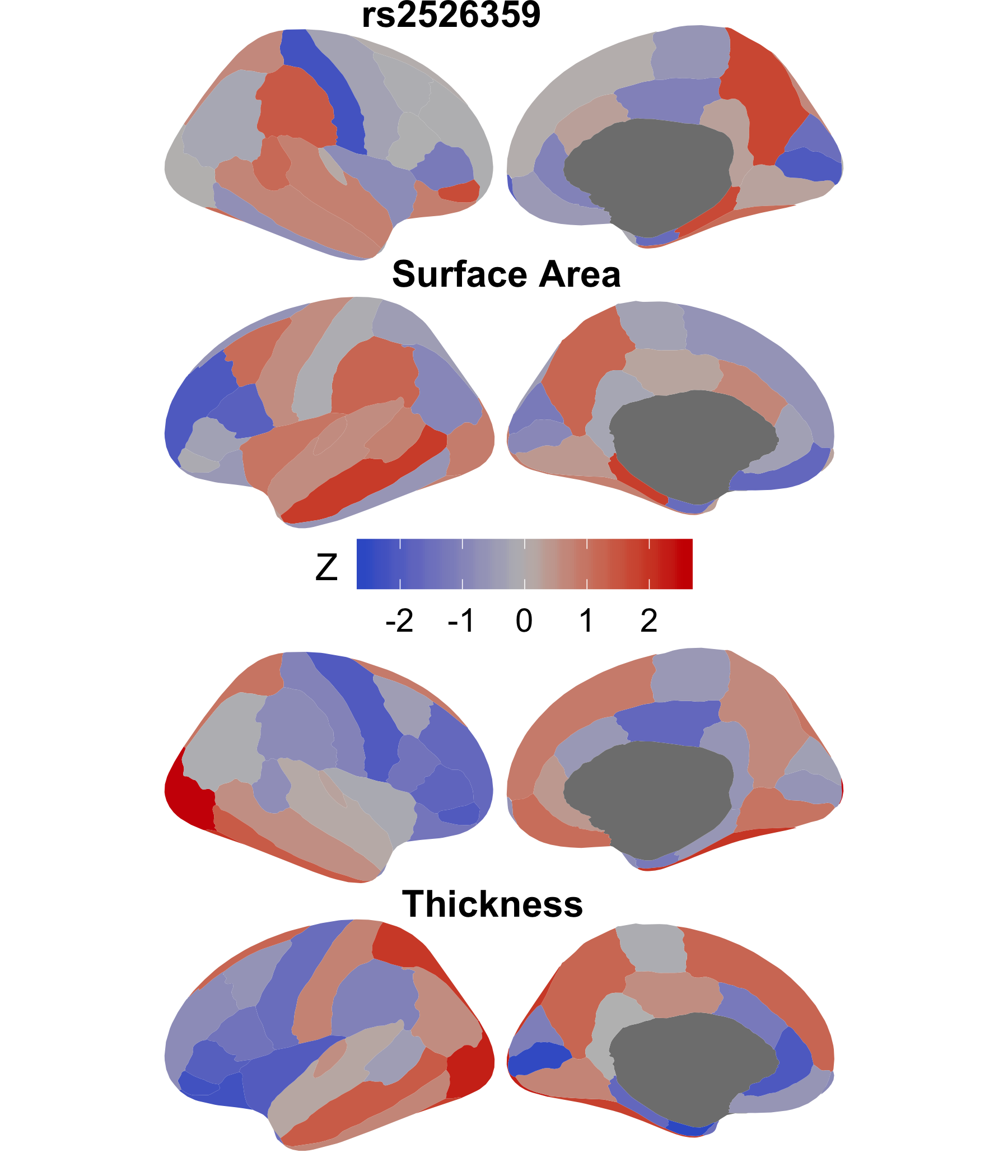

Supplement: Supplementary file 17 — Supplementary Data 14 [file 41467_2020_17368_MOESM17_ESM.gz › BrainMaps/most_aseg_vol/BrainMap156_rs2526359.png]

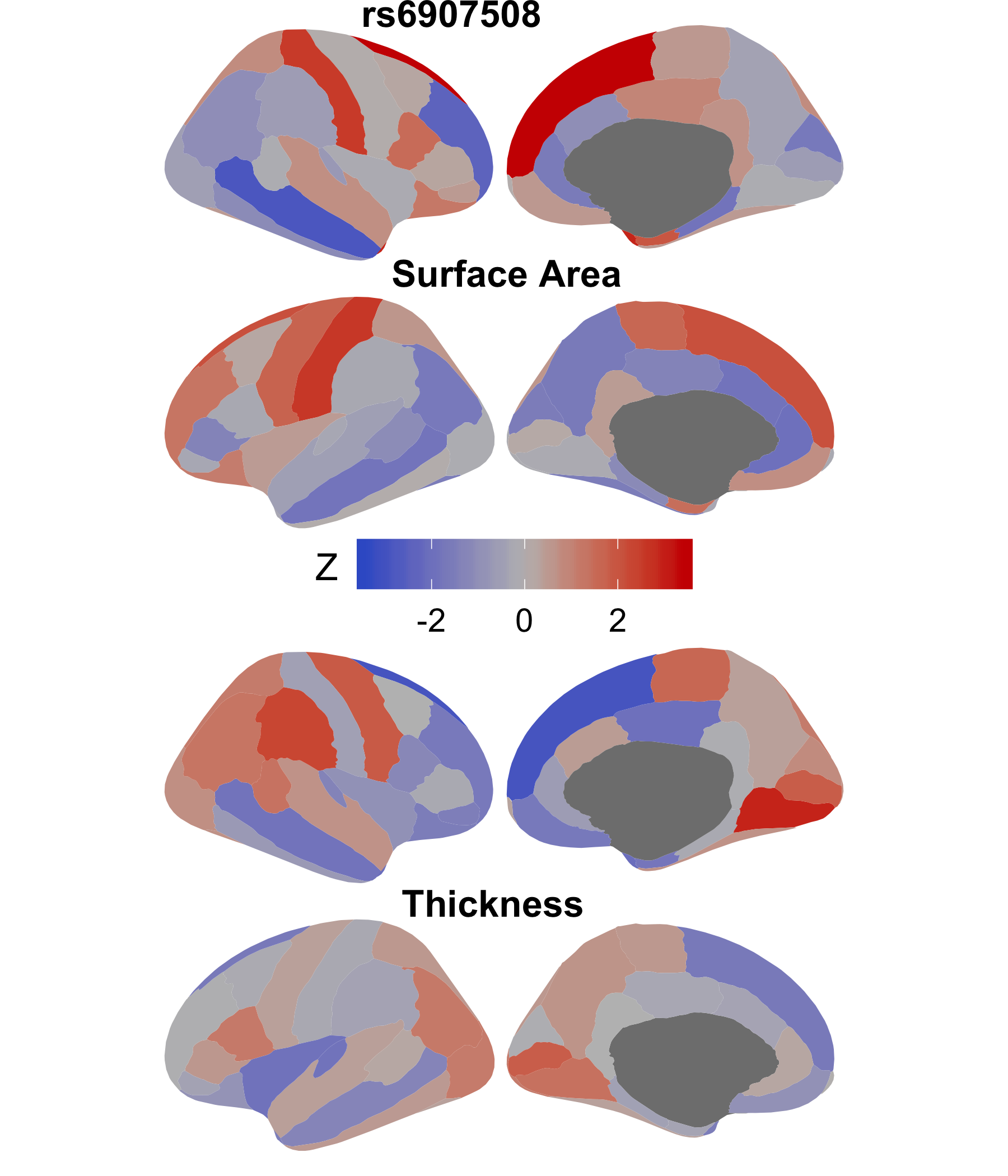

Supplement: Supplementary file 17 — Supplementary Data 14 [file 41467_2020_17368_MOESM17_ESM.gz › BrainMaps/most_aseg_vol/BrainMap170_rs6907508.png]

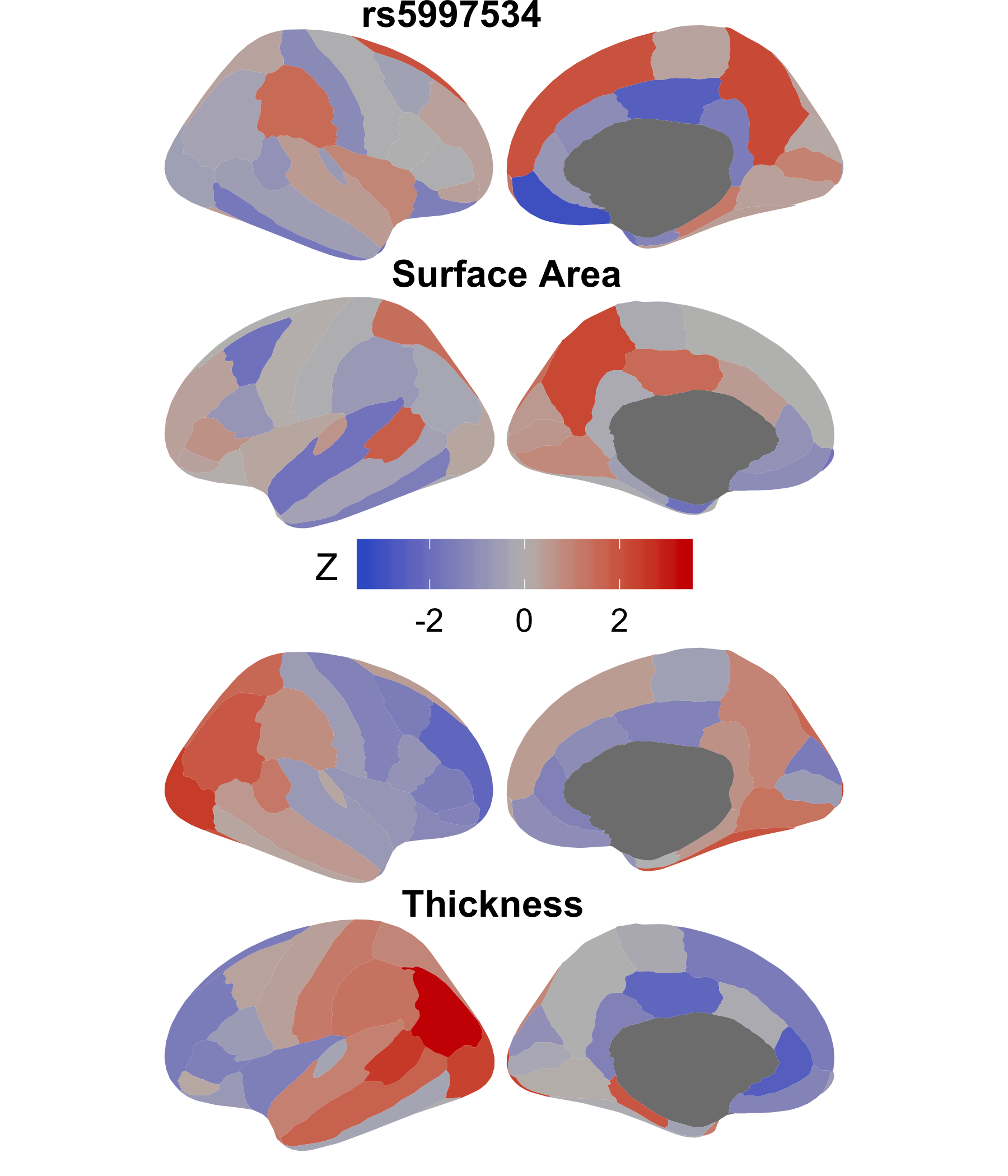

Supplement: Supplementary file 17 — Supplementary Data 14 [file 41467_2020_17368_MOESM17_ESM.gz › BrainMaps/most_aseg_vol/BrainMap162_rs5997534.png]

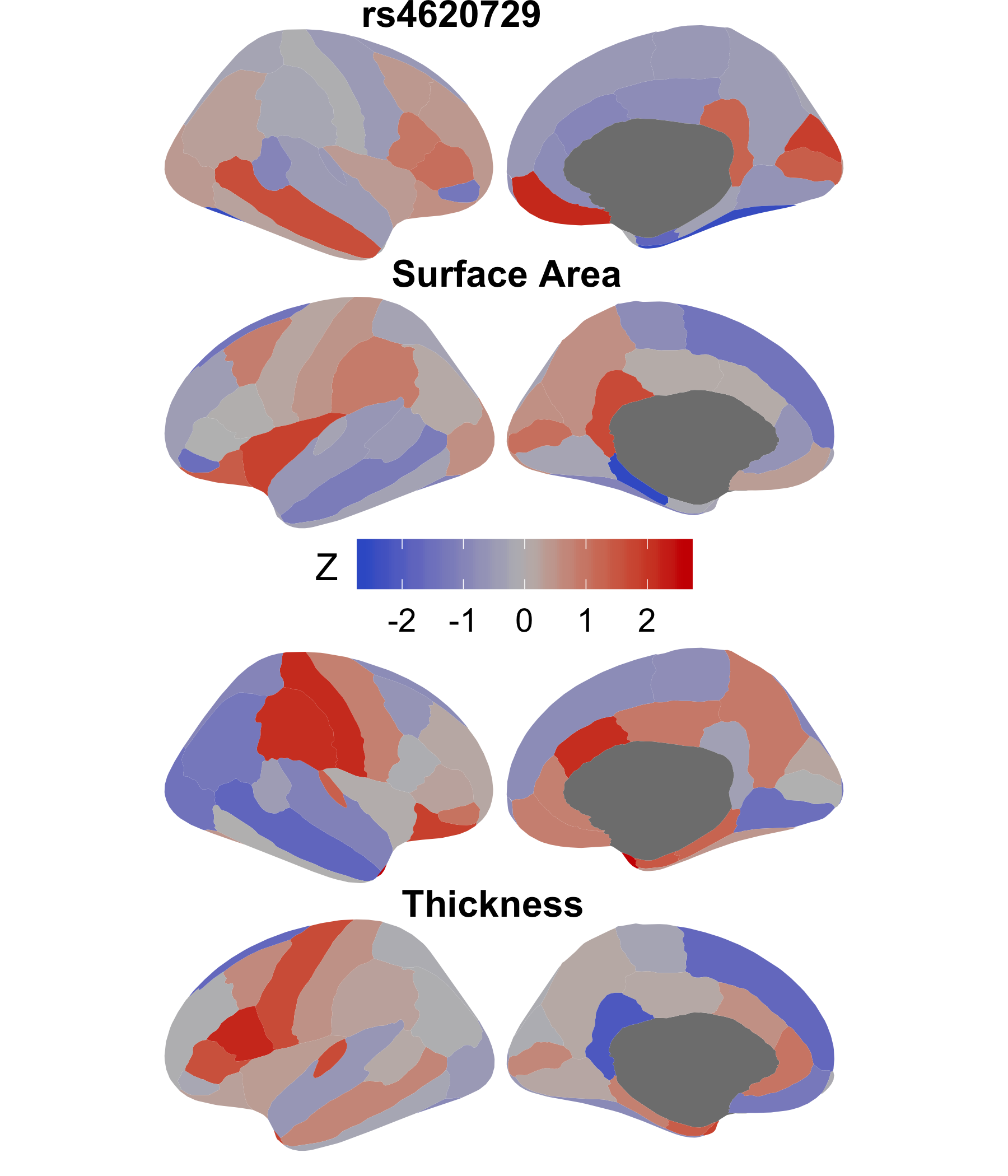

Supplement: Supplementary file 17 — Supplementary Data 14 [file 41467_2020_17368_MOESM17_ESM.gz › BrainMaps/most_aseg_vol/BrainMap099_rs4620729.png]

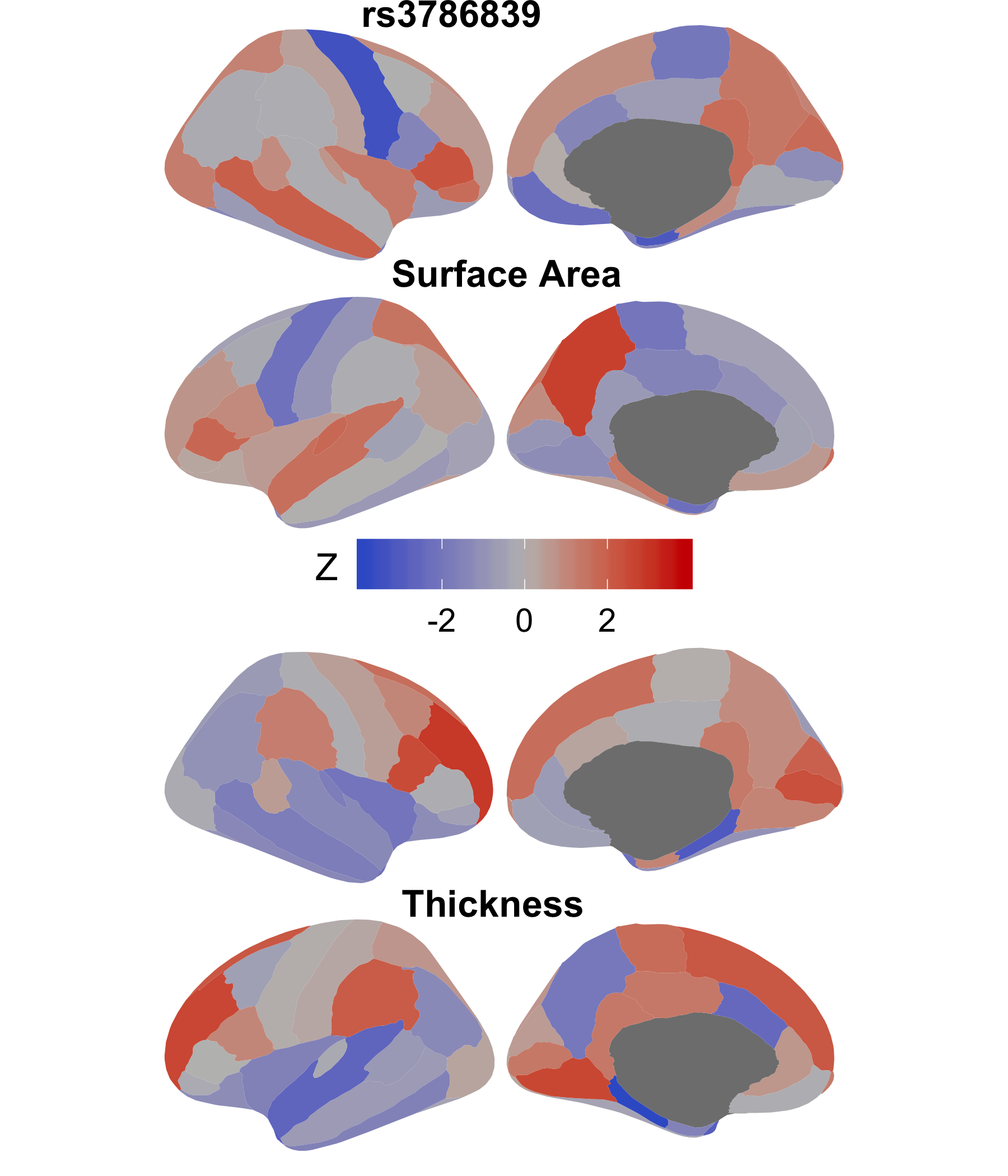

Supplement: Supplementary file 17 — Supplementary Data 14 [file 41467_2020_17368_MOESM17_ESM.gz › BrainMaps/most_aseg_vol/BrainMap105_rs3786839.png]

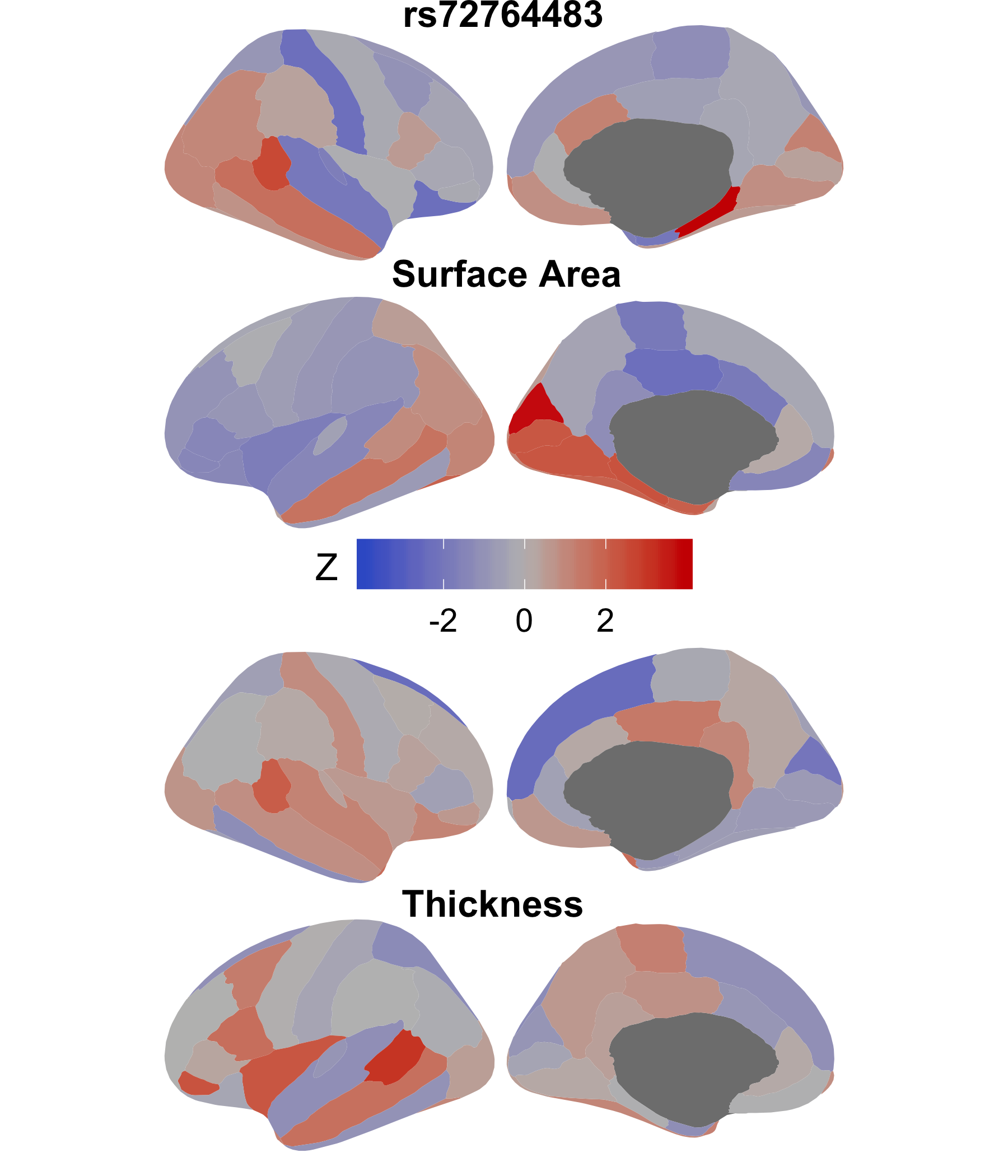

Supplement: Supplementary file 17 — Supplementary Data 14 [file 41467_2020_17368_MOESM17_ESM.gz › BrainMaps/most_aseg_vol/BrainMap023_rs72764483.png]
